# Two Catalytic Annulation Modes via Cu-Allenylidenes with Sulfur Ylides that Are Dominated by the Presence or Absence of Trifluoromethyl Substituents

**DOI:** 10.1016/j.isci.2020.100994

**Published:** 2020-03-20

**Authors:** Malla Reddy Gannarapu, Jun Zhou, Bingyao Jiang, Norio Shibata

**Affiliations:** 1 Departments of Nanopharmaceutical Science & Life Science and Applied Chemistry, Nagoya Institute of Technology, Gokiso, Showa-ku, Nagoya 466-8555, Japan; 2 Institute of Advanced Fluorine-Containing Materials, Zhejiang Normal University, 688 Yingbin Avenue, 321004 Jinhua, China

**Keywords:** Organic Chemistry, Organic Synthesis, Physical Organic Chemistry

## Abstract

We disclose the Cu-catalyzed enantioselective synthesis of 3-methyl-3-propargyl-indolines, which contain a quaternary stereogenic carbon center, via the decarboxylative [4 + 1] annulation of 4-methyl-4-propargyl-benzoxazinanones with variety of sulfur ylides. The reaction proceeds predominantly through a γ-attack at the Cu-allenylidene intermediates by sulfur ylides to provide the corresponding indolines in good yield and high enantioselectivity (up to 91% ee). In contrast, the reaction of 4-trifluoromethyl-4-propargyl-benzoxazinanones with sulfur ylides delivers 3-trifluoromethyl-2-functionalized indoles in good to high yield via an unexpected α-attack at the Cu-allenylidene intermediates. Control over the α/γ-attack at the Cu-allenylidene intermediates by the same interceptors was achieved for the first time by the use of trifluoromethyl substituents.

## Highlights

• Fluorine changes the catalytic decarboxylative annulation modes
• All carbon quarternary stereocentered indolines, up to 91% ee
• An unexpected α-attack at the Cu-allenylidene intermediate with CF_3_
• 3-CF_3_-substituted indoles with a 2-functional group

## Graphical Abstract

**Figure:**
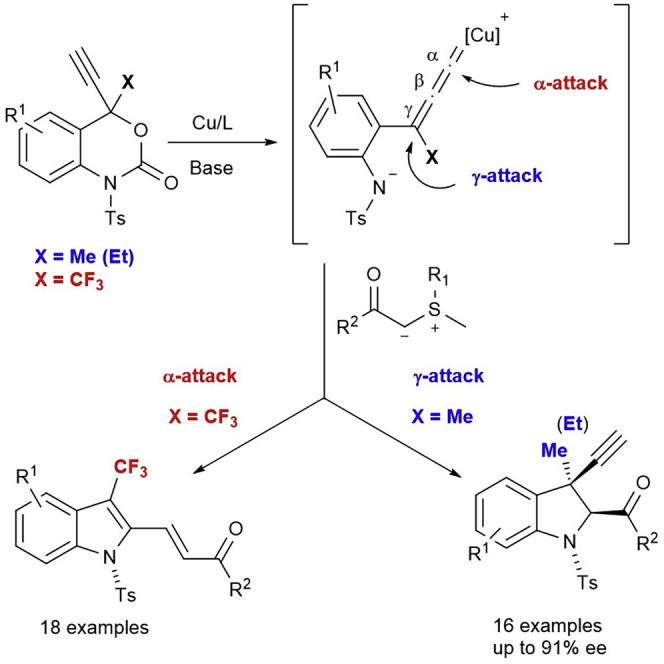

## Introduction

Transition-metal-catalyzed annulation reactions have been extensively investigated, especially in the context of constructing multiply functionalized nitrogen (*N*)-containing heterocycles (D'Souza and Muller, 2007, Gulevich et al., 2013, Nakamura and Yamamoto, 2004, Patil and Yamamoto, 2008, Qiao et al., 2019, Reen et al., 2019, Sole and Fernandez, 2018, Yamamoto, 2014). Indoles and indolines have received a significant amount of that attention, as these heterocycles represent privileged structural fragments in pharmaceuticals and natural products (Sundberg, 1996, Kochanowska-Karamyan and Hamann, 2010, Sharma et al., 2010, Zhang et al., 2011, Kaushik et al., 2013, Ishikura et al., 2015, Mo et al., 2015, Patil et al., 2016, Zeeli et al., 2018, Cacchi and Fabrizi, 2011, Li et al., 2014, Guo et al., 2015, Giorgio, 2017, Liang and Xia, 2017, Mancuso and Dalpozzo, 2018, Huang and Yin, 2019, Silva et al., 2019). Among the multitude of synthetic methods for the preparation of indoles and indolines, we were particularly interested in annulation reactions with 4-propargyl benzoxazinanones (**1**) (Wang et al., 2016, Wang et al., 2018a, Wang et al., 2018b, Wang et al., 2018c, Li et al., 2016, Li et al., 2017, Li et al., 2018, Song et al., 2017, Lu et al., 2017, Lu et al., 2018a, Lu et al., 2018b, Shao and You, 2017, Chen et al., 2018, Jiang et al., 2018, Zhang et al., 2018a, Zhang et al., 2019, Ji et al., 2018, Simlandy et al., 2019, Sun et al., 2019), which were first reported by Xiao, Lu, and co-workers in 2016 (Wang et al., 2016) and have since rapidly attracted attention as attractive reactants for the preparation of *N*-heterocycles via metal-catalyzed annulation reactions (Wang et al., 2016, Wang et al., 2018a, Wang et al., 2018b, Wang et al., 2018c, Li et al., 2016, Li et al., 2017, Li et al., 2018, Song et al., 2017, Lu et al., 2017, Lu et al., 2018a, Shao and You, 2017, Chen et al., 2018, Jiang et al., 2018, Zhang et al., 2018a, Zhang et al., 2019, Ji et al., 2018, Simlandy et al., 2019, Sun et al., 2019). Crucial for annulation reactions involving **1** is the decarboxylative generation of Cu-stabilized allenylidene zwitterionic intermediates (**I**), which can be trapped by suitable interceptors to construct various types of *N*-heterocycles. Accordingly, new types of annulation reactions can be easily developed by judiciously choosing the interceptors.

**Scheme 1:**
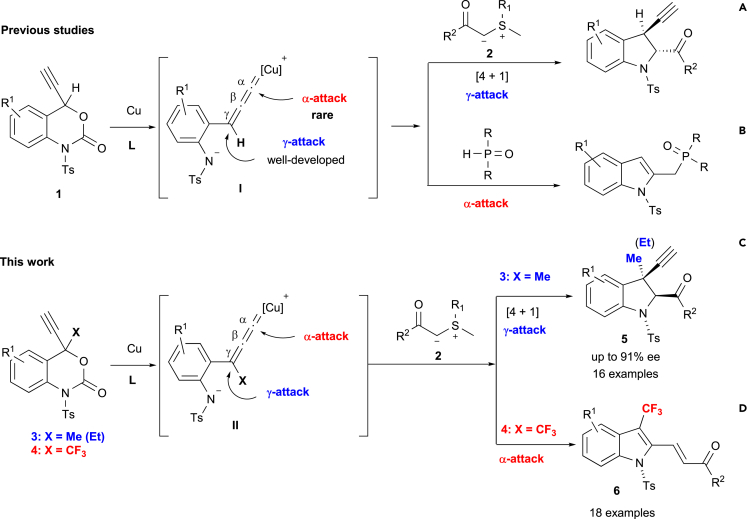
Decarboxylative Annulations of 4-Substituted Benzoxazinanones via Cu-Allenylidene Intermediates (A) and (B): Previous studies. (C) and (D): Present work.

It should be noted that annulation reactions involving **1** may proceed via two different reaction modes as the Cu-allenylidenes of the type **I** contain two reactive electrophilic positions, i.e., α and γ relative to the Cu atom. For example, the decarboxylative [4 + 1] cycloaddition of **1** with sulfur ylides **2** provides enantio-enriched 3-propargyl indolines via the γ-addition (Wang et al., 2016, Wang et al., 2018a, Wang et al., 2018b, Li et al., 2016, Li et al., 2017, Li et al., 2018, Song et al., 2017, Lu et al., 2017, Shao and You, 2017, Chen et al., 2018, Jiang et al., 2018, Zhang et al., 2018a, Zhang et al., 2019, Ji et al., 2018, Simlandy et al., 2019, Sun et al., 2019) of **I** (Scheme 1A) (Wang et al., 2016). Such a α-addition at **I** has been reported for the use of phosphonates as interceptors, which exclusively provides 2-phosphorylmethyl indoles (Scheme 1B) (Wang et al., 2018c). Although the α/γ chemo-selectivity at **I** can be controlled by the interceptors (nucleophiles) as mentioned above, most of these induce γ-addition reactions (Wang et al., 2016, Wang et al., 2018a, Wang et al., 2018b, Li et al., 2016, Li et al., 2017, Li et al., 2018, Song et al., 2017, Lu et al., 2017, Shao and You, 2017, Chen et al., 2018, Jiang et al., 2018, Zhang et al., 2018a, Zhang et al., 2019, Ji et al., 2018, Simlandy et al., 2019, Sun et al., 2019), whereas the α-addition-mode is very rare (Wang et al., 2018c). In other words, controlling the α/γ chemoselectivity at Cu-allenylidene zwitterionic intermediates of the type **I** to induce the α-addition mode remains highly challenging.

Herein, we disclose the first successful attempt to control the α/γ chemo-selectivity at Cu-allenylidene zwitterionic intermediates via a fluorine effect. Specifically, the Cu-catalyzed decarboxylative annulation of non-fluorinated 4-methyl (Me)-4-propargylic benzoxazinanones **3** with sulfur yields **2** furnished chiral non-racemic 3-Me-3-propargyl-indolines **5** in a γ-selective fashion in good to high yield with high enantioselectivity (up to 91% ee; Scheme 1C). As examples of the generation of all-carbon quaternary stereocenters at the propargylic position are rare (Tsuchida et al., 2016, Sanz-Marco et al., 2016, Shemet and Carreira, 2017, Wendlandt et al., 2018, Zhang et al., 2018a, Li et al., 2019, Xu and Hu, 2019), the obtained results might help to activate the corresponding area of research. On the other hand, the α-selective addition was predominantly observed for the Cu-catalyzed decarboxylative annulation of fluorinated variants such as 4-trifluoromethyl (CF_3_)-4-propargylic benzoxazinanones **4** with **2**, which led to the formation of 3-CF_3_-2-functionalized indoles **6** in good to high yield with high *E*/*Z*-selectively via a rare α-attack at the Cu-allenylidene zwitterionic intermediates (Scheme 1D). Given that CF_3_-containing *N*-heterocycles have gained considerable attention in academic and industrial research on pharmaceutics and agrochemicals (Kawai and Shibata, 2014, Engl et al., 2015, Huang et al., 2015, Meyer, 2016, He et al., 2019), CF_3_-substituted indoles **6** that contain 2-functional groups should represent versatile building blocks for the preparation of drug candidates. To the best of our knowledge, this is the first example of controlling the α/γ chemoselectivity at Cu-allenylidene zwitterionic intermediates that does not depend on the interceptor.

## Results and Discussion

### Optimization

**Scheme 2:**
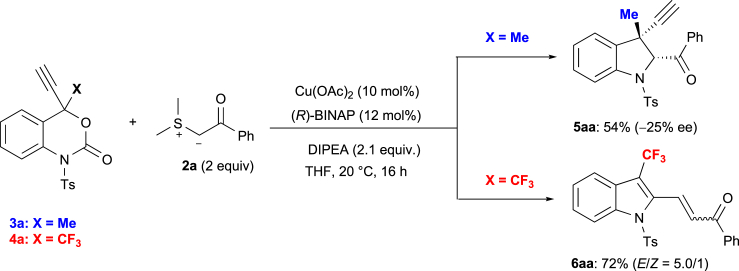
Two Reaction Modes for the Decarboxylative Annulation of 4-Substituted 4-Propargyl-Benzoxazinanones (3, 4) with Sulfur Ylides 2a under Cu Catalysis Conditions

Recently, we reported the Pd-catalyzed decarboxylation of 4-trifluoromethyl benzoxazinanones (Punna et al., 2018, Punna et al., 2019, Das et al., 2018) with sulfur ylides **2** to provide 3-CF_3_-substituted indolines with high diastereoselectivity (Punna et al., 2018). Stimulated by the seminal work of Xiao, Lu, and co-workers (Scheme 1A) (Wang et al., 2016), we were interested in the enantioselective formation of previously unknown 3-propargyl indolines with an all-carbon quaternary stereogenic center such as **5** by the reaction of 4-tetrasubstituted propargyl benzoxazinanones (**3**, **4**) with sulfur ylides **2** via a catalytic decarboxylative [4 + 1] cycloaddition. To our great surprise, the targeted 3-Me-3-propargyl-indoline **5aa** was obtained in 54% yield with 25% ee when we treated 4-Me-4-propargyl benzoxazinanone **3a** with benzoyl sulfur ylide **2a** and *i*-Pr_2_NEt (DIPEA, 2.1 equiv.) in the presence of a catalytic amount of Cu(OAc)_2_ and (*R*)-BINAP in THF. However, when we used 4-CF_3_-4-propargyl benzoxazinanone **4a** instead of **3a** under otherwise identical conditions, we unexpectedly obtained 3-CF_3_-2-substituted indole **6aa** in 72% with a 5/1 *E*/*Z* selectively (Scheme 2).

**Scheme 3:**
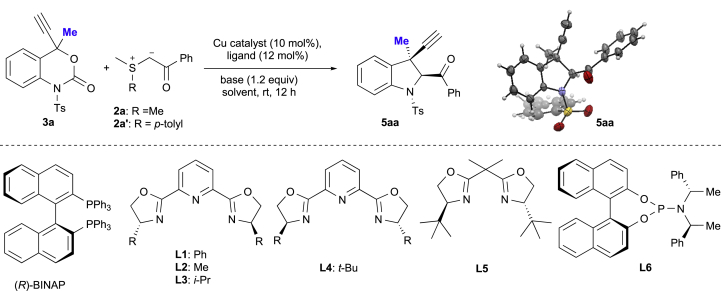
Optimization of the Reaction Conditions for the Cu-Catalyzed [4 + 1] Cycloaddition of 3a with 2a

Encouraged by these unprecedented preliminary results, we initially studied the enantioselective [4 + 1] cycloaddition reaction of 4-Me-propargyl benzoxazinanone **3a** with sulfur ylide **2a** (Scheme 3, Table 1). First, the effect of (*R*)-BINAP on this transformation was examined at room temperature under a variety of conditions (entries 1–4). However, the enantioselectivity of **5aa** was only moderate (up to 44%; entry 2). Subsequently, we focused on the use of Pybox ligands for the improvement of the enantioselectivity in this transformation. After a careful evaluation of chiral ligands, Lewis acids, solvents, and substituents on sulfur ylides **2a** (**2a′**) (entries 5–16; Tables S1–S7), we found that the commercially available *iso-*propyl-substituted Pybox ligand **L3** exhibited the best performance, producing chiral indoline **5aa** in 72% yield with 74% *ee* (entry 10). More details of the screening of other ligands such as **L5** and **L6** are shown in the Supplemental Information (Table S1). An investigation into the solvent effect (Table S3) revealed that dichloromethane (DCM) provided the best reaction efficiency with a slightly lower yield and improved enantiocontrol (entry 12, 69% yield, 78% *ee*). An evaluation of different bases showed that *N*-ethyl morpholine was superior to other bases (entry 13, 84% yield, 82% *ee*). Gratifyingly, a more favorable outcome (85% *ee*) was observed without a significant decrease in yield when the reaction was carried out with 1.5 equiv. of **2a’** (entry 15, 83% yield, 85% *ee*). In all these cases, >95:5 diastereoselectivity was confirmed by a ^1^H NMR analysis of the crude reaction mixture. While the amount of *N*-ethylmorpholine can be reduced to a catalytic amount, the corresponding yield decreased slightly (79% yield, 85% *ee*, entry 16). The absolute configuration of **5aa**, induced by **L3**, was determined to be 2(*S*) and 3(*R*) by a single-crystal *X*-ray diffraction analysis (CCDC1971179). The 2(*S*), 3(*R*)-stereochemistry of **5aa** is a surprise, as we expected the configuration of **5aa** to be 2(*R*),3(*R*) or 2(*S*),3(*S*) based on a previous report (Scheme 1A) (Wang et al., 2016). Ts group on **3a** is important since the reaction of Boc-protected variant of **3a** with **2a′** under the same conditions resulted in a complex mixture.

**Table 1:** Optimization of the Reaction Conditions for the Cu-Catalyzed [4 + 1] Cycloaddition of 3a with 2a

Entry	Ligand	R (2a or 2a′)	Cu	Solvent	Yield (%)^a^	*ee* (%)^b^
1^c^	(*R*)-BINAP	Me (**2a**)	Cu(OAc)_2_	THF	54	−25
2	(*R*)-BINAP	Me (**2a**)	Cu(OAc)_2_	THF	55	−44
3	(*R*)-BINAP	*p*-tolyl (**2a′**)	Cu(OAc)_2_	THF	49	−32
4	(*R*)-BINAP	*p*-tolyl (**2a′**)	Cu(OTf)_2_	THF	31	0
5	L1	Me (**2a**)	Cu(OAc)_2_	THF	59	−38
6	L1	Me (**2a**)	Cu(OTf)_2_	THF	52	42
7	L1	*p*-tolyl (**2a′**)	Cu(OAc)_2_	THF	49	0
8	L1	*p*-tolyl (**2a′**)	Cu(OTf)_2_	THF	30	42
9	L2	*p*-tolyl (**2a′**)	Cu(OTf)_2_	THF	50	56
10	L3	*p*-tolyl (**2a′**)	Cu(OTf)_2_	THF	72	74
11	L4	*p*-tolyl (**2a′**)	Cu(OTf)_2_	THF	63	−46
12	L3	*p*-tolyl (**2a′**)	Cu(OTf)_2_	DCM	69	78
13^d^	L3	*p*-tolyl (**2a′**)	Cu(OTf)_2_	DCM	84	82
14^d^	L3	Me (**2a**)	Cu(OTf)_2_	DCM	79	63
15^d^^,^^e^	L3	*p*-tolyl (**2a′**)	Cu(OTf)_2_	DCM	83	85
16^d^^,^^e^^,^^f^	L3	*p*-tolyl (**2a′**)	Cu(OTf)_2_	DCM	79	85

a Determined by a ^1^H NMR analysis of the crude reaction mixture using 1,3,5-trimethoxybenzene as the internal standard.

b Determined by a chiral HPLC analysis.

c Using *i*-Pr_2_NEt (2.1 equiv.).

d Using *N*-ethylmorpholine.

e Using **2a’** (0.15 mmol).

f Using 0.015 mmol of *N*-ethylmorpholine.

### Substrate Scope and Synthetic Application I

**Scheme 4:**
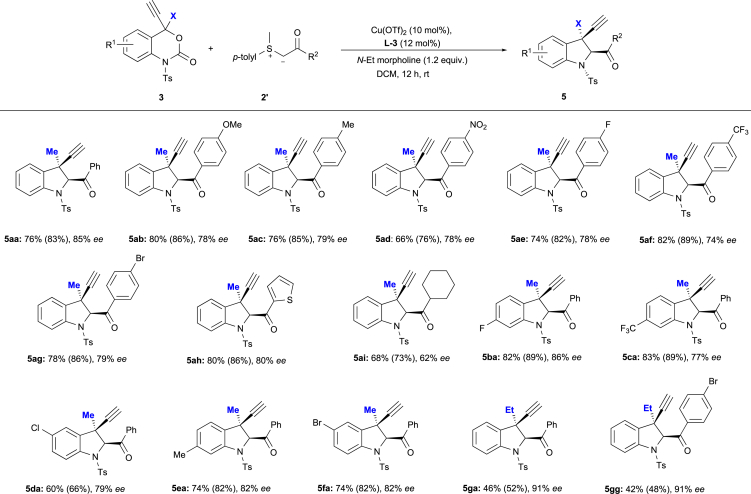
Substrate Scope for 4-Propargyl Benzoxazinanones 3a-3g and Sulfur Ylides 2a′-2i′ for the Formation of 5aa-5gg via a Decarboxylative [4 + 1] Cycloaddition Experiments were carried out using **3** (0.1 mmol), **2'** (0.15 mmol), Cu(OTf)_2_ (10 mol %), **L3** (12 mol %), and *N*-ethyl morpholine (0.12 mmol) in dry DCM (1.0 mL). Isolated yields are shown together with ^1^H NMR yields (in parenthesis; using 1,3,5-trimethoxybenzene as the internal standard). In all cases, the diastereomeric ratio of the products **5** was >95:5. The *ee* values were determined based on a chiral HPLC analysis.

With the optimal reaction conditions for the enantioselective formation of **5** in hand (Table 1, entry 15), the scope of this reaction with respect to the sulfur ylides was examined by treating 4-Me-4-propargyl benzoxazinanone **3a** with **2b′–2i’** (Scheme 4). All ylide derivatives **2′** were well tolerated under the applied reaction conditions and delivered the desired products (**5ab**–**5ai**) in moderate to good yield (≤82%) with decent enantioselectivity (62%–80% *ee*). Substrates bearing electron-withdrawing groups such as 4-NO_2_ (**2d′**) or 4-CF_3_ (**2f′**) afforded the desired products in good yield with moderate enantioselectivity (**5ad**: 66%, 78% *ee*; **5af**: 82%, 74% *ee*). Furthermore, both electron-donating and -withdrawing substituents are tolerated in this reaction and exert only a minimal effect on the enantioselectivity (74%–79% *ee*). Particularly, heteroaromatic sulfur ylide **2h′** also smoothly produces the desired product in high yield (**5ah**, 80%) with a good enantioselectivity (80% ee). Cyclohexyl-substituted sulfur ylide **2i′** also delivers the corresponding product (**5ai**) in decent yield (68%) with moderate enantioselectivity (62% ee). Next, we examined the substrate scope with respect to the 4-Me-4-propargyl benzoxazinanones by treating **3a–3f** with sulfur ylide **2a’** (Scheme 4). The introduction of the substituent at different positions of the benzoxazinanone moiety resulted in higher levels of enantioselectivity (77%–86% *ee*). The variation of the substituent pattern exerts a subtle impact on the selectivity. For instance, substrates bearing halogen substituents such as 7-F (**3b**), 6-Cl (**3d**), or 6-Br (**3f**) smoothly furnish the desired products (**5b**, **5d**, and **5f**) in moderate to good yield (60%–82%) with good enantioselectivity, albeit that the product yield is lower for 6-Br substitution than for 6-Cl substitution. A substrate bearing an electron-withdrawing group (**3c**: 7-CF_3_) delivered the corresponding product in good yield with good enantioselectivity (**5ca**: 83%, 77% *ee*). Furthermore, a benzoxazinanone with an electron-donating group (**3e**: 7-Me) yielded the desired product in good yield with high enantioselectivity (**5ea**: 74%, 82% *ee*). To understand the effect of the 4-Me substitution of **3** on this transformation, we carried out the same reactions using 4-ethyl (Et)-4-propargyl benzoxazinanone **3g** instead of 4-Me-substituted **3a**. To our satisfaction, the reaction of **3g** with sulfur ylides **2a′** and **2g′** under standard conditions resulted in the formation of the desired products in acceptable yield with excellent enantioselectivity (**5ga**: 46%, 91% *ee*; **5gg**: 42%, 91% *ee*). The increased steric demand at the propargylic position (Me→Et) presumably improves the enantioselectivity under concomitant decrease of the reactivity.

**Scheme 5:**
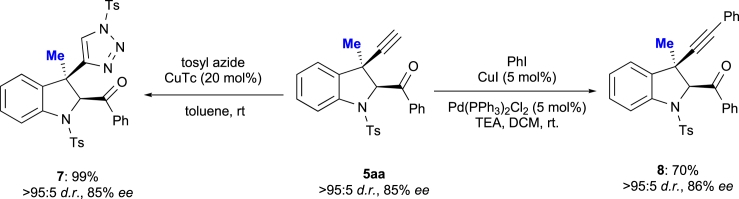
Derivatization of 5; Transformations of 5aa to 7 and 8

To demonstrate the synthetic utility of the 3-propargyl indoline products **5**, we carried out two subsequent transformations (Scheme 5). Optically active indoline **5aa** was smoothly converted into triazole **7** via a 1,3-dipolar cycloaddition with tosyl azide in the presence of CuTc. As expected, **7** was formed in 99% yield without any loss of enantiopurity (85% *ee*). Furthermore, a Sonogashira coupling of **5aa** with iodobenzene afforded the disubstituted alkyne **8** in 70% yield under retention of its enantiopurity.

### Optimization, Substrate Scope, and Synthetic Application II

**Scheme 6:**
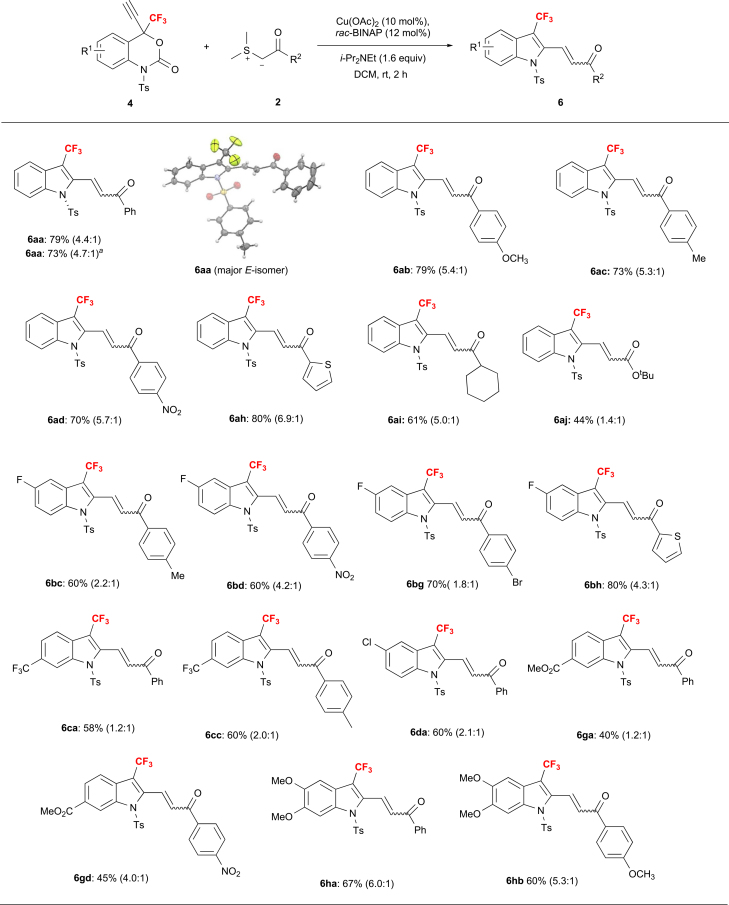
Substrate Scope with Respect to CF_3_-Propargyl Benzoxazinanones 4a-4h and Sulfur Ylides 2a-2j for the Formation of 6aa-6hb via a Decarboxylative Annulation Gram scale reaction using **4a** (1.185 g, 3.0 mmol) was performed. The *E/Z* ratio was determined by ^19^F NMR spectroscopy on the isolated products (in parenthesis). Experiments were carried out using **4** (0.1 mmol), **2** (0.2 mmol), Cu(OAc)_2_ (10 mol %), *rac*-BINAP (12 mol %), and i-Pr_2_NEt (0.16 mmol) in dry DCM (2.0 mL).

Next, we focused our attention on the unexpected annulation observed for the reaction between 4-CF_3_-4-propargyl-benzoxazinanone **4a** and **2a**. As mentioned in Scheme 2, the formation of, e.g., **5a**, i.e., the product of a γ-attack on the indoline, was not observed, and 2-functionalized indole **6aa** was obtained instead. After an extensive screening of combinations of copper catalysts, ligands, bases, and solvents (Tables S8 and S9), we identified the optimal conditions as: dimethyl-sulfur ylide **2**, Cu(OAc)_2_ (10 mol%), *rac*-BINAP (12 mol%), and *i*-Pr_2_NEt (1.6 equiv.) in DCM at rt. Ts group on **4a** is again important since the reaction of Boc-protected variant of **4a** with **2a** under the same conditions resulted in no reaction. The substrate scope for the reaction between CF_3_-propargyl benzoxazinanones **4** and sulfur ylides **2** for the formation of **6** is shown in Scheme 6. A variety of substituted sulfur ylides **2** are suitable for this transformation and smoothly produce the corresponding 3-CF_3_-indole products **6**. Sulfur ylides with either electron-donating groups (**2b**: 4-OMe; **2c**: 4-Me) or a -withdrawing group (**2d**: 4-NO_2_) furnish the corresponding 3-CF_3_-indoles in good yield (**6ab**, 79%; **6ac**, 73%; **6ad**, 70%) with a good *E/Z* ratio (≥5.3:1). Heteroaromatic sulfur ylide **2h** also smoothly produces the desired product in high yield (**6ah**, 80%) with a good *E/Z* ratio (6.9:1). Notably, cyclohexyl-substituted sulfur ylide **2i** also delivers the corresponding product (**6ai**) in moderate yield (61%). Remarkably, sterically demanding *t*-Bu ester sulfur ylide **2j** also provided corresponding product (**6aj**) with acceptable yield (44%) and *E/Z* ratio (1.4:1). Furthermore, we examined the reaction scope with respect to 4-CF_3_-4-propargyl benzoxazinanones **4** under the aforementioned reaction conditions. Substrates with electron-withdrawing groups on the benzene ring, such as 7-CF_3_ (**4c**) or 6-Cl (**4d**) efficiently produced the desired products in moderate yield (**6ca**: 58%; **6da**: 60%) with a low *E/Z* ratio (≤2.1:1). When 6,7-di-OMe-substituted benzoxazinanone **4h** was treated with sulfur ylides **2a** or **2b**, the corresponding products were obtained in good yield (**6ha**: 67%; **6hb**: 60%) with an improved *E/Z* ratio (≥5.3:1). In addition, the reaction of 6-F-substituted **4b** with sulfur ylides **2c**, **2d**, **2g**, and **2h** provided the desired products in moderate to good yield and *E/Z* ratio (**6bc**: 60%; **6bd**: 60%; **6bg**: 70%; **6bh**: 80%). It should be noted here that the introduction of a reactive ester moiety at the 7-position of benzoxazinanone also yielded the desired products in acceptable yield (**6ga**: 40%; **6gd**: 45%) with a moderate *E/Z* ratio. We further carried out a reaction of **4a** with **2a** on the gram scale using the optimal reaction conditions, which afforded **6aa** in 73% yield. The configuration of the major isomer (*E*) was determined based on an *X*-ray diffraction analysis of single crystals of **6aa** (CCDC1971178, Scheme 6). The configuration of the other indole products was accomplished by comparison.

**Scheme 7:**
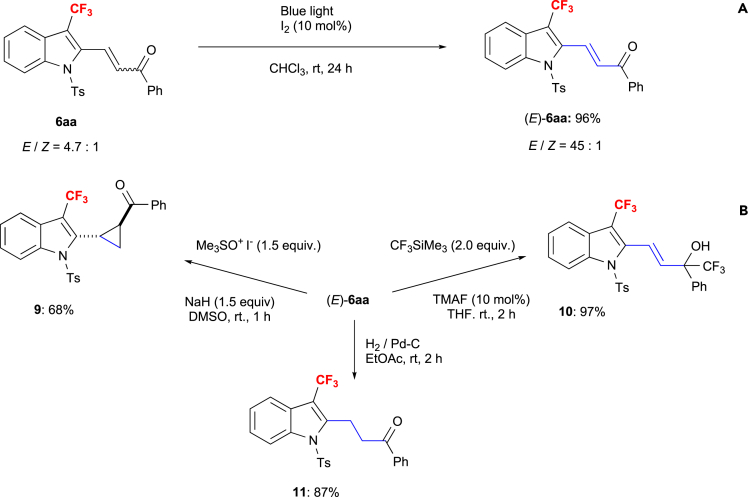
Transformations of 6aa (A) Photolytic isomerization of the *E*/*Z* isomers of **6aa** into predominantly the *E* isomer. (B) Cyclopropanation of **(*E*)-6aa**; 1,2-chemoselective addition of CF_3_SiMe_3_; hydrogenation of **(*E*)-6aa**.

While the 3-CF_3_-2-functionalized indoles were obtained as a mixture of *E/Z* isomers, the isomerization to the *E* isomer proceeded smoothly upon treatment of, e.g., **6aa** with iodine under irradiation with blue light (96% yield; Scheme 7A). Moreover, we performed a couple of transformations of **6aa** to demonstrate the utility of the functionalized CF_3_-indoles **6** (Scheme 7B). First, the cyclopropanation of (*E*)-**6aa** via a Corey-Chaykovsky reaction furnished cyclopropane **9** in 68% yield. A 1,2-selective trifluoromethylation of (*E*)-**6aa** with CF_3_-SiMe_3_ in the presence of a catalytic amount of tetramethylammonium fluoride (TMAF) provided trifluoromethyl-carbinol derivative **10** in 97% yield. Pd–C catalytic hydrogenation of (*E*)-**6aa** provided indole ketone **11** in 87% yield.

**Scheme 8:**
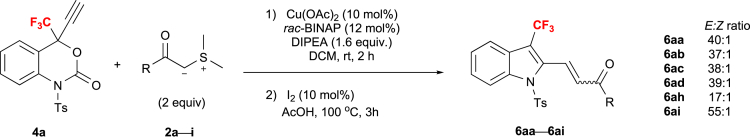
Single Step Formation of 6aa-6ai into Predominantly the *E* Isomer

Furthermore, we examined the reaction conditions to generate the indole product **6** with major *E* isomer. As mentioned in Scheme 8, the formation of the indole product **6** (standard reaction condition) and *E/Z* isomerization were achieved in concerted manner (Scheme 8).

### Proposed Reaction Mechanisms

**Figure 1:**
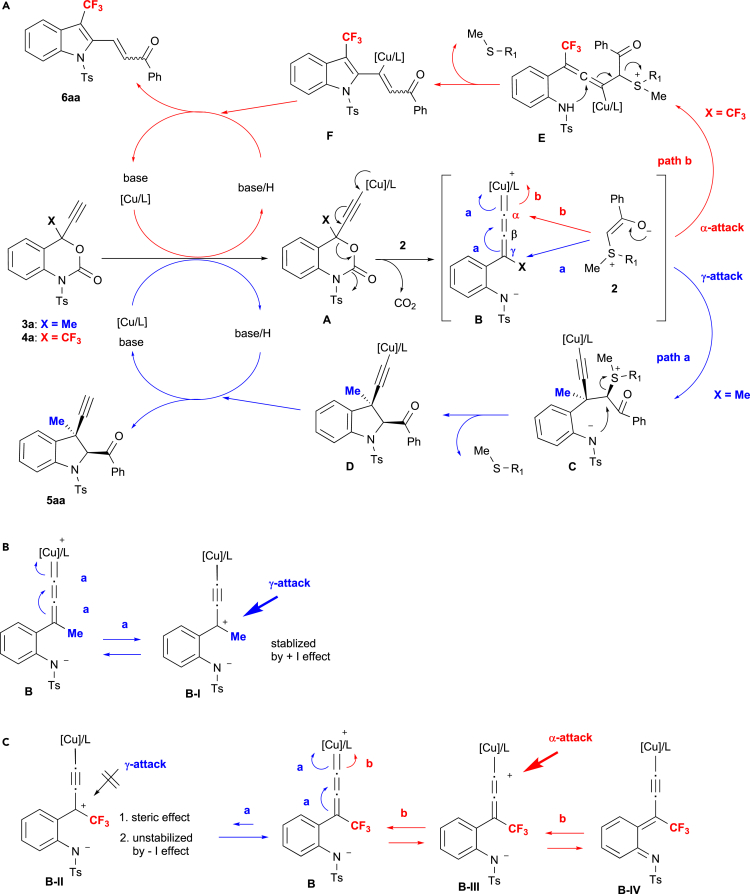
Feasible Reaction Mechanism (A) Two modes of the reaction mechanism are proposed for the catalytic decarboxylative annulation via Cu-allenylidene intermediates **B**. (B) Stabilization of the γ-cation of Cu-allenylidene **B-I** by the Me group. (C) Destabilization of the γ-cation by the CF_3_ group and steric blocking of the nucleophiles in **B-II**, whereas α-vinyl cation intermediate **B-III** might be stabilized by the resonance induced by the CF_3_ group.

Based on the observed experimental results and previous reports (Wang et al., 2016, Wang et al., 2018a, Wang et al., 2018b, Wang et al., 2018c, Li et al., 2016, Li et al., 2017, Li et al., 2018, Song et al., 2017, Lu et al., 2017, Lu et al., 2018a, Shao and You, 2017, Chen et al., 2018, Jiang et al., 2018, Zhang et al., 2018a, Zhang et al., 2018b, Zhang et al., 2019, Ji et al., 2018, Simlandy et al., 2019, Sun et al., 2019), we would like to propose a feasible mechanism to rationalize the chemo/stereoselective formation of indolines/indoles from 4-substituted 4-propargyl benzoxazinanones (**3**, **4**) with sulfur ylides **2** (**2′**) (Figure 1A). As described in Figure 1A, the Cu complex initially activates the propargyl benzoxazinanone (**3a** or **4a**) in the presence of a base to generate Cu−acetylide **A**. Then, the Cu-allenylidene zwitterionic intermediate **B**, which is stabilized by its resonance form, is generated via an extrusion of CO_2_. Depending on the substitution pattern at the propargylic position of the Cu-stabilized allenylidene zwitterionic intermediate **B**, the sulfur ylide **2** attacks at the γ- (X = Me) or α-position (X = CF_3_). The Me-substitution at the propargylic position of transient species **B** allows sulfur ylide **2a** to attack at the γ-position (propargylic position) to generate intermediate **C**, which further converts into copper-containing cycloadduct **D** via an intramolecular SN_2_ reaction. Finally, 3-Me-3-propargyl indoline **5aa** is produced through a proton transfer under concomitant regeneration of the copper catalyst to close the catalytic cycle. The 2,3-*cis*-selectivity of alkyne and benzoyl groups in **5aa** could be explained by the bulkiness of 4-methyl group (C_sp3_ group) rather than 4-alkynyl moiety (C_sp_ group). On the other hand, in the unprecedented catalytic reaction of 4-trifluoromethyl 4-propargyl benzoxazinanone **4a** with sulfur ylide **2a**, the α-addition of sulfur ylide **2a** to transient species **B** should afford intermediate **E**. Finally, **6aa** is furnished through the subsequent intramolecular addition/sulfide elimination from **E**, followed by protolysis of intermediate **F** under regeneration of the Cu catalyst in the final stage.

Although the reasons for the noticeable α/γ-selectivity depend on the 4-substitution in 4-propargyl benzoxazinanones **3** (Me) and **4** (CF_3_) remain obscure at present, the α/γ-selectivity could potentially be rationalized in terms of stabilization and steric effects of the reactive intermediates. Specifically, the Cu-stabilized allenylidene zwitterionic intermediate **B**, which contains a Me group, has a resonance structure **B-I**, in which the carbocation is stabilized by the positive inductive (+I) effect of the Me group. Thus, nucleophilic **2** approaches the γ-position of Cu-allenylidene intermediate **B** (Figure 1B). In the case of **4a**, however, the similar intermediate carbocation **B-II**, generated from the Cu-stabilized allenylidene zwitterionic intermediate **B** with a CF_3_ group, is not stabilized by the strong electron-withdrawing effect of the CF_3_ group, whereas the vinyl cation in intermediate **B-III** is stabilized by the additional resonance structure **B-IV** induced by the electron-withdrawing effect of the CF_3_ substituent. Moreover, the γ-attack should also be unfavorable owing to the steric demand of the bulky CF_3_ group. All of the aforementioned aspects should favor the unprecedented α-attack (Figure 1C).

### Conclusion

In conclusion, we have constructed optically active indolines **5**, which contain an all-carbon quaternary stereocenter, in good yield with high enantioselectivity from the decarboxylative [4 + 1] annulation of Me-propargyl benzoxazinanones **3** and sulfur ylides **2**. Irrespective of the substituents on **3** and **2**, the reaction yielded the corresponding indoline derivatives **5** with excellent enantioselectivity (up to 91% *ee*) via a γ-attack on a Cu-allenylidene zwitterionic intermediate. Interestingly, the reaction between CF_3_-propargyl benzoxazinanones **4** and **2** delivered indole derivatives **6** in good yield via an unprecedented α-attack on the Cu-allenylidene zwitterionic intermediate. In their entirety, these results represent the first example of controlling two modes (α- versus γ-attack) of decarboxylative annulation of propargyl benzoxazinanones via Cu-allenylidenes with the same interceptors. With respect to the importance for research in the area of *N*-containing heterocycles, enantio-enriched indolines with all-carbon quaternary propargyl stereogenic center and CF_3_-substituted indoles with a 2-functional group are both extremely useful precursors in medicinal chemistry. Further investigations into unique reaction patterns that are dominated by fluorine-containing groups and non-fluorinated groups are currently in progress in our laboratories.

### Limitations of the Study

**Figure 2:**
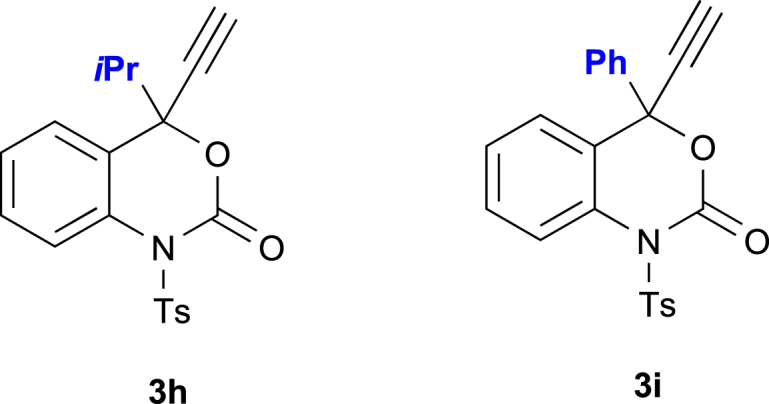
Other 4-Substituted Benzoxazinanones, 4-Isopropyl (3h) and 4-Phenyl (3i) Analogues

The *N*-tosyl group of 4-propargyl benzoxazinanones (**3**, **4**) is crucial for this two-mode of transformations, and the *N*-Boc-protected variants of them under the same conditions resulted in complex mixtures. Other 4-substituted benzoxazinanones such as 4-isopropyl (**3h**) and 4-phenyl (**3i**) analogs (Figure 2) were unsuccessful in generating desired annulation products. The reactions using 4-isopropyl (**3h**) and 4-phenyl (**3i**) variants gave very different products. The preliminary results were shown in Supplemental Information (Figure S1), and further extension is under consideration.

## Methods

All methods can be found in the accompanying Transparent Methods supplemental file.

## Data and Code Availability

Crystallographic data for the structures reported in this article have been deposited at the Cambridge Crystallographic Data Center (CCDC) under accession numbers CCDC 1971179 (**5aa**) and of CCDC1971178 (**6aa**). Copies of the data can be obtained free of charge from www.ccdc.cam.ac.uk/structures/.

## Supplemental Information

Document S1. Transparent Methods, Figures S1–S170, Schemes S1–S8, and Tables S1–S10

## Footnotes

Supplemental Information can be found online at https://doi.org/10.1016/j.isci.2020.100994.
