# Supplementary material for: Two Catalytic Annulation Modes via Cu-Allenylidenes with Sulfur Ylides that Are Dominated by the Presence or Absence of Trifluoromethyl Substituents
Source: iScience. 2020 Mar 20;23(4):100994. doi: 10.1016/j.isci.2020.100994 (PMC7132161; doi:10.1016/j.isci.2020.100994)
Supplement: Document S1. Transparent Methods, Figures S1–S170, Schemes S1–S8, and Tables S1–S10 [file mmc1.pdf]

iScience, Volume *23*

Supplemental Information

Two Catalytic Annulation Modes via Cu-Allenylidenes with Sulfur Ylides that Are Dominated by the Presence or Absence of Trifluoromethyl Substituents

Malla Reddy Gannarapu, Jun Zhou, Bingyao Jiang, and Norio Shibata

Supplemental Figures:

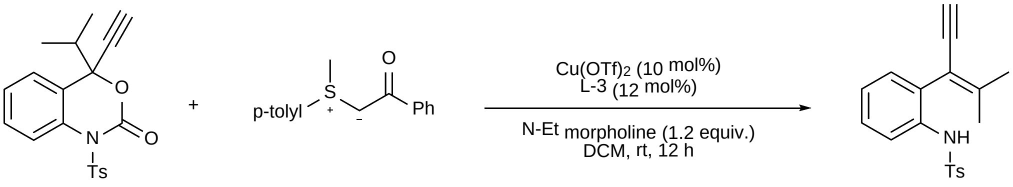

3h 2a' 5ha

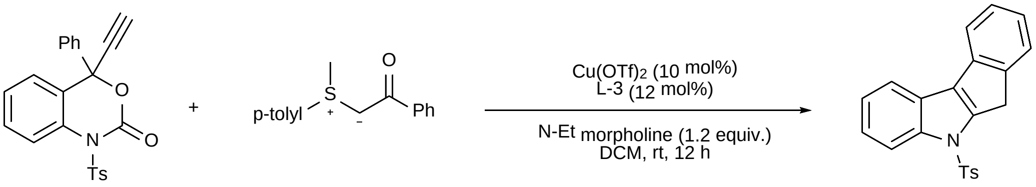

3i 2a' 5ia

Figure S1: Implementation of [4+1] cyclo addition reaction to other 4-substituted benzoxazinanones, related to **Figure 2**

Supplemental Figures for HPLC spectra

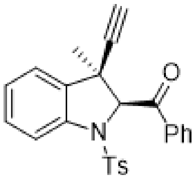

((2*S*,3*R*)-3-ethynyl-3-methyl-1-tosylindolin-2-yl)(phenyl)methanone (**5aa**)

HPLC using CHIRALPAK® IC (*n*-hexane/isopropanol = 95.0/5.0, flow rate 1.0 mL/min, λ=254 nm)

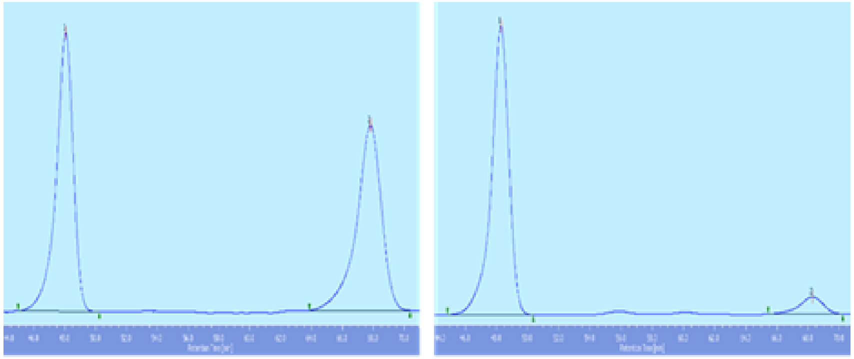

No.	t_R (min)	Area (%)	Height (%)
1	48.058	50.748	60.073
2	67.792	49.252	39.927

No.	t_R (min)	Area (%)	Height (%)
1	48.275	92.637	94.408
2	68.258	7.363	5.592

Figure S2. HPLC spectrum of **5aa**, related to **Scheme 4**.

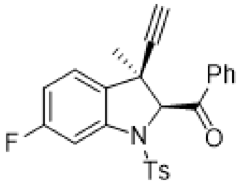

((2*S*,3*R*)-3-ethynyl-6-fluoro-3-methyl-1-tosylindolin-2-yl)(phenyl)methanone (**5ba**)

HPLC using CHIRALPAK® IC (*n*-hexane/isopropanol = 95.0/5.0, flow rate 1.0 mL/min, λ=254 nm)

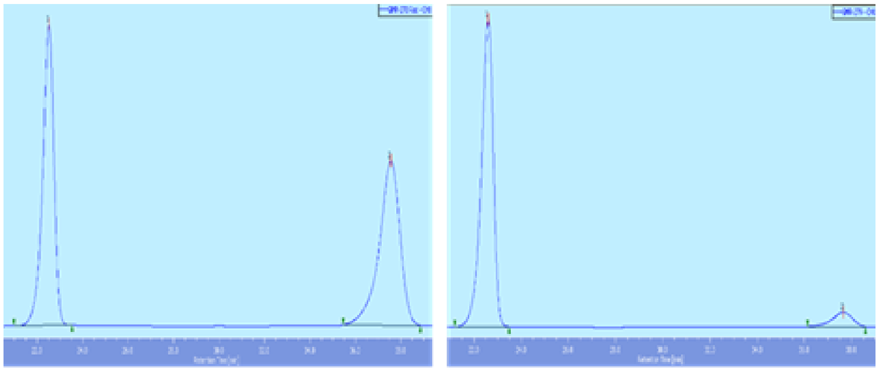

No.	t_R (min)	Area (%)	Height (%)
1	22.525	49.948	64.631
2	37.550	50.052	35.369

No.	t_R (min)	Area (%)	Height (%)
1	22.600	92.832	95.524
2	37.633	7.168	4.476

Figure S3. HPLC spectrum of **5ba**, related to **Scheme 4**.

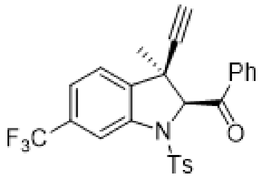

((2*S*,3*R*)-3-ethynyl-3-methyl-1-tosyl-6-(trifluoromethyl)indolin-2-yl)(phenyl)methanone (**5ca**)

HPLC using CHIRALPAK® IB-IC (*n*-hexane/isopropanol = 95.0/5.0, flow rate 1.0 mL/min, λ=254 nm)

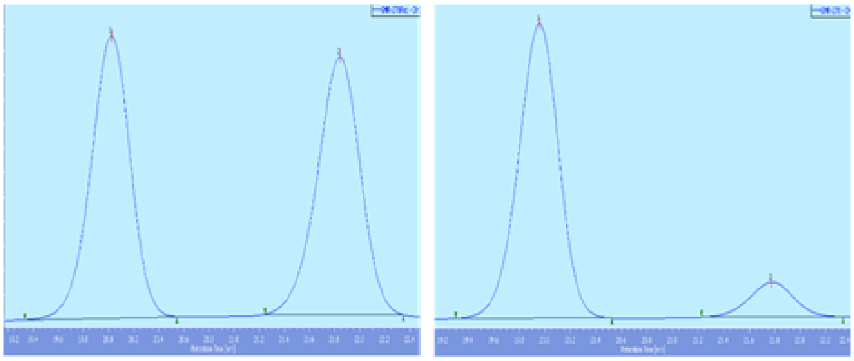

No.	t_R (min)	Area (%)	Height (%)
1	20.025	50.065	52.292
2	21.842	49.935	47.708

No.	t_R (min)	Area (%)	Height (%)
1	19.958	88.468	89.426
2	21.775	11.532	10.574

Figure S4. HPLC spectrum of **5ca**, related to **Scheme 4**.

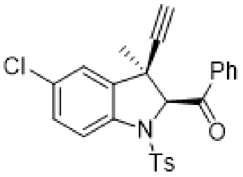

((2*S*,3*R*)-5-chloro-3-ethynyl-3-methyl-1-tosylindolin-2-yl)(phenyl)methanone (**5da**)

HPLC using CHIRALPAK® IG (*n*-hexane/isopropanol = 95.0/5.0, flow rate 1.0 mL/min, λ=254 nm)

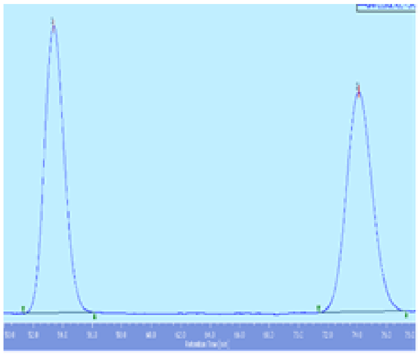

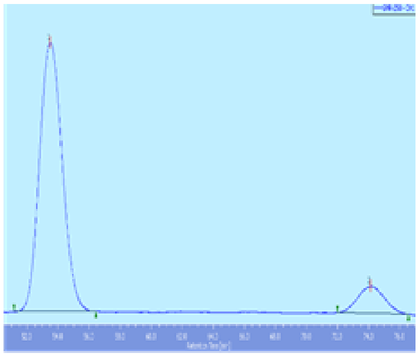

No.	t_R (min)	Area (%)	Height (%)
1	53.358	50.075	56.620
2	74.067	49.925	43.380

No.	t_R (min)	Area (%)	Height (%)
1	53.500	89.378	90.868
2	74.108	10.622	9.132

Figure S5. HPLC spectrum of **5da**, related to **Scheme 4**.

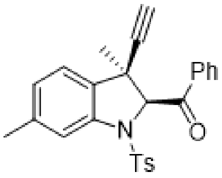

((2*S*,3*R*)-3-ethynyl-3,6-dimethyl-1-tosylindolin-2-yl)(phenyl)methanone (**5ea**)

HPLC using CHIRALPAK® IC (*n*-hexane/isopropanol = 95.0/5.0, flow rate 1.0 mL/min, λ=254 nm)

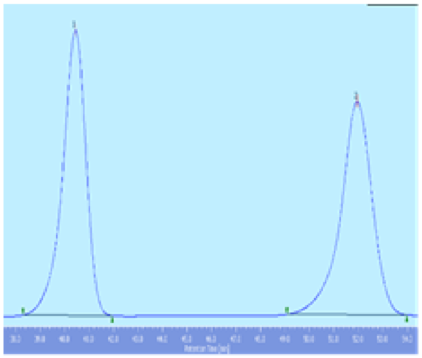

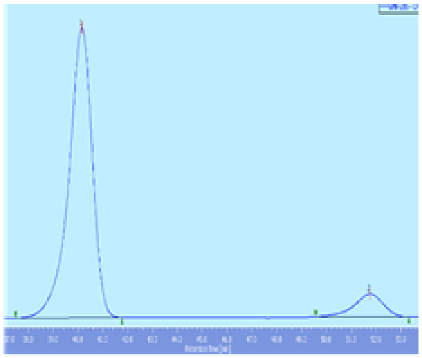

No.	t_R (min)	Area (%)	Height (%)
1	40.442	49.988	57.214
2	51.933	50.012	42.786

No.	t_R (min)	Area (%)	Height (%)
1	40.158	91.141	92.732
2	51.733	8.859	7.268

Figure S6. HPLC spectrum of **5ea**, related to **Scheme 4**.

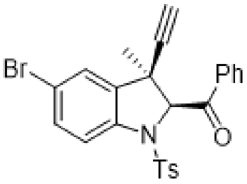

((2*S*,3*R*)-5-bromo-3-ethynyl-3-methyl-1-tosylindolin-2-yl)(phenyl)methanone (**5fa**)

HPLC using CHIRALPAK® IF (*n*-hexane/isopropanol = 95.0/5.0, flow rate 1.0 mL/min, λ=254 nm)

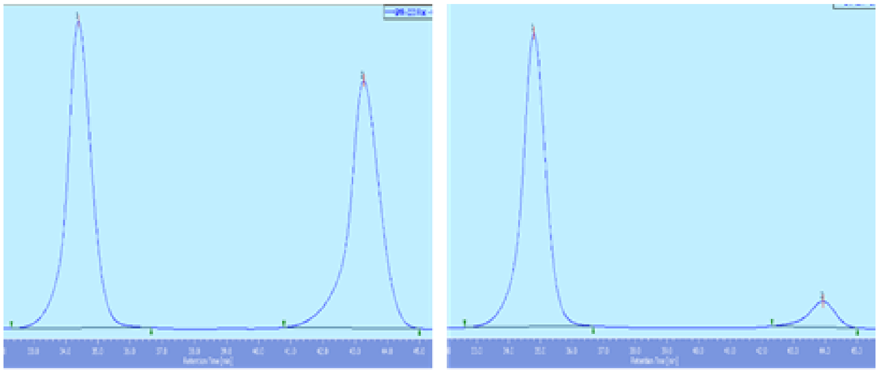

No.	t_R (min)	Area (%)	Height (%)
1	34.392	49.947	55.378
2	43.250	50.053	44.622

No.	t_R (min)	Area (%)	Height (%)
1	34.792	90.988	91.902
2	43.925	9.012	8.098

Figure S7. HPLC spectrum of **5fa**, related to **Scheme 4**.

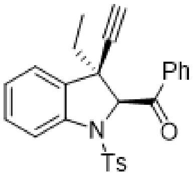

((2*S*,3*R*)-3-ethyl-3-ethynyl-1-tosylindolin-2-yl)(phenyl)methanone (**5ga**)

HPLC using CHIRALPAK® IC (*n*-hexane/isopropanol = 90.0/10.0, flow rate 1.0 mL/min, λ=254 nm)

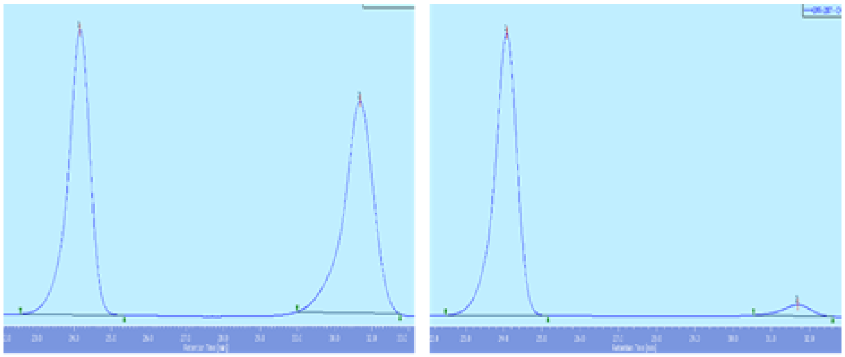

No.	t_R (min)	Area (%)	Height (%)
1	24.150	49.844	57.328
2	31.675	50.156	42.672

No.	t_R (min)	Area (%)	Height (%)
1	24.075	95.326	96.167
2	31.683	4.674	3.833

Figure S8. HPLC spectrum of **5ga**, related to **Scheme 4**.

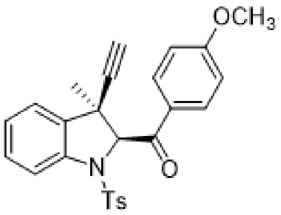

((2*S*,3*R*)-3-ethynyl-3-methyl-1-tosylindolin-2-yl)(4-methoxyphenyl)methanone (**5ab**)

HPLC using CHIRALPAK® IF (*n*-hexane/isopropanol = 90.0/10.0, flow rate 1.0 mL/min, λ=254 nm)

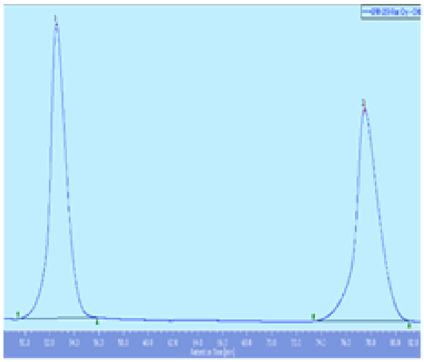

No.	t_R (min)	Area (%)	Height (%)
1	52.533	49.708	58.146
2	77.475	50.292	41.854

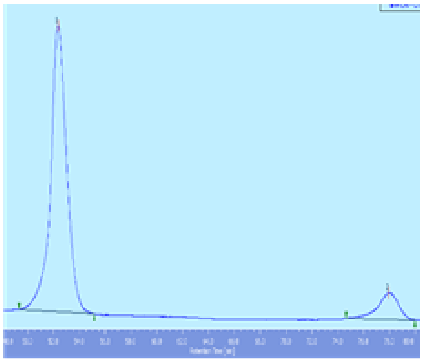

No.	t_R (min)	Area (%)	Height (%)
1	52.333	89.240	91.490
2	77.925	10.760	8.510

Figure S9. HPLC spectrum of **5ab**, related to **Scheme 4**.

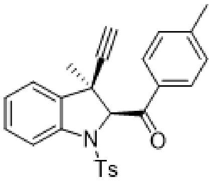

((2*S*,3*R*)-3-ethynyl-3-methyl-1-tosylindolin-2-yl)(*p*-tolyl)methanone (**5ac**)

HPLC using CHIRALPAK® IG (*n*-hexane/isopropanol = 90.0/10.0, flow rate 1.5 mL/min, λ=254 nm)

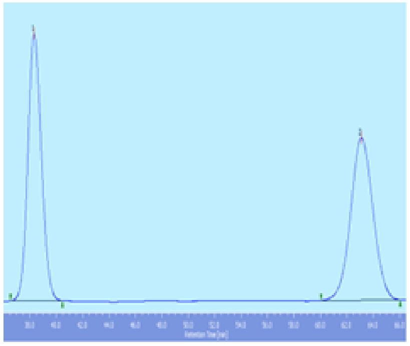

No.	t_R (min)	Area (%)	Height (%)
1	38.325	49.810	62.165
2	63.075	50.190	37.835

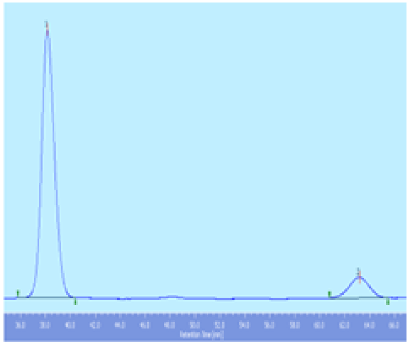

No.	t_R (min)	Area (%)	Height (%)
1	38.158	89.323	92.920
2	63.133	10.677	7.080

Figure S10. HPLC spectrum of **5ac**, related to **Scheme 4**.

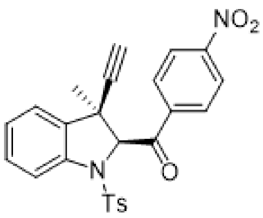

((2*S*,3*R*)-3-ethynyl-3-methyl-1-tosylindolin-2-yl)(4-nitrophenyl)methanone (**8ad**)

HPLC using CHIRALPAK® IB-IC (*n*-hexane/isopropanol = 90.0/10.0, flow rate 1.5 mL/min, λ=254 nm)

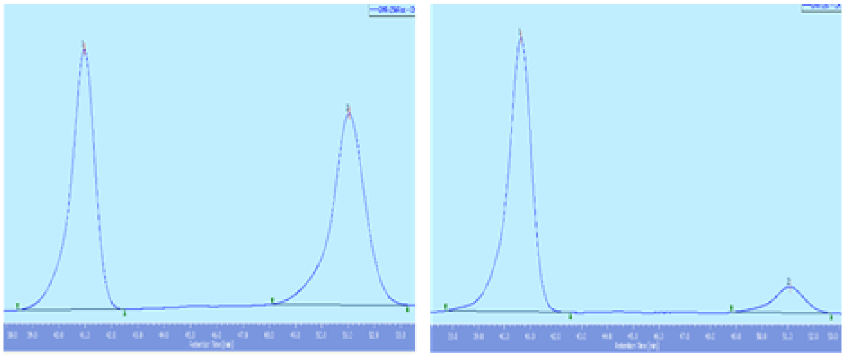

No.	t_R (min)	Area (%)	Height (%)
1	40.958	50.125	57.675
2	51.017	49.875	42.325

No.	t_R (min)	Area (%)	Height (%)
1	40.633	89.157	91.378
2	51.075	10.843	8.622

Figure S11. HPLC spectrum of **5ad**, related to **Scheme 4**.

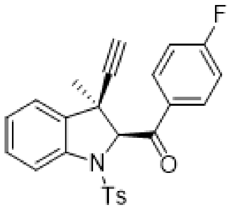

((2*S*,3*R*)-3-ethynyl-3-methyl-1-tosylindolin-2-yl)(4-fluorophenyl)methanone (**5ae**)

HPLC using CHIRALPAK® IC (*n*-hexane/isopropanol = 95.0/5.0, flow rate 1.0 mL/min, λ=254 nm)

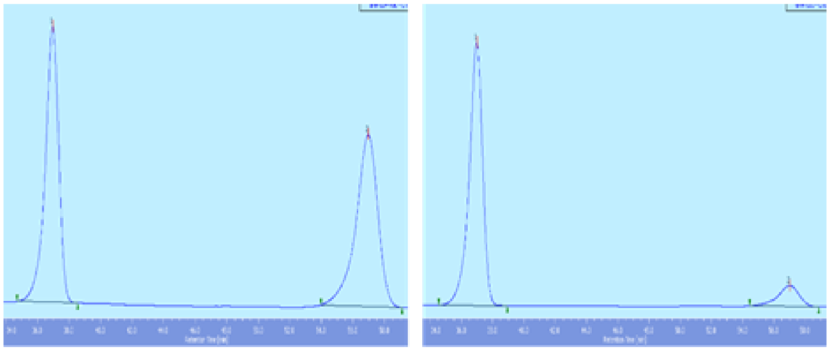

No.	t_R (min)	Area (%)	Height (%)
1	36.975	49.913	61.643
2	56.933	50.087	38.357

No.	t_R (min)	Area (%)	Height (%)
1	36.967	88.950	92.618
2	57.000	11.050	7.382

Figure S12. HPLC spectrum of **5ae**, related to **Scheme 4**.

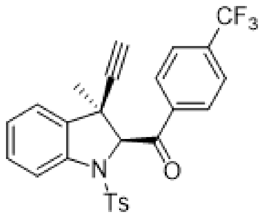

((2*S*,3*R*)-3-ethynyl-3-methyl-1-tosylindolin-2-yl)(4-(trifluoromethyl)phenyl)methanone (**5af**)

HPLC using CHIRALPAK® IG (*n*-hexane/isopropanol = 90.0/10.0, flow rate 1.5 mL/min, λ=254 nm)

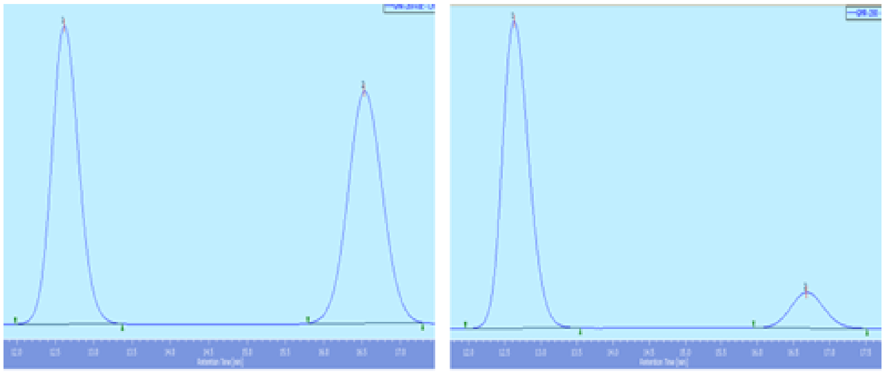

No.	t_R (min)	Area (%)	Height (%)
1	12.617	50.053	56265
2	16.525	49.947	43.735

No.	t_R (min)	Area (%)	Height (%)
1	12.625	87.115	89.563
2	16.675	12.885	10.437

Figure S13. HPLC spectrum of **5af**, related to **Scheme 4**.

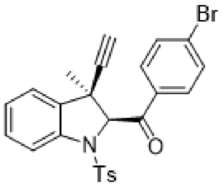

(4-bromophenyl)((2*S*,3*R*)-3-ethynyl-3-methyl-1-tosylindolin-2-yl)methanone (**5ag**)

HPLC using CHIRALPAK® IC (*n*-hexane/isopropanol = 95.0/5.0, flow rate 1.0 mL/min, λ=254 nm)

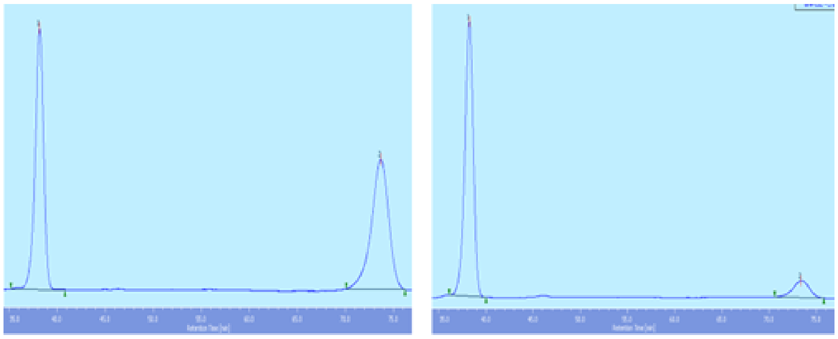

No.	t_R (min)	Area (%)	Height (%)
1	38.183	49.917	66.739
2	73.658	50.083	33.261

No.	t_R (min)	Area (%)	Height (%)
1	38.225	89.336	94.157
2	73.358	10.664	5.843

Figure S14. HPLC spectrum of **5ag**, related to **Scheme 4**.

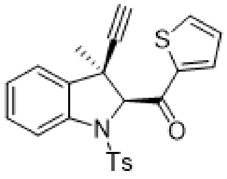

((2*S*,3*R*)-3-ethynyl-3-methyl-1-tosylindolin-2-yl)(thiophen-2-yl)methanone (**5ah**)

HPLC using CHIRALPAK® IA (*n*-hexane/isopropanol = 95.0/5.0, flow rate 1.0 mL/min, λ=254 nm)

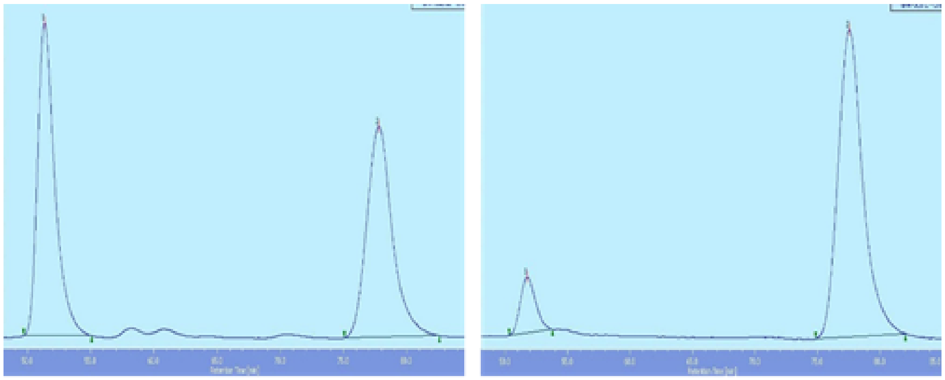

No.	t_R (min)	Area (%)	Height (%)
1	51.350	50.225	59.719
2	77.758	49.775	49.775

No.	t_R (min)	Area (%)	Height (%)
1	51.758	10.035	15.394
2	77.533	89.965	84.606

Figure S15. HPLC spectrum of **5ah**, related to **Scheme 4**.

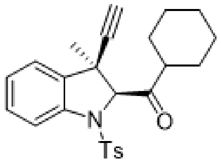

Cyclohexyl((2*S*,3*R*)-3-ethynyl-3-methyl-1-tosylindolin-2-yl)methanone (**5ai**)

HPLC using CHIRALPAK® IG (*n*-hexane/isopropanol = 95.0/5.0, flow rate 1.0 mL/min, λ=254 nm)

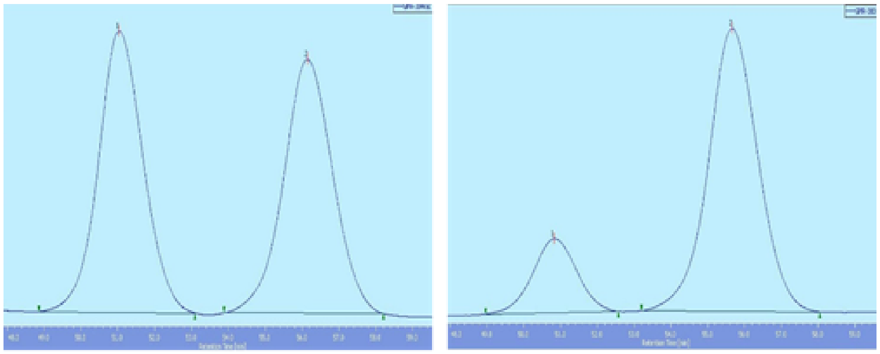

No.	t_R (min)	Area (%)	Height (%)
1	51.003	50.180	52.557
2	56.133	49.820	47.443

No.	t_R (min)	Area (%)	Height (%)
1	50.825	18.798	20.743
2	55.658	81.202	79.257

Figure S16. HPLC spectrum of **5ai**, related to **Scheme 4**.

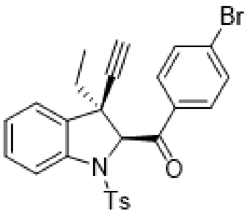

(4-bromophenyl)((2*S*,3*R*)-3-ethyl-3-ethynyl-1-tosylindolin-2-yl)methanone (**5gg**)

HPLC using CHIRALPAK® IC (*n*-hexane/isopropanol = 95.0/5.0, flow rate 1.0 mL/min, λ=254 nm)

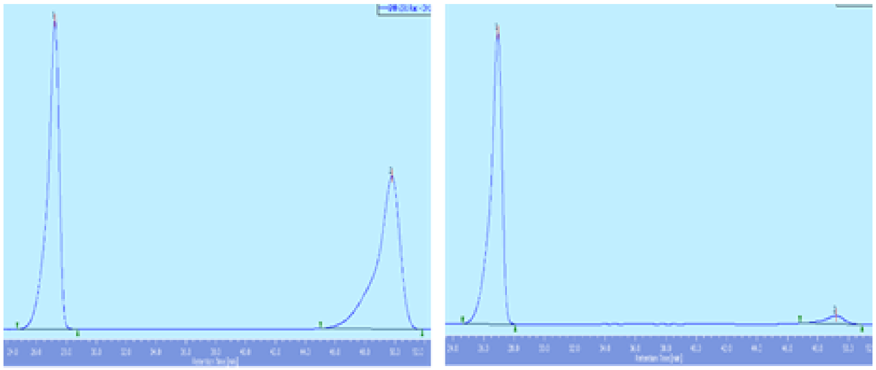

No.	t_R (min)	Area (%)	Height (%)
1	27.217	49.991	66.929
2	49.767	50.009	33.071

No.	t_R (min)	Area (%)	Height (%)
1	26.925	95.547	97.299
2	49.192	4.453	2.701

Figure S17. HPLC spectrum of **5gg**, related to **Scheme 4**.

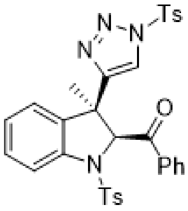

((2*S*,3*R*)-3-methyl-1-tosyl-3-(1-tosyl-1*H*-1,2,3-triazol-4-yl)indolin-2-yl)(phenyl)methanone (**7**)

HPLC using CHIRALPAK® IC (*n*-hexane/isopropanol = 85.0/15.0, flow rate 1.0 mL/min, λ=254 nm)

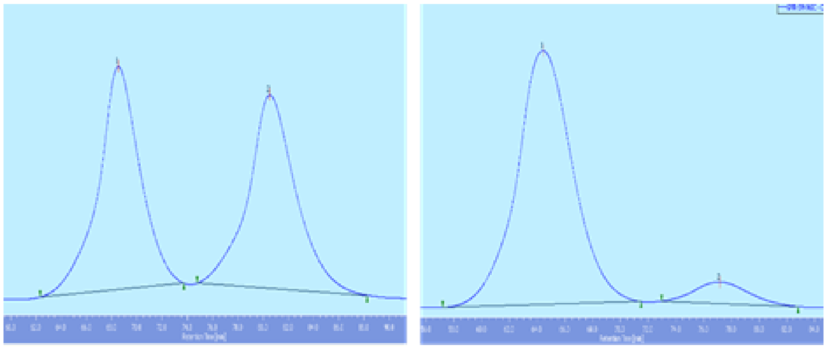

No.	t_R (min)	Area (%)	Height (%)
1	68.508	50.228	53.559
2	80.500	49.772	46.441

No.	t_R (min)	Area (%)	Height (%)
1	64.408	92.481	92.185
2	77.175	7.519	7.815

Figure S18. HPLC spectrum of **7**, related to **Scheme 5**.

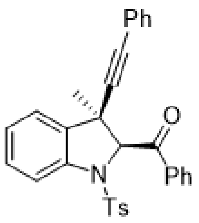

((2S,3R)-3-methyl-3-(phenylethynyl)-1-tosylindolin-2-yl)(phenyl)methanone (**8**)

HPLC using CHIRALPAK® IB IB (*n*-hexane/isopropanol = 98.0/2.0, flow rate 1.0 mL/min, λ=254 nm)

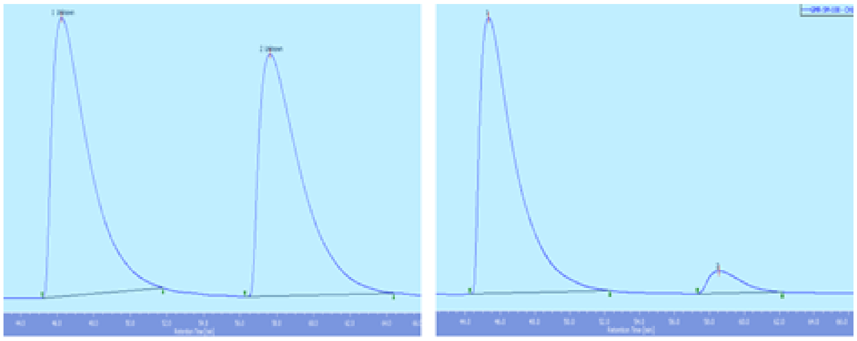

No.	tR (min)	Area (%)	Height (%)
1	46.242	50.321	53.508
2	57.608	49.679	46.492

No.	tR (min)	Area (%)	Height (%)
1	45.333	93.058	92.443
2	58.517	6.942	7.557

Figure S19. HPLC spectrum of **8**, related to **Scheme 5**.

Supplemental Figures for NMR spectrums:

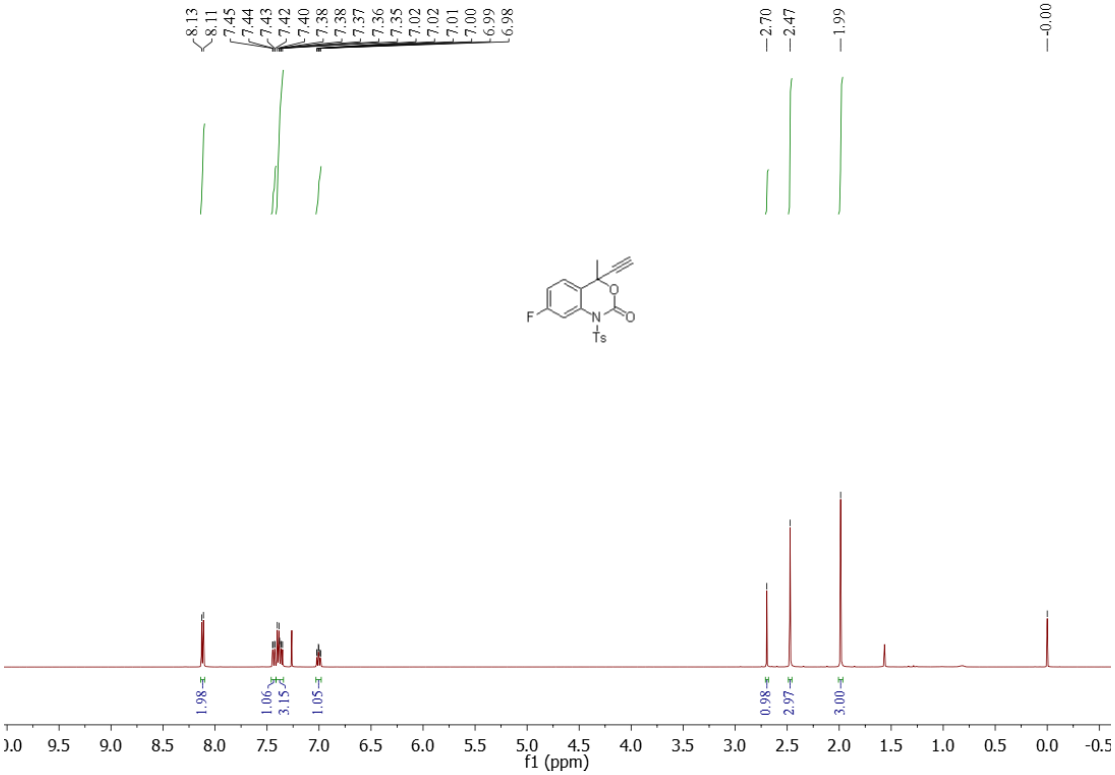

Figure S20. ^{1}H NMR spectrum of **3b**, related to **Scheme 4**.

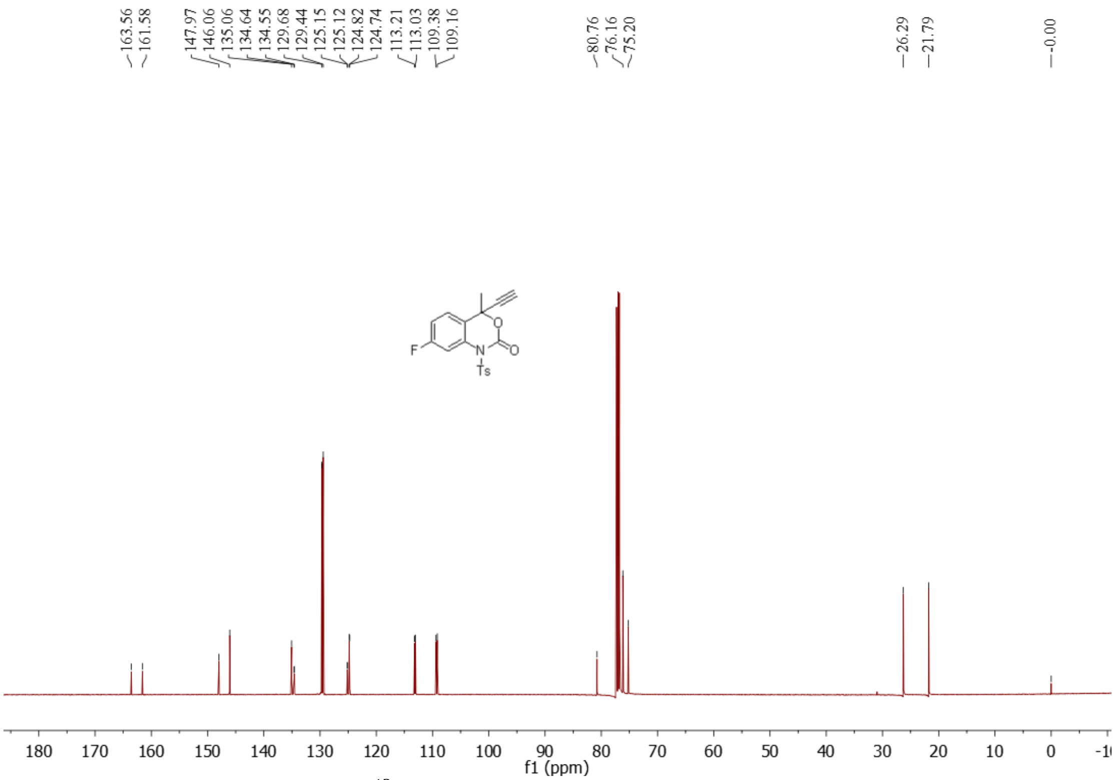

Figure S21. ^{13}C NMR spectrum of **3b**, related to **Scheme 4**.

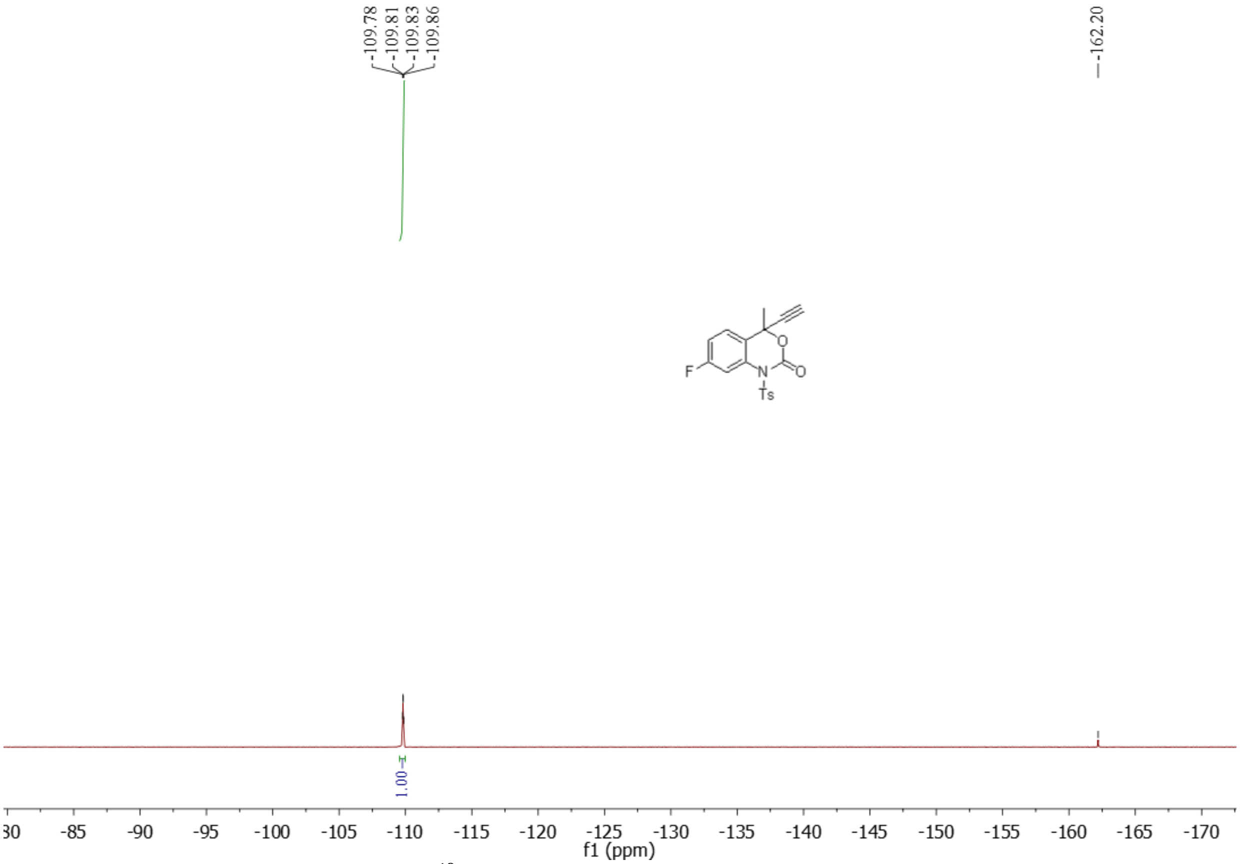

Figure S22. ^{19}F NMR spectrum of **3b**, related to **Scheme 4**.

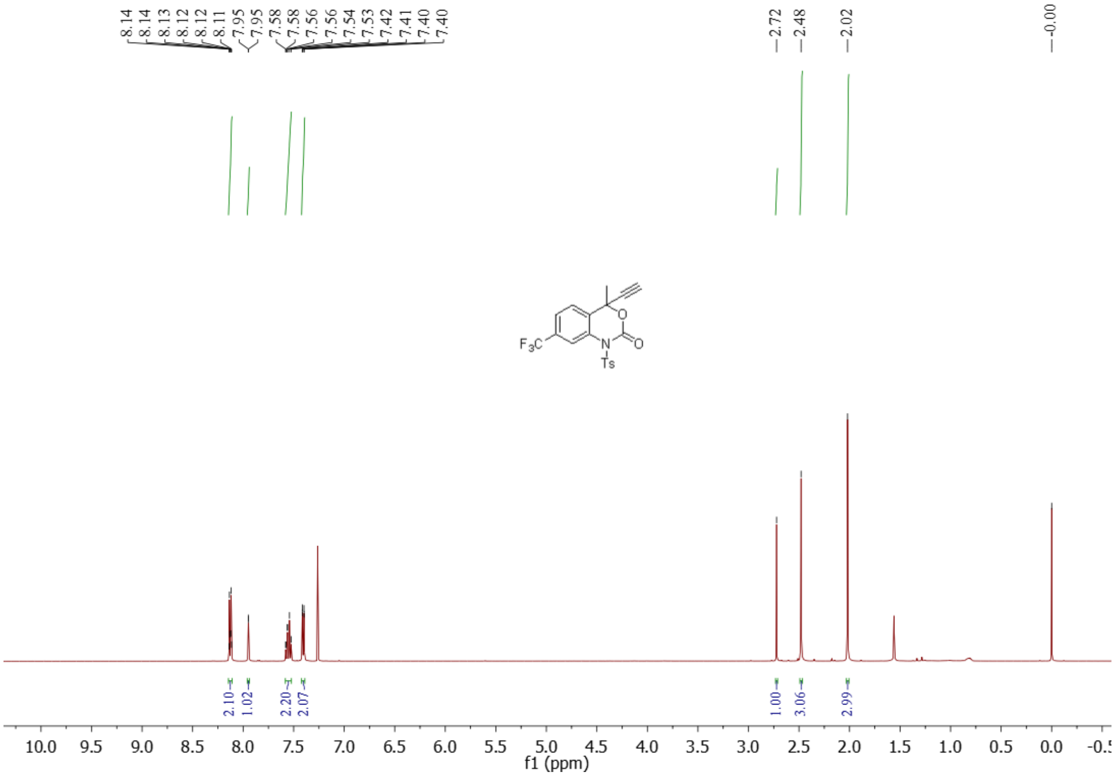

Figure S23. ^{1}H NMR spectrum of **3c**, related to **Scheme 4**.

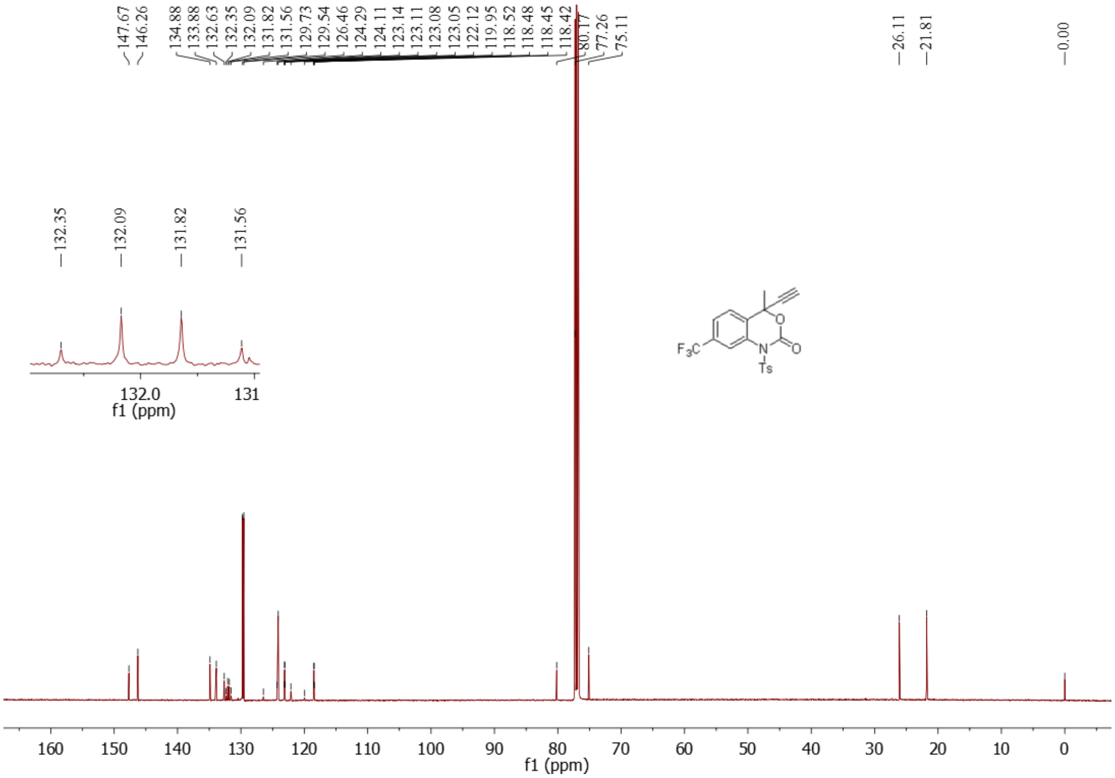

Figure S24. ^{13}C NMR spectrum of **3c**, related to **Scheme 4**.

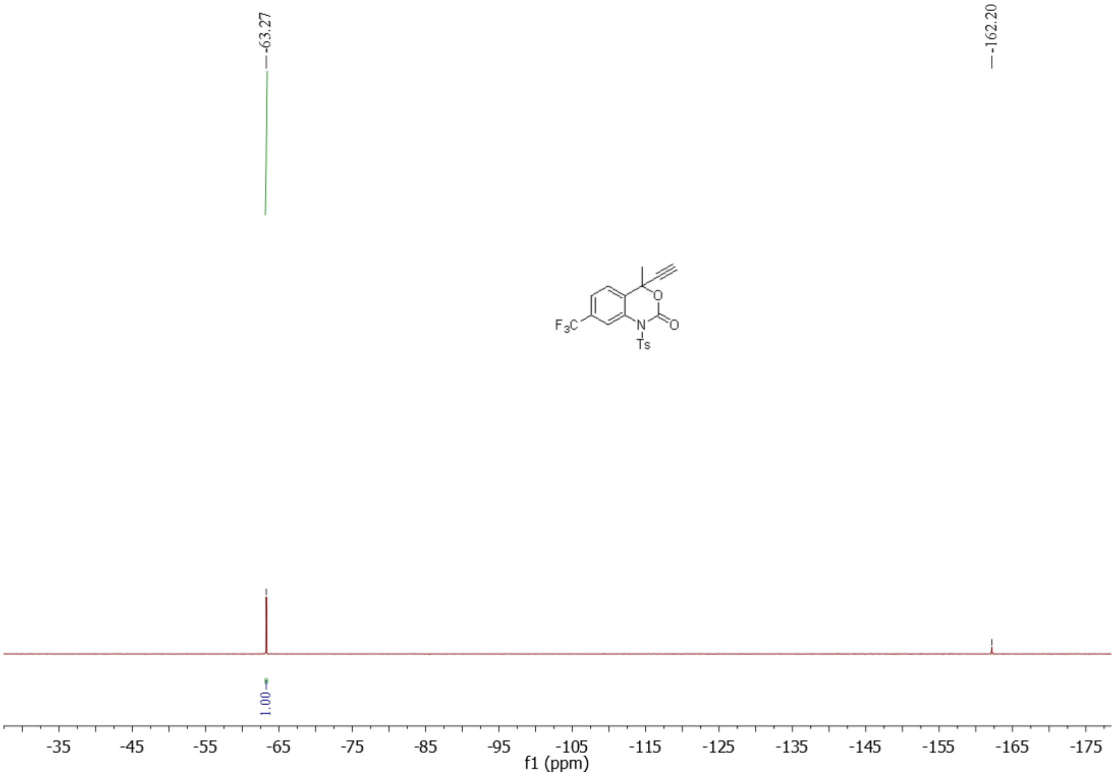

Figure S25. ^{19}F NMR spectrum of **3c**, related to **Scheme 4**.

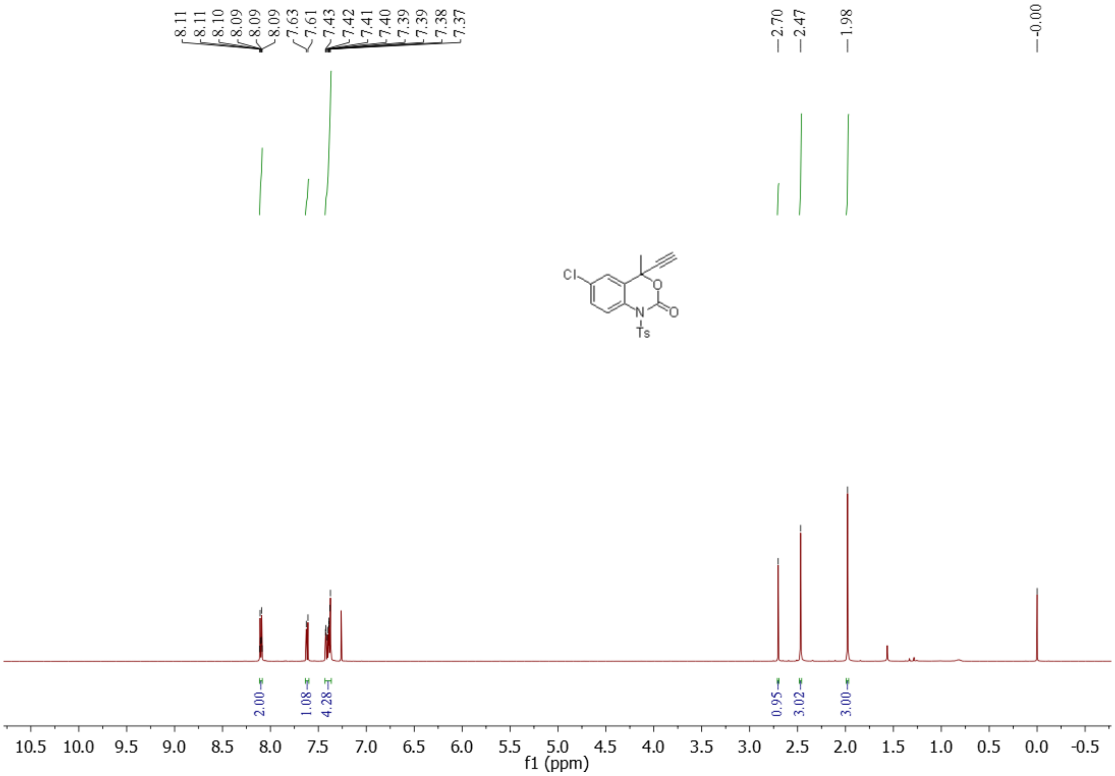

Figure S26. ^{1}H NMR spectrum of **3d**, related to **Scheme 4**.

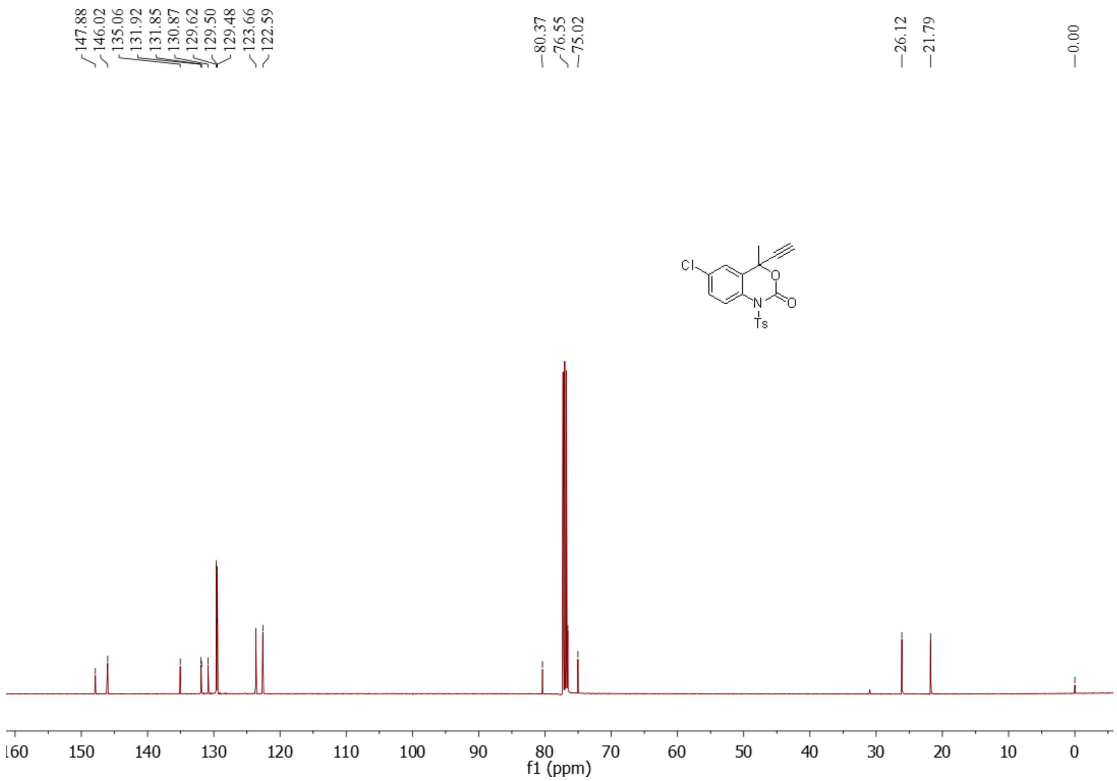

Figure S27. ^{13}C NMR spectrum of **3d**, related to **Scheme 4**.

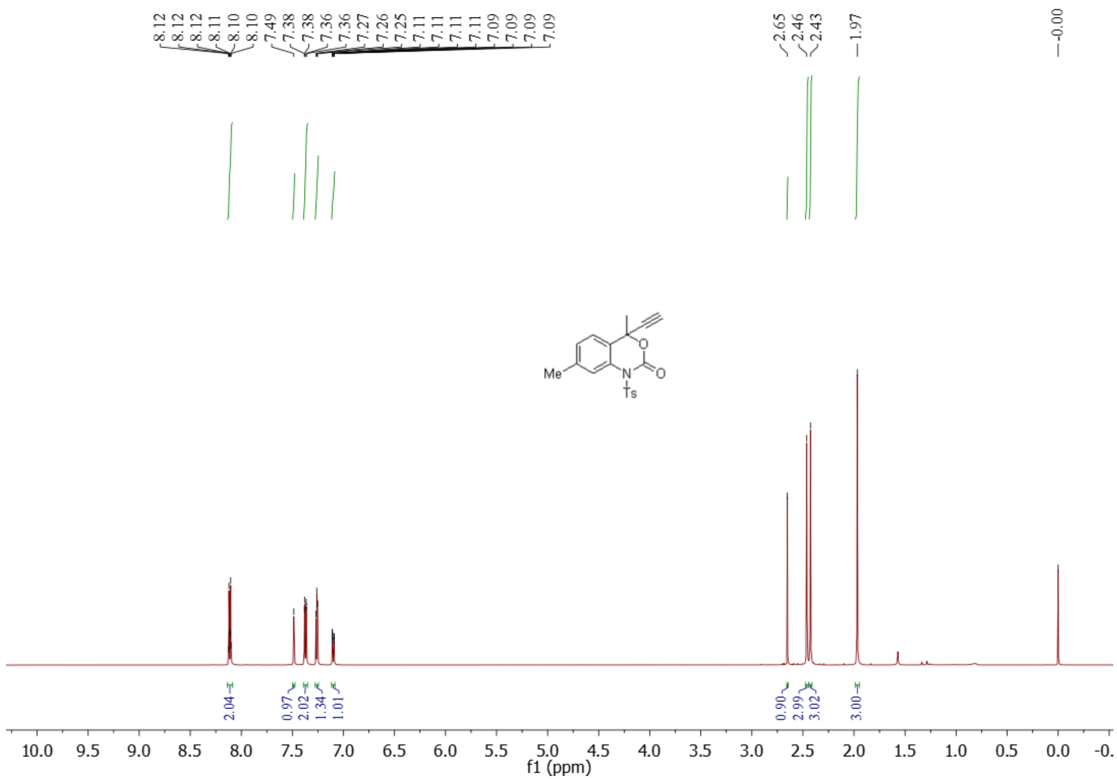

Figure S28. ^{1}H NMR spectrum of **3e**, related to **Scheme 4**.

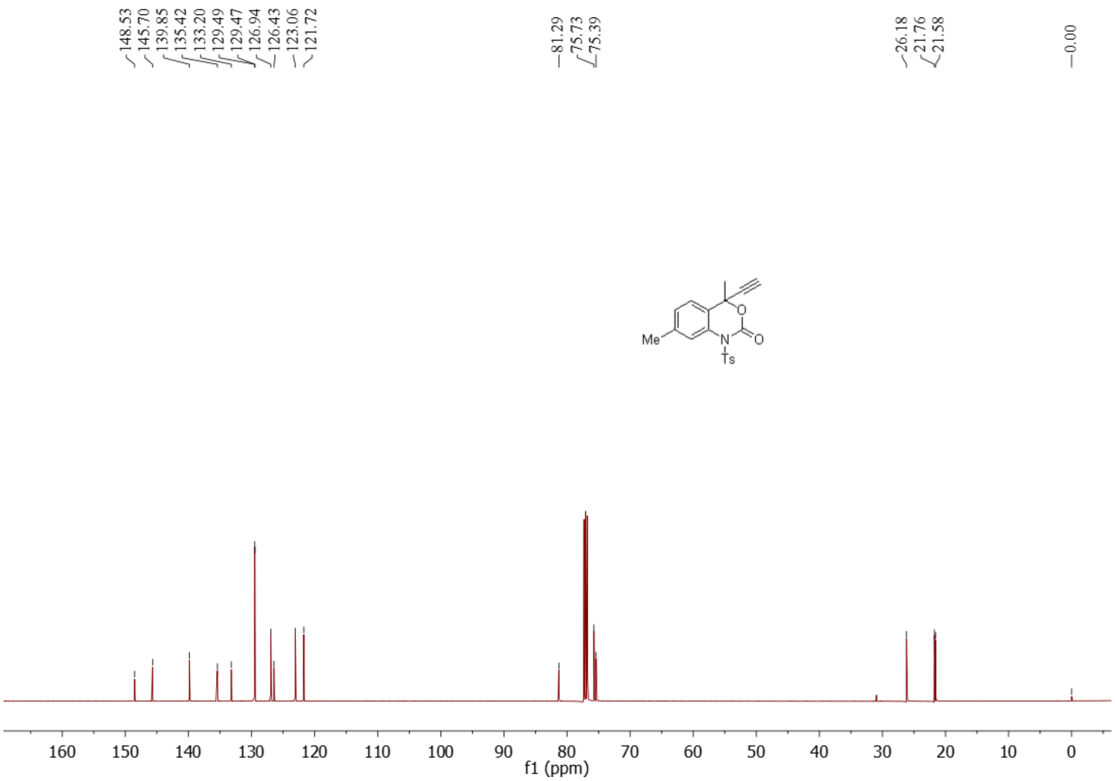

Figure S29. ^{13}C NMR spectrum of **3e**, related to **Scheme 4**.

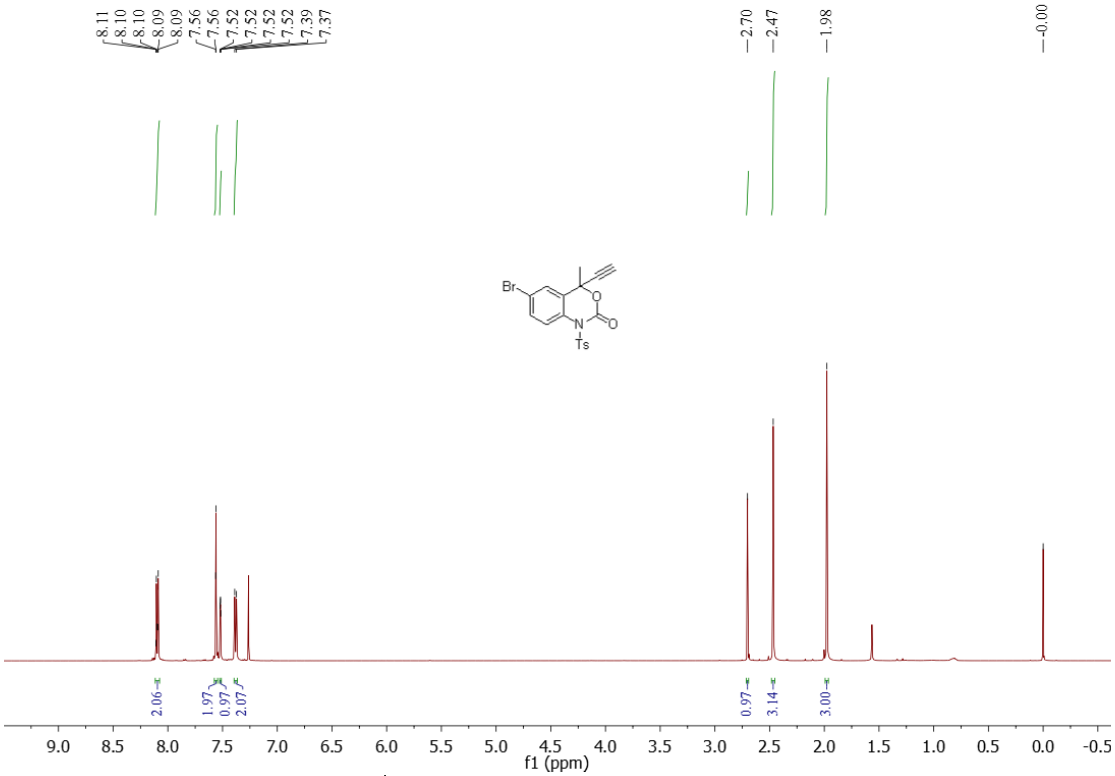

Figure S30. ^{1}H NMR spectrum of **3f**, related to **Scheme 4**.

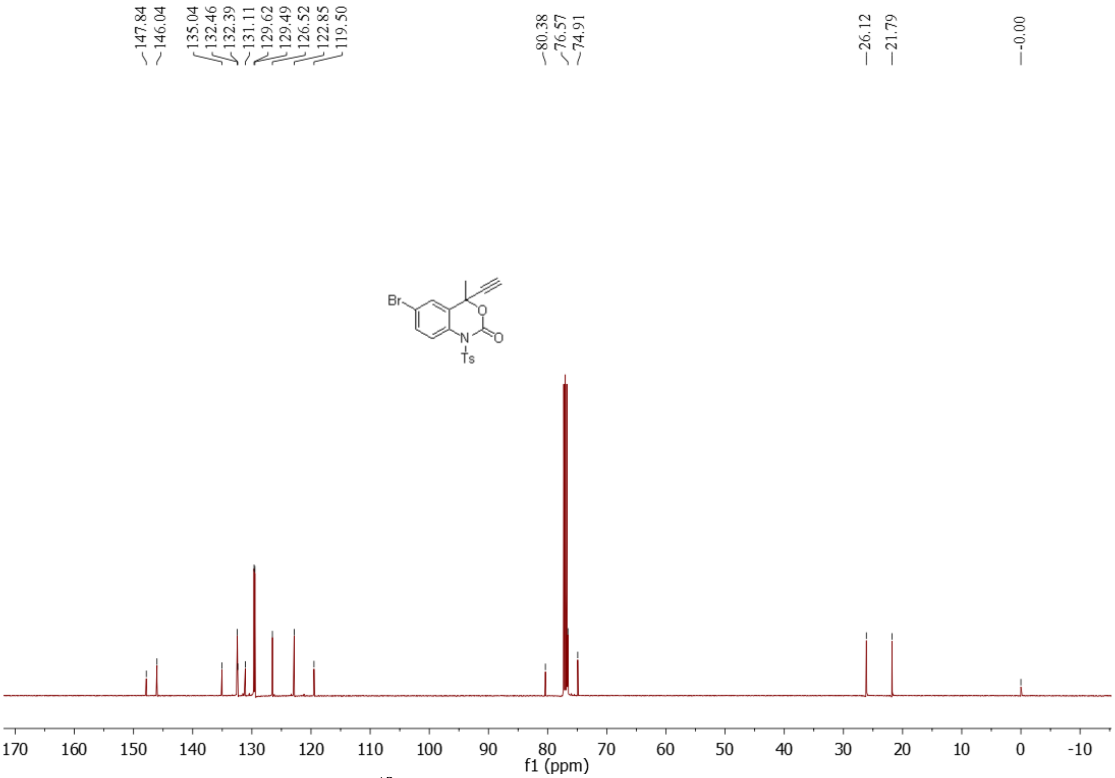

Figure S31. ^{13}C NMR spectrum of **3f**, related to **Scheme 4**.

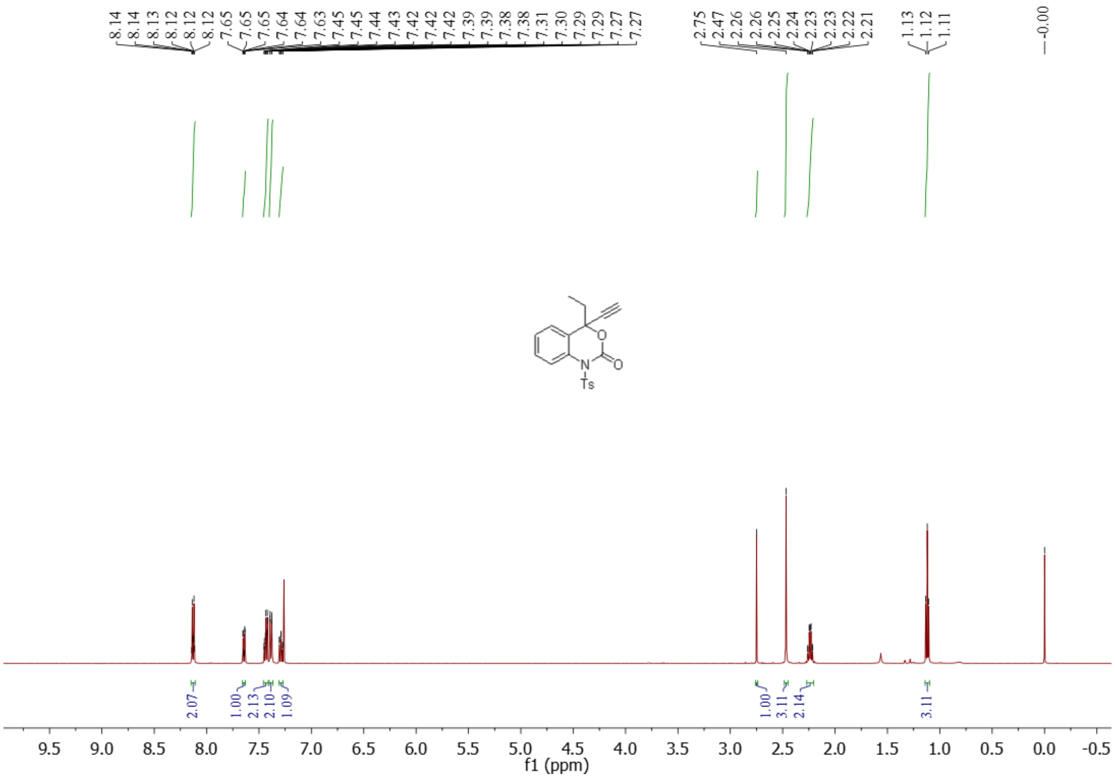

Figure S32. ^{1}H NMR spectrum of **3g**, related to **Scheme 4**.

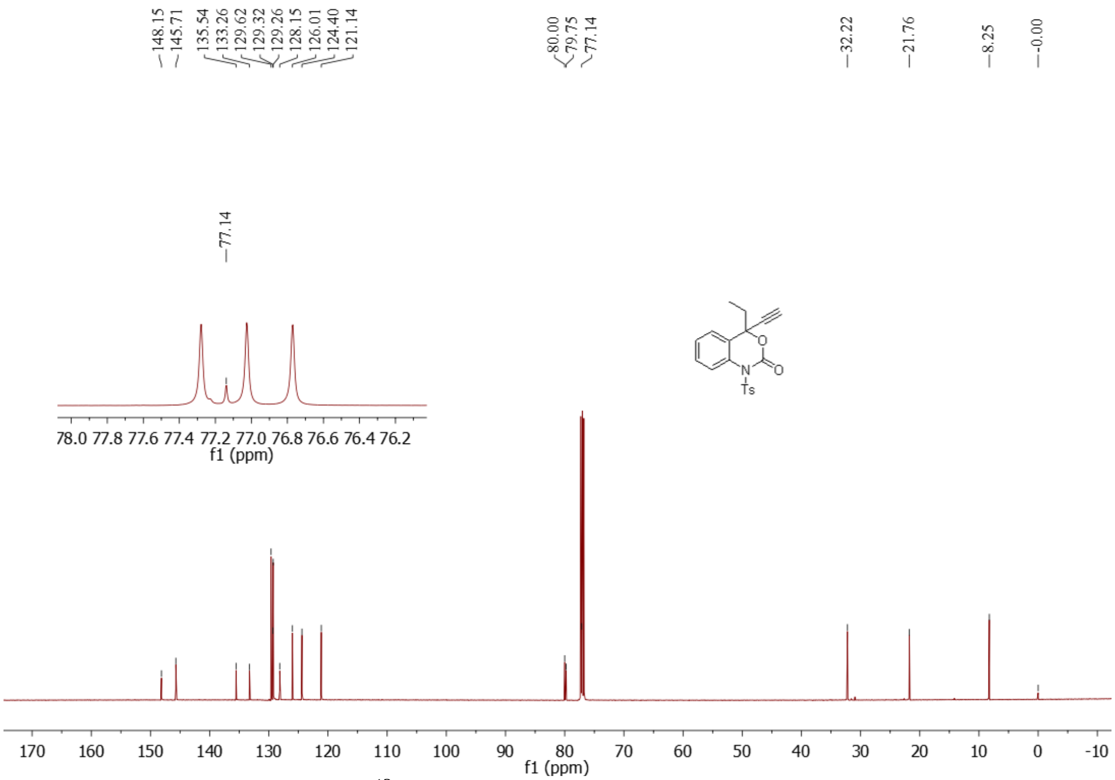

Figure S33. ^{13}C NMR spectrum of **3g**, related to **Scheme 4**.

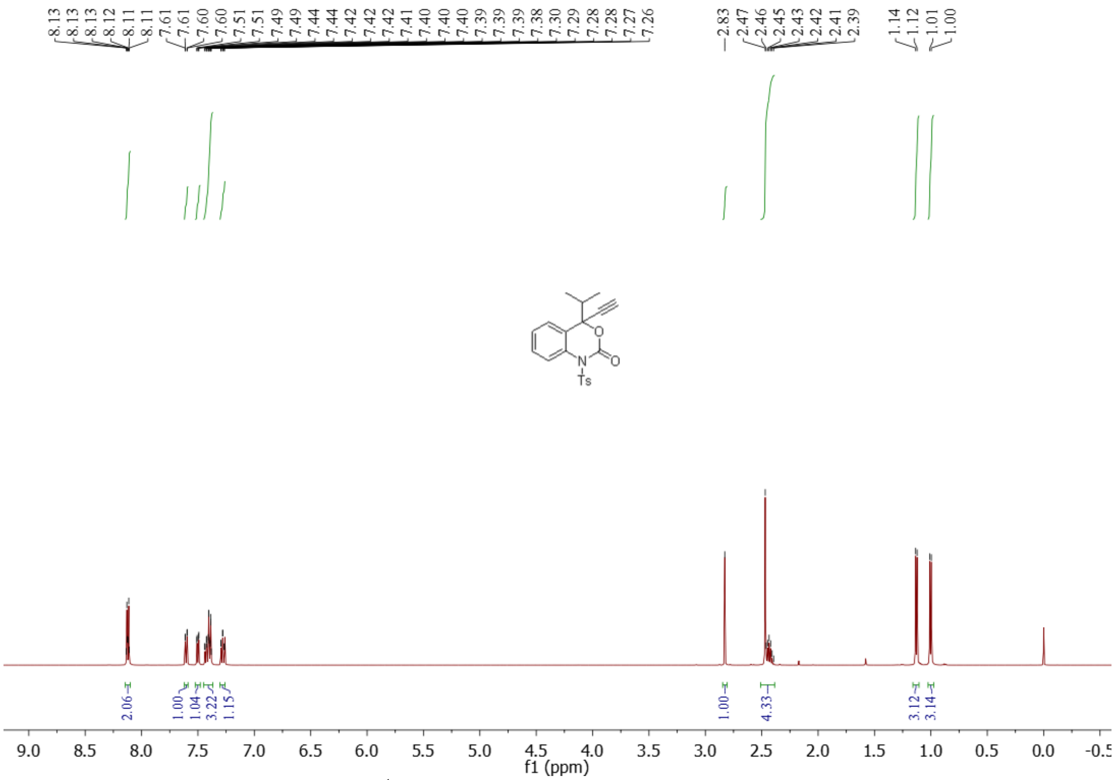

Figure S34. ^{1}H NMR spectrum of **3h**, related to **Figure 2**.

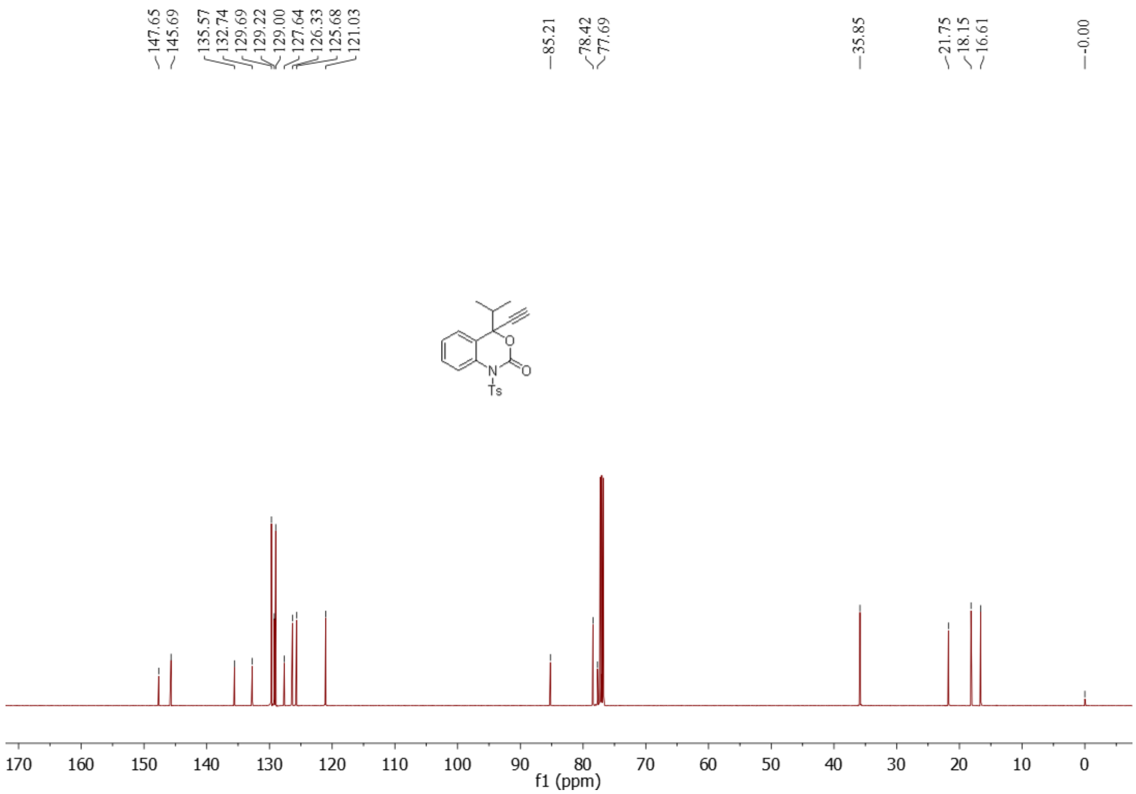

Figure S35. ^{13}C NMR spectrum of **3h**, related to **Figure 2**.

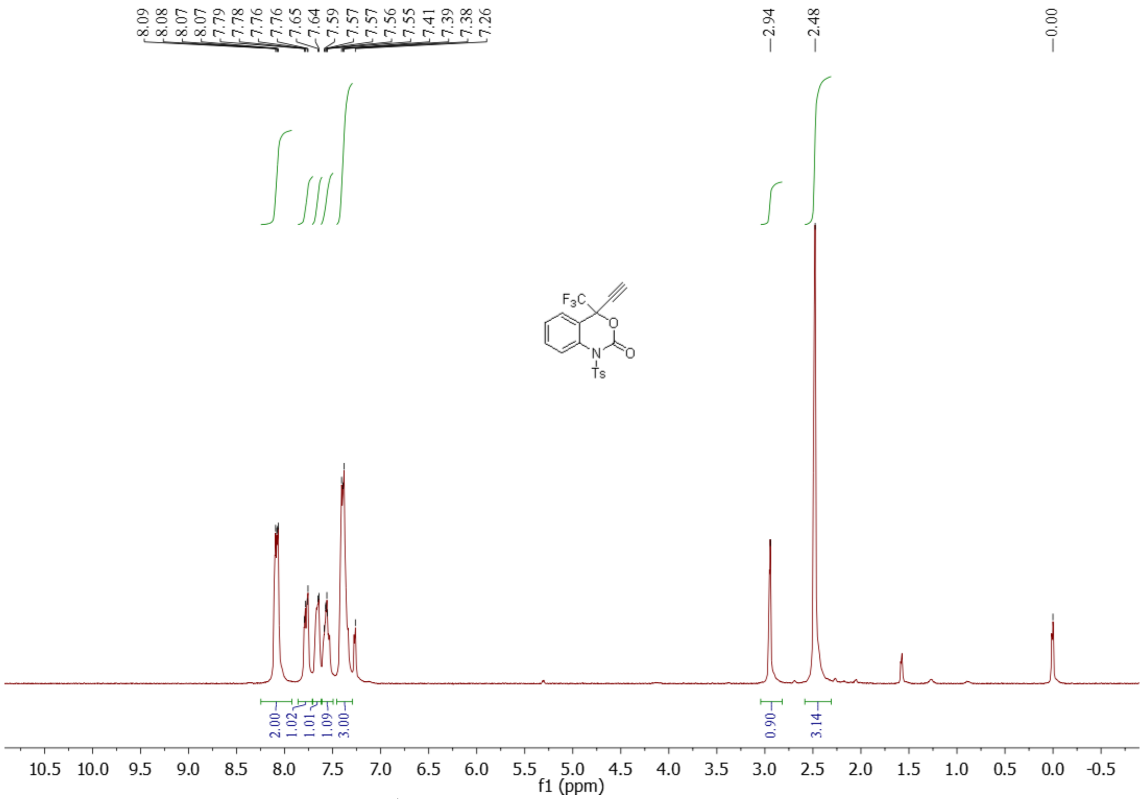

Figure S36. ^{1}H NMR spectrum of **4a**, related to **Scheme 6**.

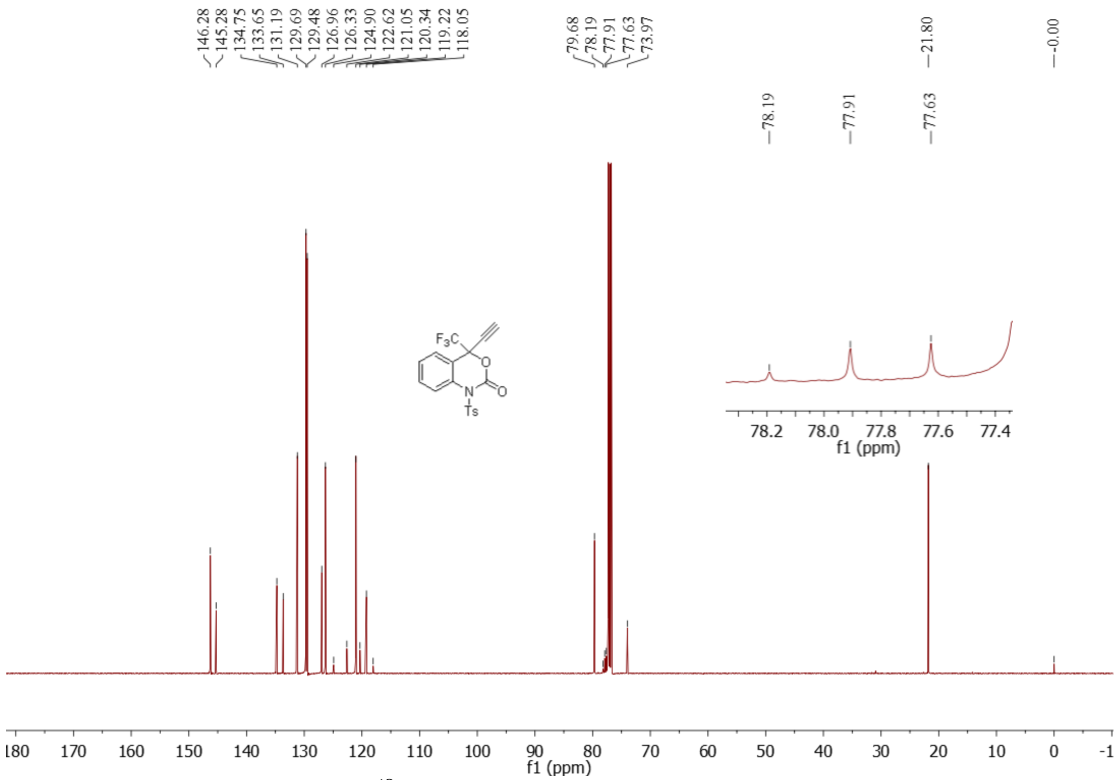

Figure S37. ^{13}C NMR spectrum of **4a**, related to **Scheme 6**.

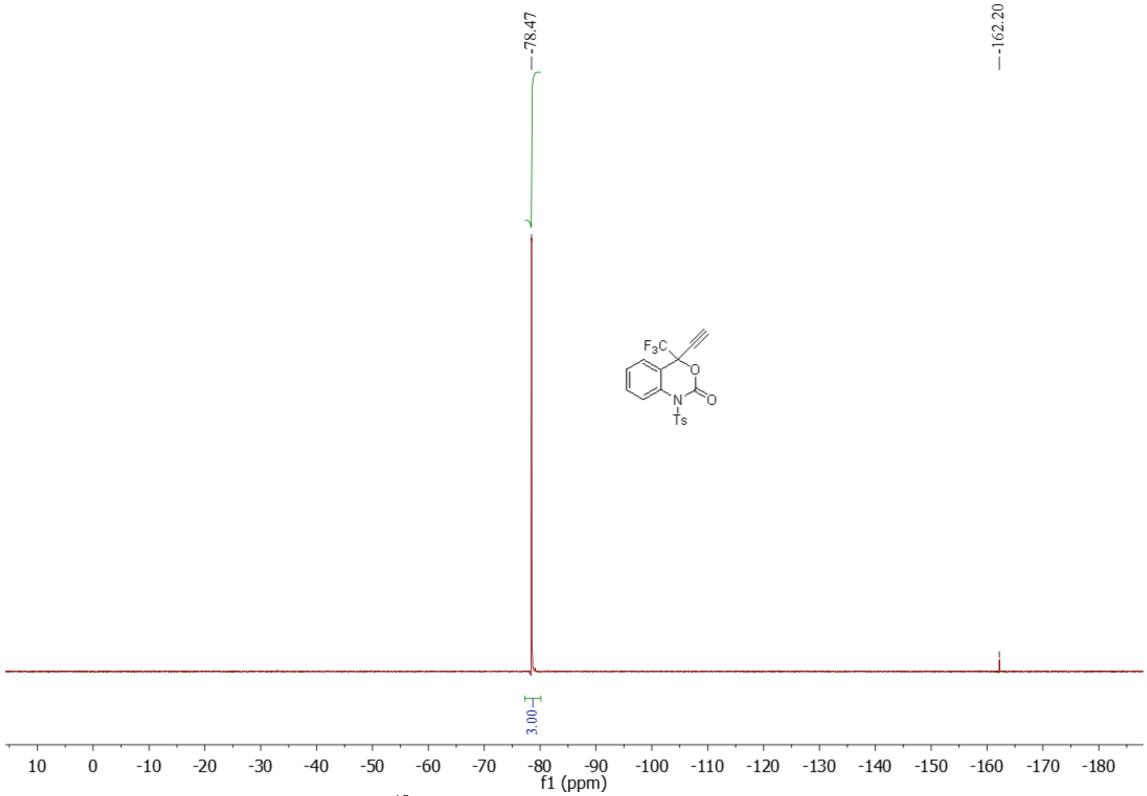

Figure S38. ^{19}F NMR spectrum of **4a**, related to **Scheme 6**.

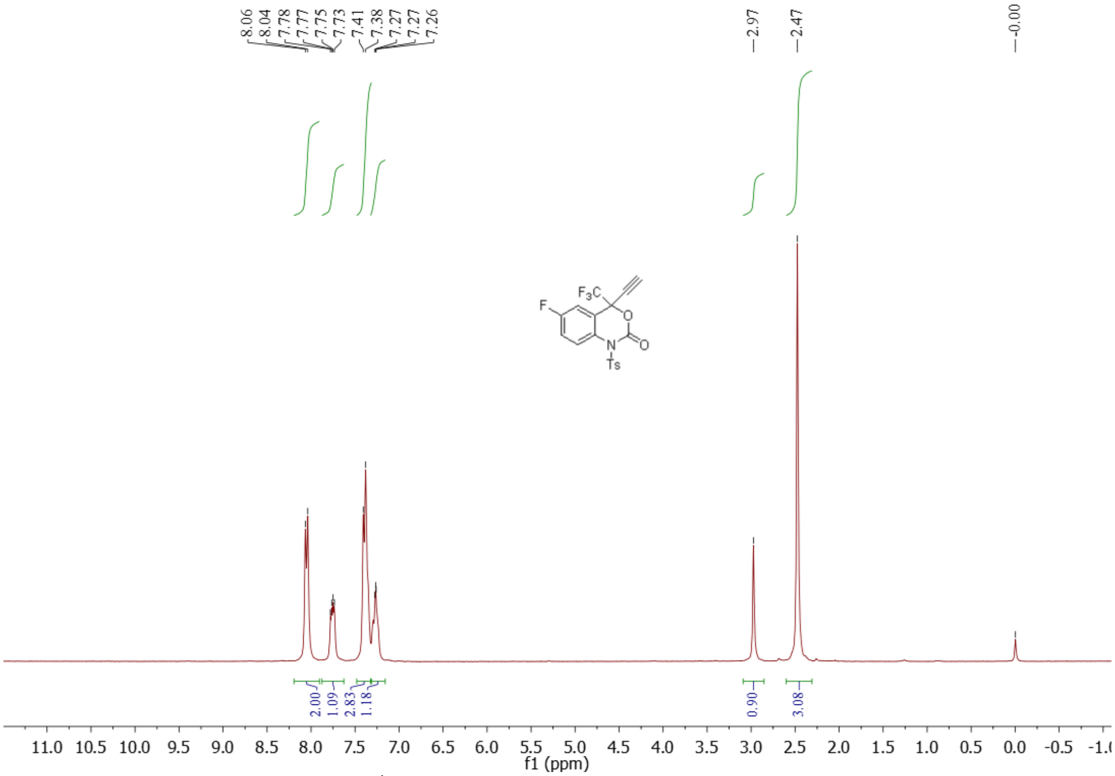

Figure S39. ^{1}H NMR spectrum of **4b**, related to **Scheme 6**.

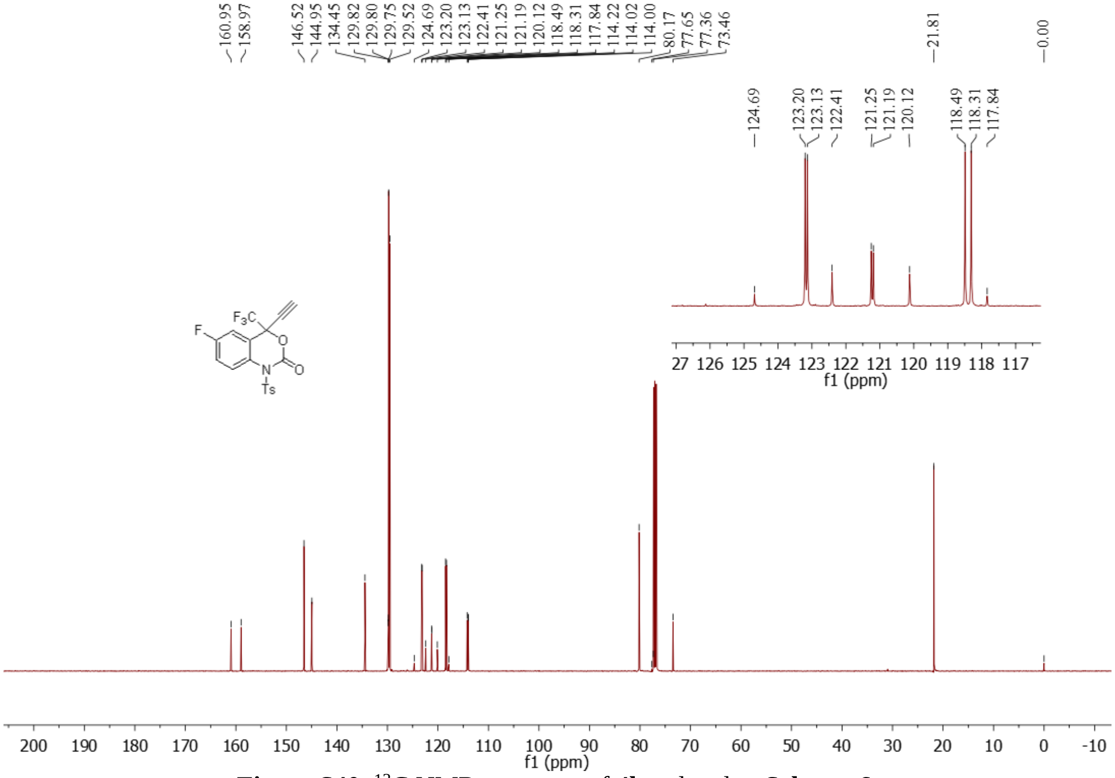

Figure S40. ^{13}C NMR spectrum of **4b**, related to **Scheme 6**.

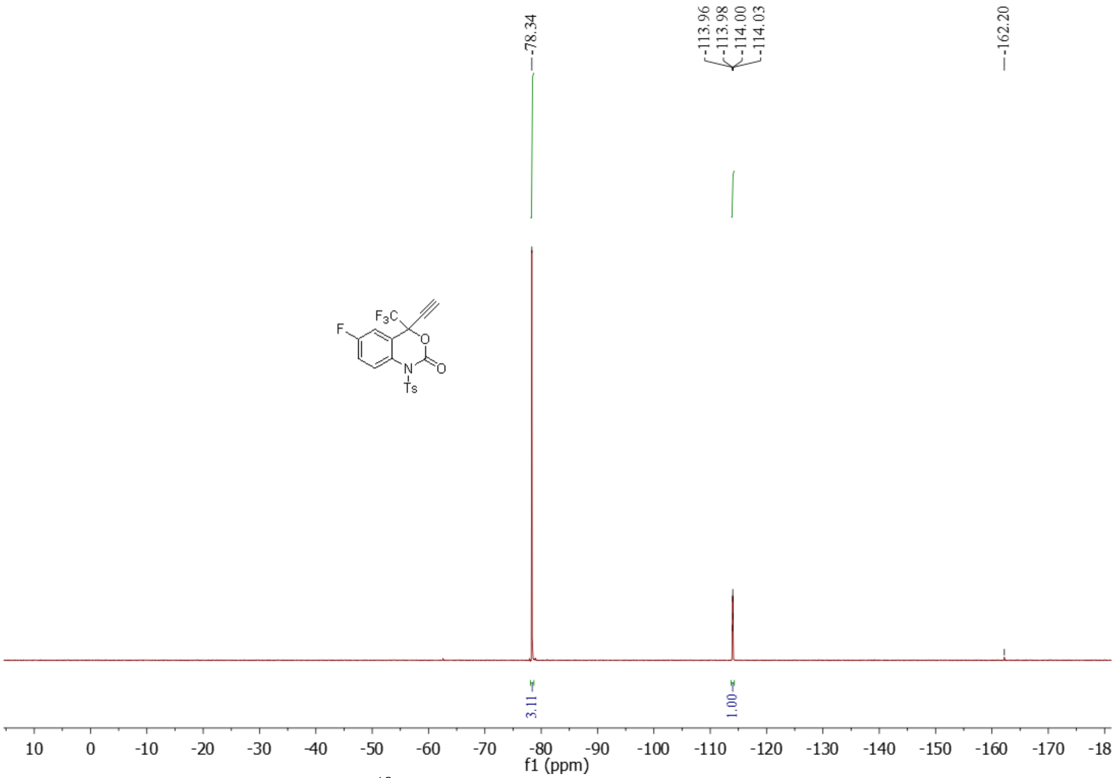

Figure S41. ^{19}F NMR spectrum of **4b**, related to **Scheme 6**.

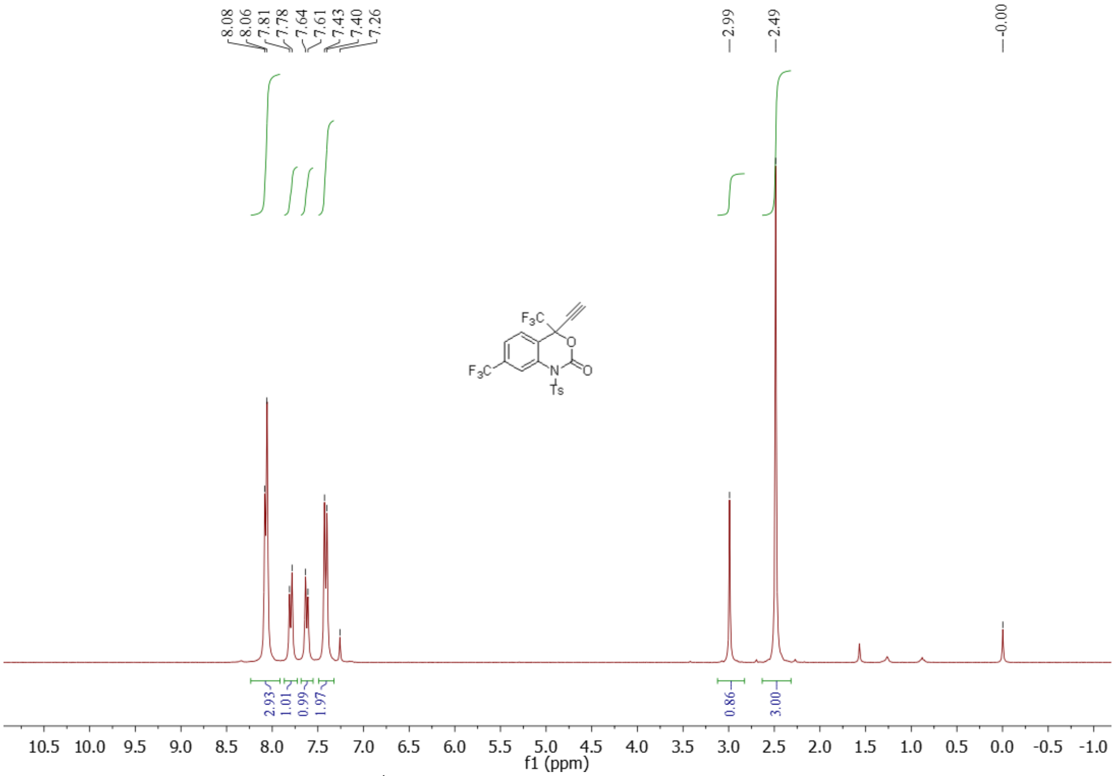

Figure S42. ^{1}H NMR spectrum of **4c**, related to **Scheme 6**.

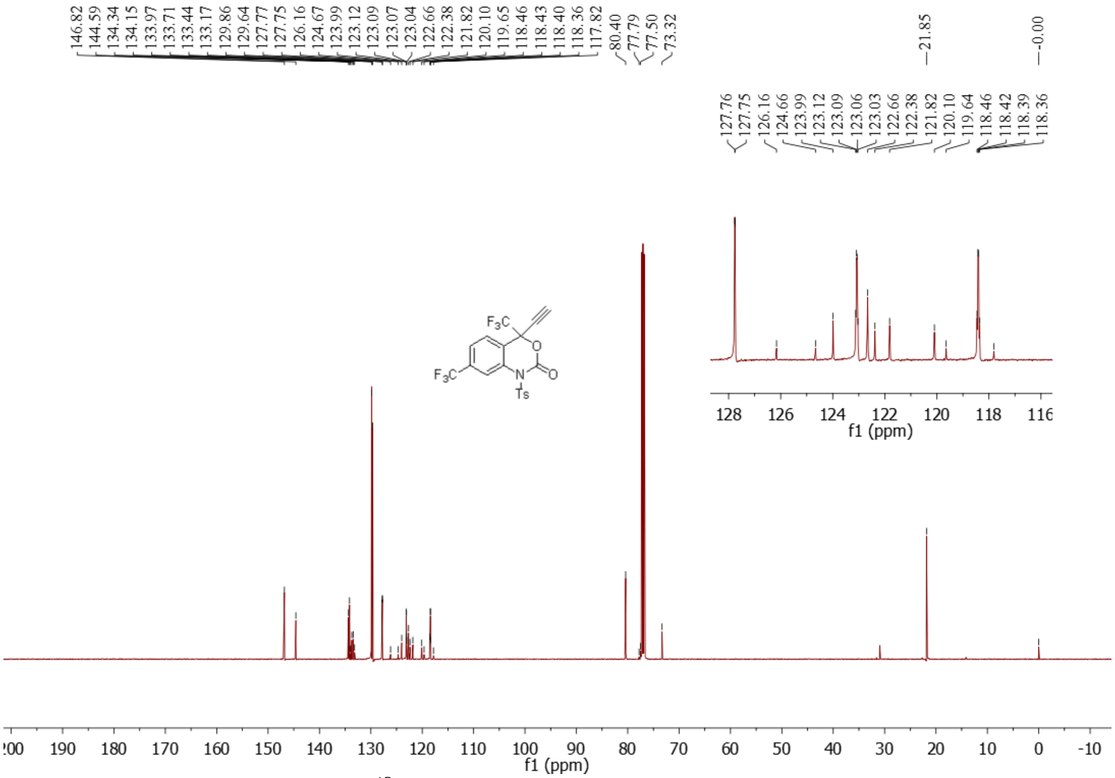

Figure S43. ^{13}C NMR spectrum of **4c**, related to **Scheme 6**.

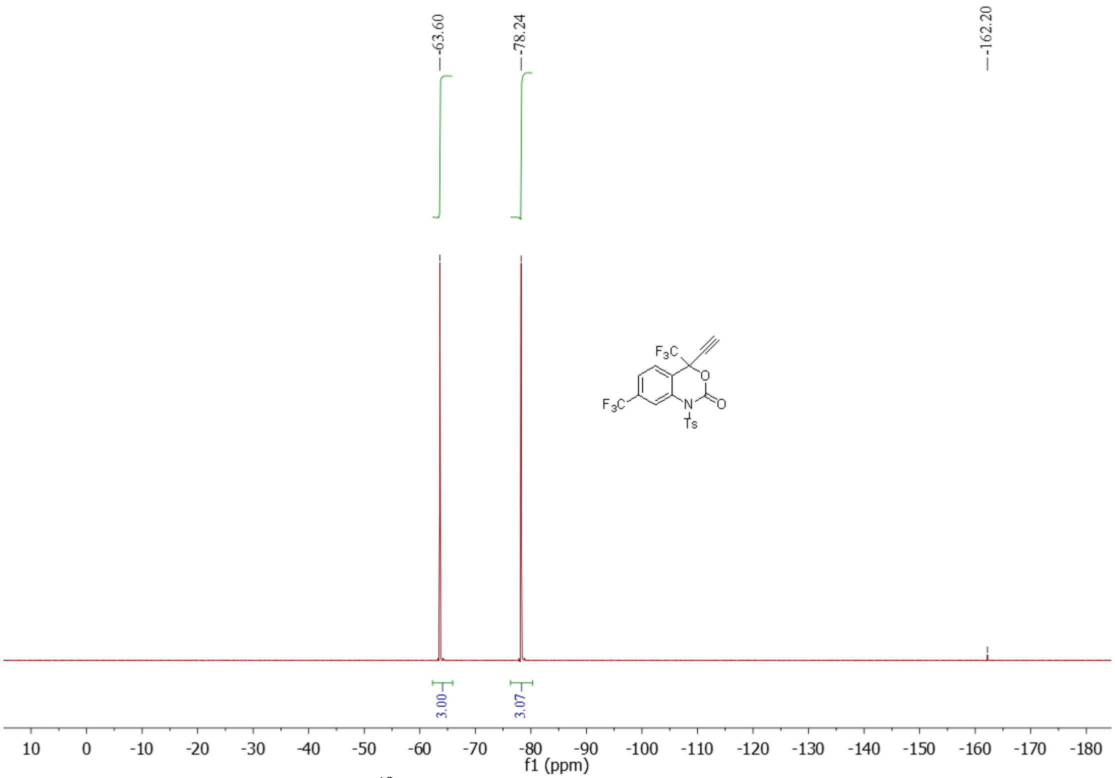

Figure S44. ^{19}F NMR spectrum of **4c**, related to **Scheme 6**.

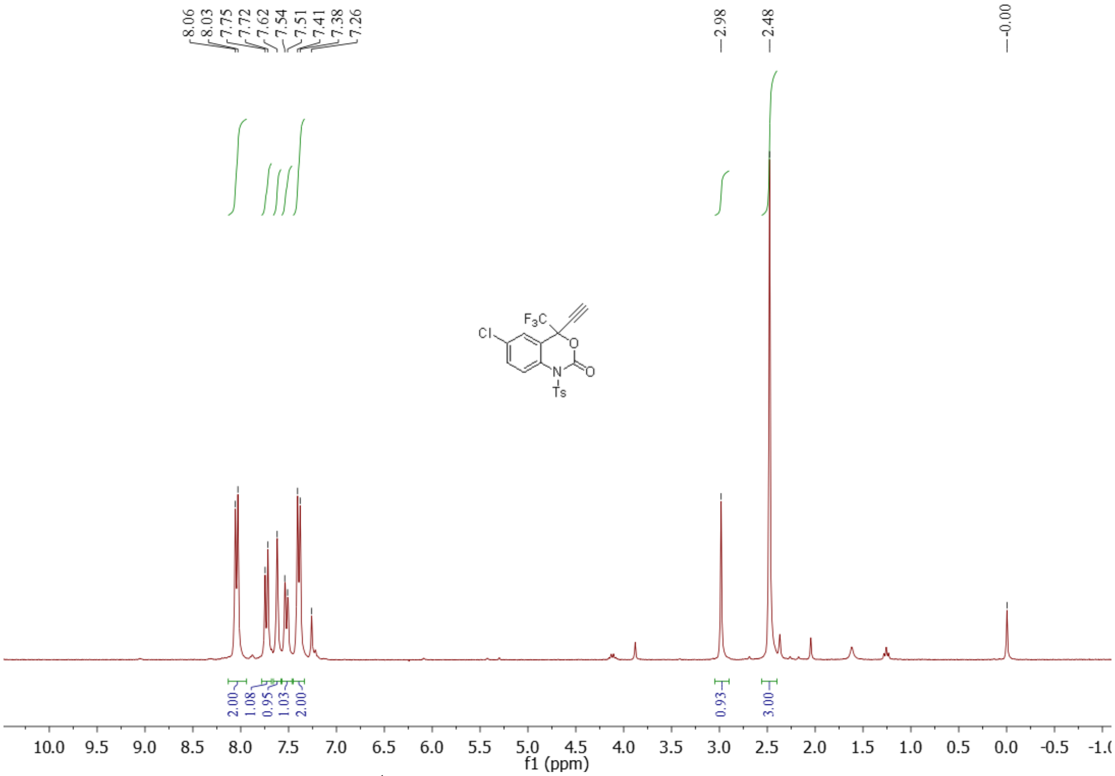

Figure S45. ^{1}H NMR spectrum of **4d**, related to **Scheme 6**.

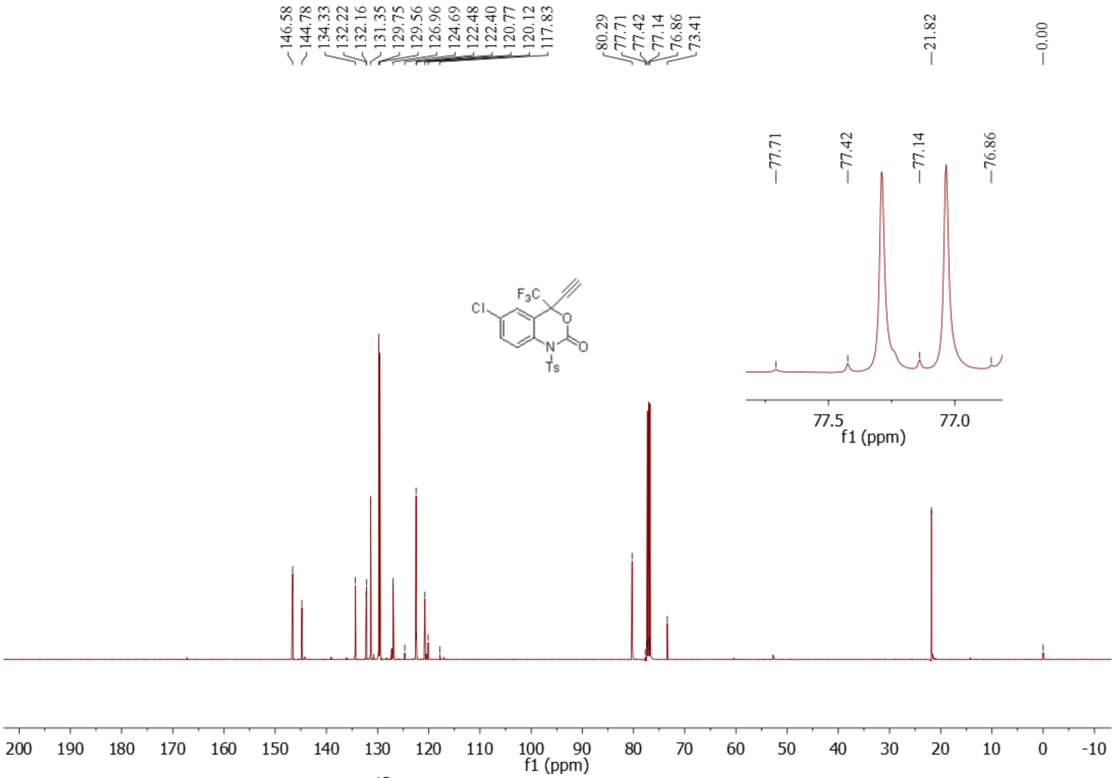

Figure S46. ^{13}C NMR spectrum of **4d**, related to **Scheme 6**.

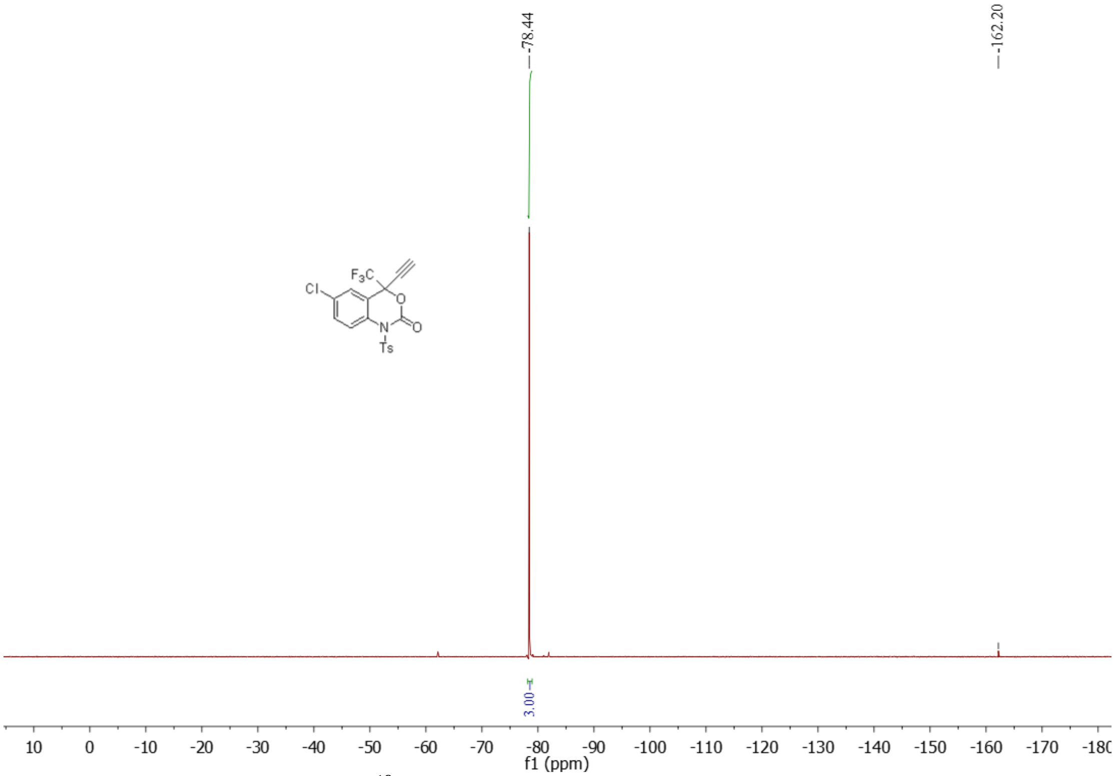

Figure S47. ^{19}F NMR spectrum of **4d**, related to **Scheme 6**.

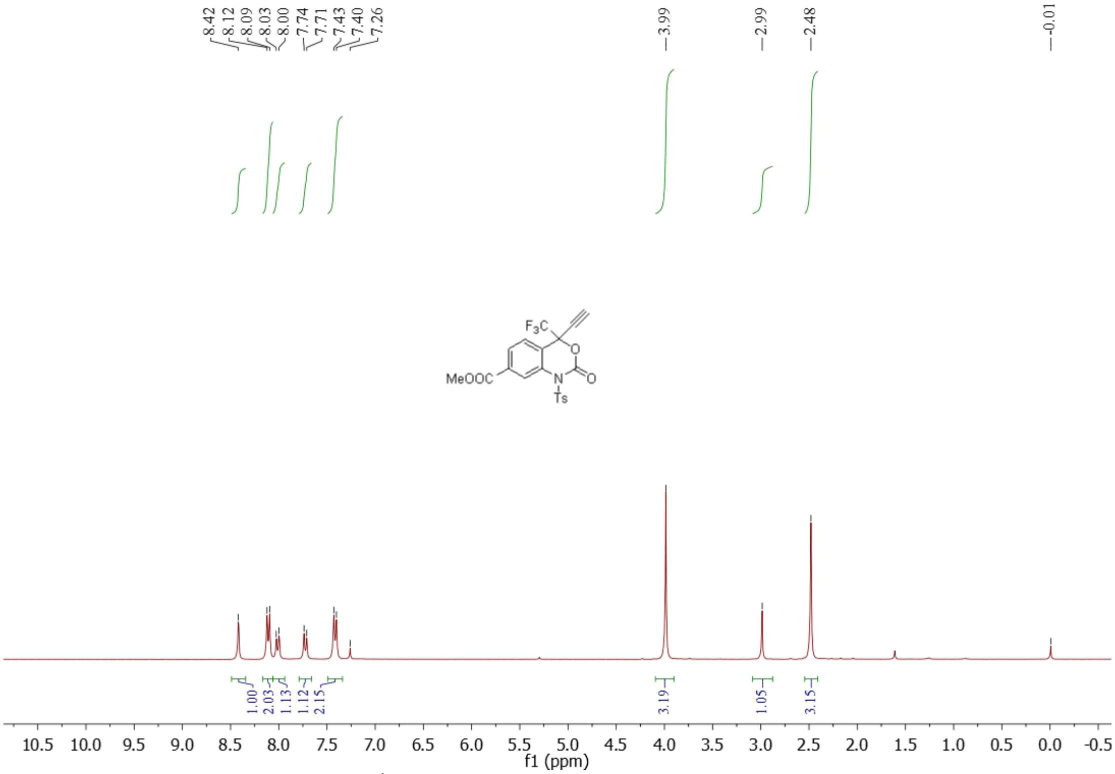

Figure S48. ^{1}H NMR spectrum of **4g**, related to **Scheme 6**.

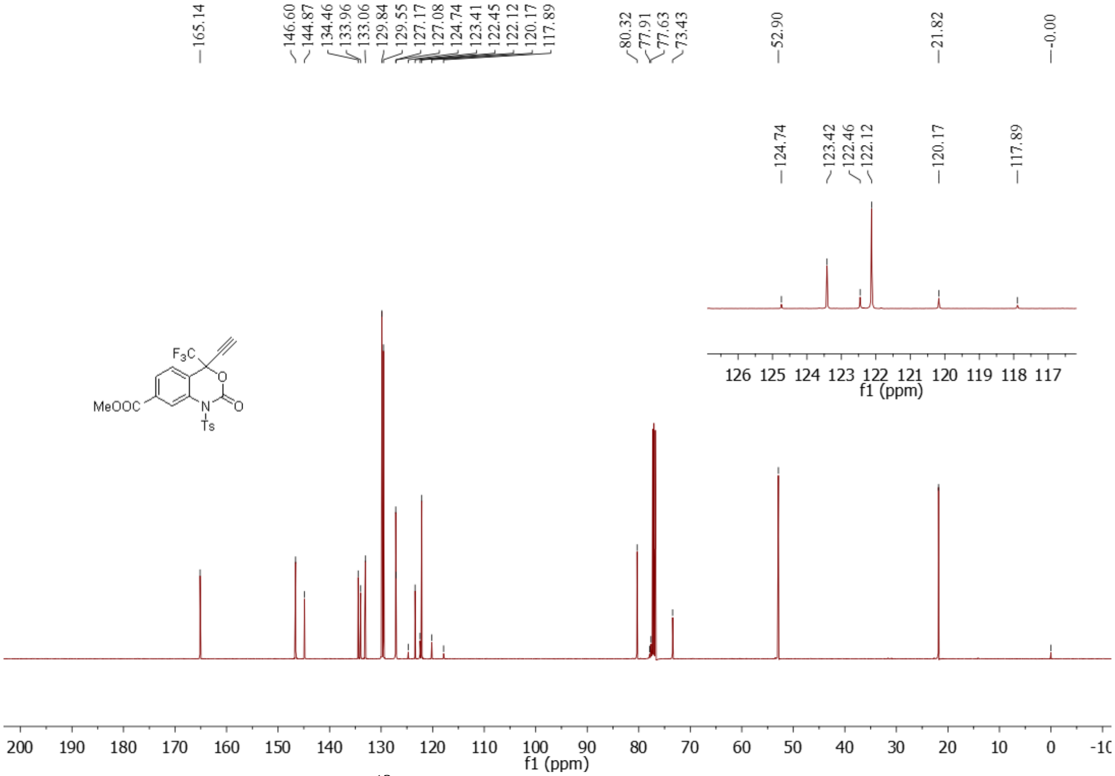

Figure S49. ${}^{13}C$ NMR spectrum of **4g**, related to **Scheme 6**.

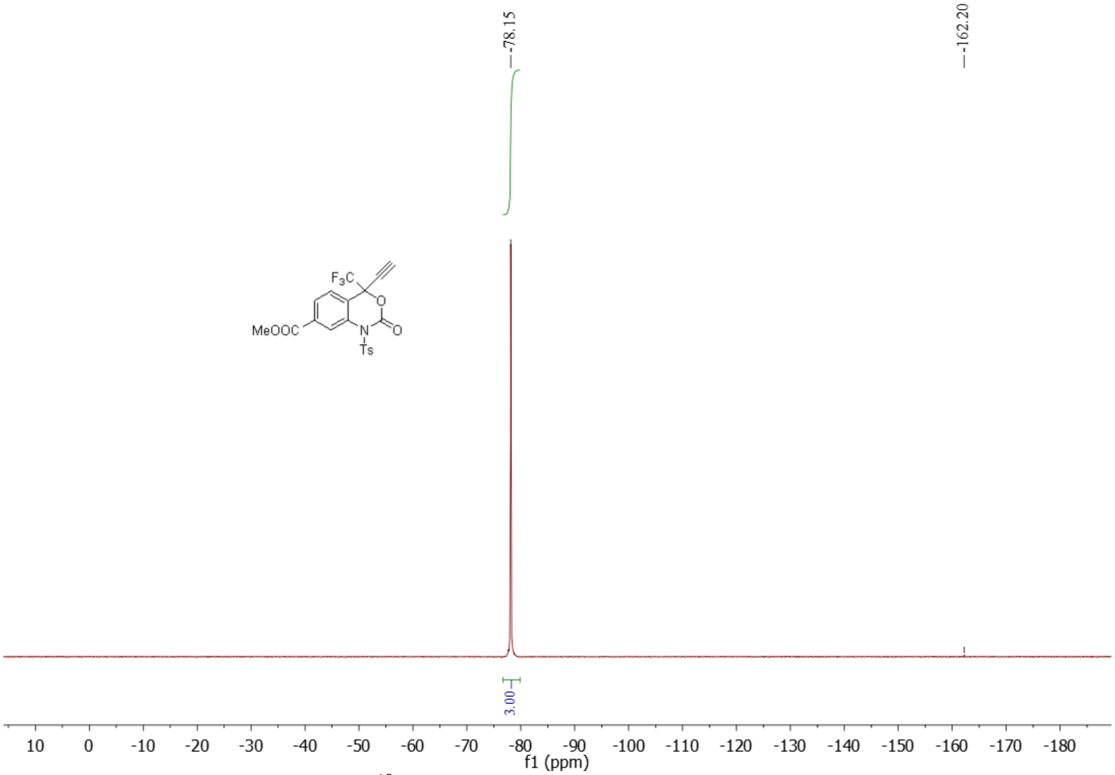

Figure S50. ${}^{19}F$ NMR spectrum of **4g**, related to **Scheme 6**.

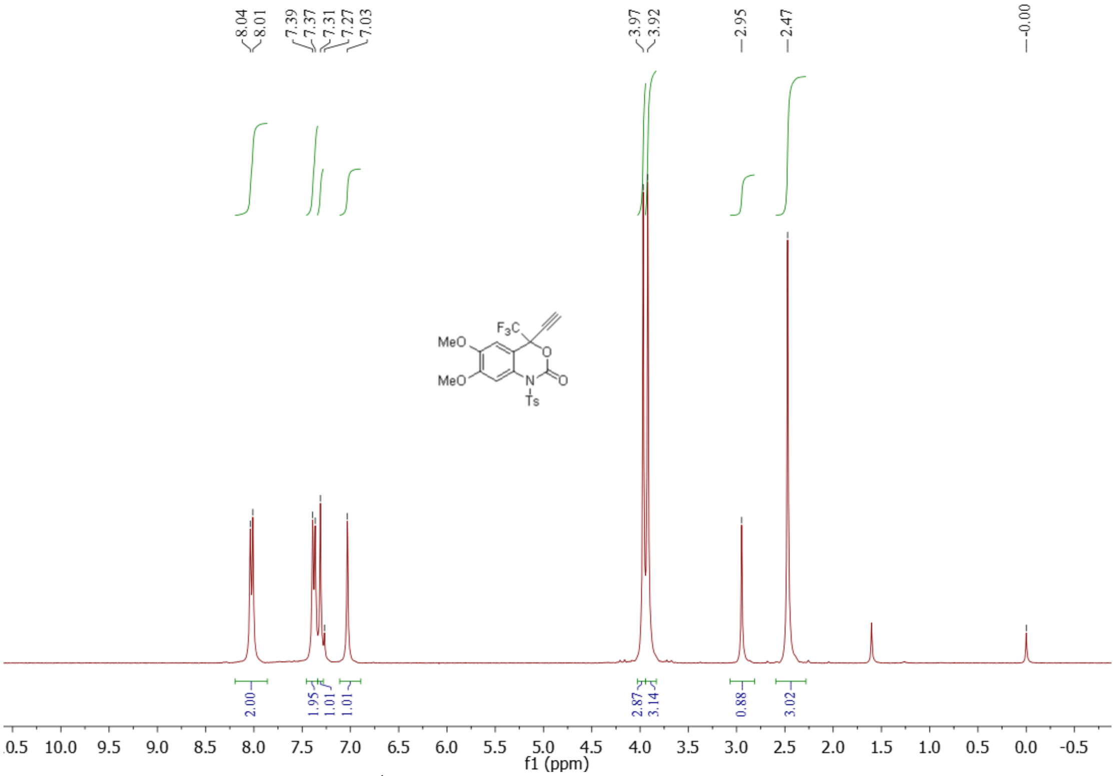

Figure S51. ^{1}H NMR spectrum of **4h**, related to **Scheme 6**.

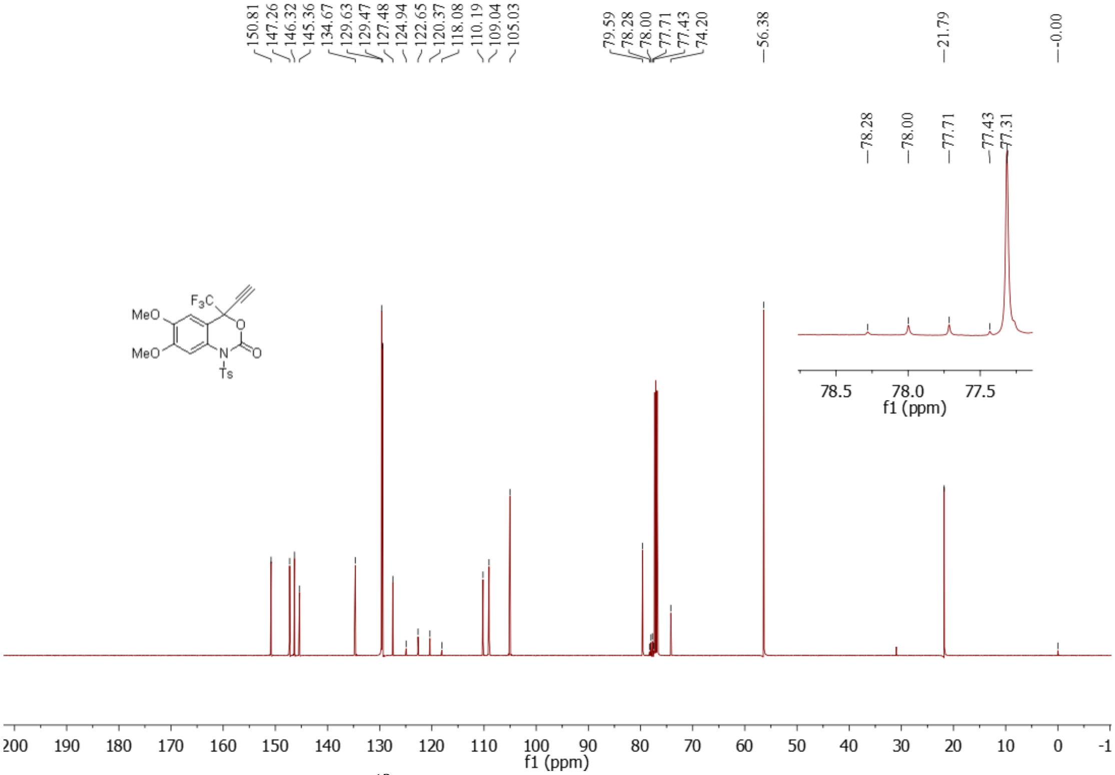

Figure S52. ^{13}C NMR spectrum of **4h**, related to **Scheme 6**.

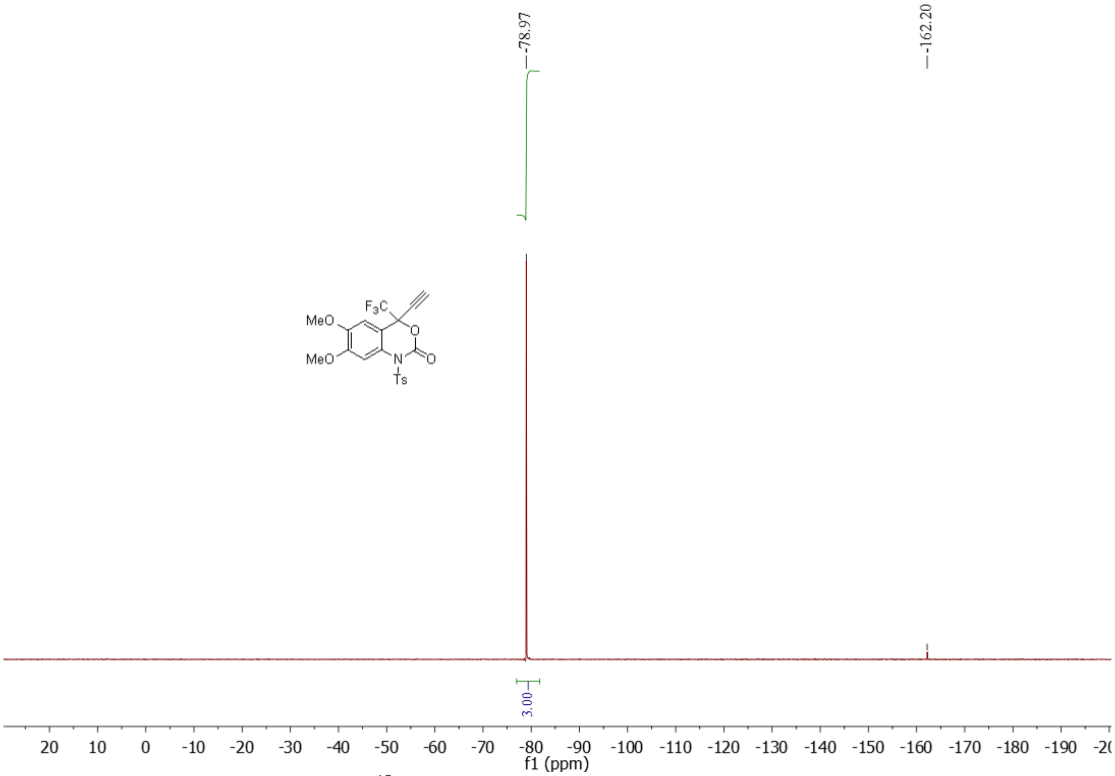

Figure S53. ^{19}F NMR spectrum of **4h**, related to **Scheme 6.**

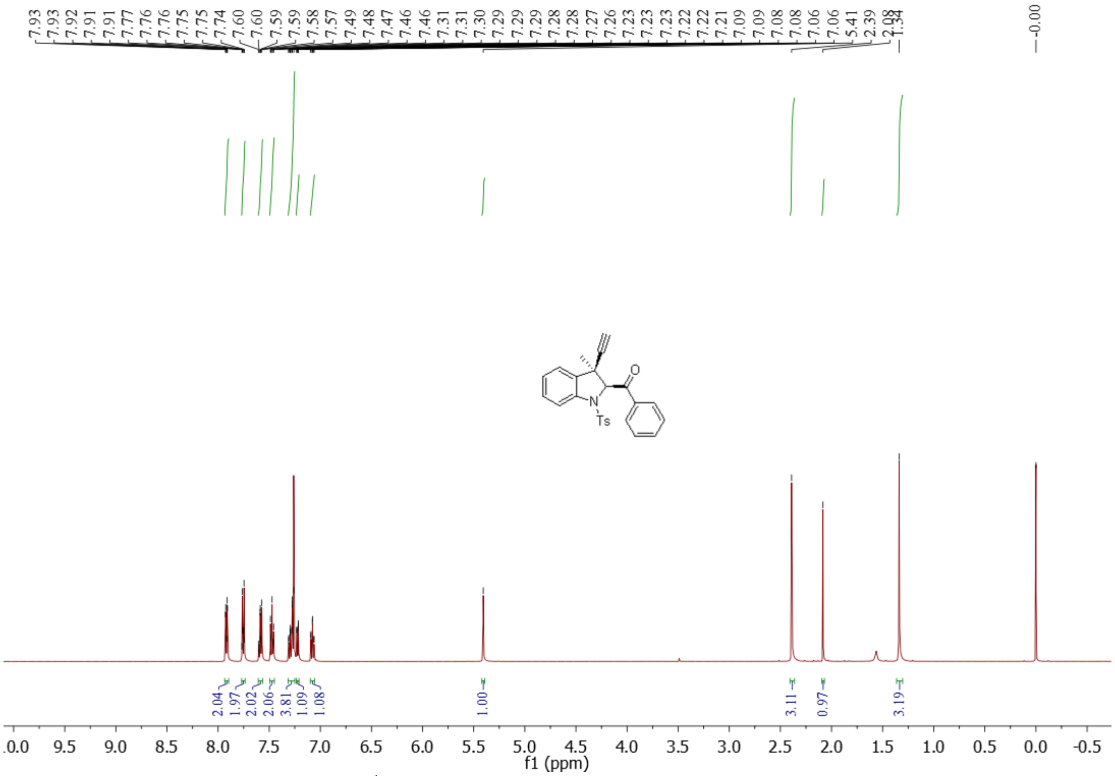

Figure S54. ^{1}H NMR spectrum of **5aa**, related to **Scheme 4**.

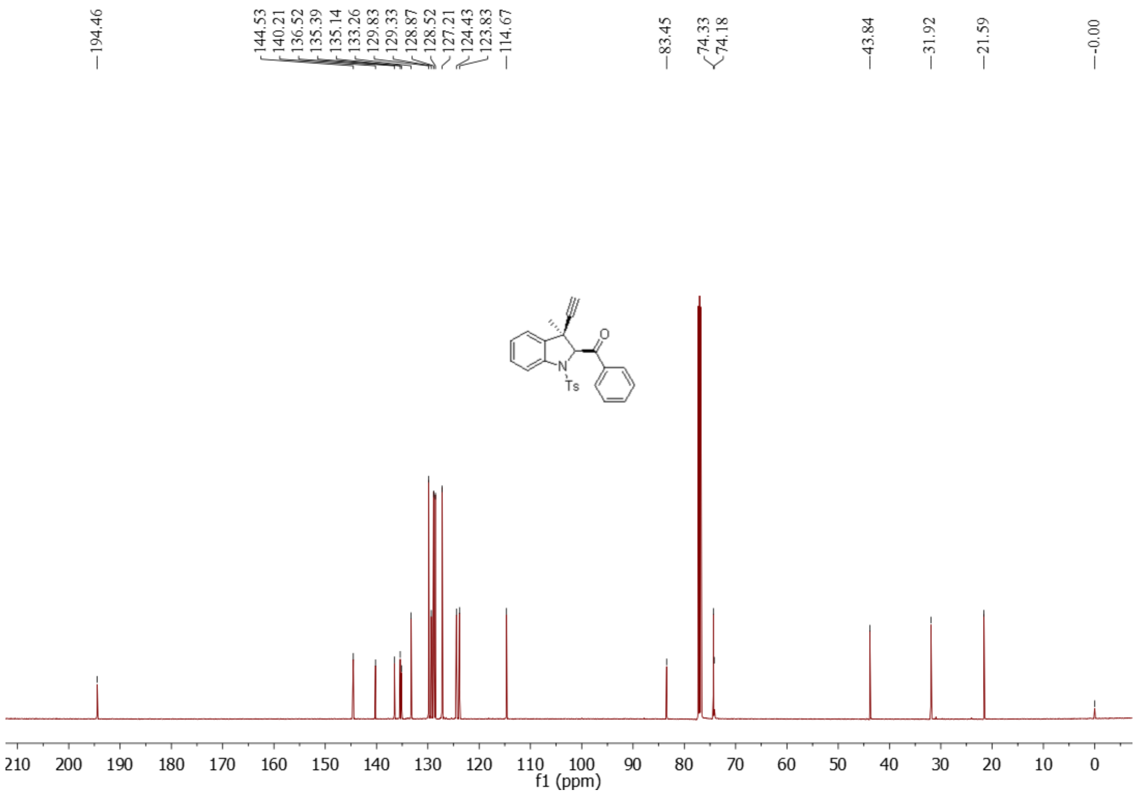

Figure S55. ^{13}C NMR spectrum of **5aa**, related to **Scheme 4**.

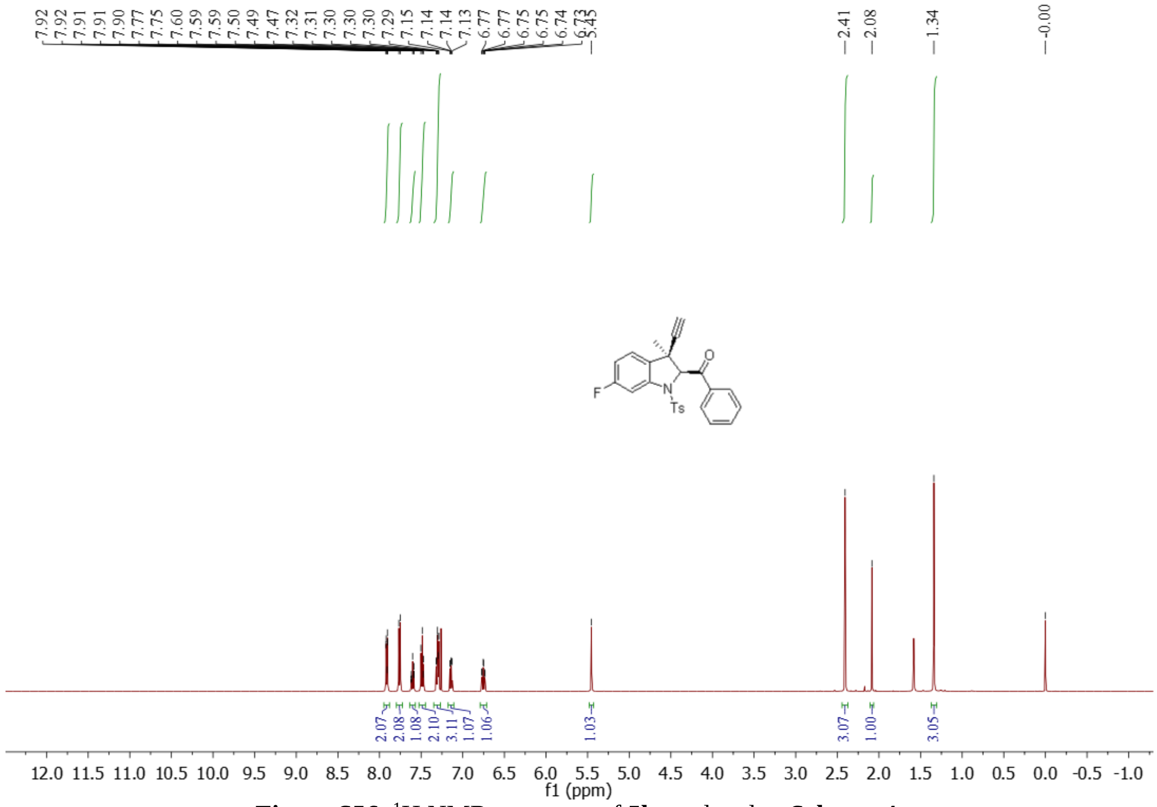

Figure S56. ^{1}H NMR spectrum of **5ba**, related to **Scheme 4**.

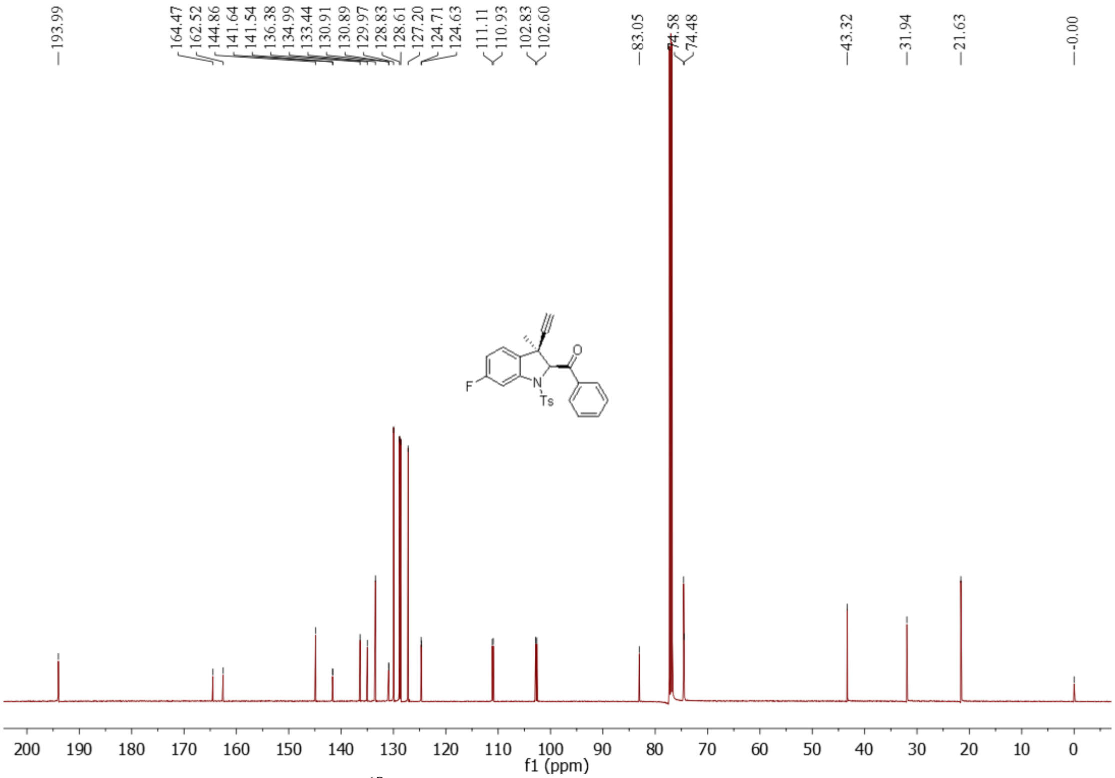

Figure S57. ^{13}C NMR spectrum of **5ba**, related to **Scheme 4**.

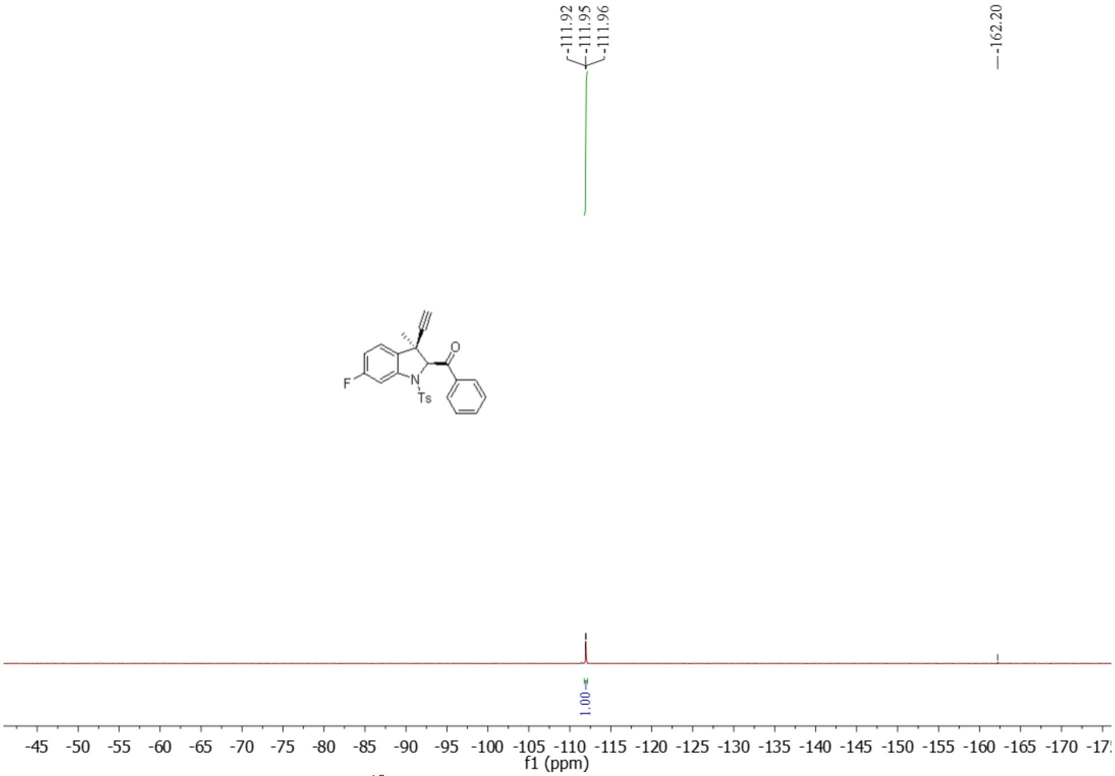

Figure S58. ^{19}F NMR spectrum of **5ba**, related to **Scheme 4**.

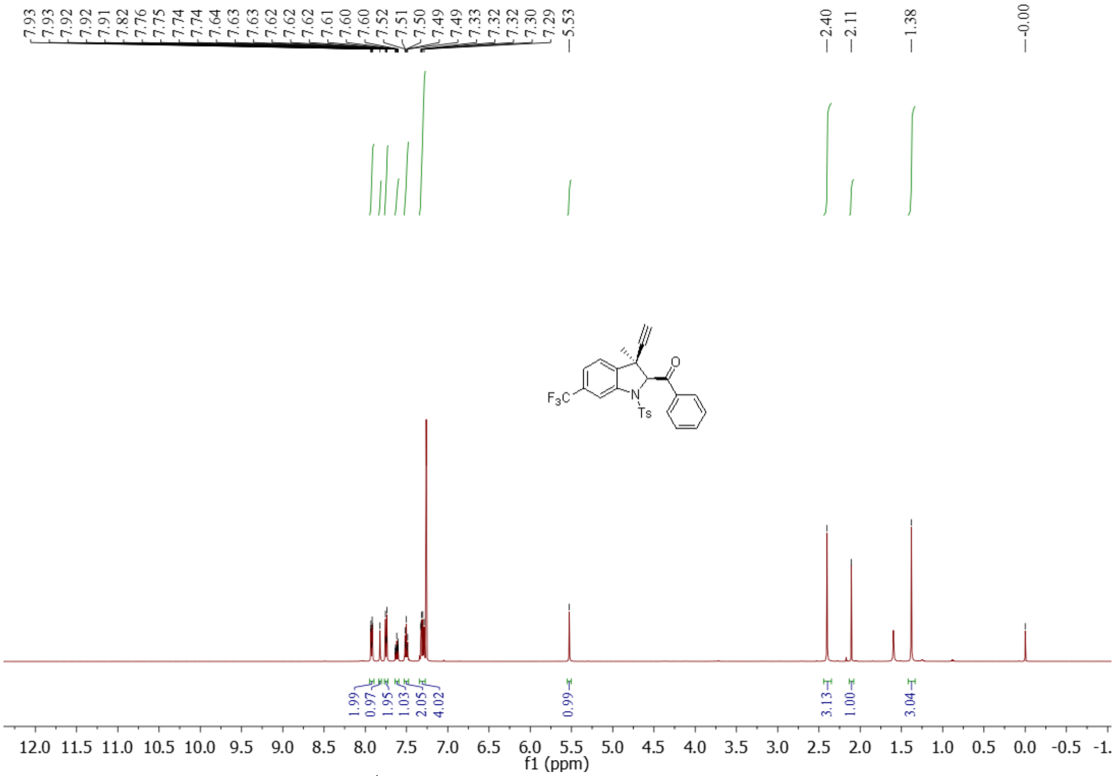

Figure S59. ^{1}H NMR spectrum of **5ca**, related to **Scheme 4**.

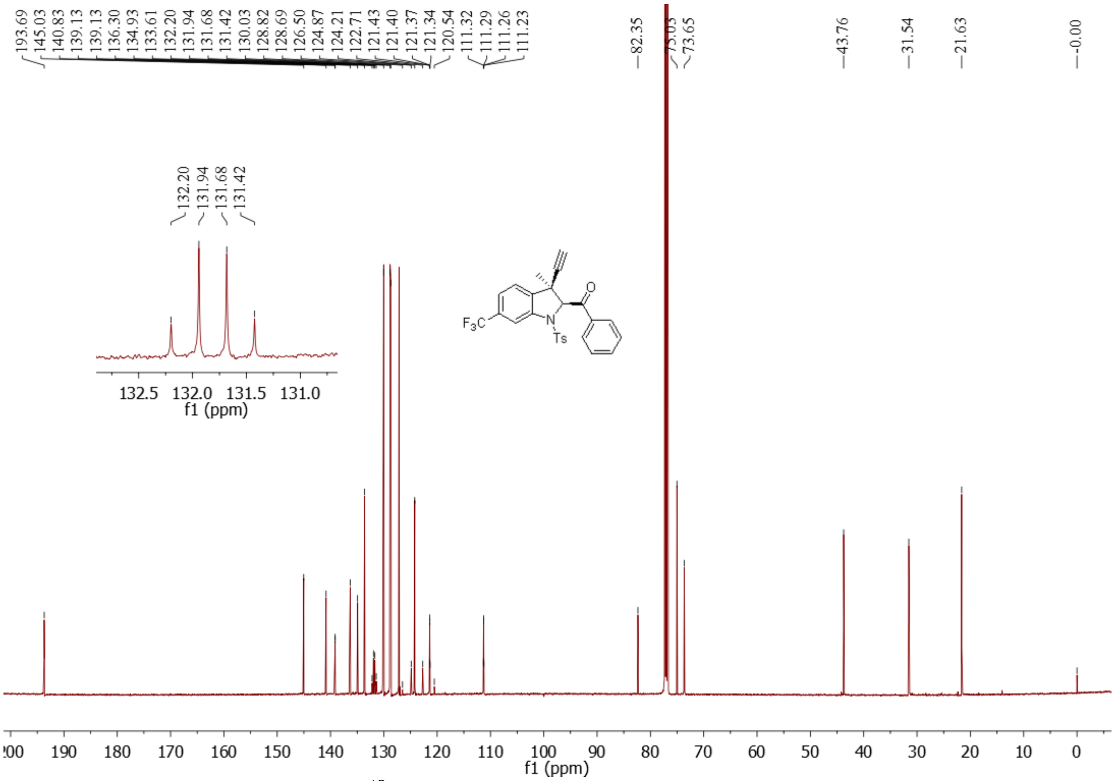

Figure S60. ^{13}C NMR spectrum of **5ca**, related to **Scheme 4**.

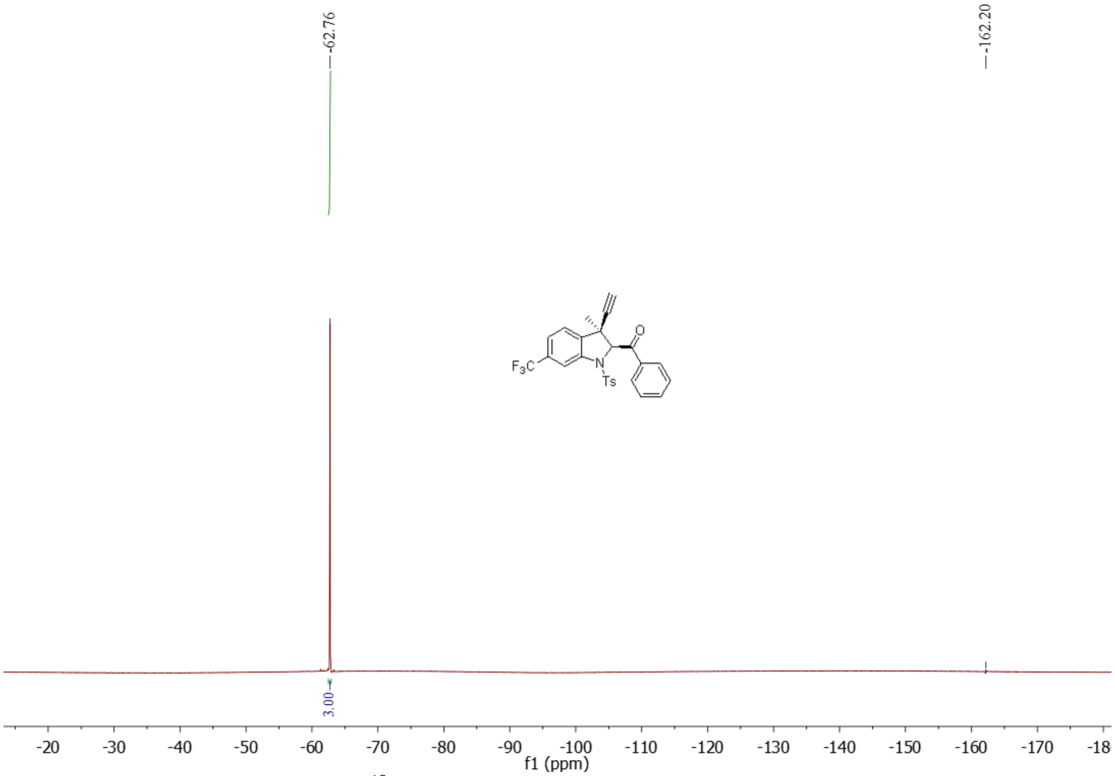

Figure S61. ^{19}F NMR spectrum of **5ca**, related to **Scheme 4**.

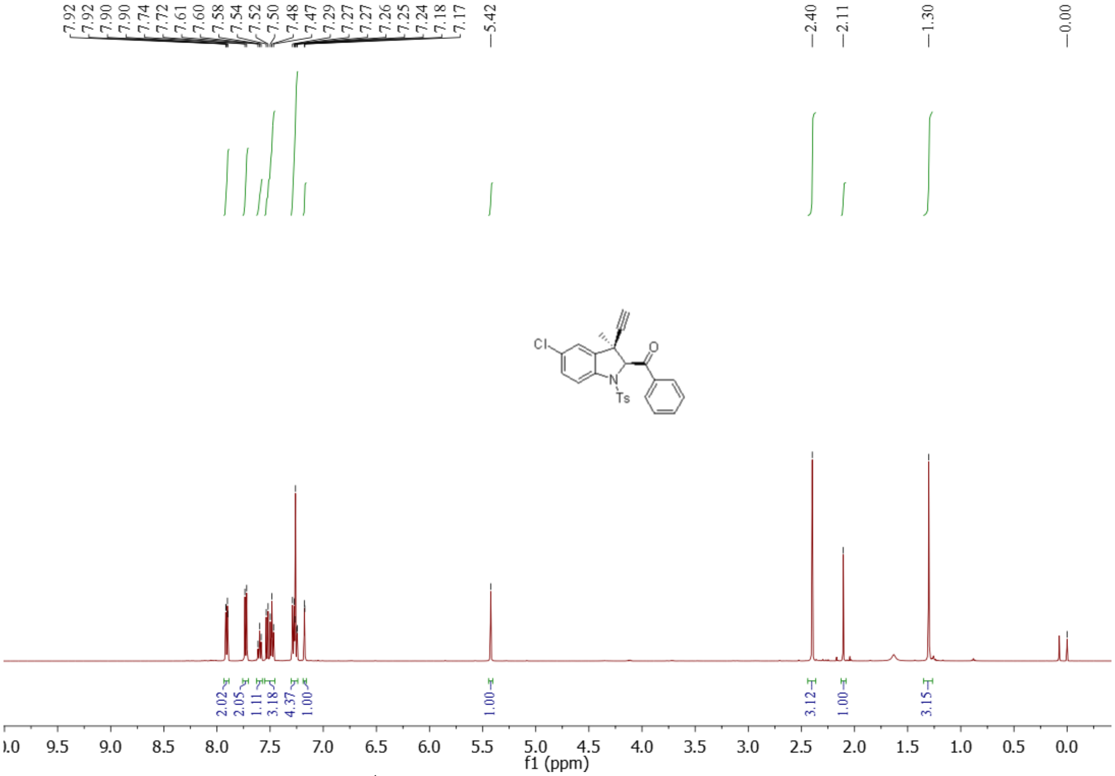

Figure S62. ^{1}H NMR spectrum of **5da**, related to **Scheme 4**.

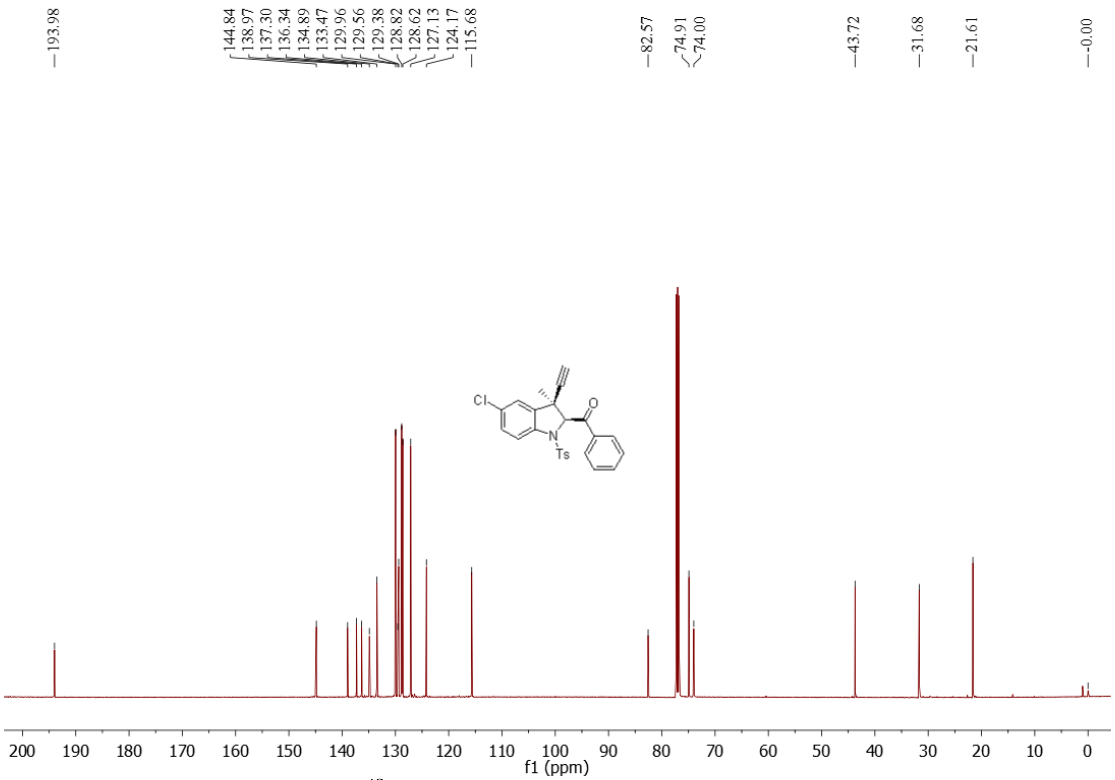

Figure S63. ^{13}C NMR spectrum of **5da**, related to **Scheme 4**.

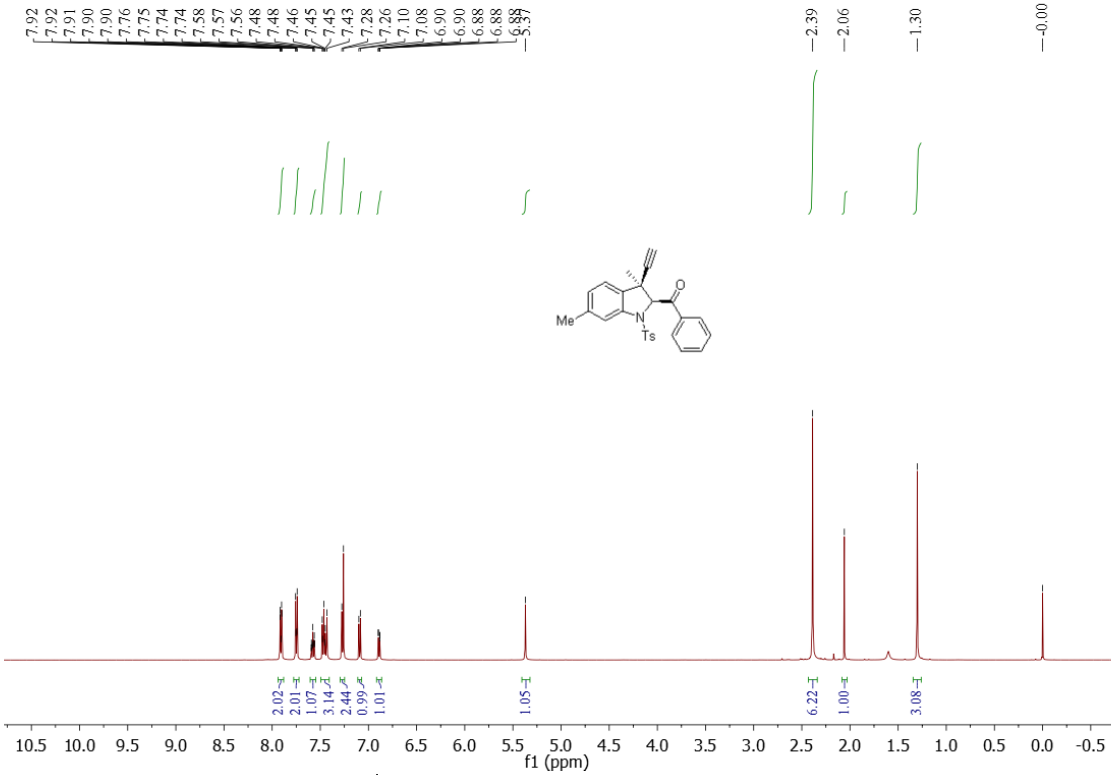

Figure S64. ^{1}H NMR spectrum of **5ea**, related to **Scheme 4**.

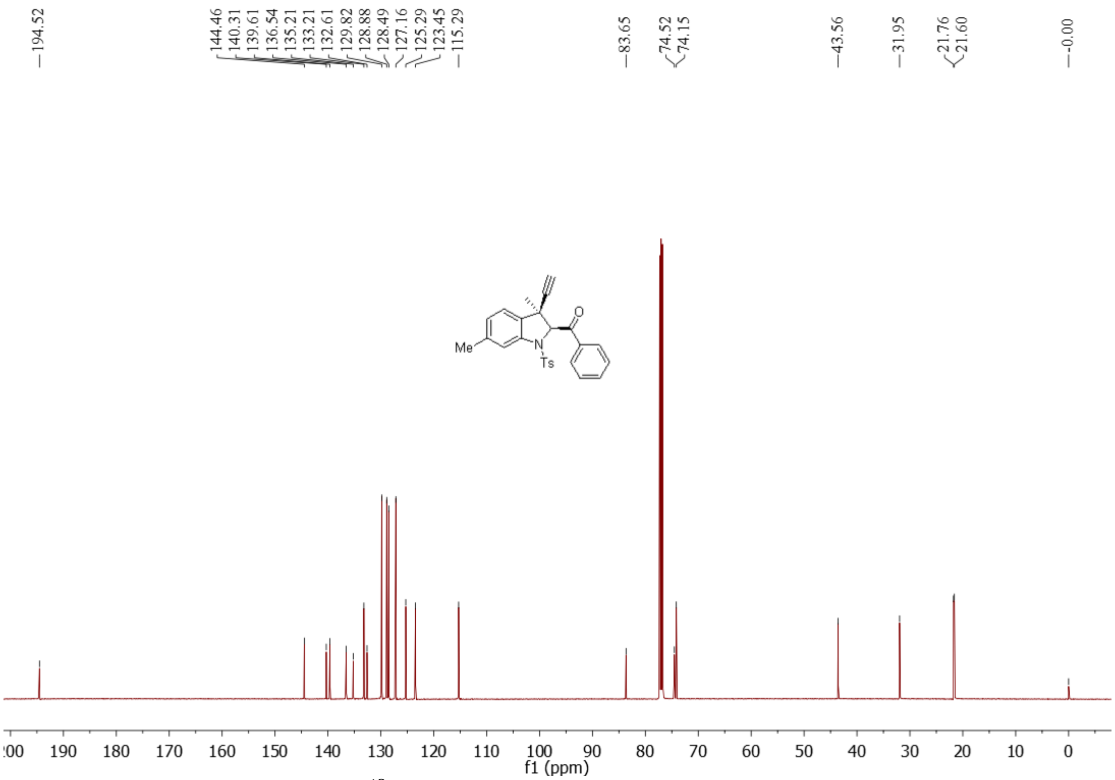

Figure S65. ^{13}C NMR spectrum of **5ea**, related to **Scheme 4**.

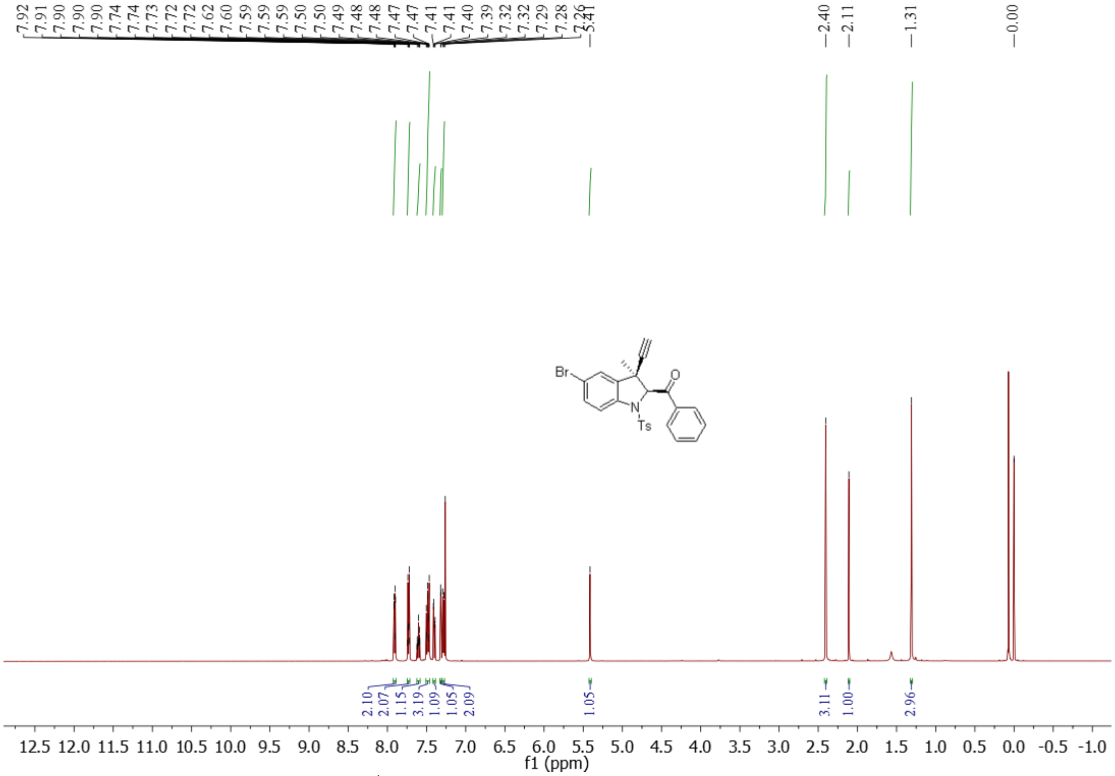

Figure S66. ^{1}H NMR spectrum of **5fa**, related to **Scheme 4**.

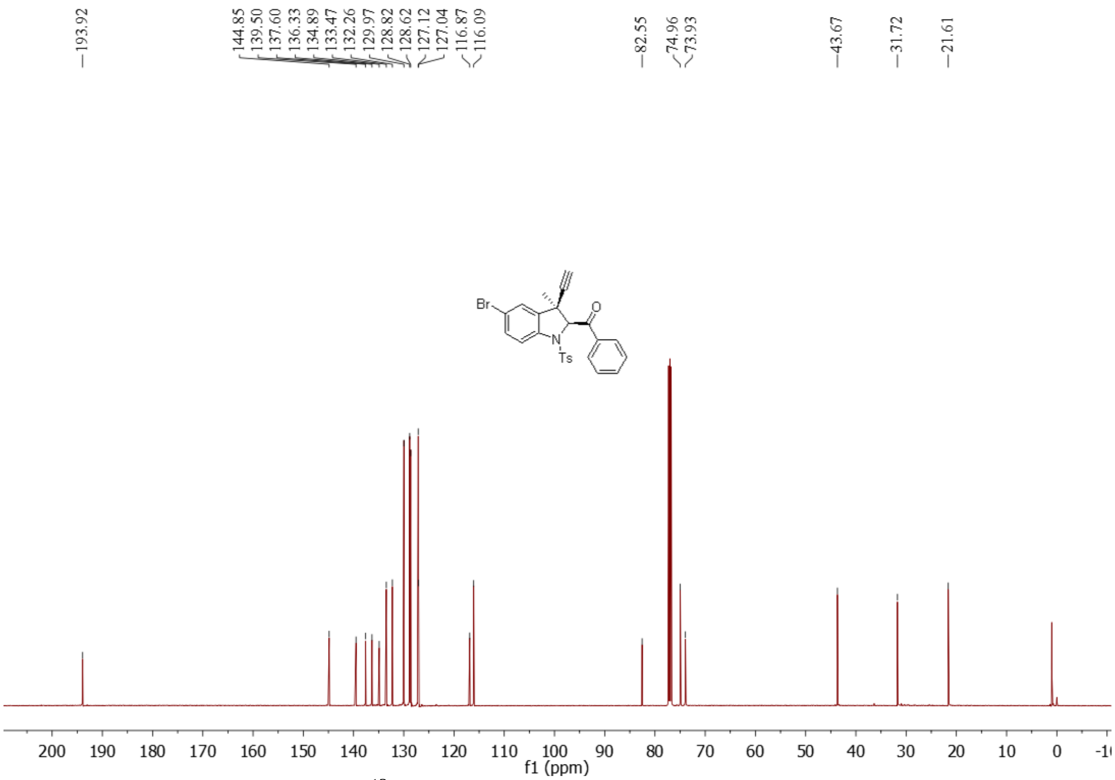

Figure S67. ^{13}C NMR spectrum of **5fa**, related to **Scheme 4**.

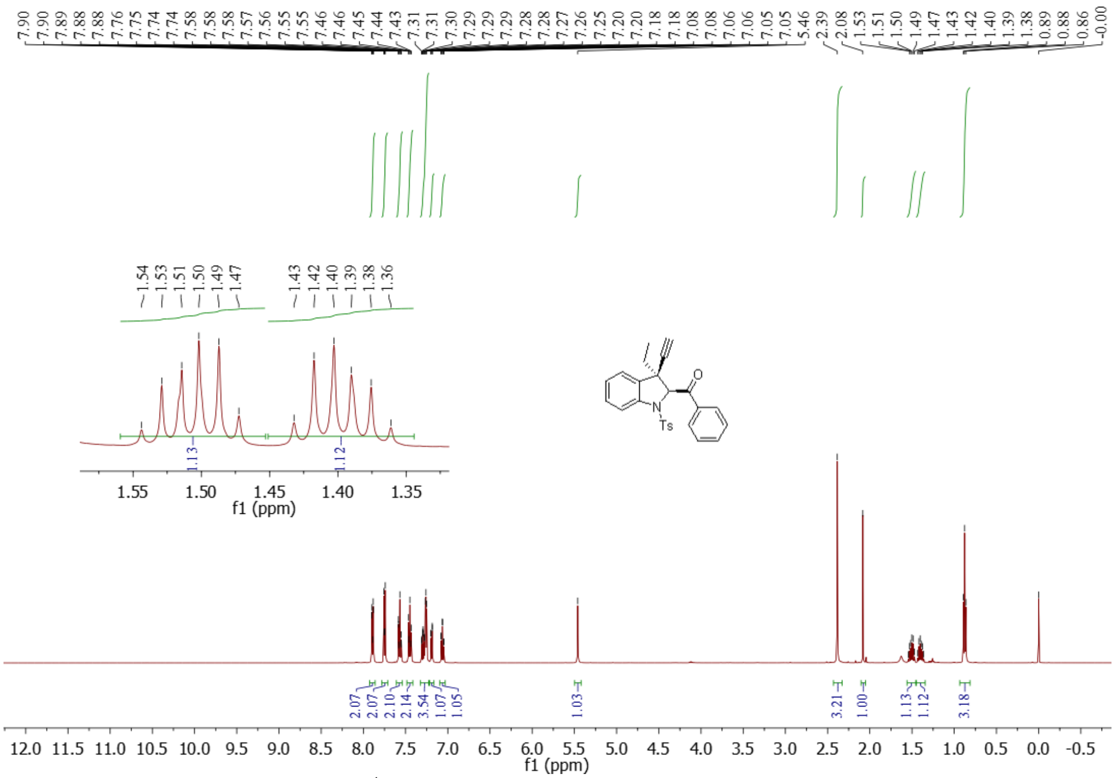

Figure S68. ^{1}H NMR spectrum of **5ga**, related to **Scheme 4**.

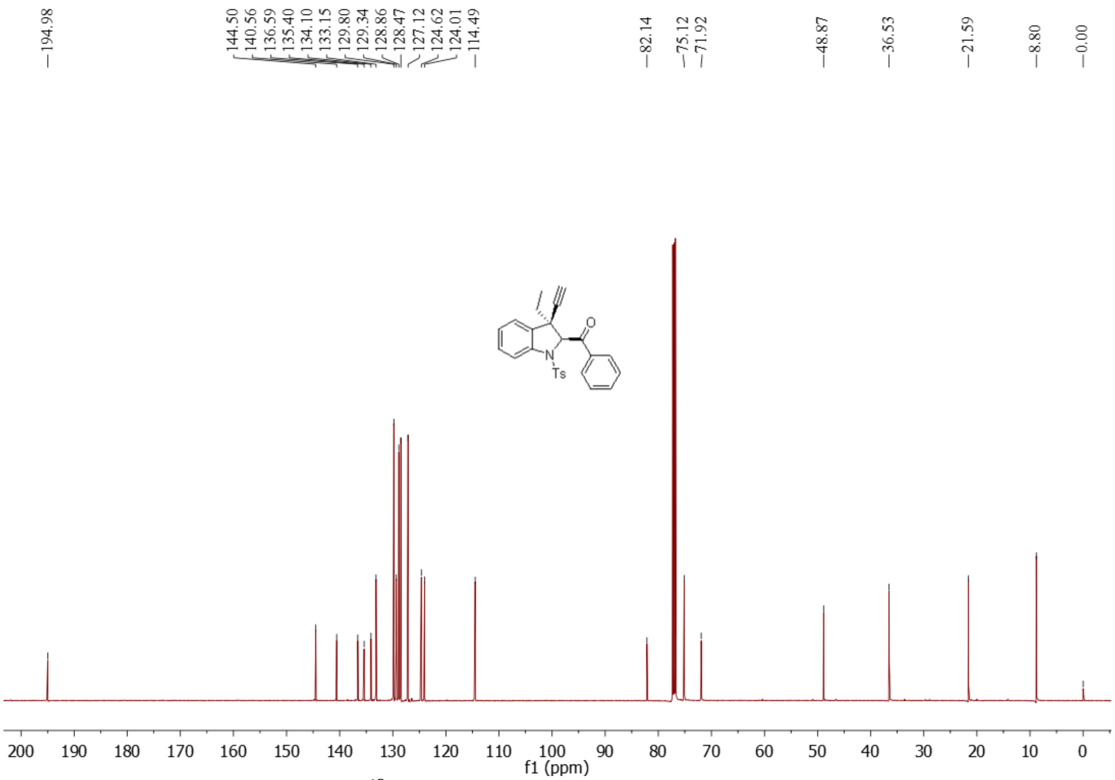

Figure S69. ^{13}C NMR spectrum of **5ga**, related to **Scheme 4**.

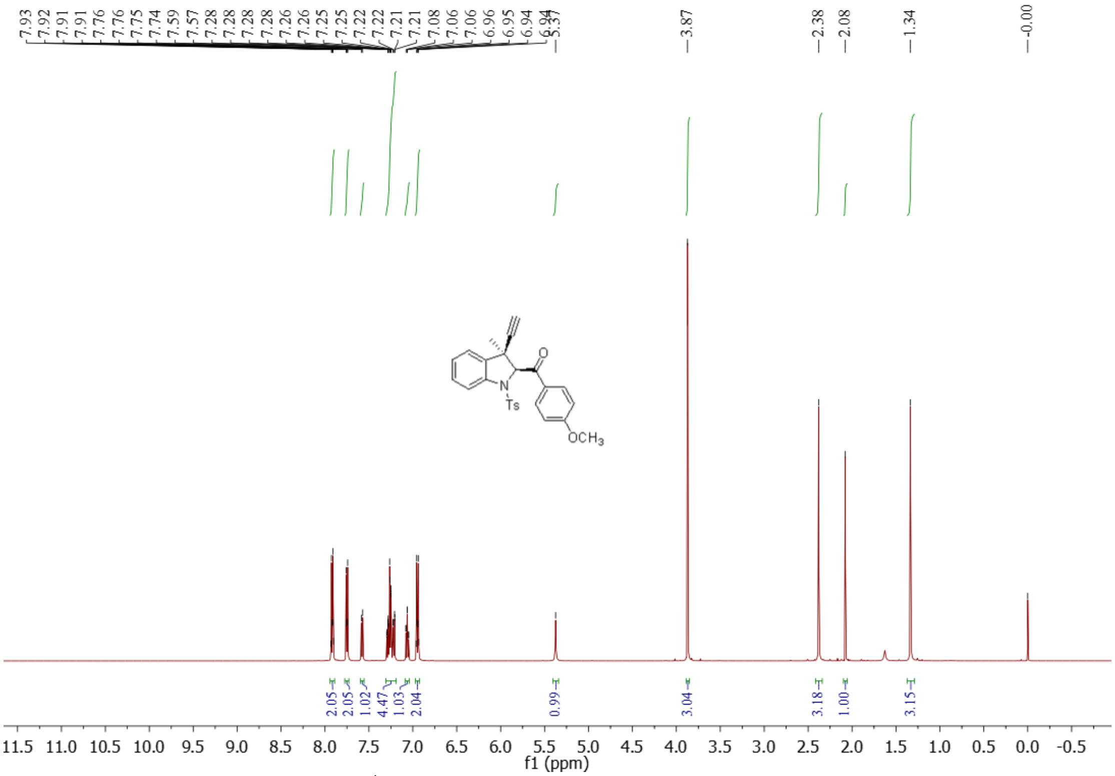

Figure S70. ^{1}H NMR spectrum of **5ab**, related to **Scheme 4**.

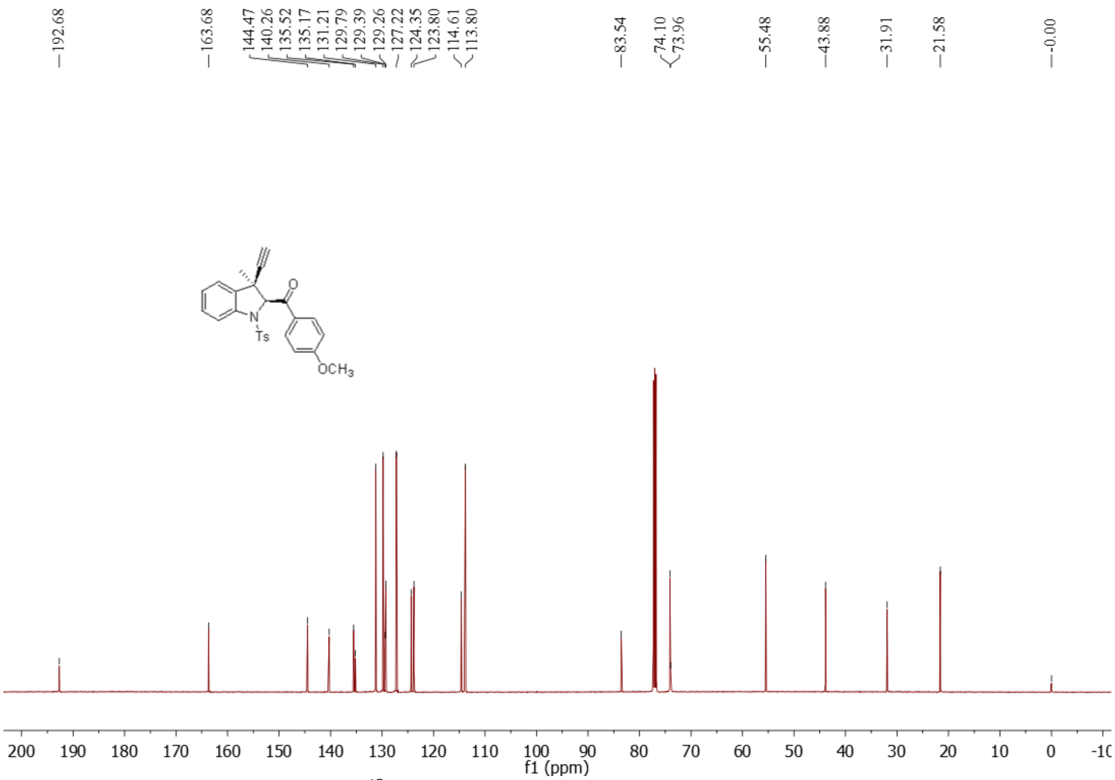

Figure S71. ^{13}C NMR spectrum of **5ab**, related to **Scheme 4**.

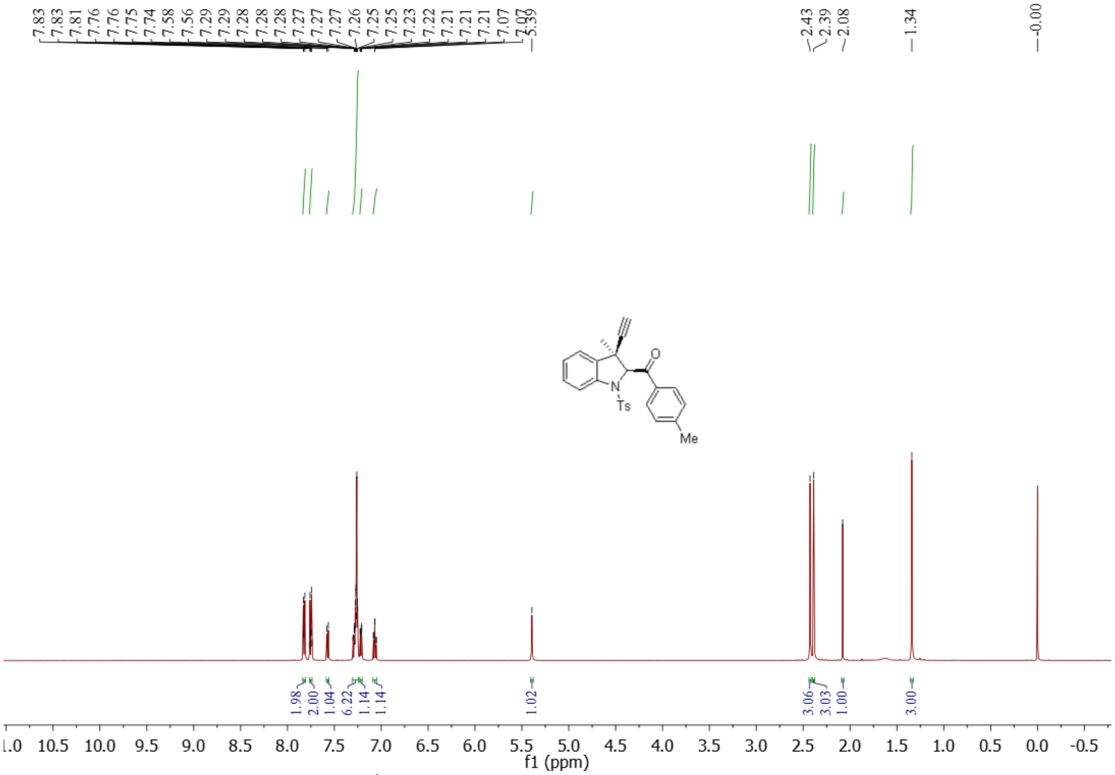

Figure S72. ^{1}H NMR spectrum of **5ac**, related to **Scheme 4**.

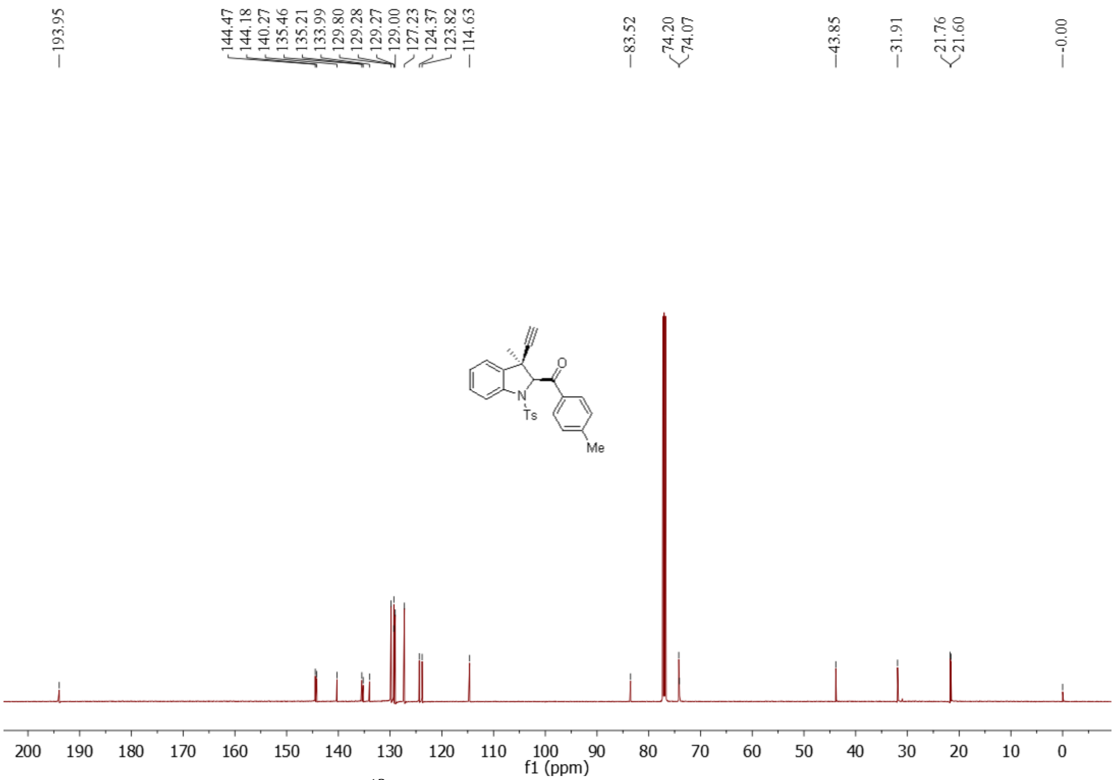

Figure S73. ^{13}C NMR spectrum of **5ac**, related to **Scheme 4**.

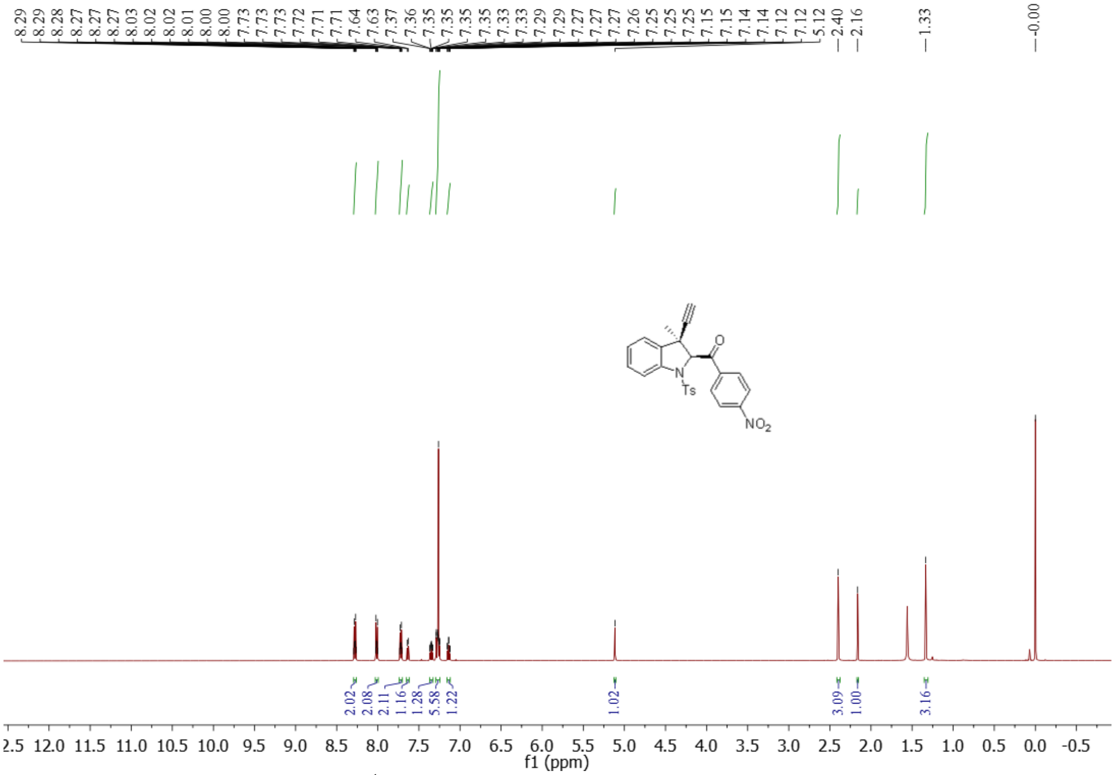

Figure S74. ^{1}H NMR spectrum of **5ad**, related to **Scheme 4**.

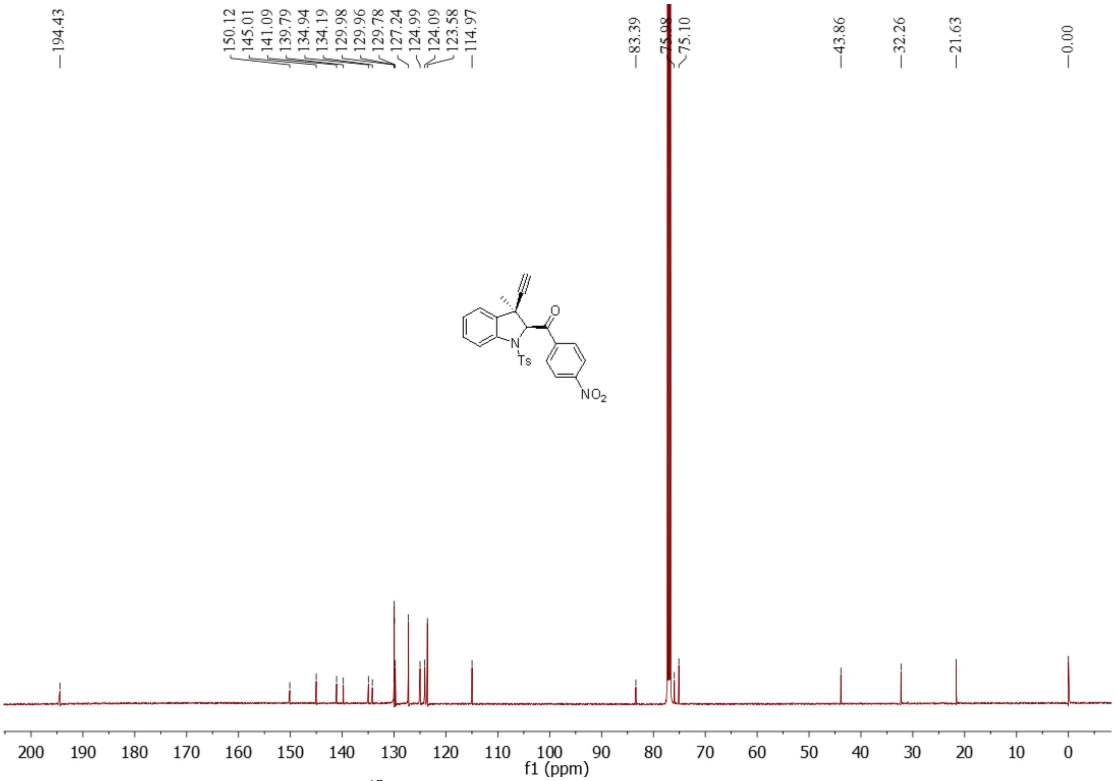

Figure S75. ^{13}C NMR spectrum of **5ad**, related to **Scheme 4**.

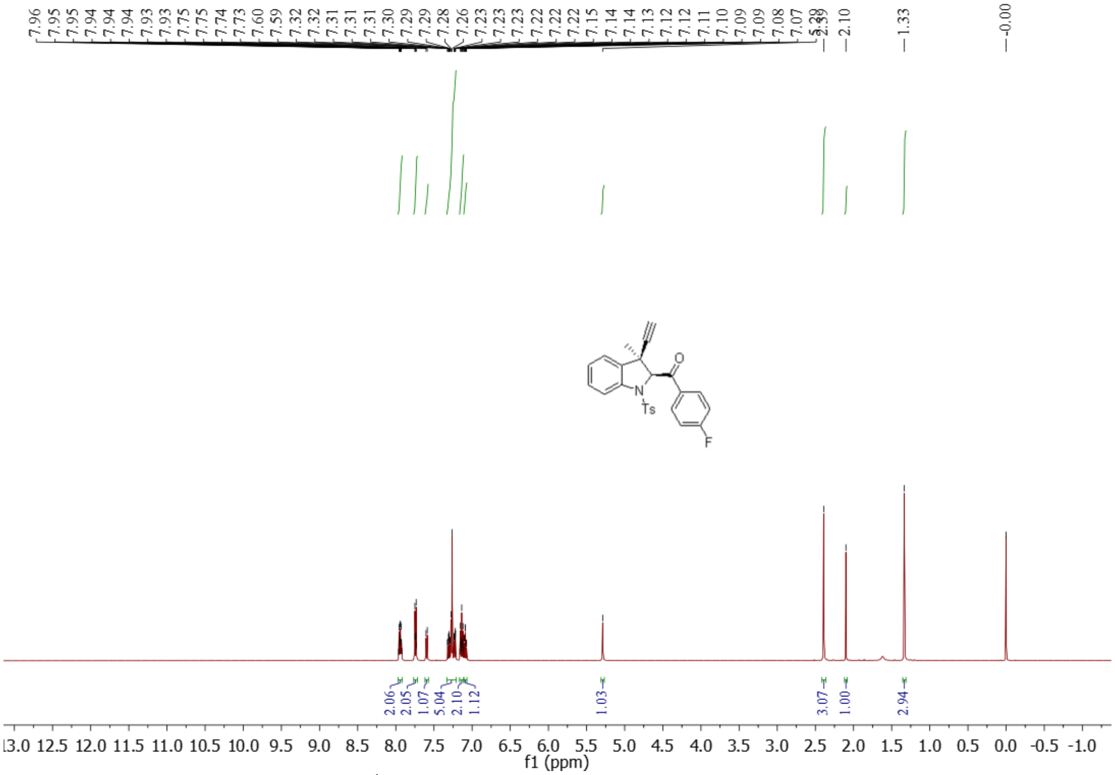

Figure S76. ^{1}H NMR spectrum of **5ae**, related to **Scheme 4**.

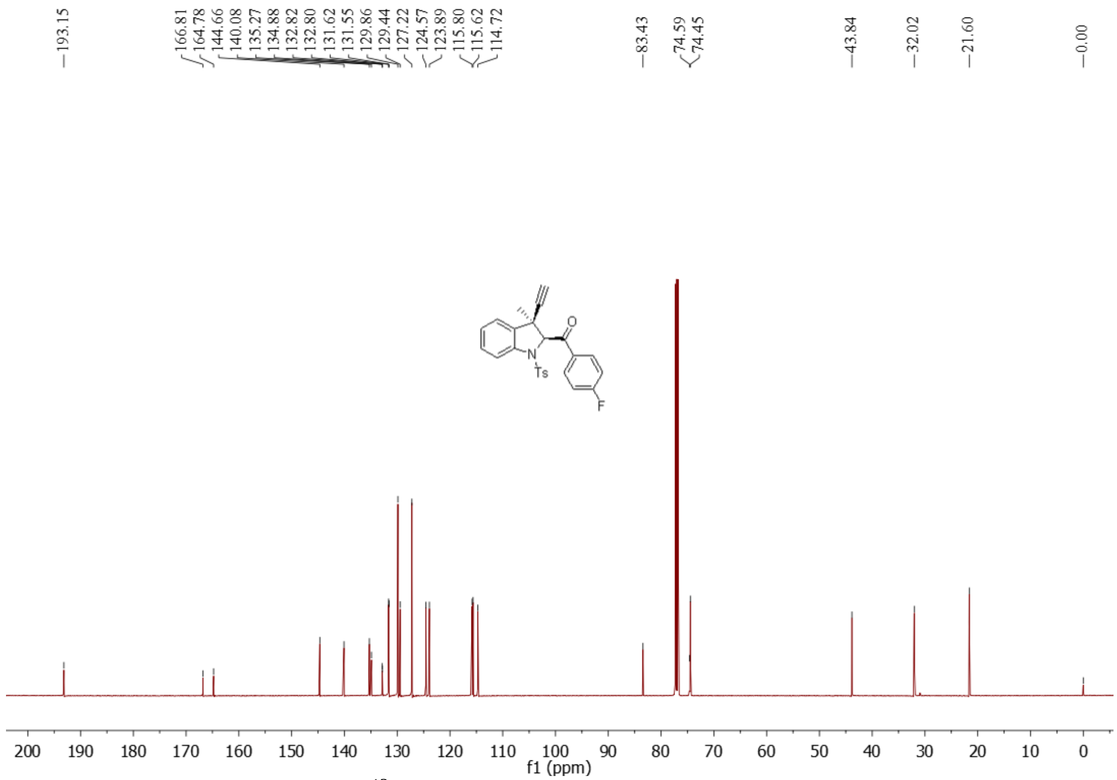

Figure S77. ^{13}C NMR spectrum of **5ae**, related to **Scheme 4**.

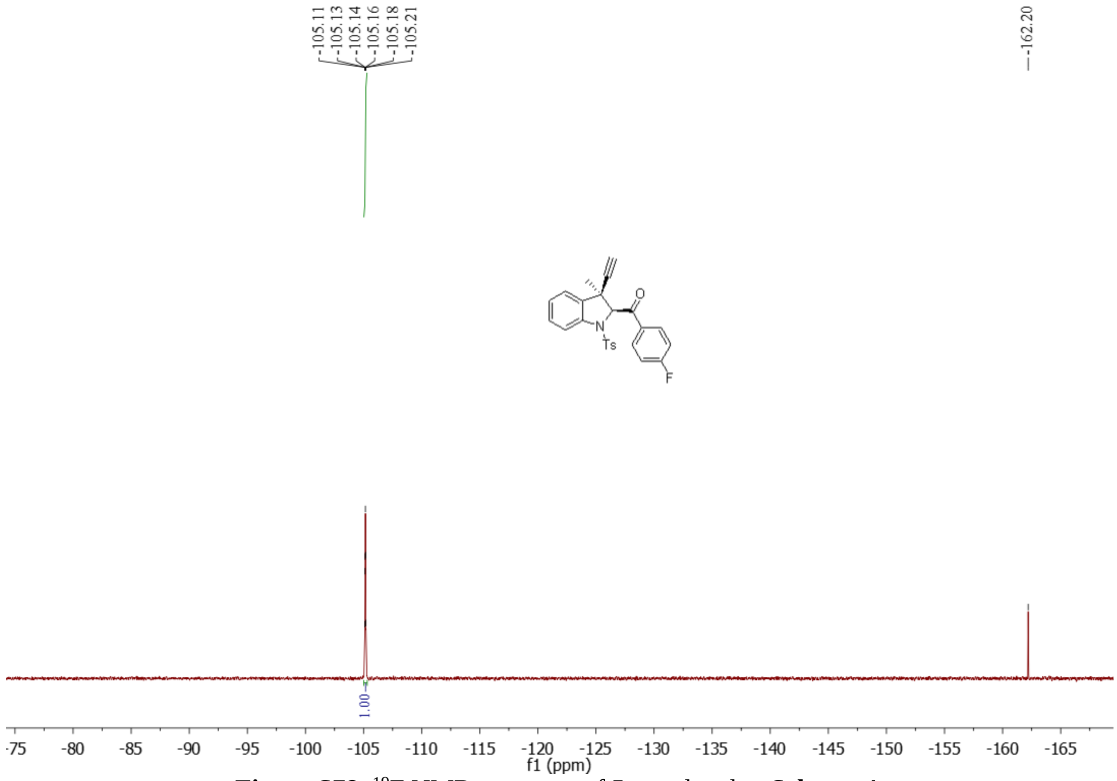

Figure S78. ^{19}F NMR spectrum of **5ae**, related to **Scheme 4**.

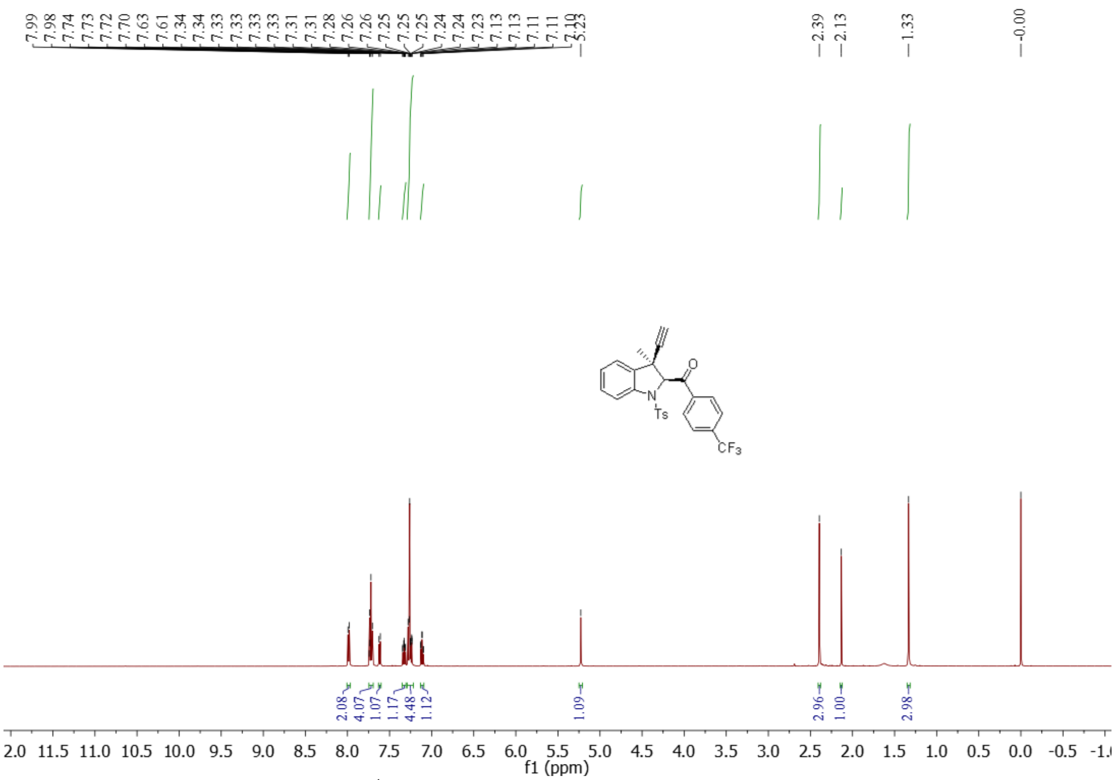

Figure S79. ^{1}H NMR spectrum of **5af**, related to **Scheme 4**.

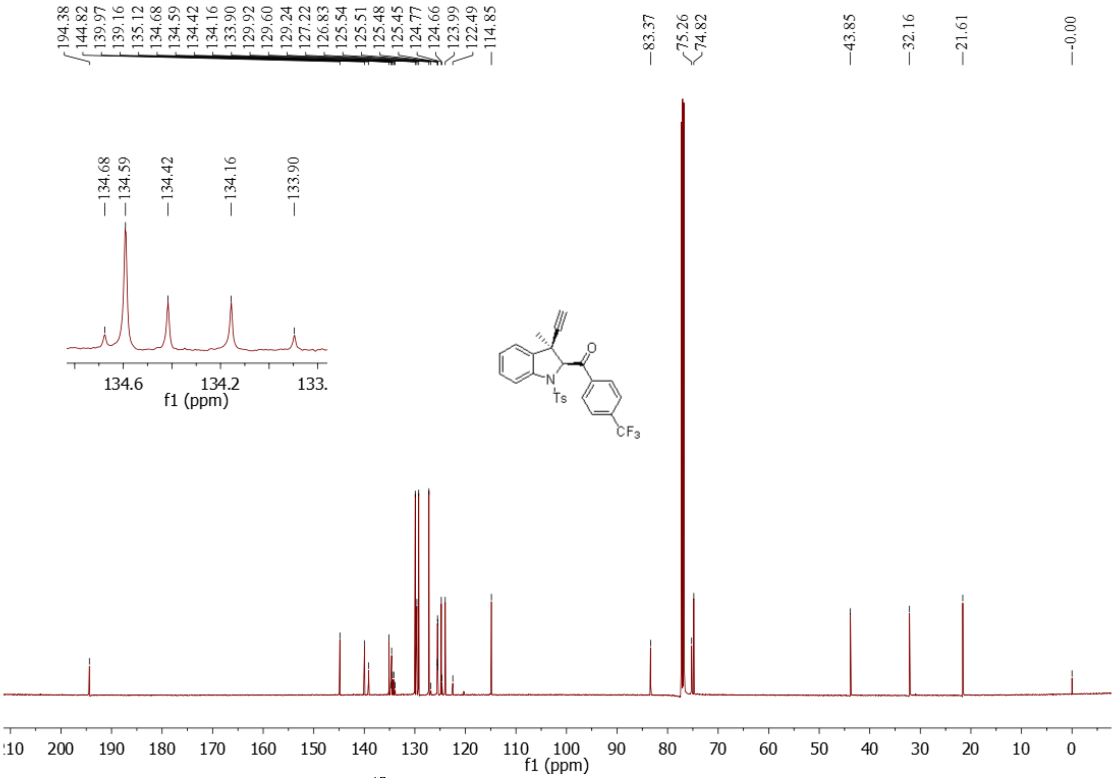

Figure S80. ^{13}C NMR spectrum of **5af**, related to **Scheme 4**.

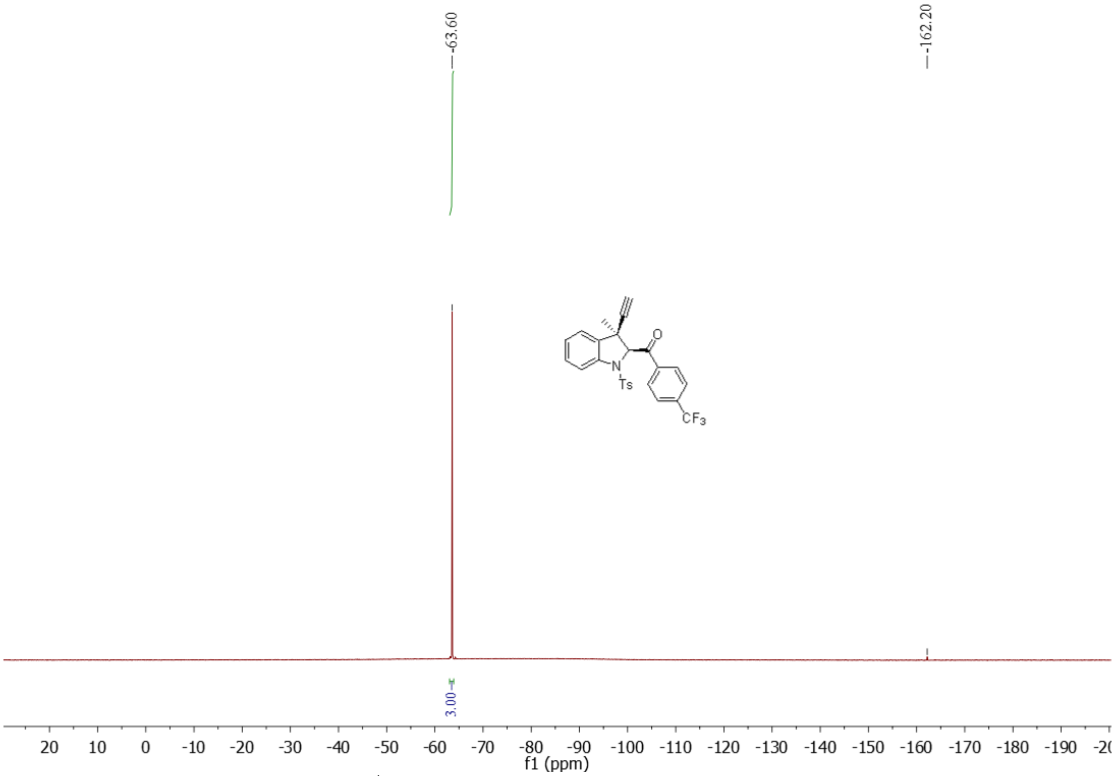

Figure S81. ^{1}H NMR spectrum of **5af**, related to **Scheme 4**.

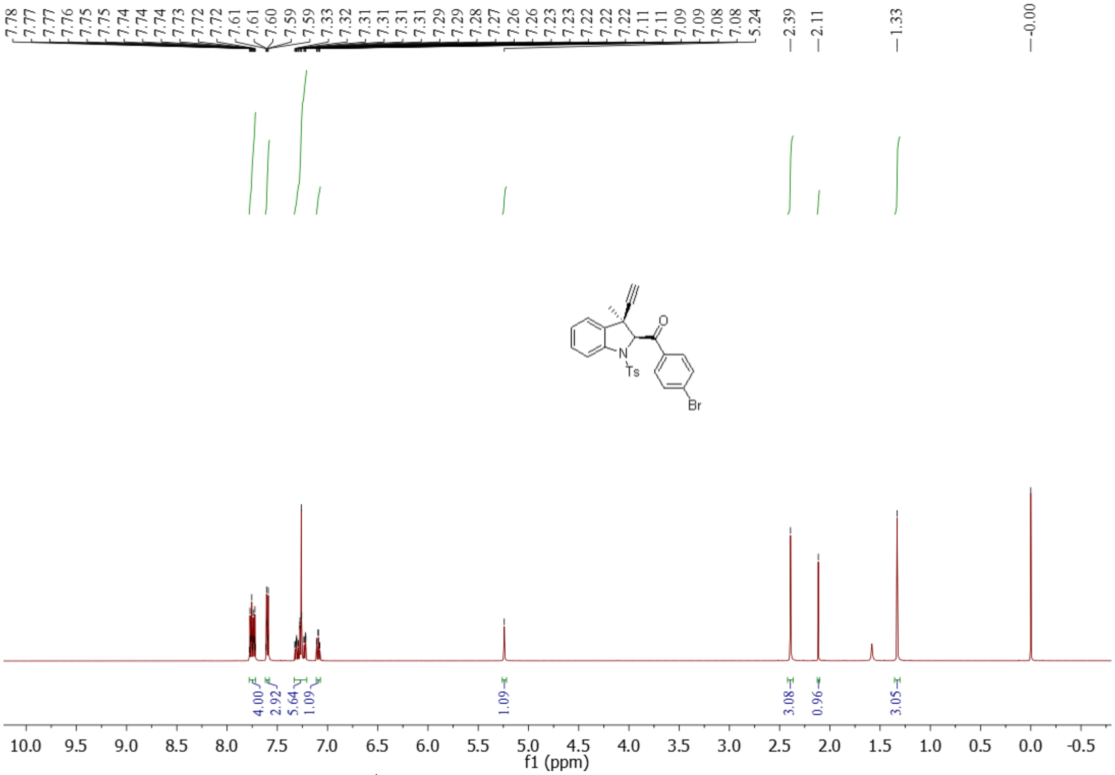

Figure S82. ^{1}H NMR spectrum of **5ag**, related to **Scheme 4**.

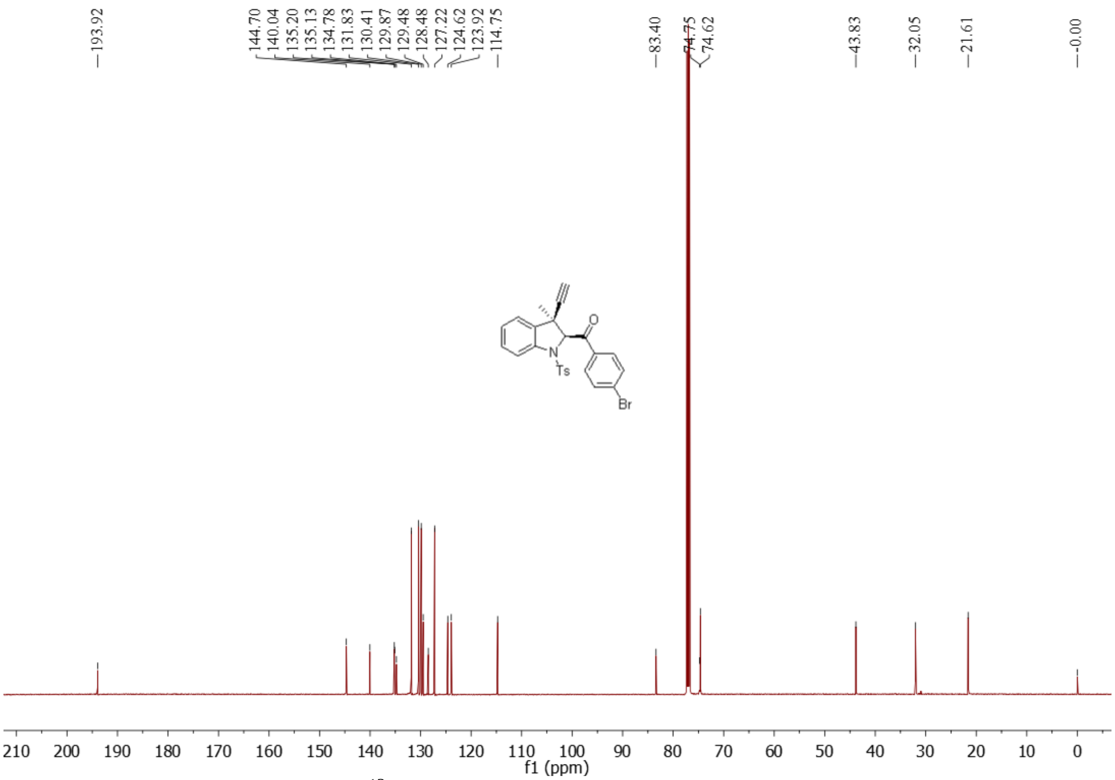

Figure S83. ^{13}C NMR spectrum of **5ag**, related to **Scheme 4**.

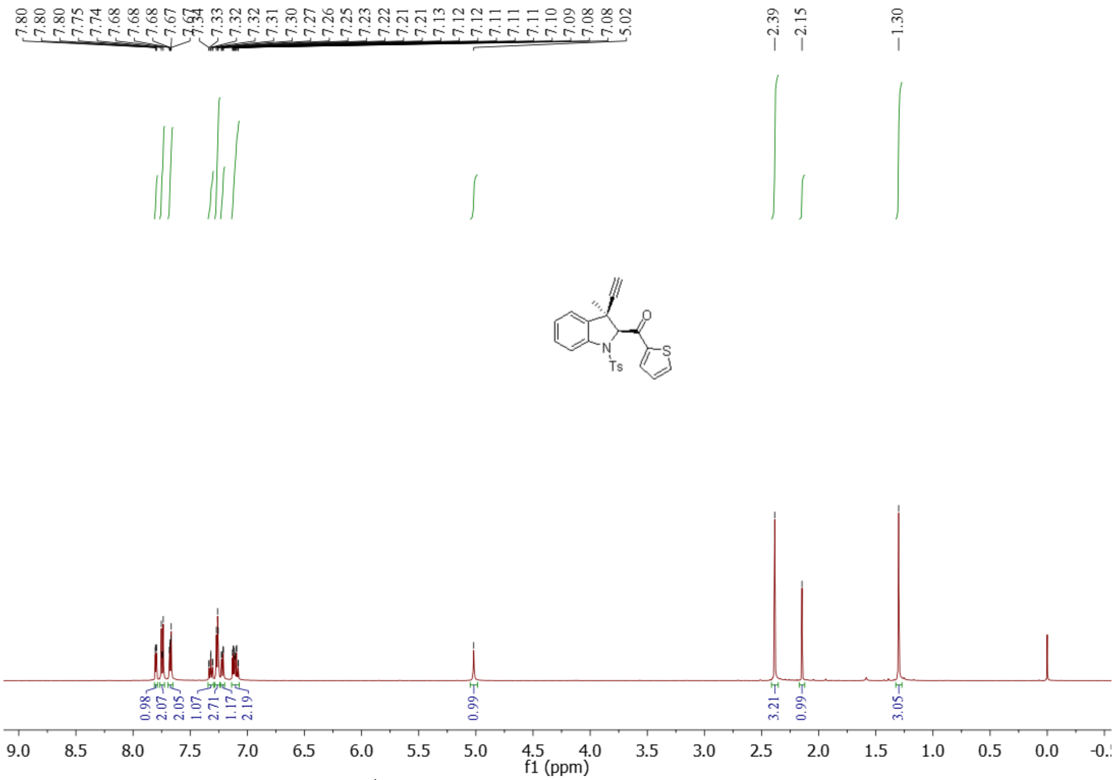

Figure S84. ^{1}H NMR spectrum of **5ah**, related to **Scheme 4**.

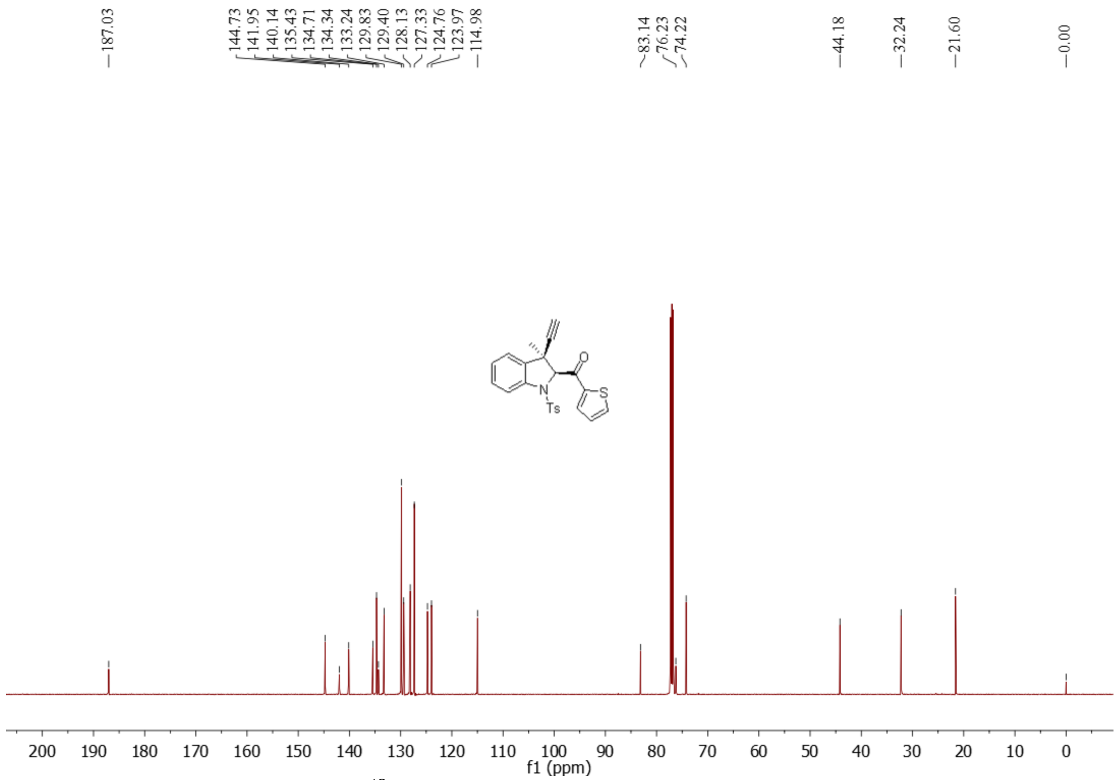

Figure S85. ^{13}C NMR spectrum of **5ah**, related to **Scheme 4**.

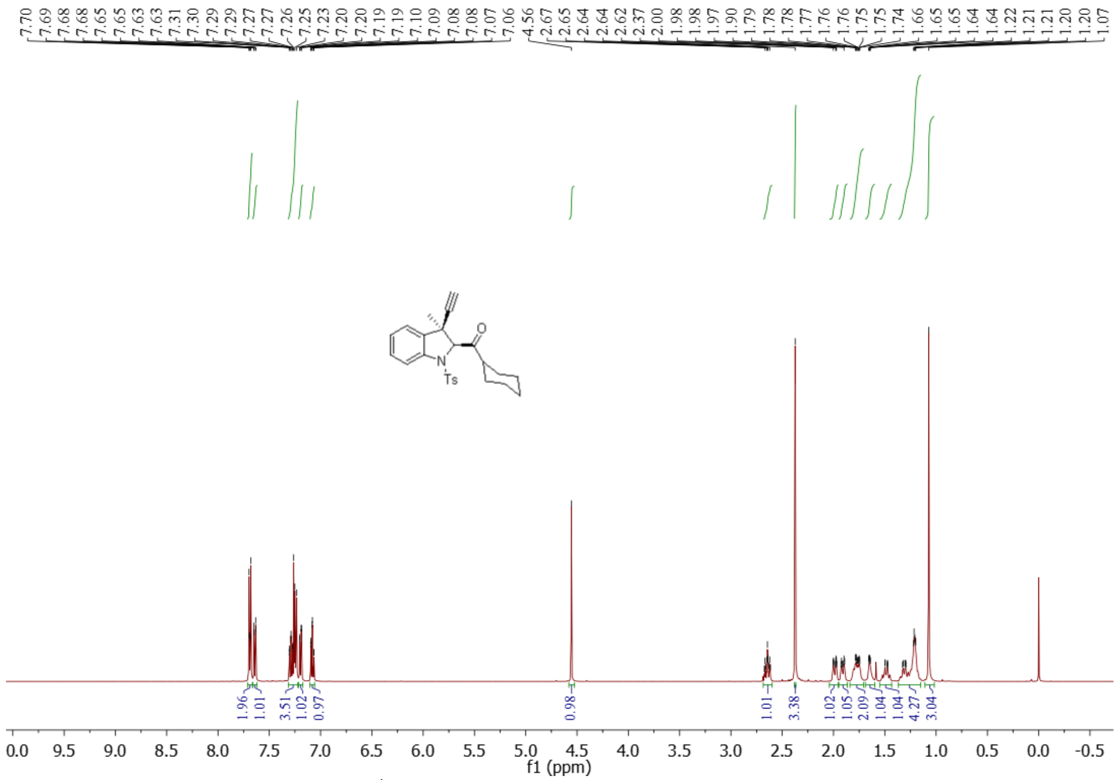

Figure S86. ^{1}H NMR spectrum of **5ai**, related to **Scheme 4**.

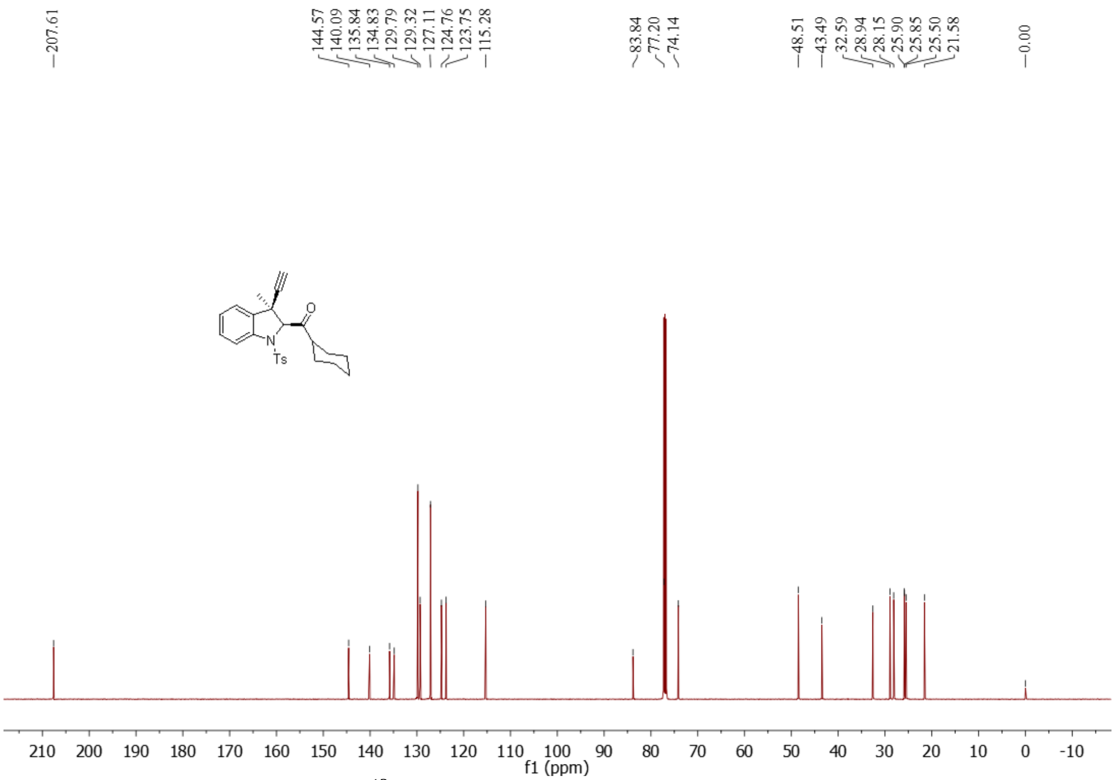

Figure S87. ^{13}C NMR spectrum of **5ai**, related to **Scheme 4**.

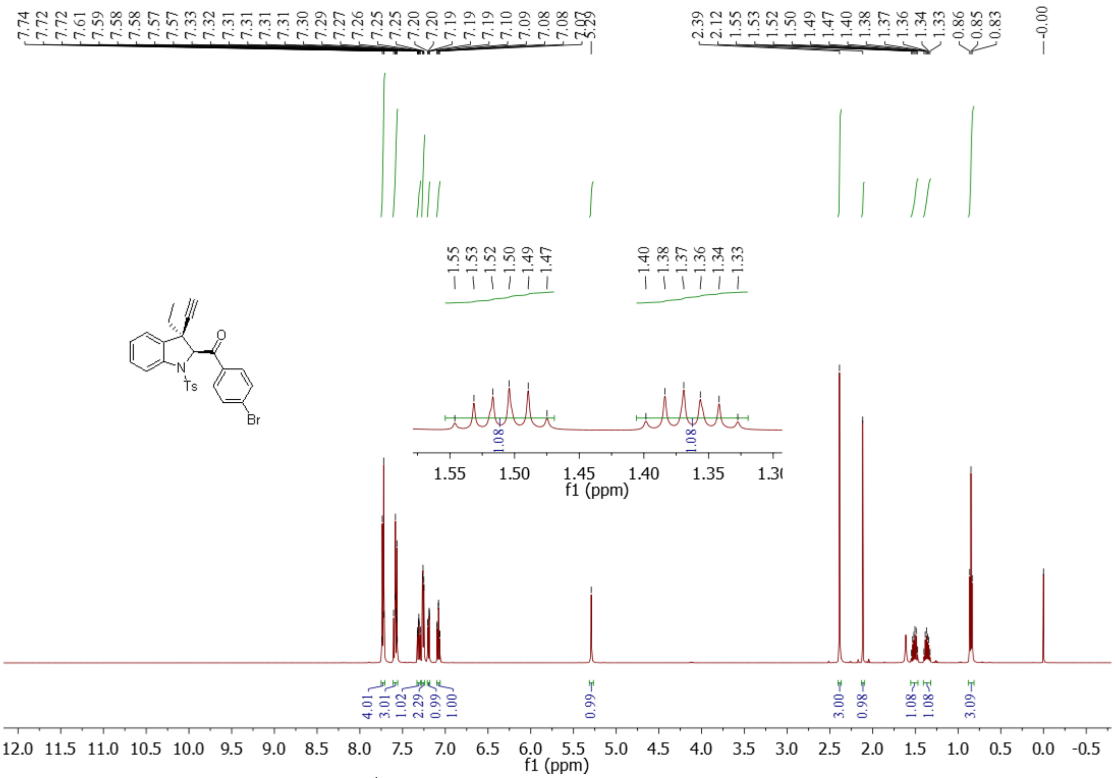

Figure S88. ^{1}H NMR spectrum of **5gg**, related to **Scheme 4**.

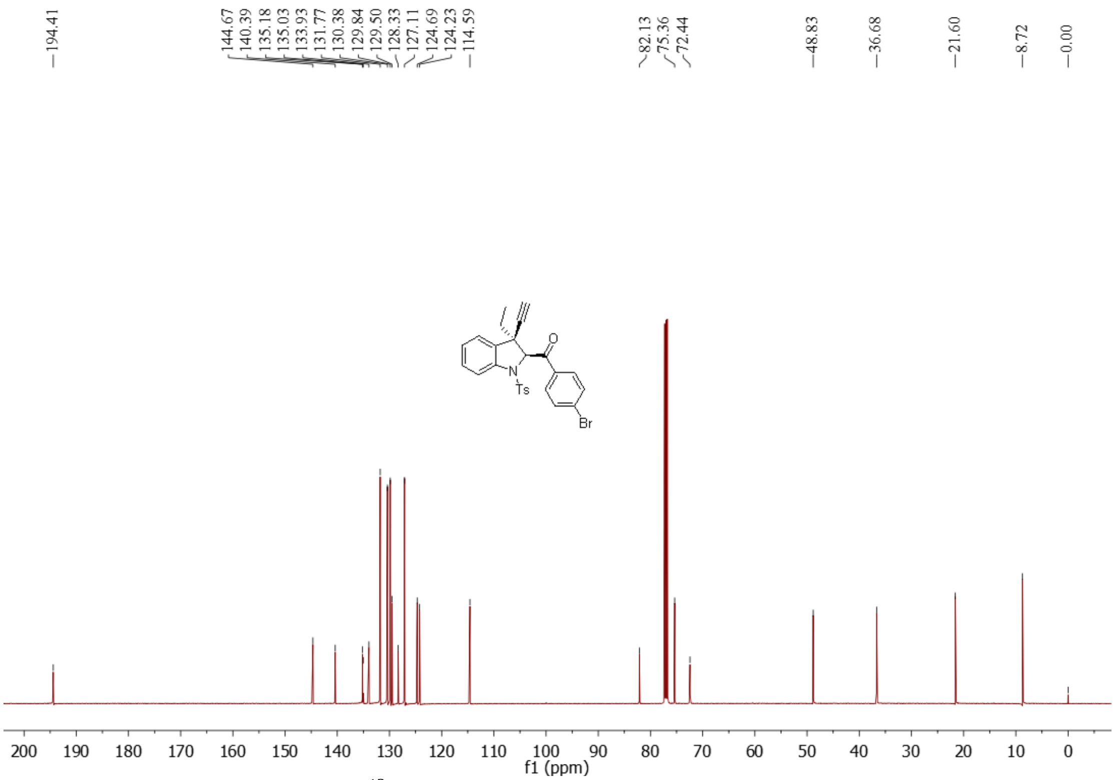

Figure S89. ^{13}C NMR spectrum of **5gg**, related to **Scheme 4**.

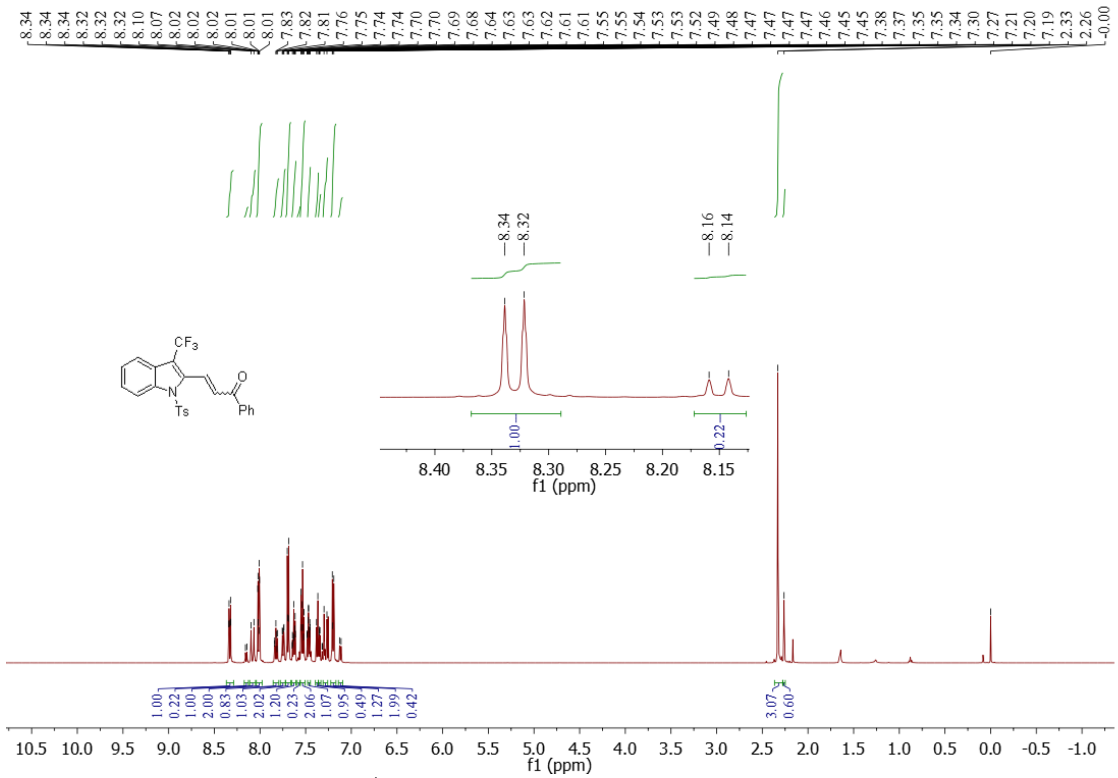

Figure S90. ^{1}H NMR spectrum of **6aa**, related to **Scheme 6**.

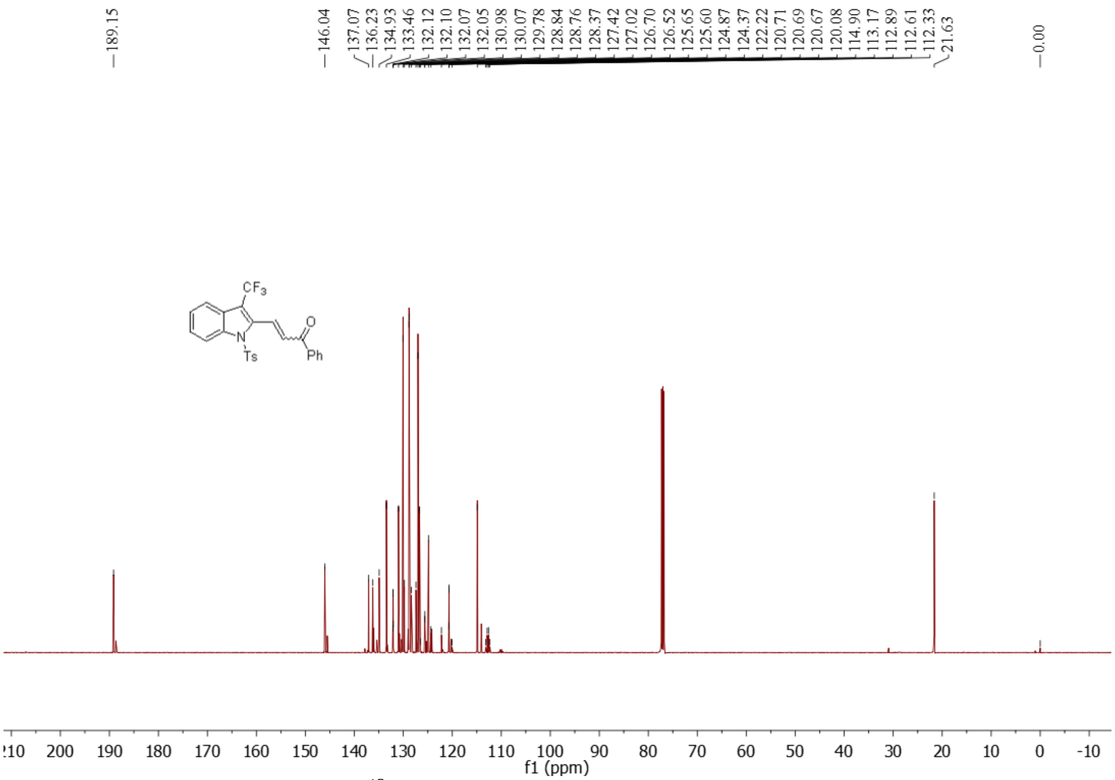

Figure S91. ^{13}C NMR spectrum of **6aa**, related to **Scheme 6**.

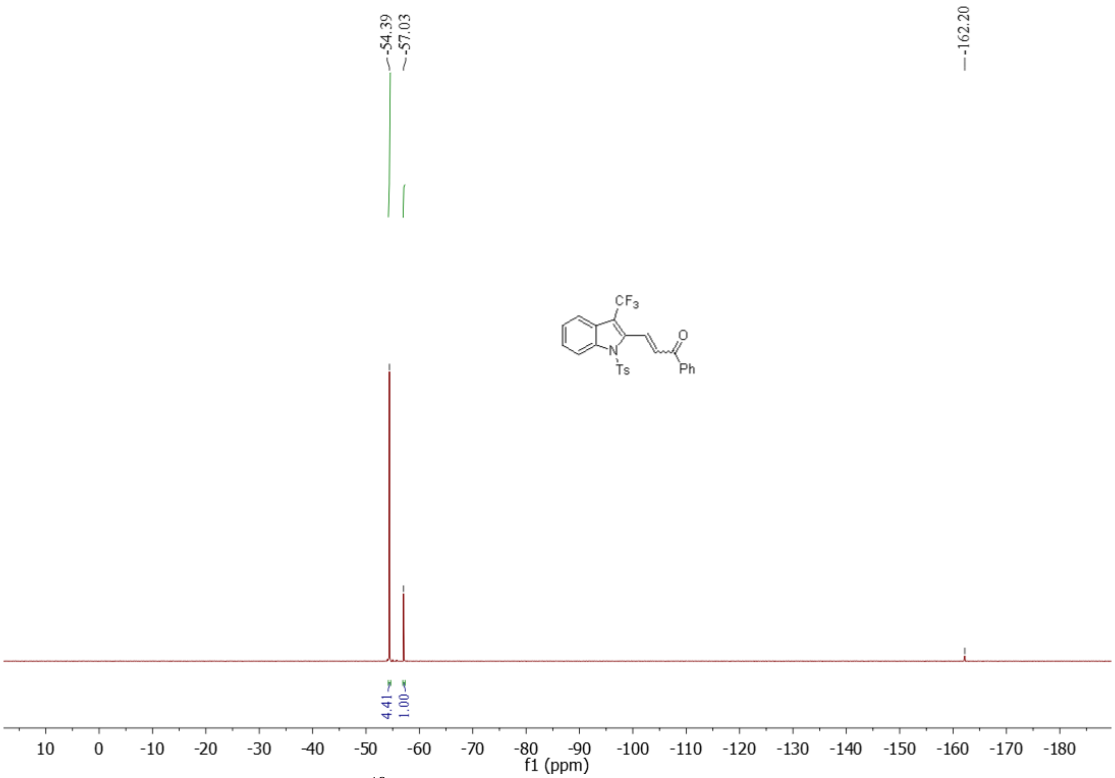

Figure S92. ^{19}F NMR spectrum of **6aa**, related to **Scheme 6**.

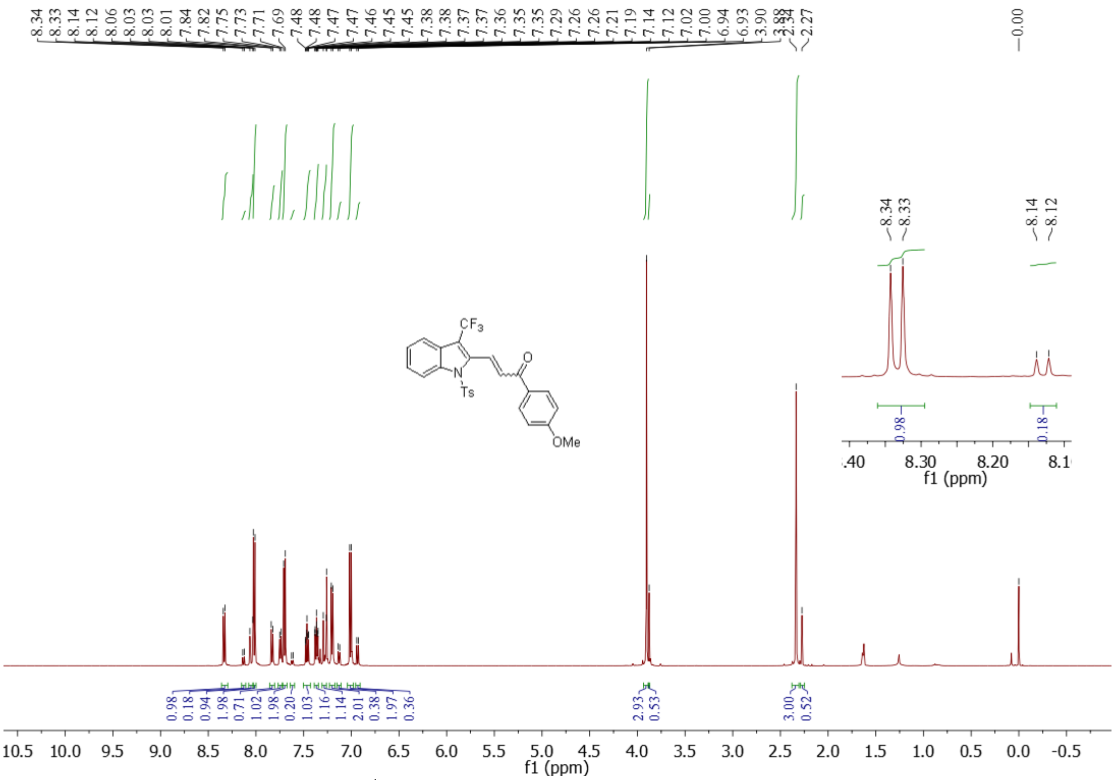

Figure S93. ^{1}H NMR spectrum of **6ab**, related to **Scheme 6**.

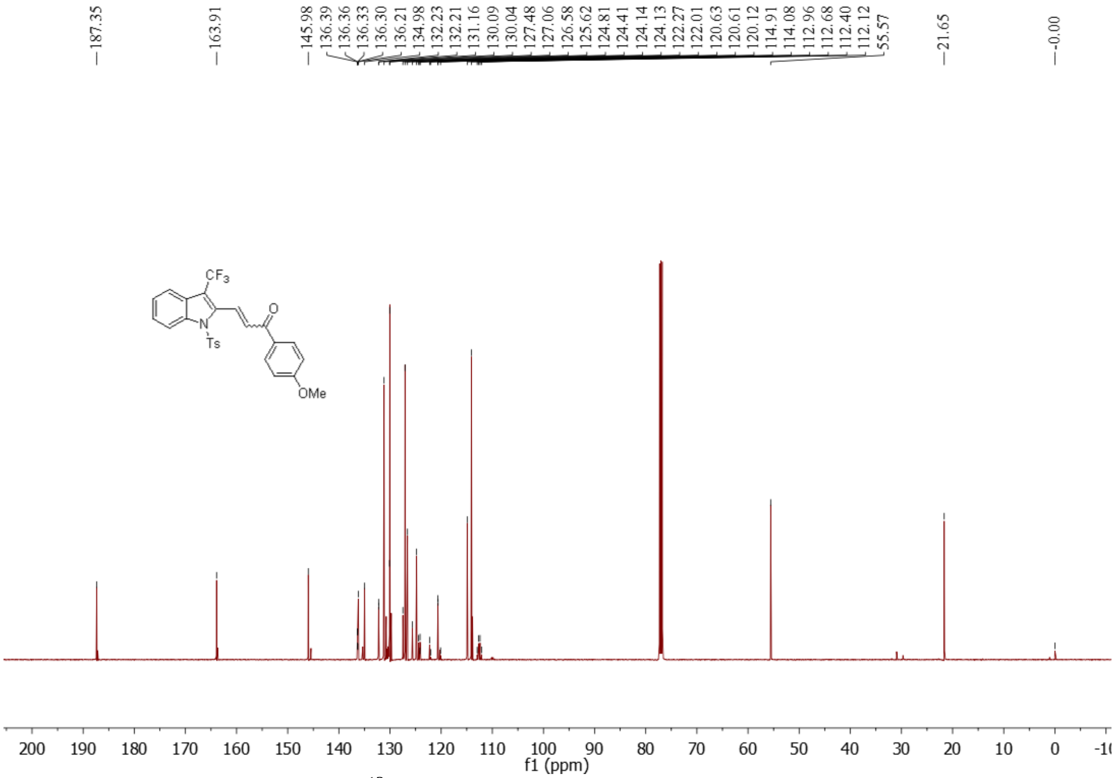

Figure S94. ^{13}C NMR spectrum of **6ab**, related to **Scheme 6**.

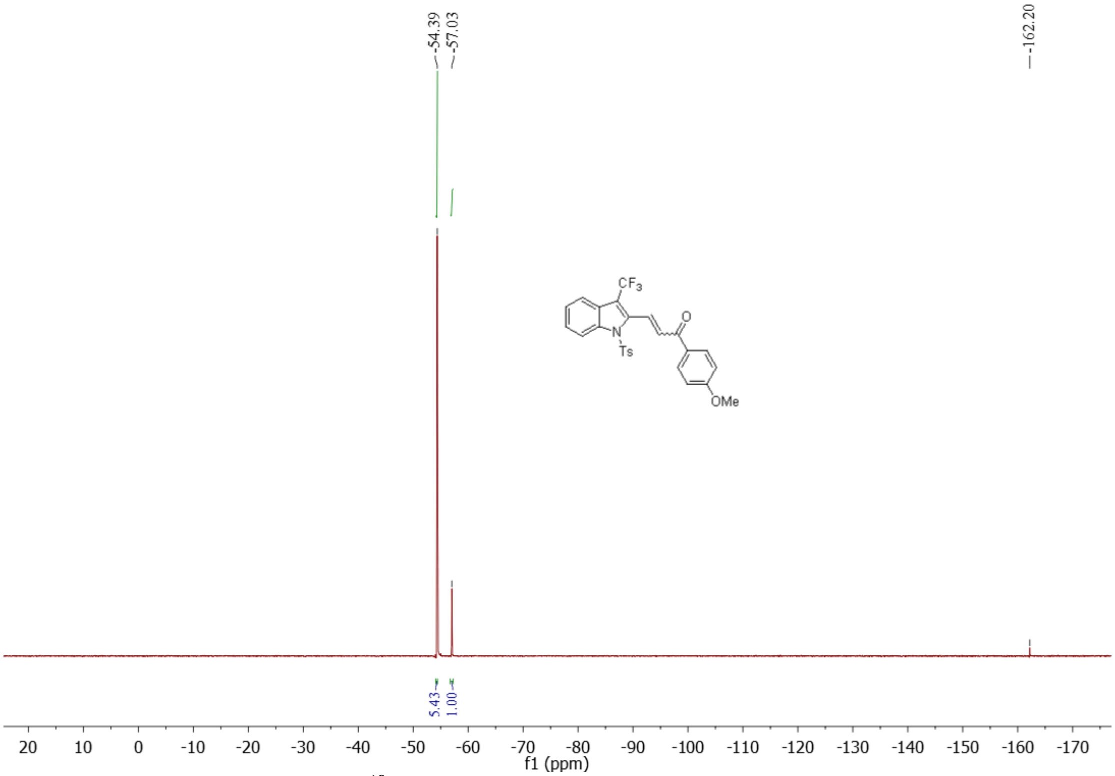

Figure S95. ^{19}F NMR spectrum of **6ab**, related to **Scheme 6**.

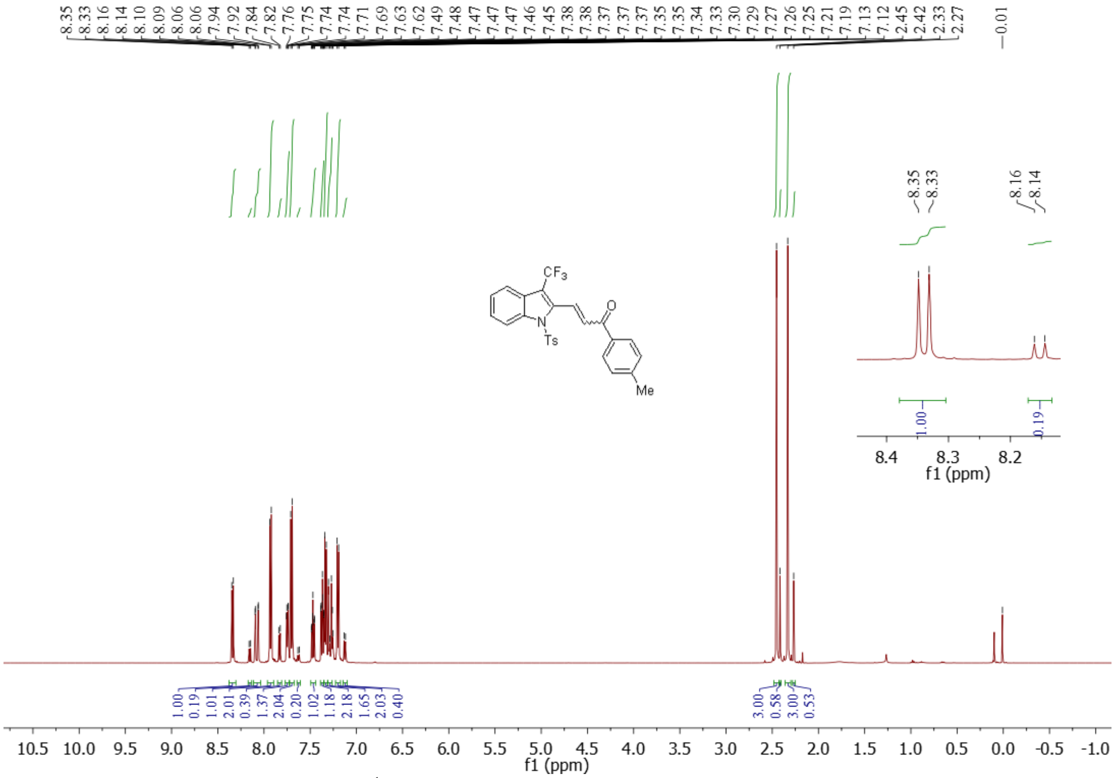

Figure S96. ^{1}H NMR spectrum of **6ac**, related to **Scheme 6**.

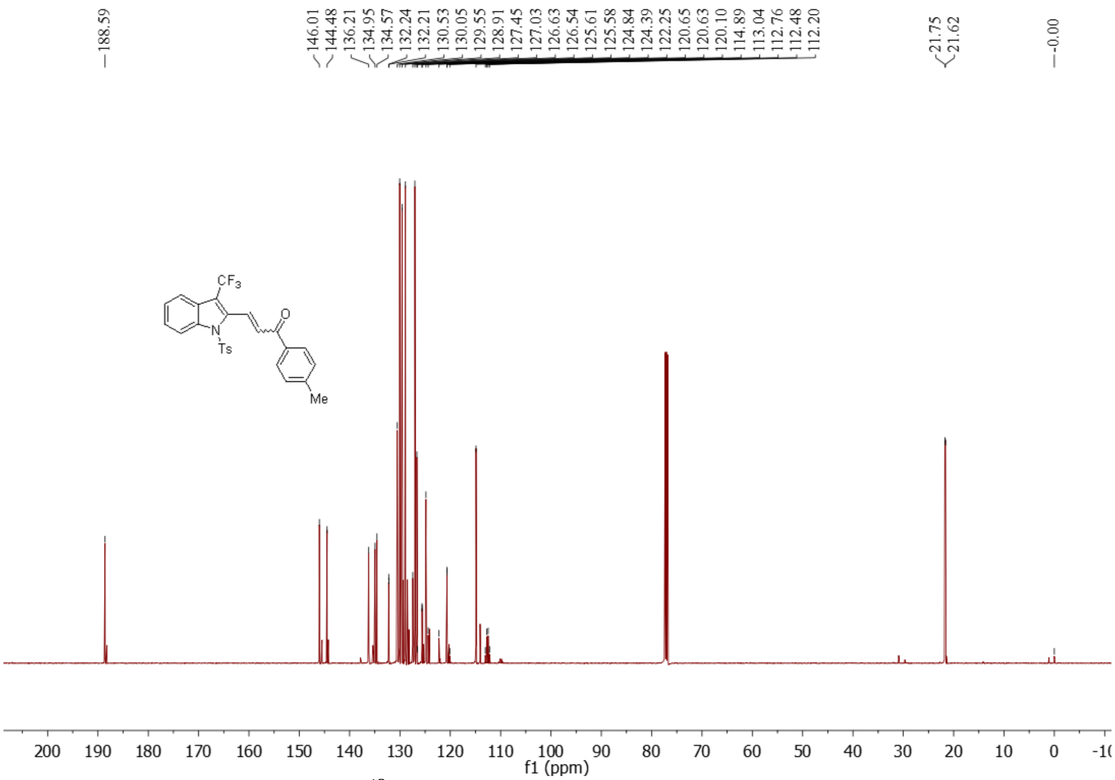

Figure S97. ^{13}C NMR spectrum of **6ac**, related to **Scheme 6**.

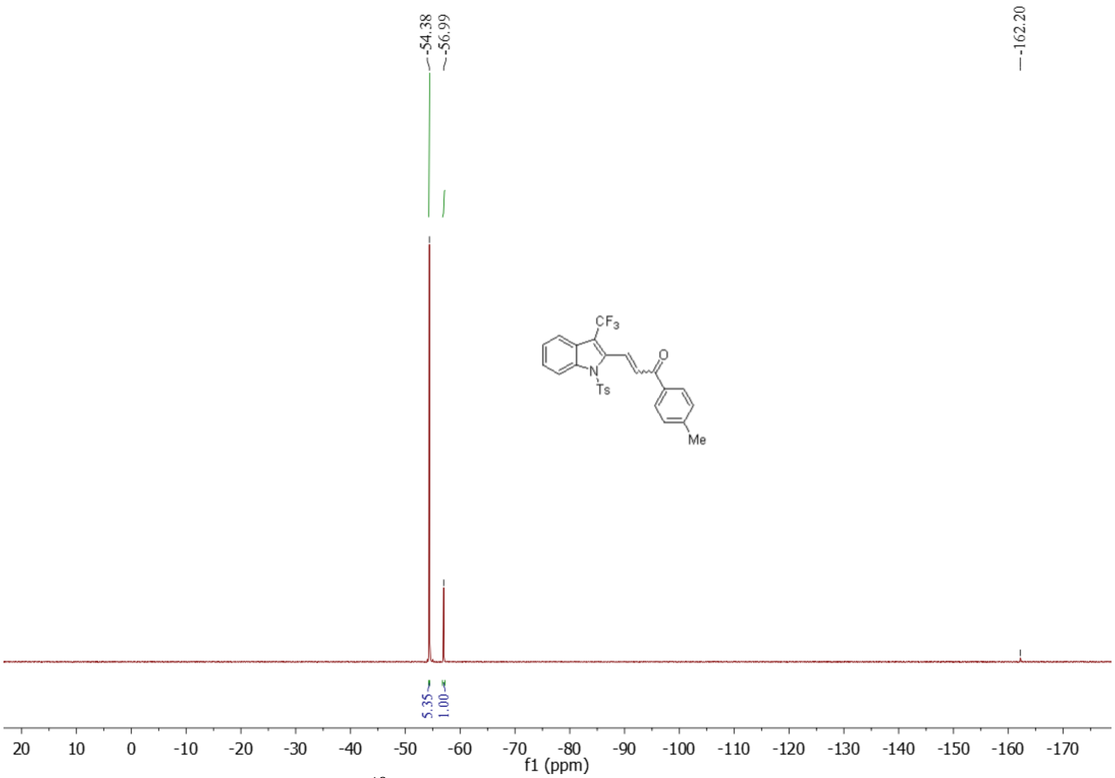

Figure S98. ^{19}F NMR spectrum of **6ac**, related to **Scheme 6**.

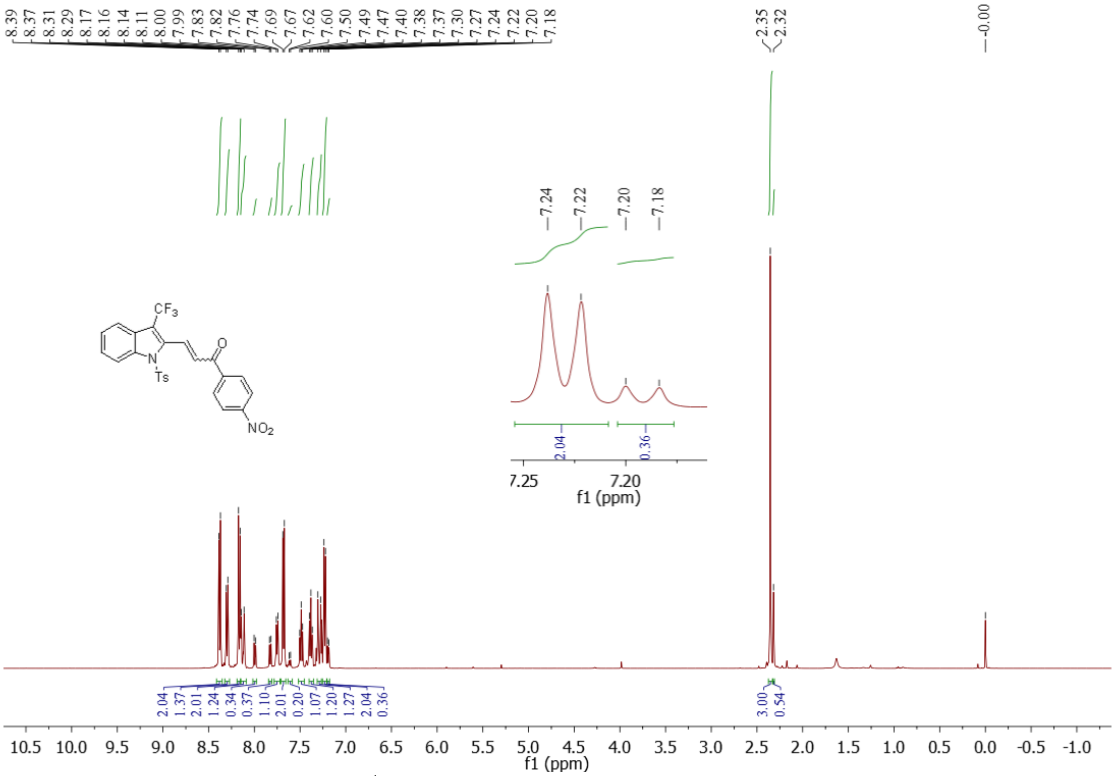

Figure S99. ^{1}H NMR spectrum of **6ad**, related to **Scheme 6**.

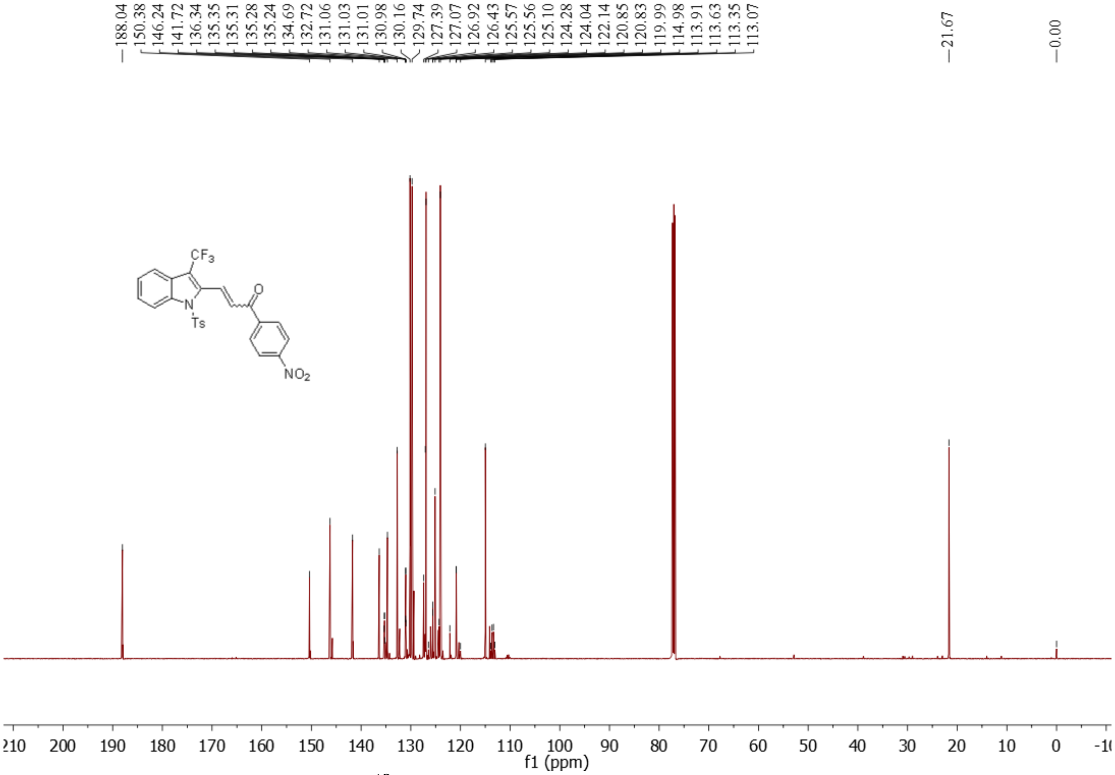

Figure S100. ^{13}C NMR spectrum of **6ad**, related to **Scheme 6**.

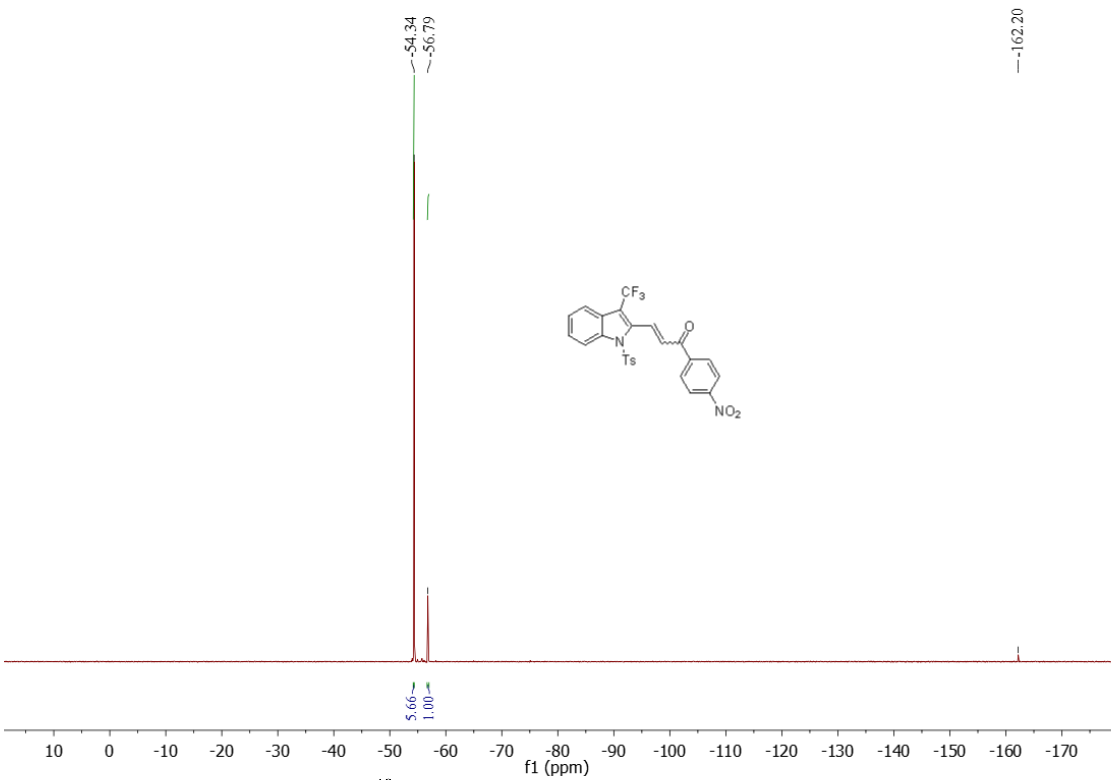

Figure S101. ^{19}F NMR spectrum of **6ad**, related to **Scheme 6**.

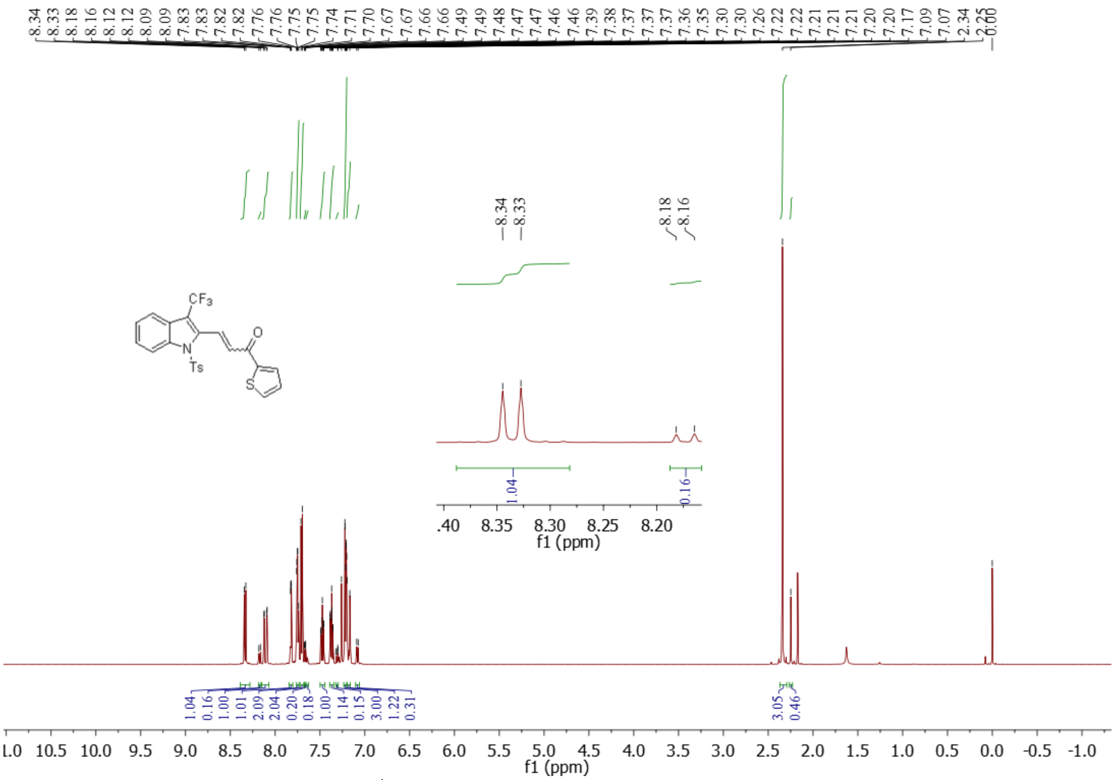

Figure S102. ^{1}H NMR spectrum of **6ah**, related to **Scheme 6**.

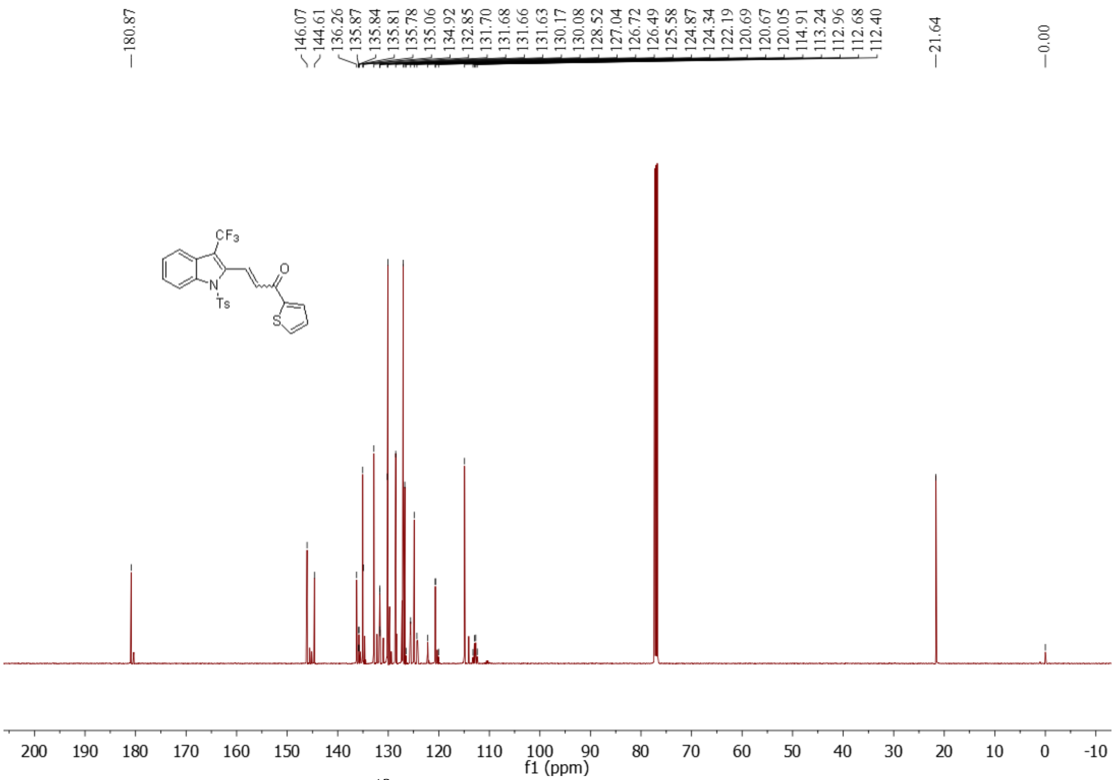

Figure S103. ^{13}C NMR spectrum of **6ah**, related to **Scheme 6**.

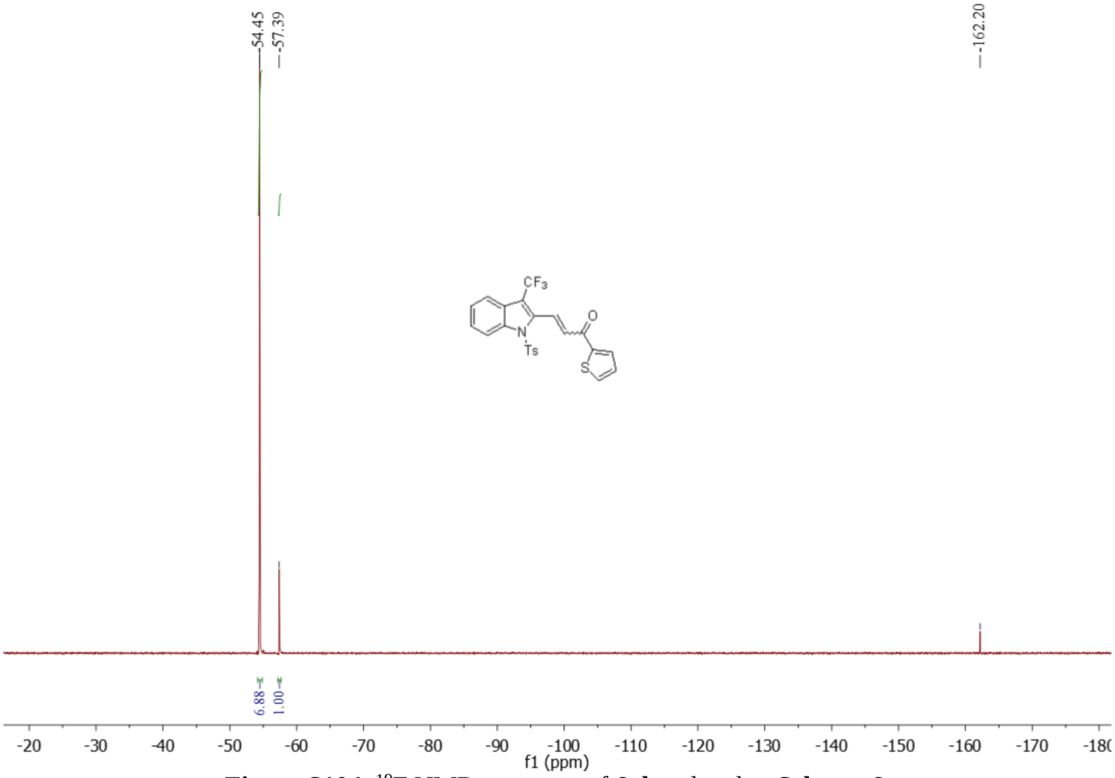

Figure S104. ^{19}F NMR spectrum of **6ah**, related to **Scheme 6**.

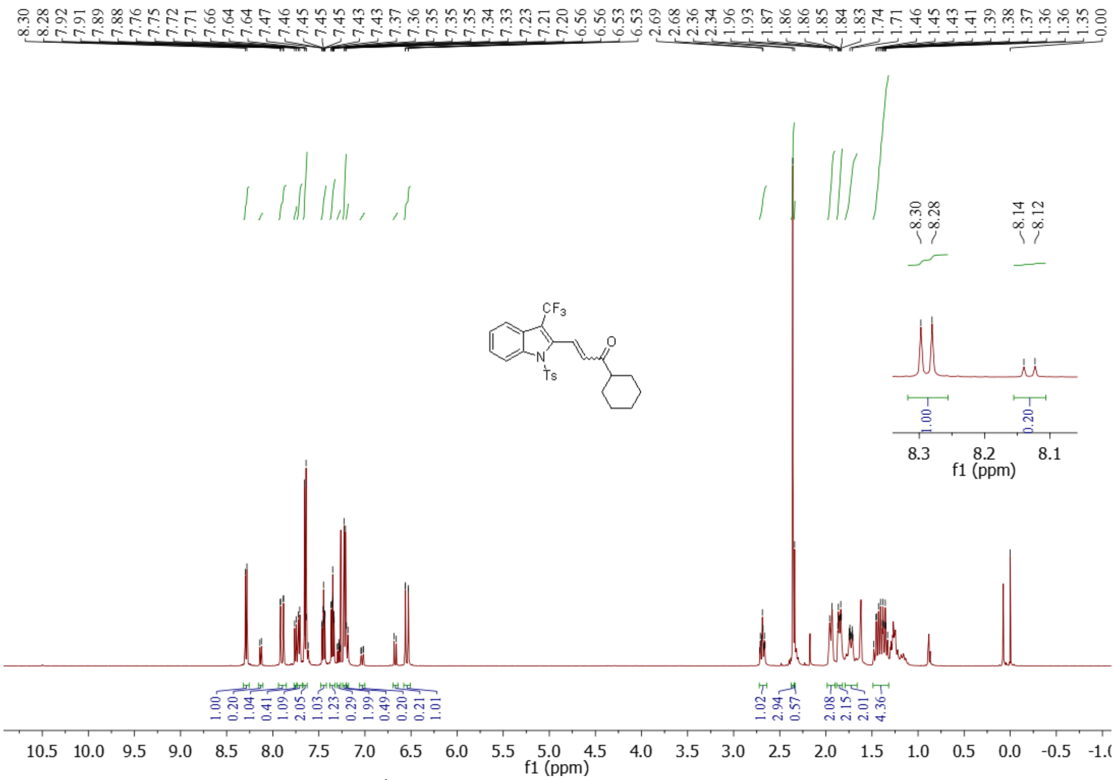

Figure S105. ^{1}H NMR spectrum of **6ai**, related to **Scheme 6**.

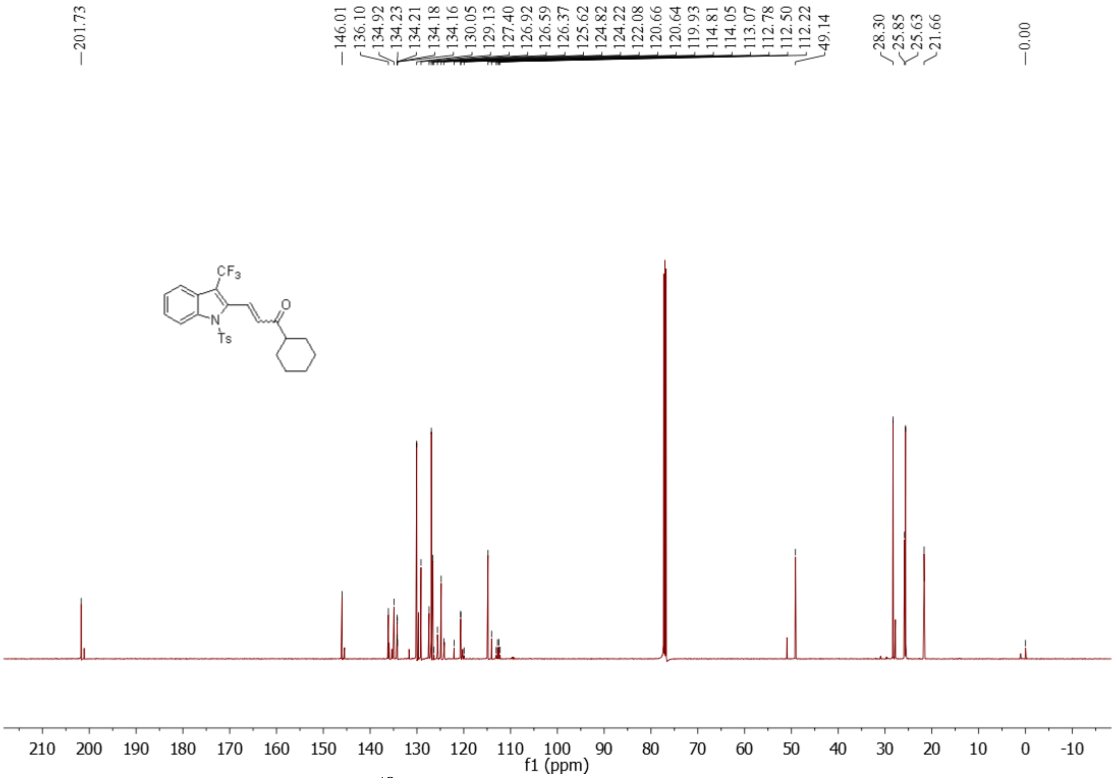

Figure S106. ^{13}C NMR spectrum of **6ai**, related to **Scheme 6**.

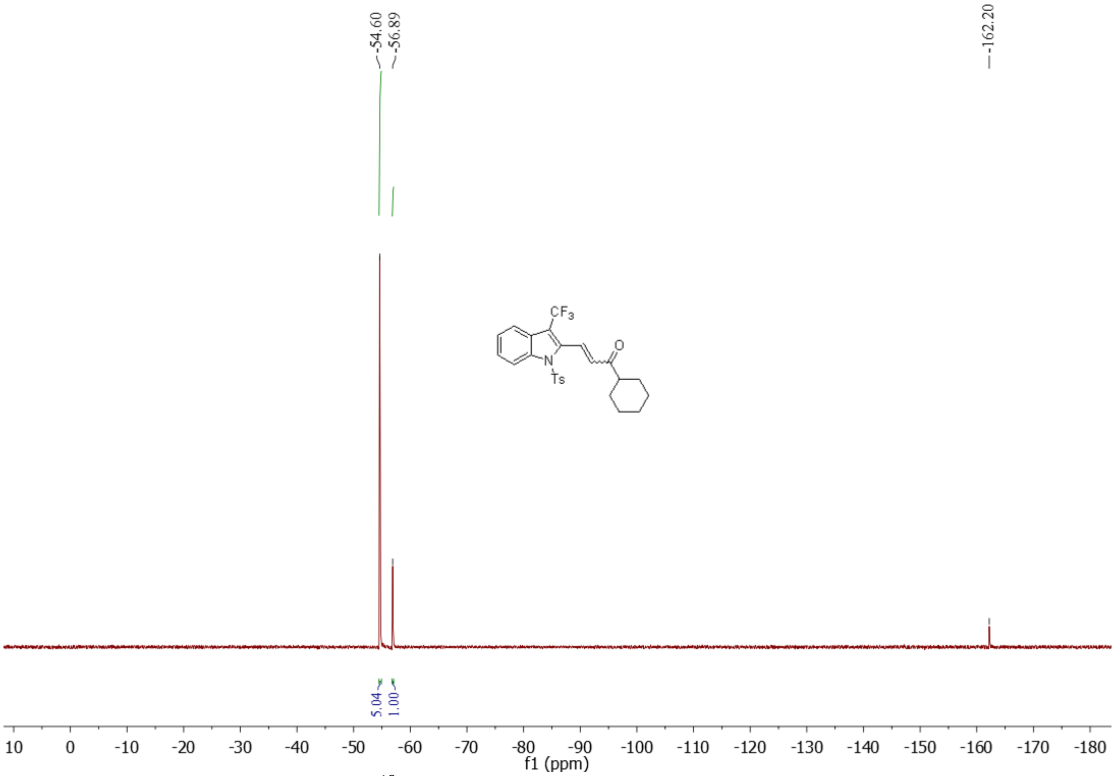

Figure S107. ^{19}F NMR spectrum of **6ai**, related to **Scheme 6**.

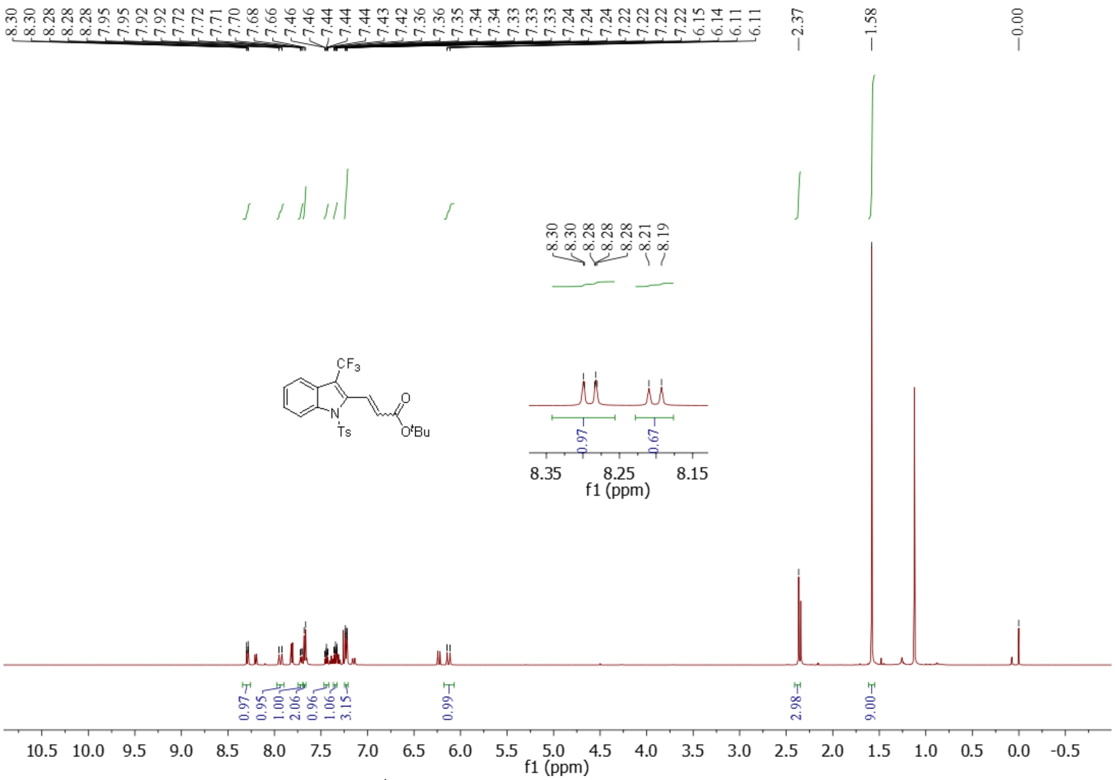

Figure S108. ^{1}H NMR spectrum of **6aj**, related to **Scheme 6**.

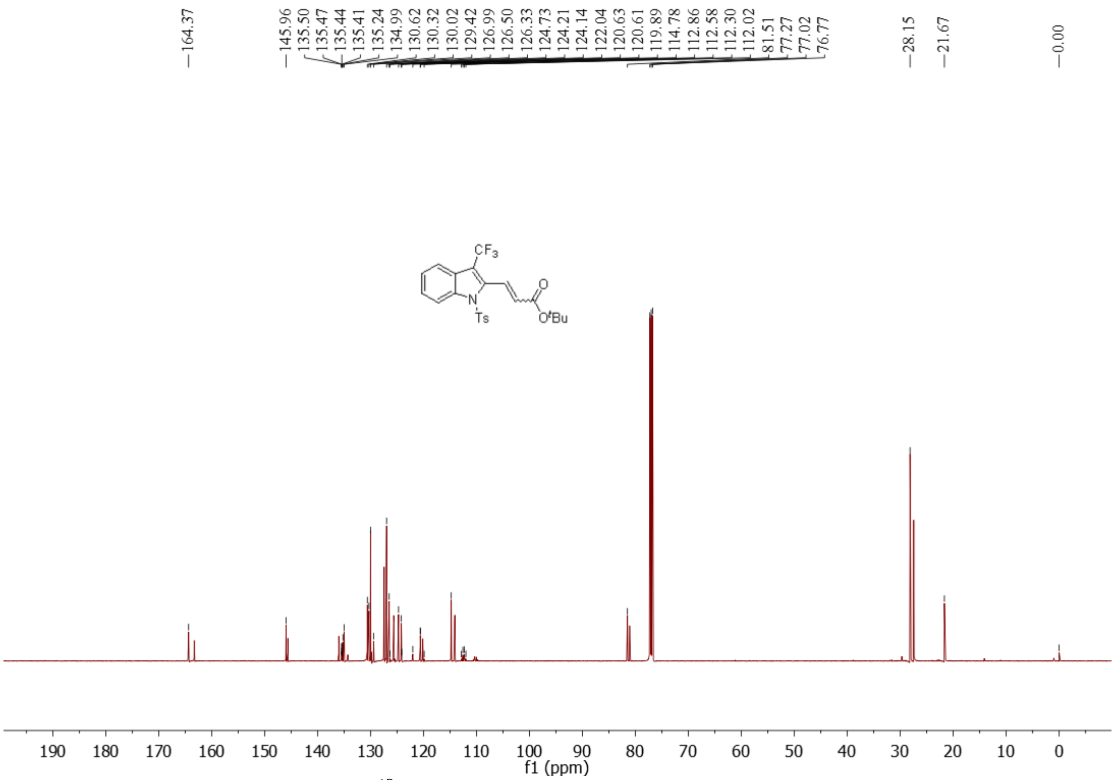

Figure S109. ^{13}C NMR spectrum of **6aj**, related to **Scheme 6**.

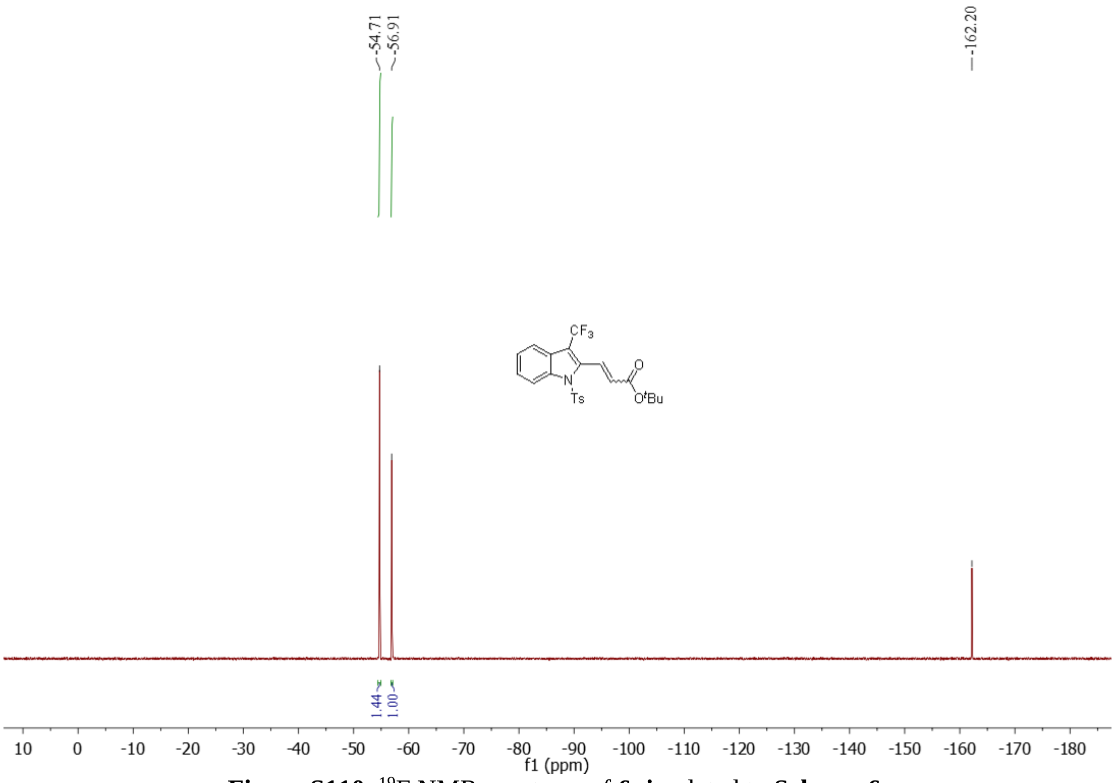

Figure S110. ^{19}F NMR spectrum of **6aj**, related to **Scheme 6**.

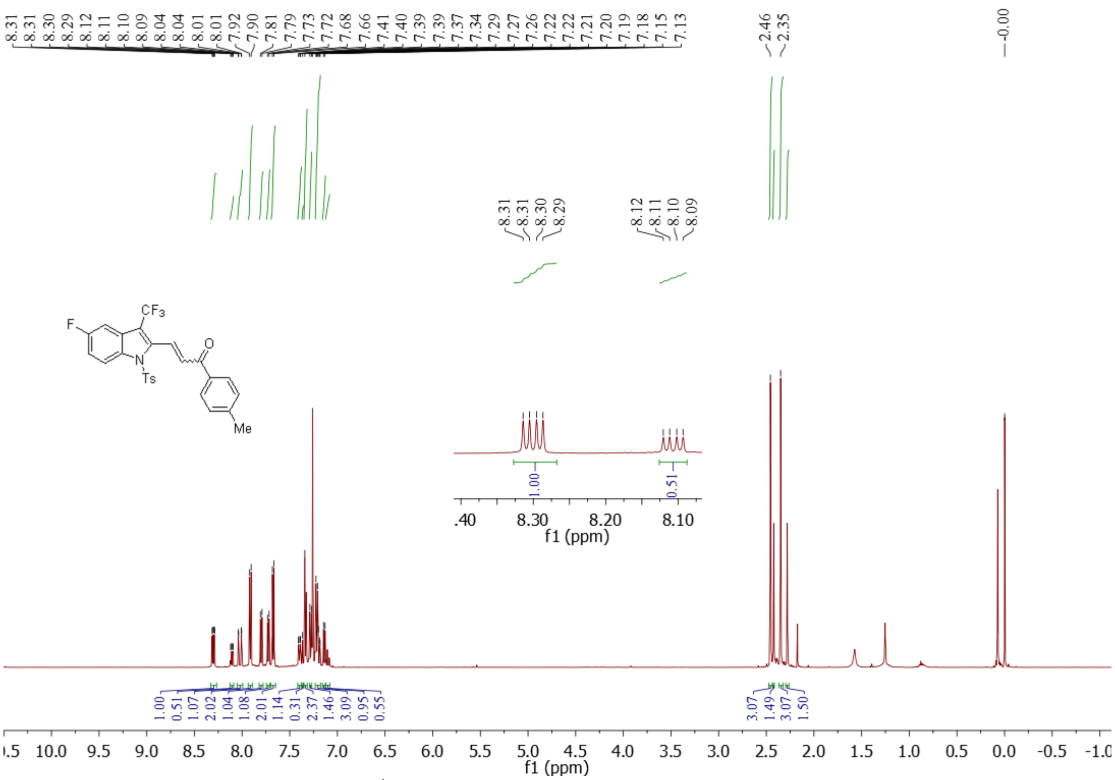

Figure S111. ^{1}H NMR spectrum of **6bc**, related to **Scheme 6**.

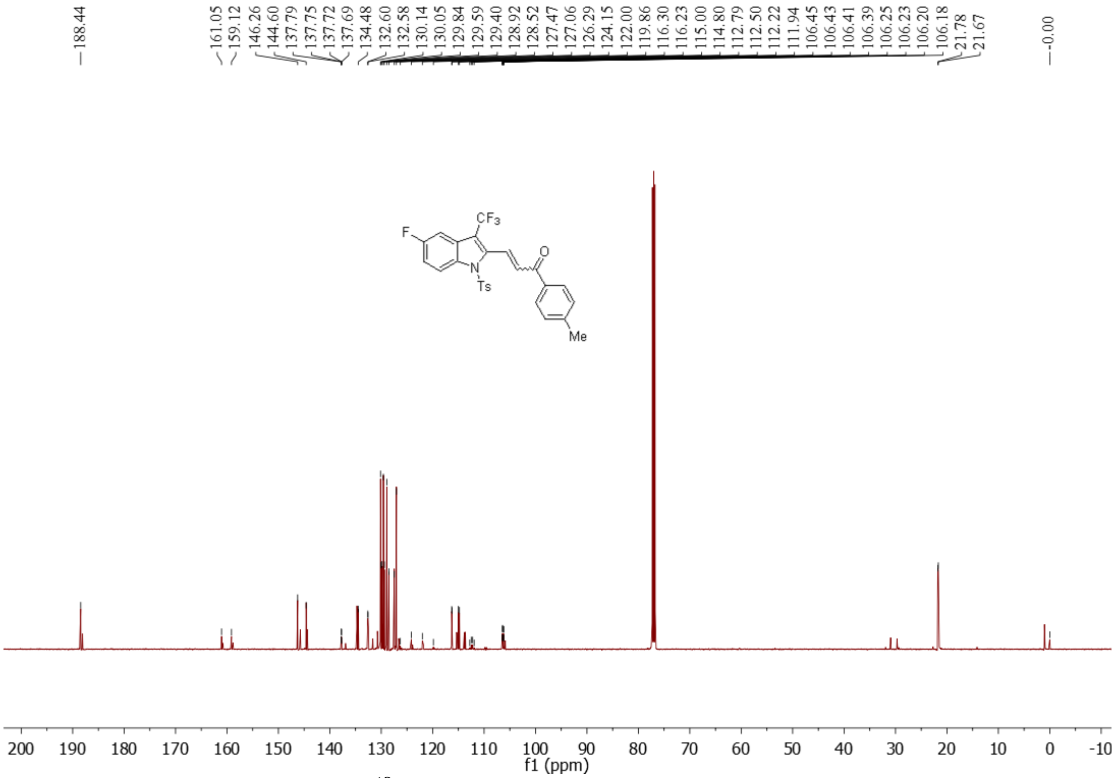

Figure S112. ^{13}C NMR spectrum of **6bc**, related to **Scheme 6**.

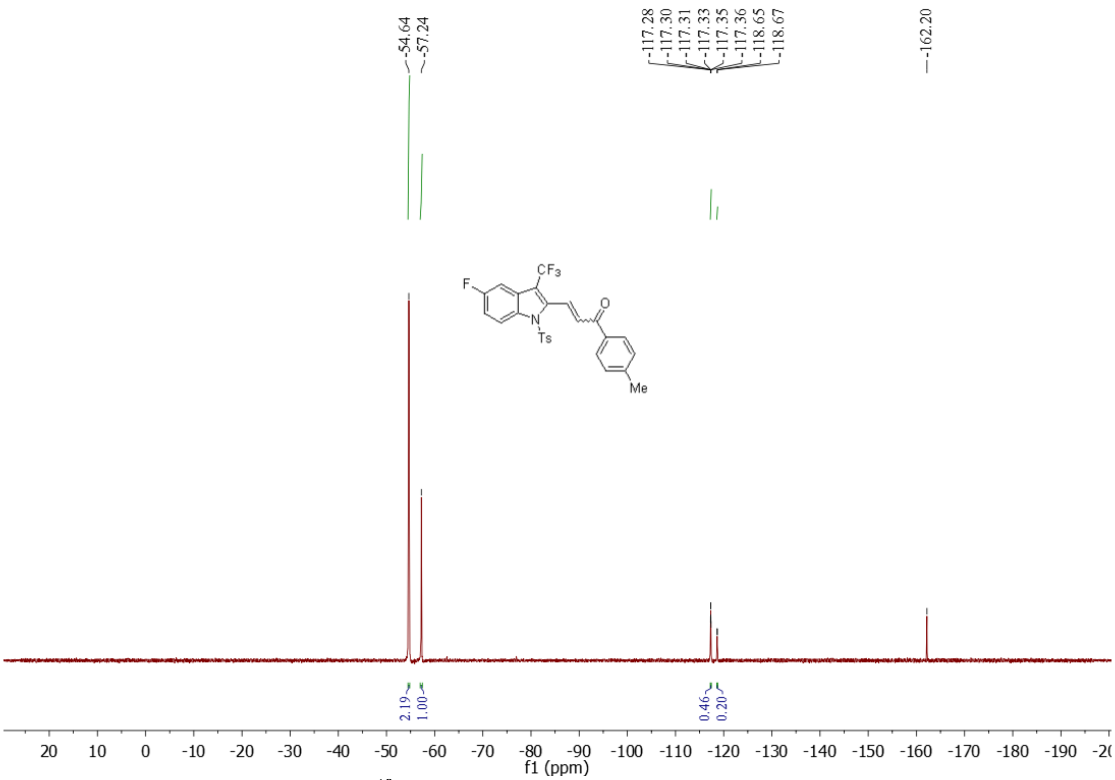

Figure S113. ^{19}F NMR spectrum of **6bc**, related to **Scheme 6**.

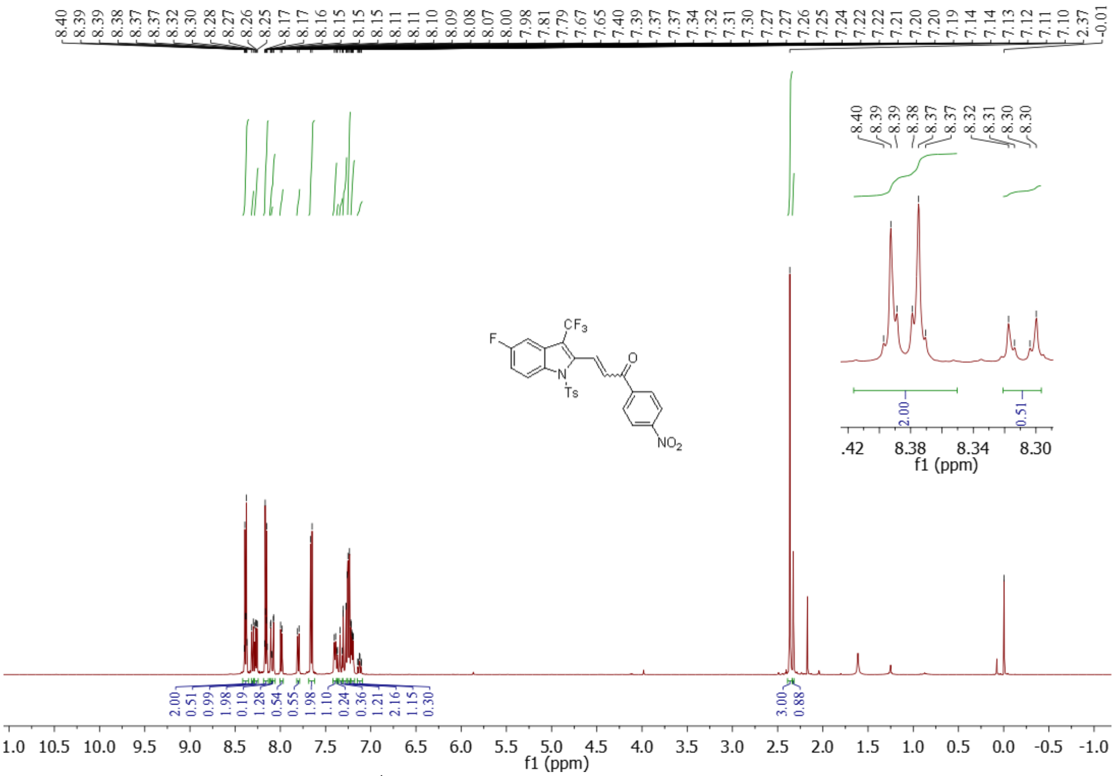

Figure S114. ^{1}H NMR spectrum of **6bd**, related to **Scheme 6**.

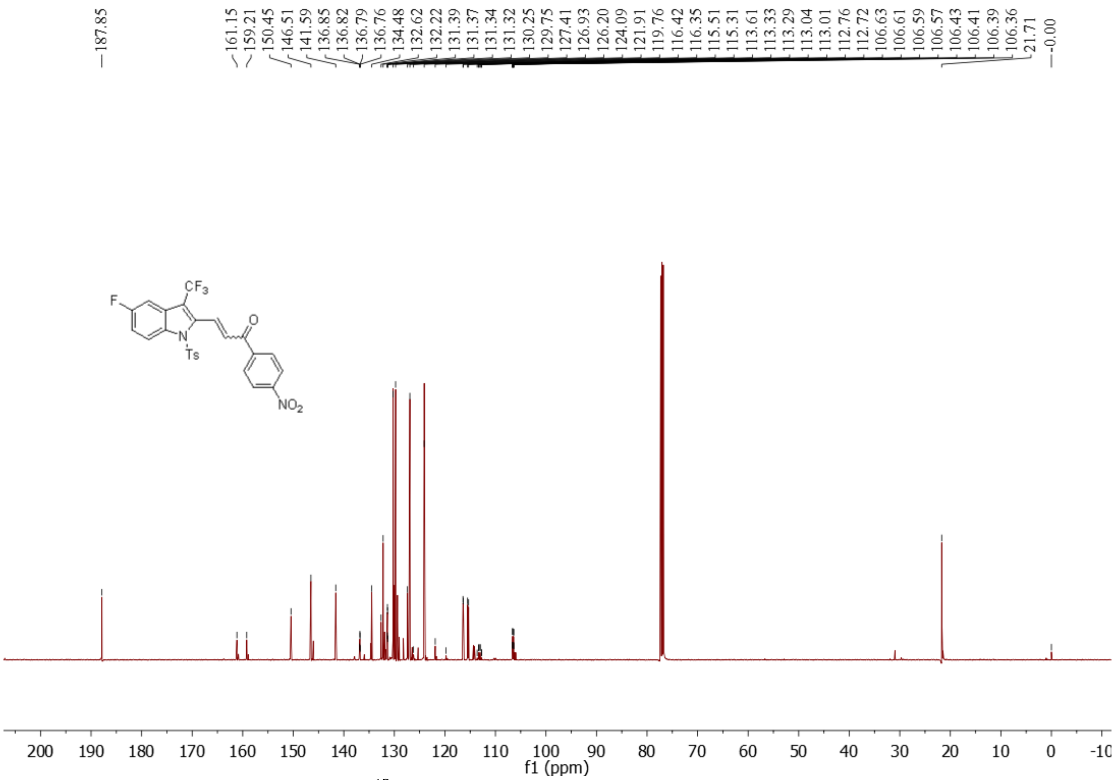

Figure S115. ^{13}C NMR spectrum of **6bd**, related to **Scheme 6**.

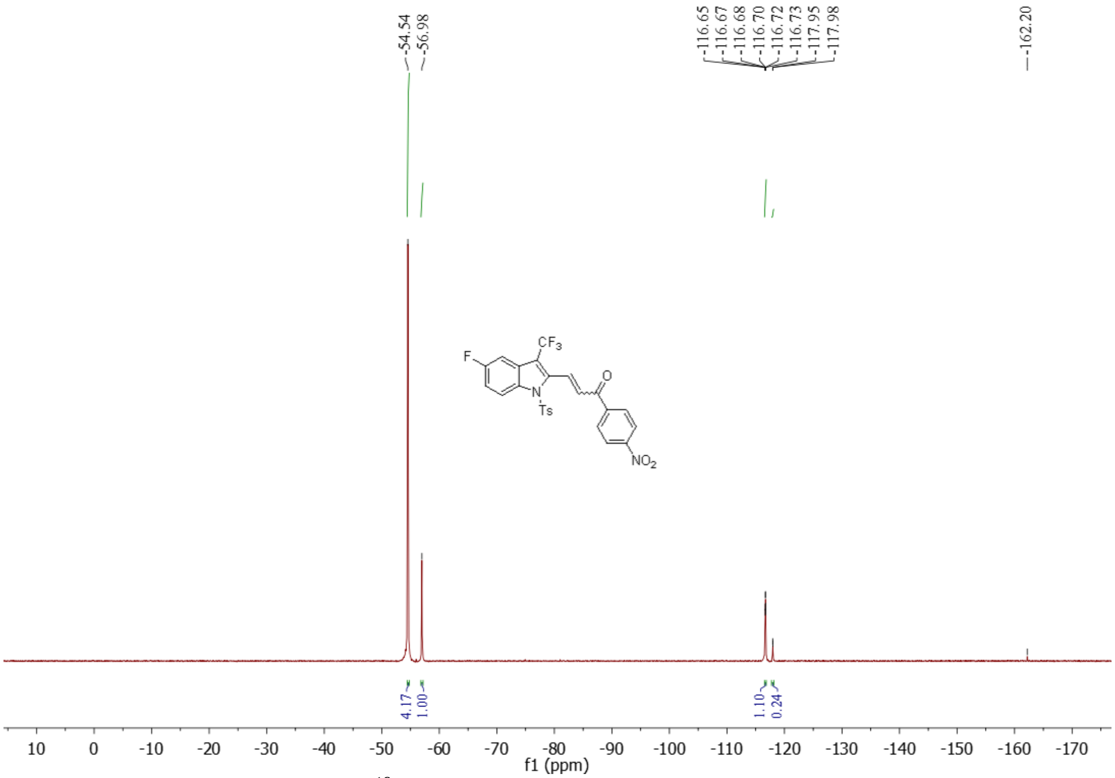

Figure S116. ^{19}F NMR spectrum of **6bd**, related to **Scheme 6**.

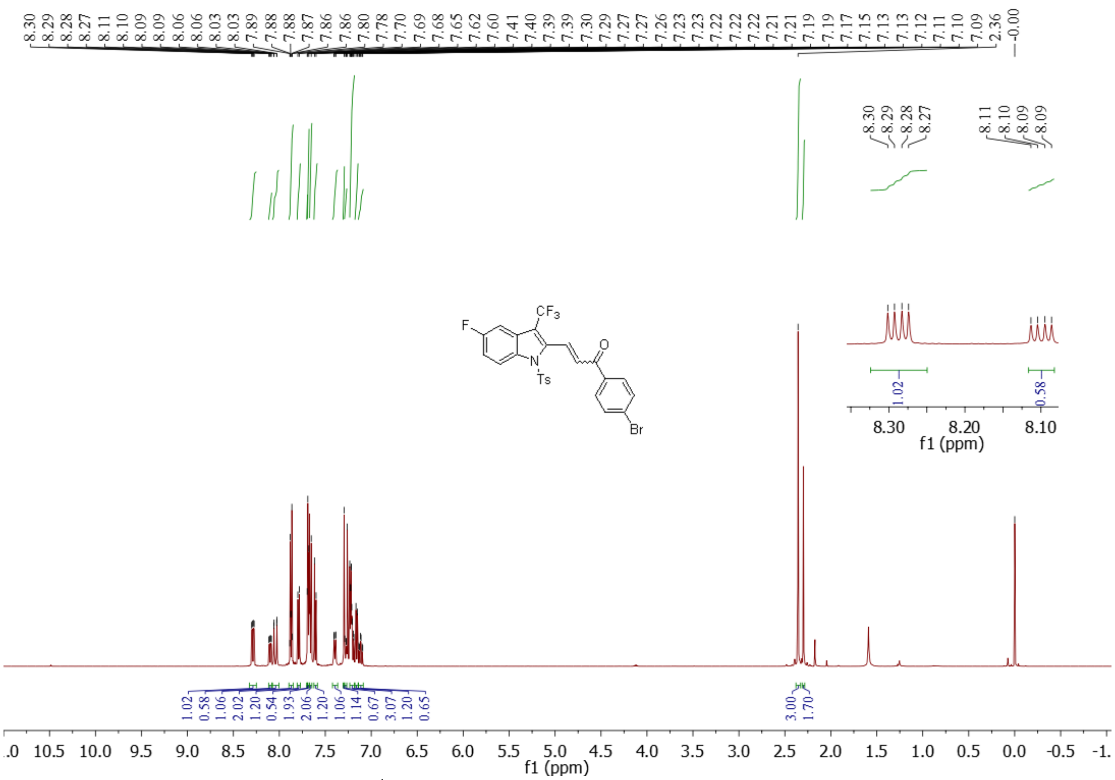

Figure S117. ^{1}H NMR spectrum of **6bg**, related to **Scheme 6**.

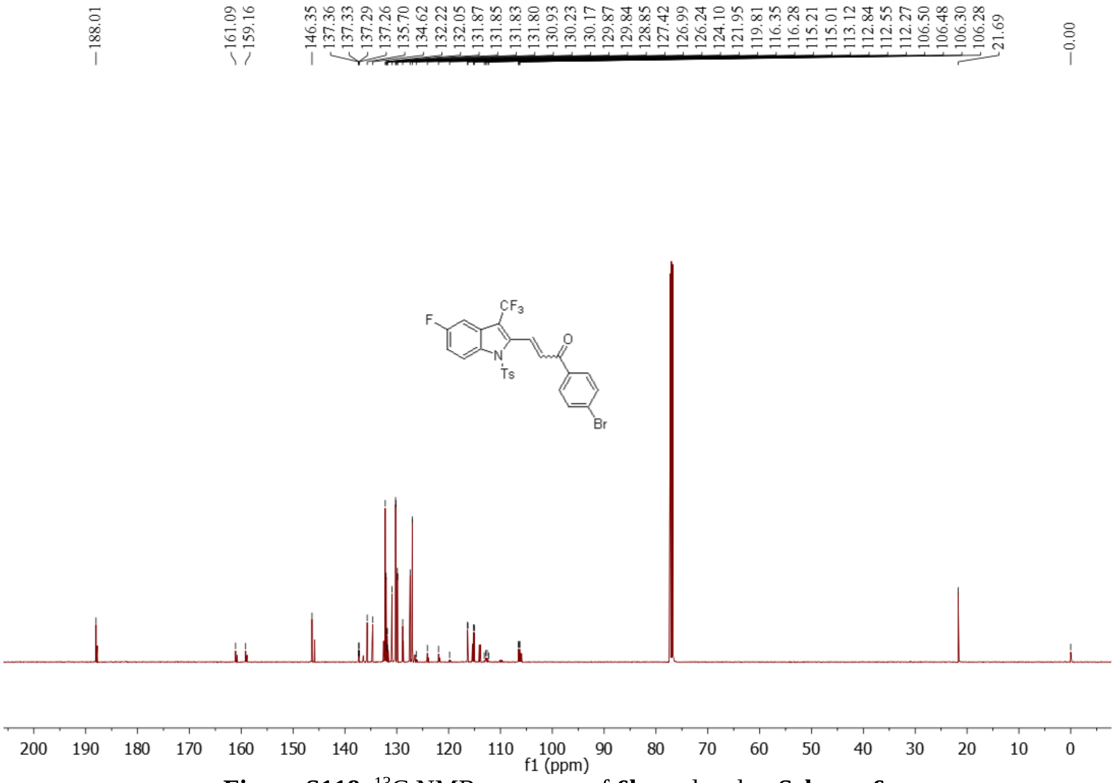

Figure S118. ^{13}C NMR spectrum of **6bg**, related to **Scheme 6**.

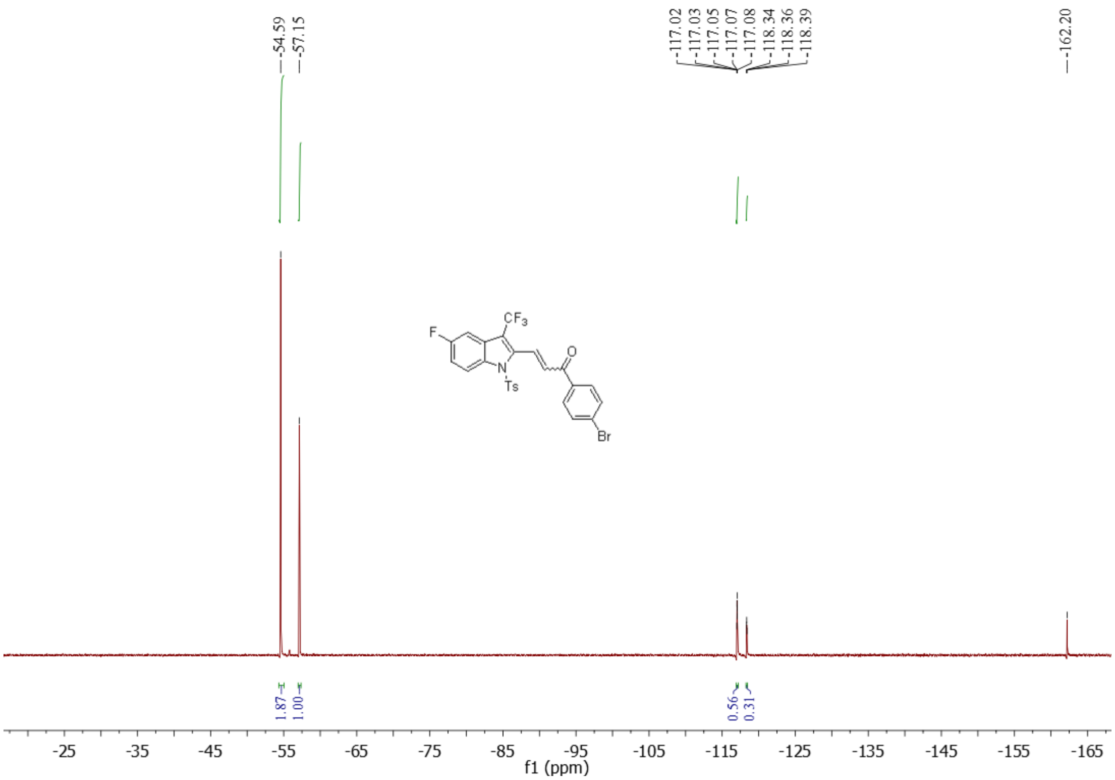

Figure S119. ^{19}F NMR spectrum of **6bg**, related to **Scheme 6**.

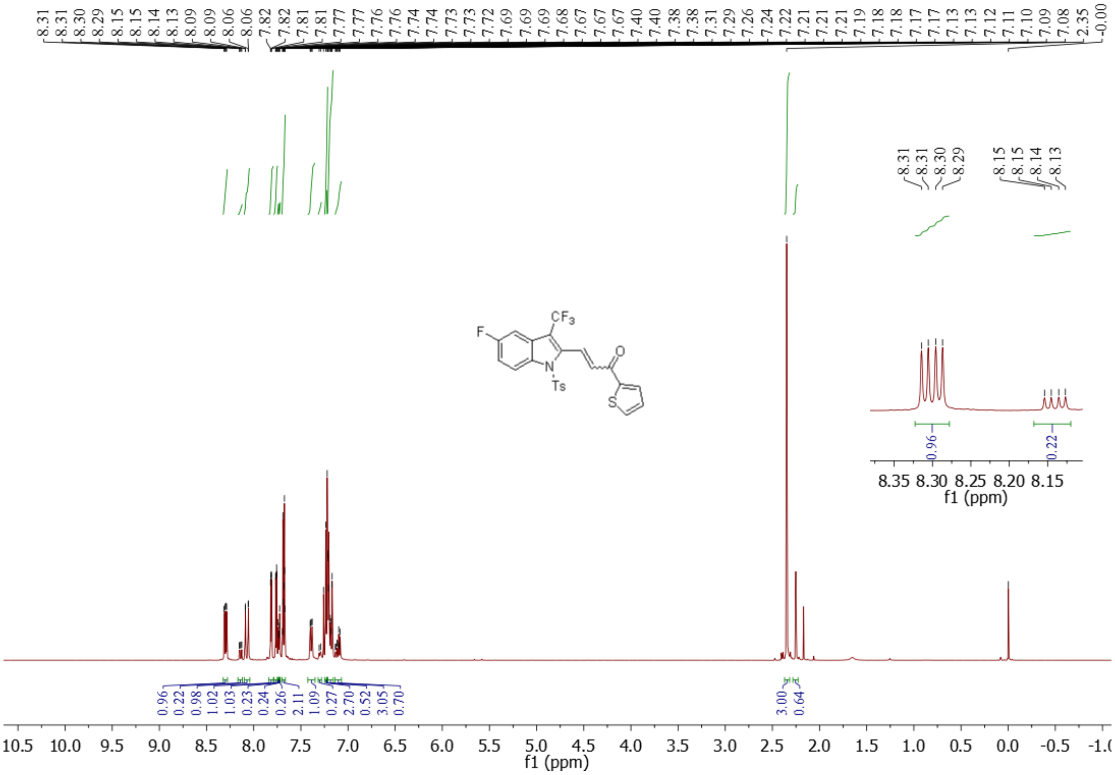

Figure S120. ^{1}H NMR spectrum of **6bh**, related to **Scheme 6**.

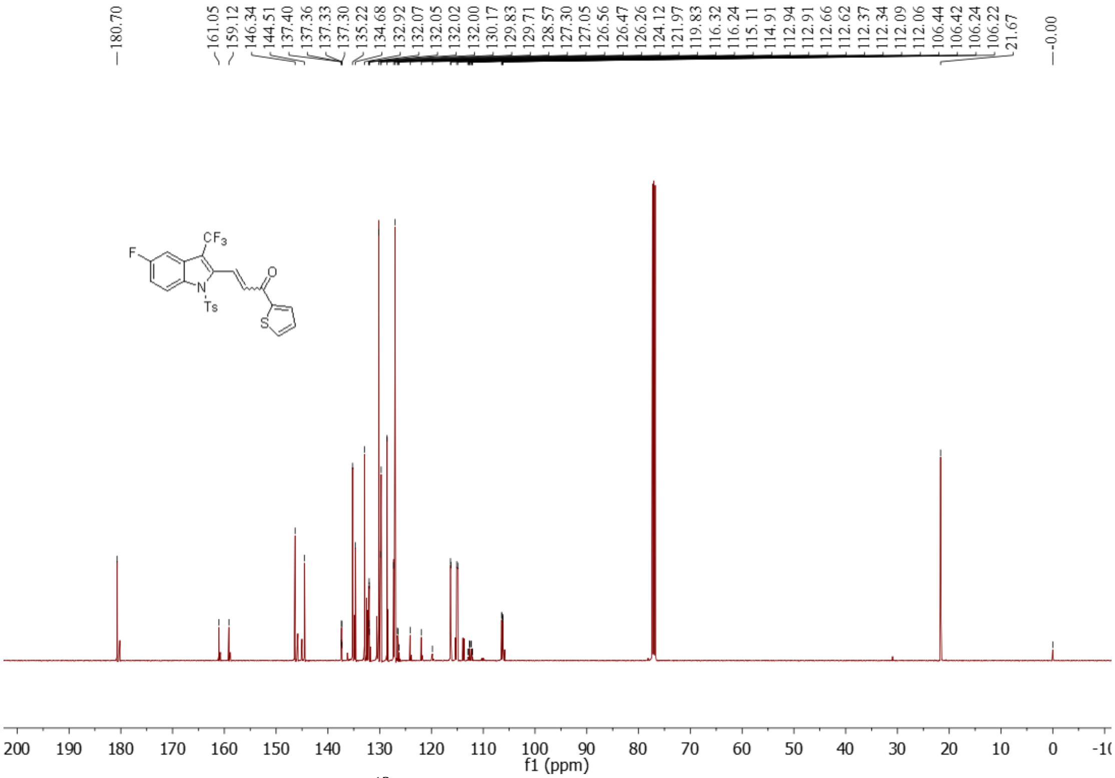

Figure S121. ^{13}C NMR spectrum of **6bh**, related to **Scheme 6**.

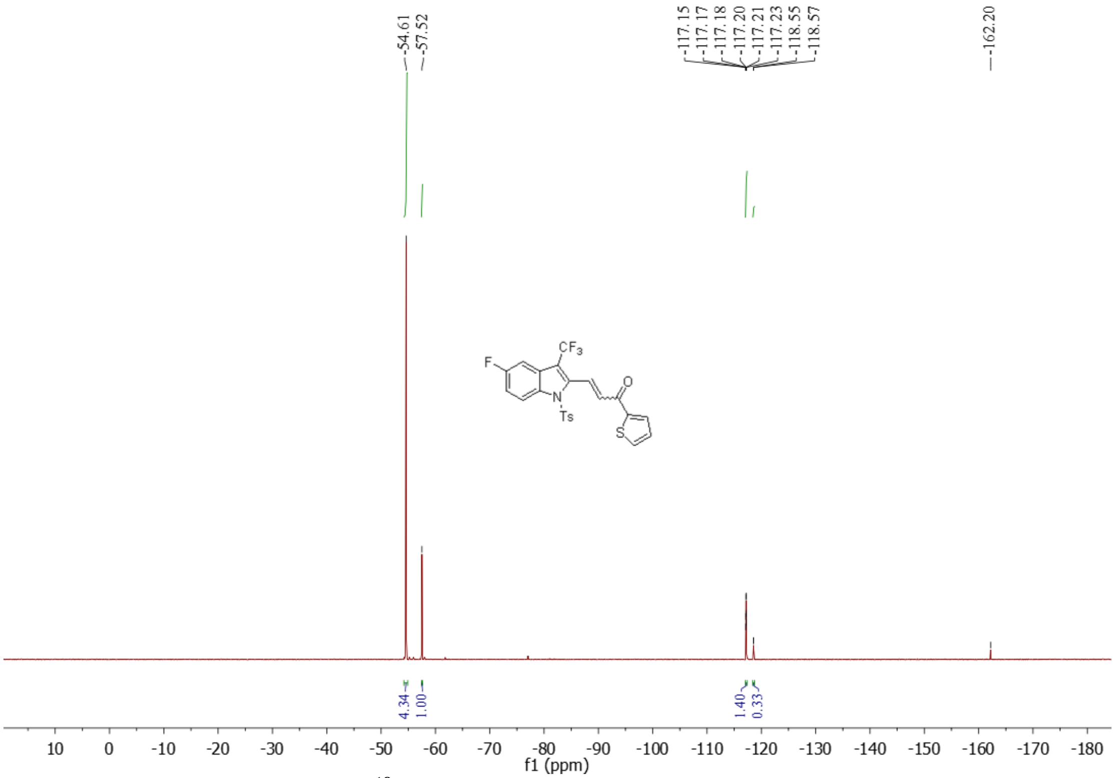

Figure S122. ^{19}F NMR spectrum of **6bh**, related to **Scheme 6**.

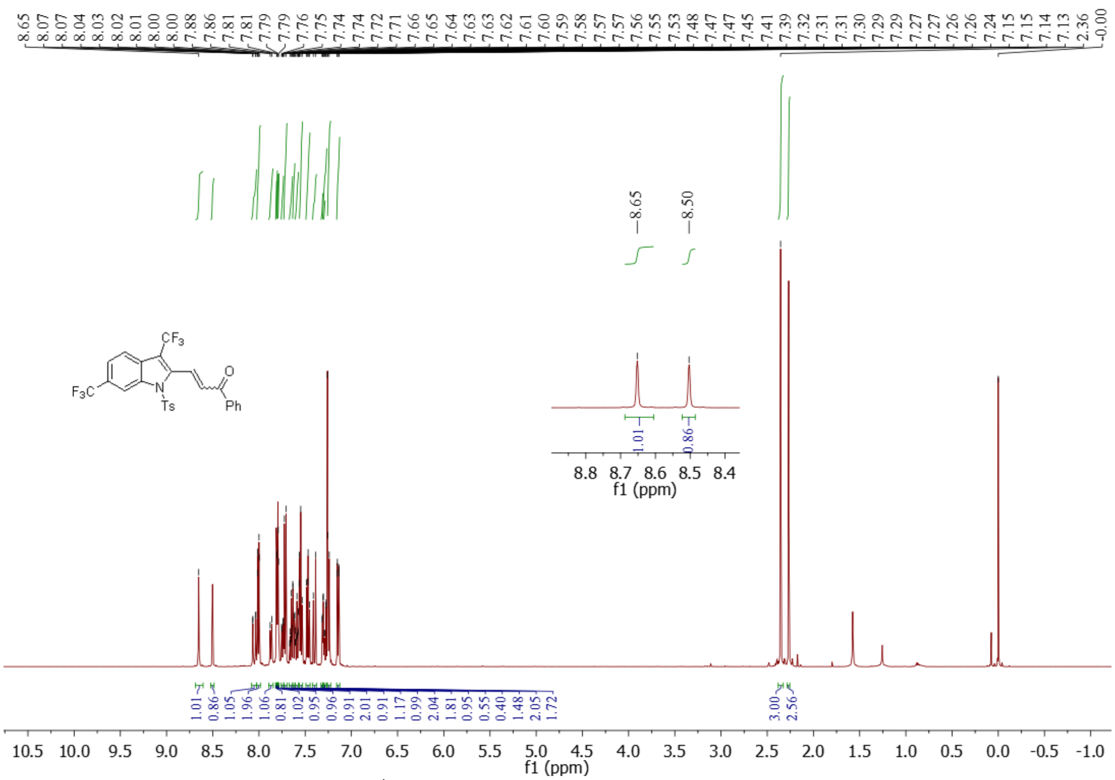

Figure S123. ^{1}H NMR spectrum of **6ca**, related to **Scheme 6**.

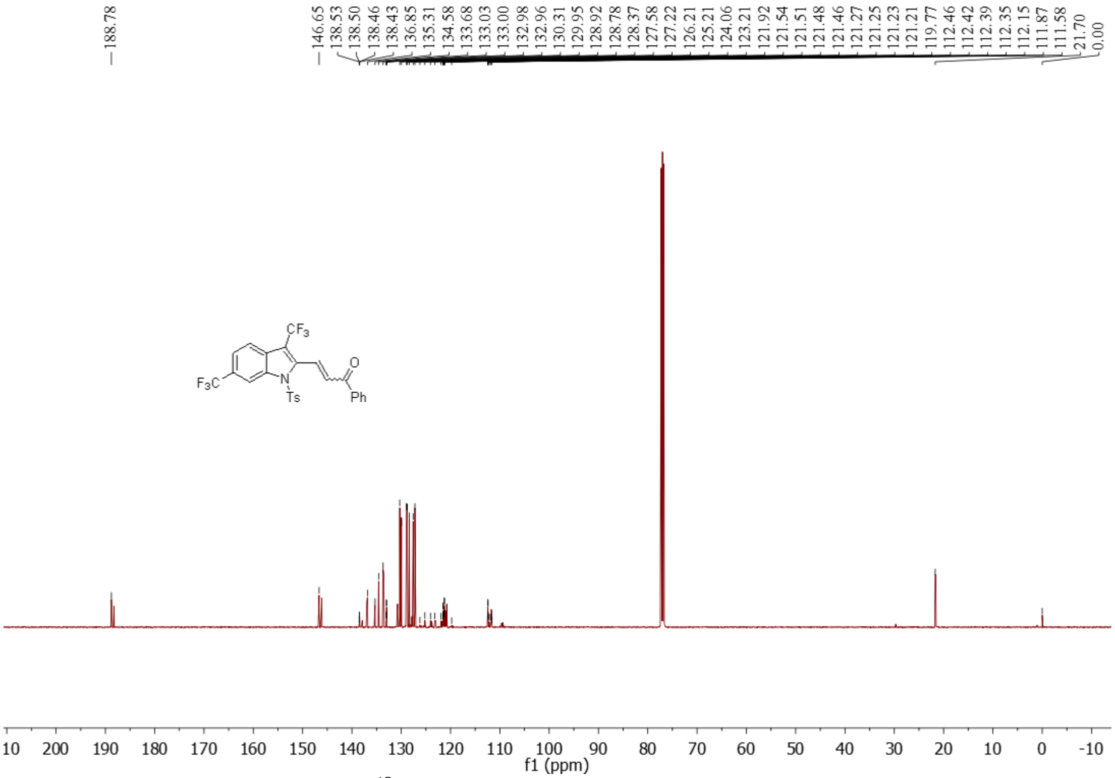

Figure S124. ^{13}C NMR spectrum of **6ca**, related to **Scheme 6**.

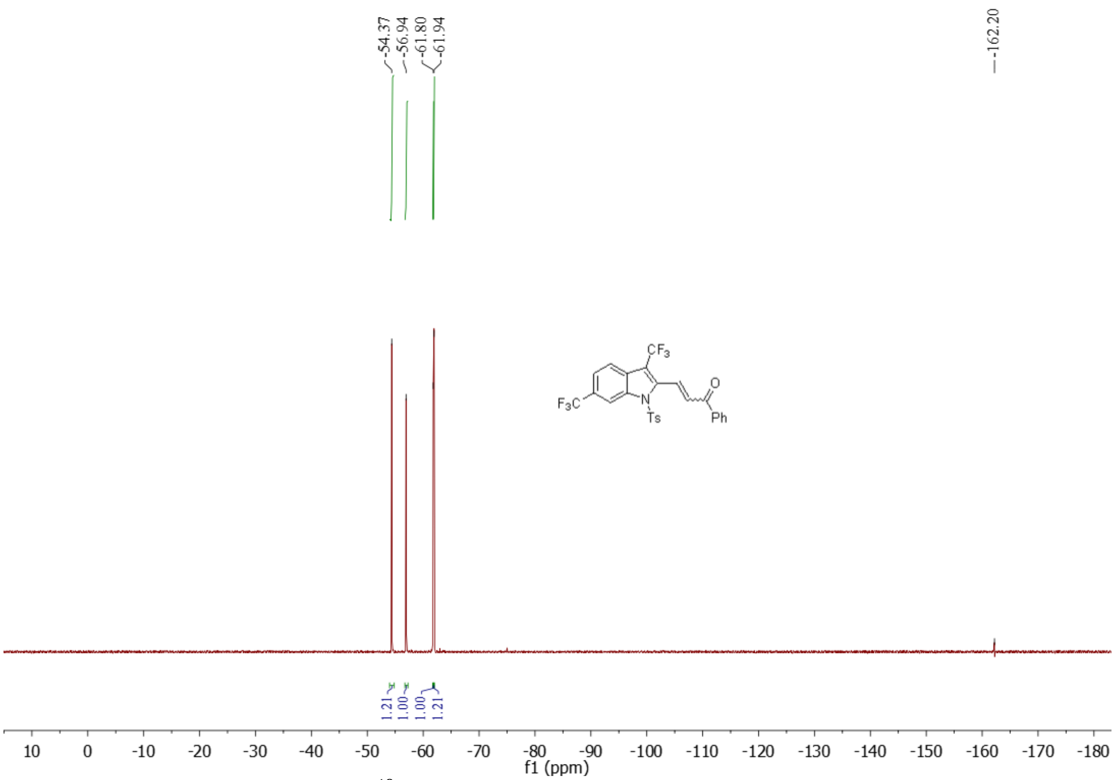

Figure S125. ^{19}F NMR spectrum of **6ca**, related to **Scheme 6**.

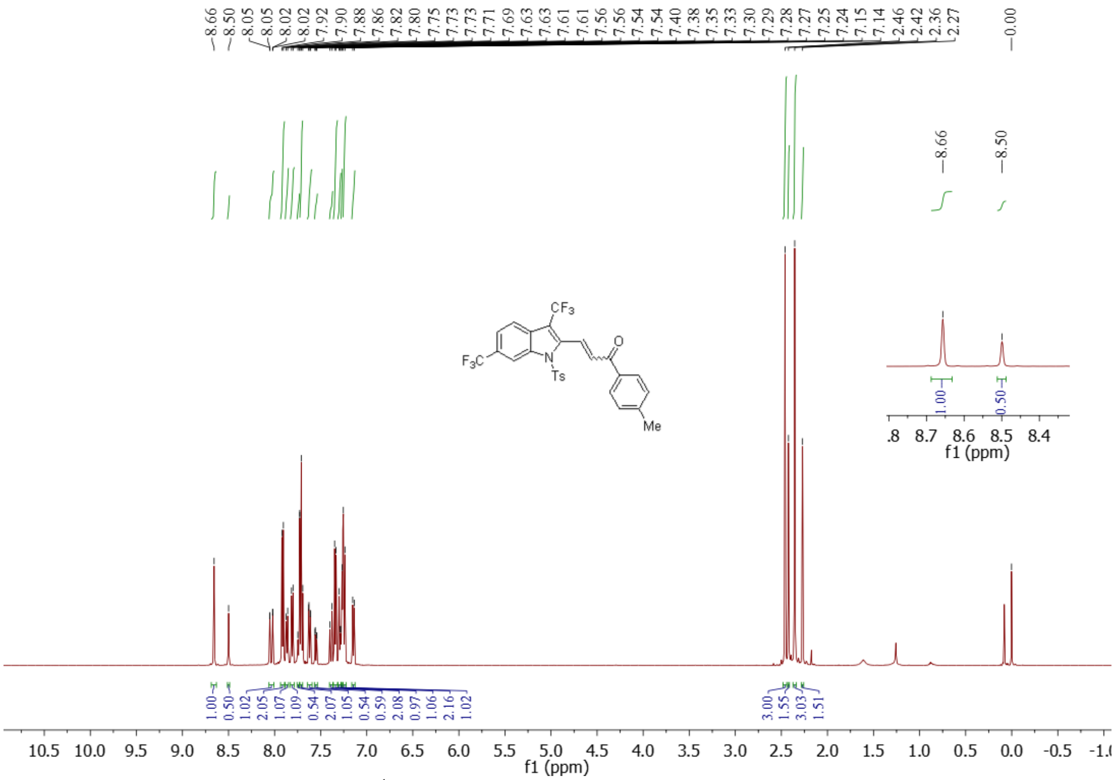

Figure S126. ^{1}H NMR spectrum of **6cc**, related to **Scheme 6**.

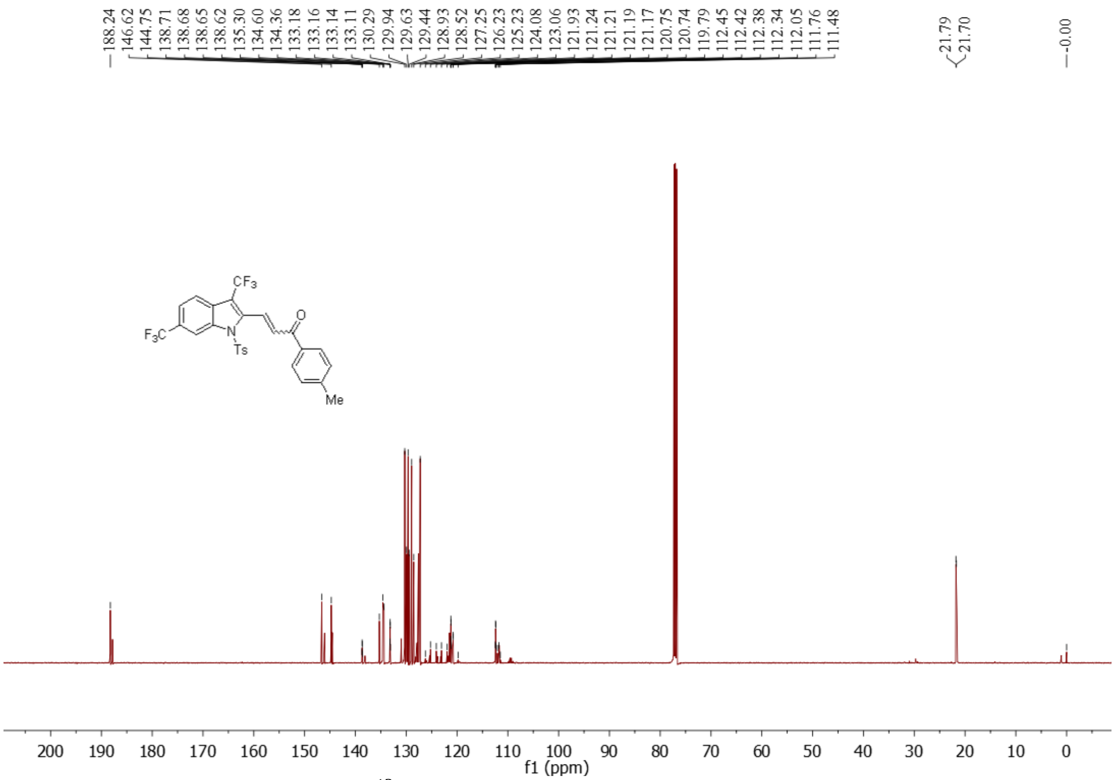

Figure S127. ^{13}C NMR spectrum of **6cc**, related to **Scheme 6**.

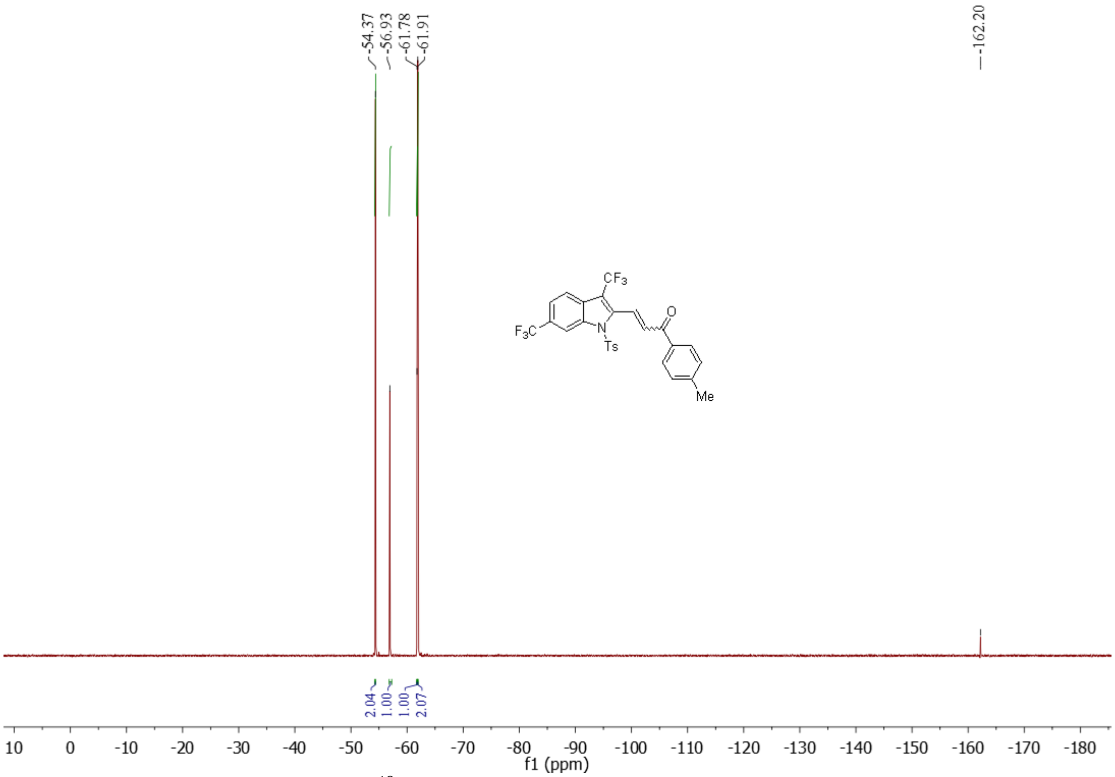

Figure S128. ^{19}F NMR spectrum of **6cc**, related to **Scheme 6**.

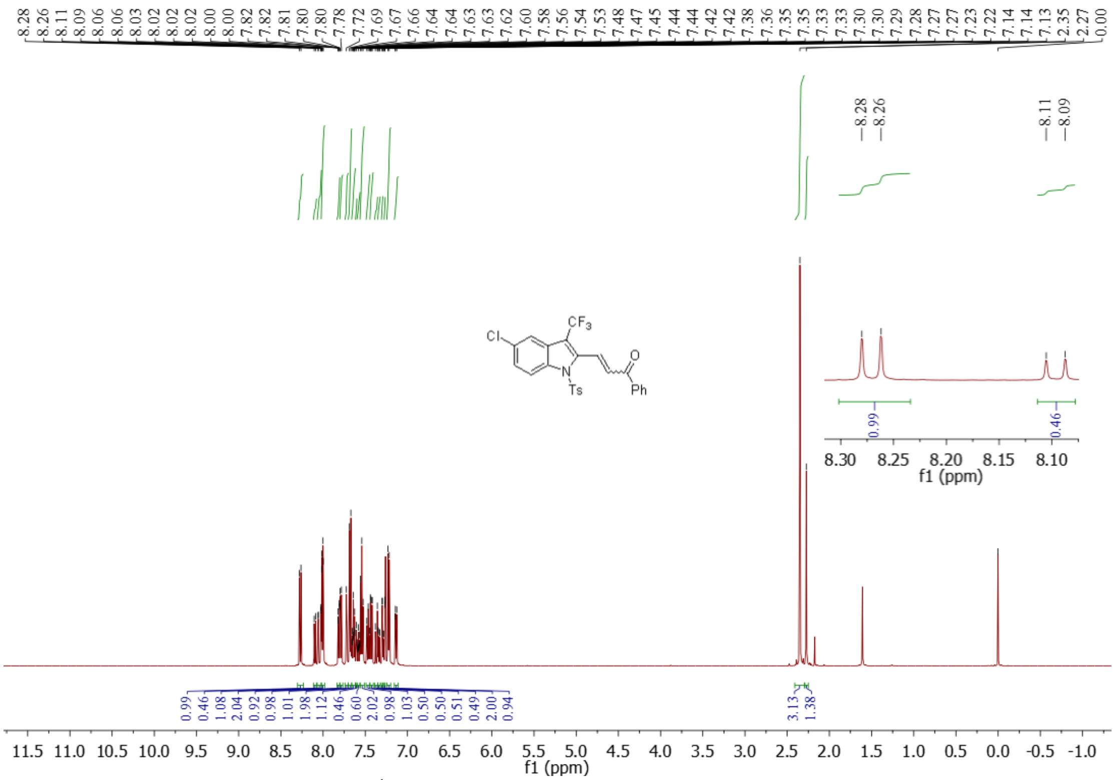

Figure S129. ^{1}H NMR spectrum of **6da**, related to **Scheme 6**.

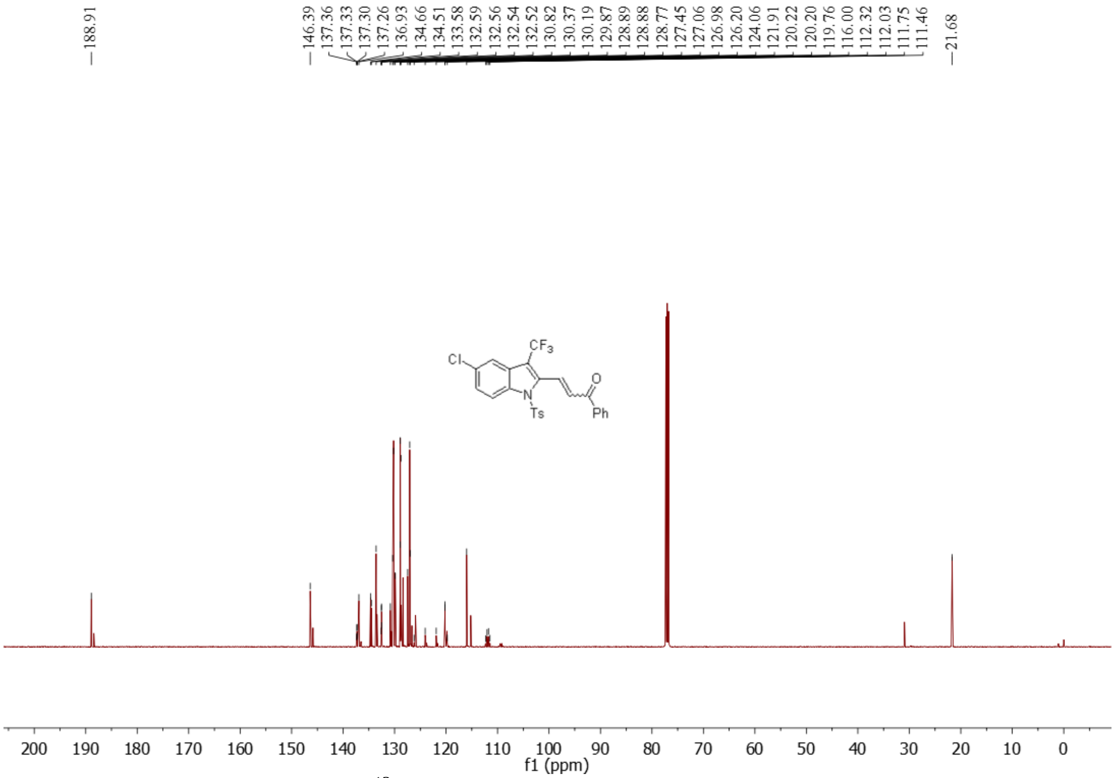

Figure S130. ^{13}C NMR spectrum of **6da**, related to **Scheme 6**.

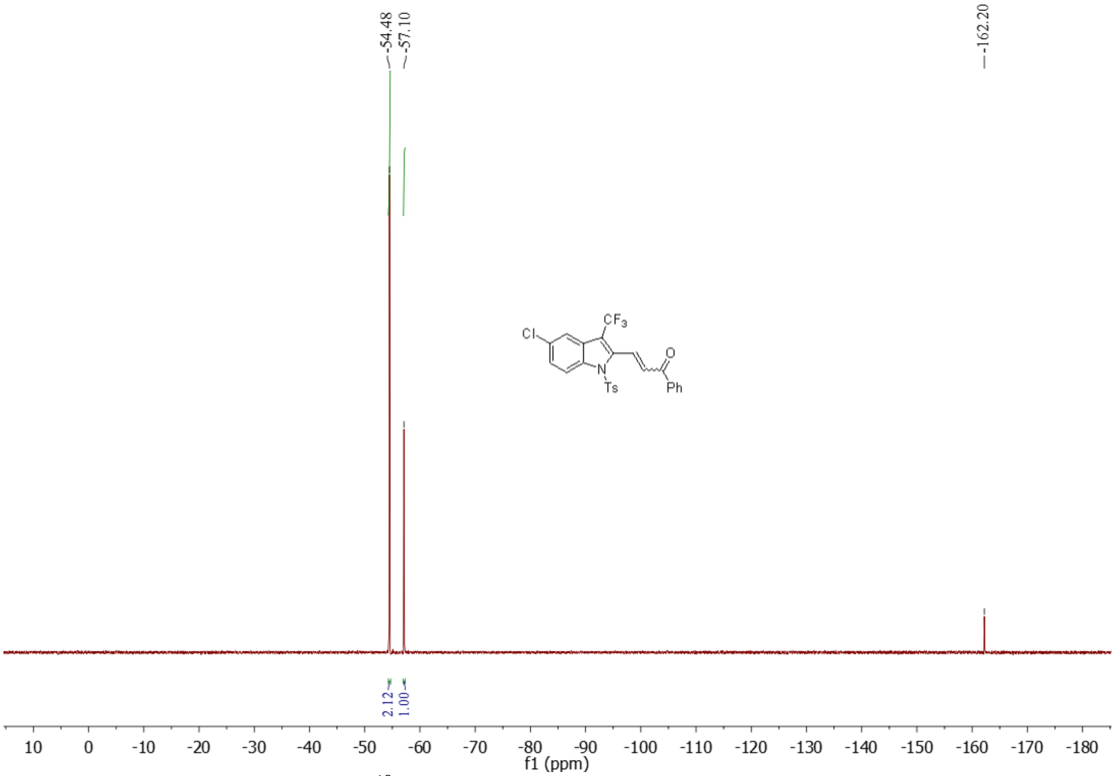

Figure S131. ^{19}F NMR spectrum of **6da**, related to **Scheme 6**.

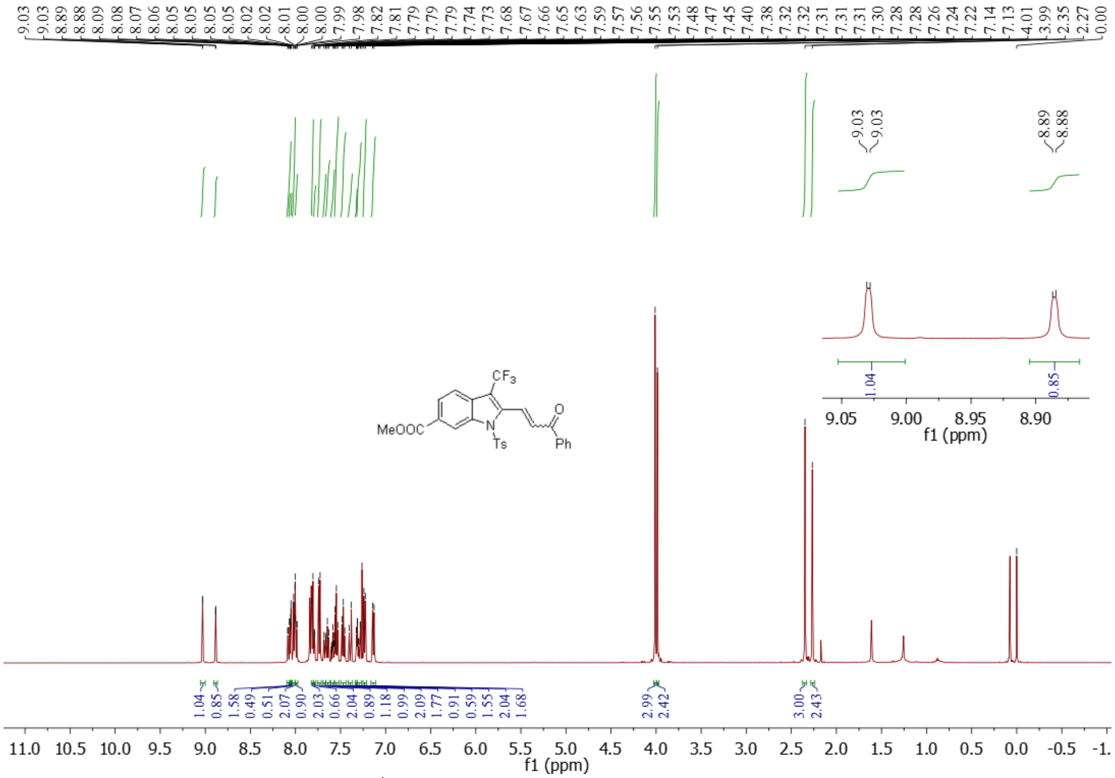

Figure S132. ^{1}H NMR spectrum of **6ga**, related to **Scheme 6**.

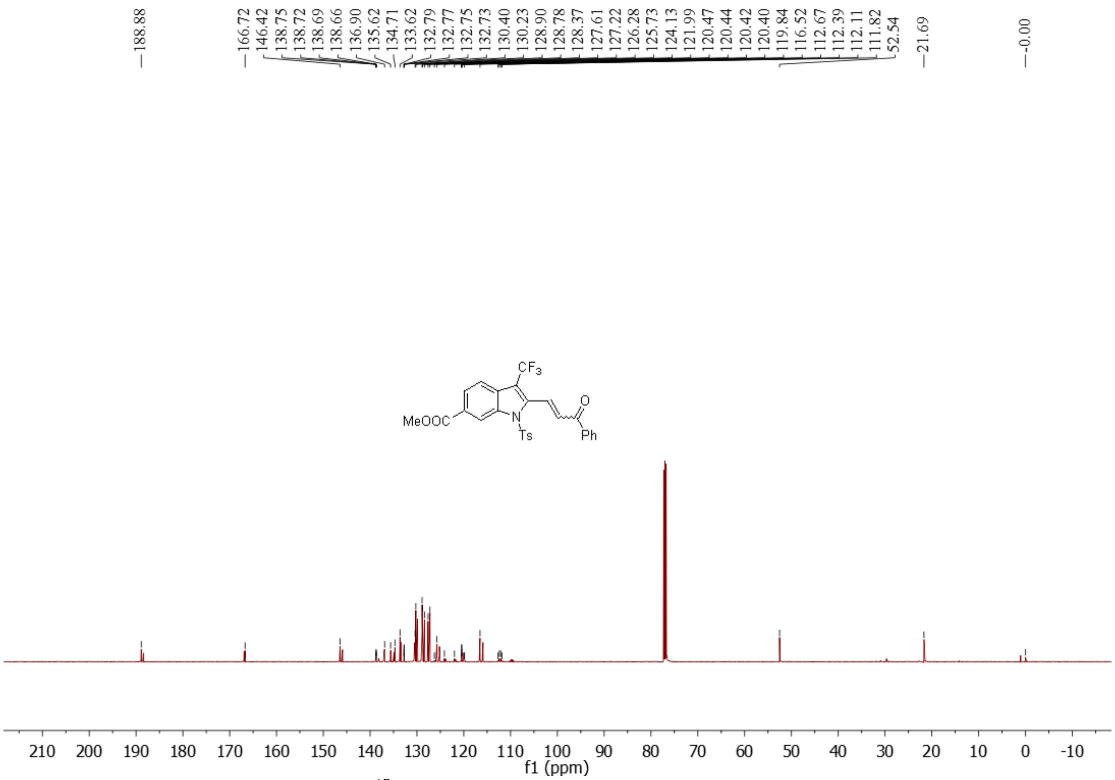

Figure S133. ^{13}C NMR spectrum of **6ga**, related to **Scheme 6**.

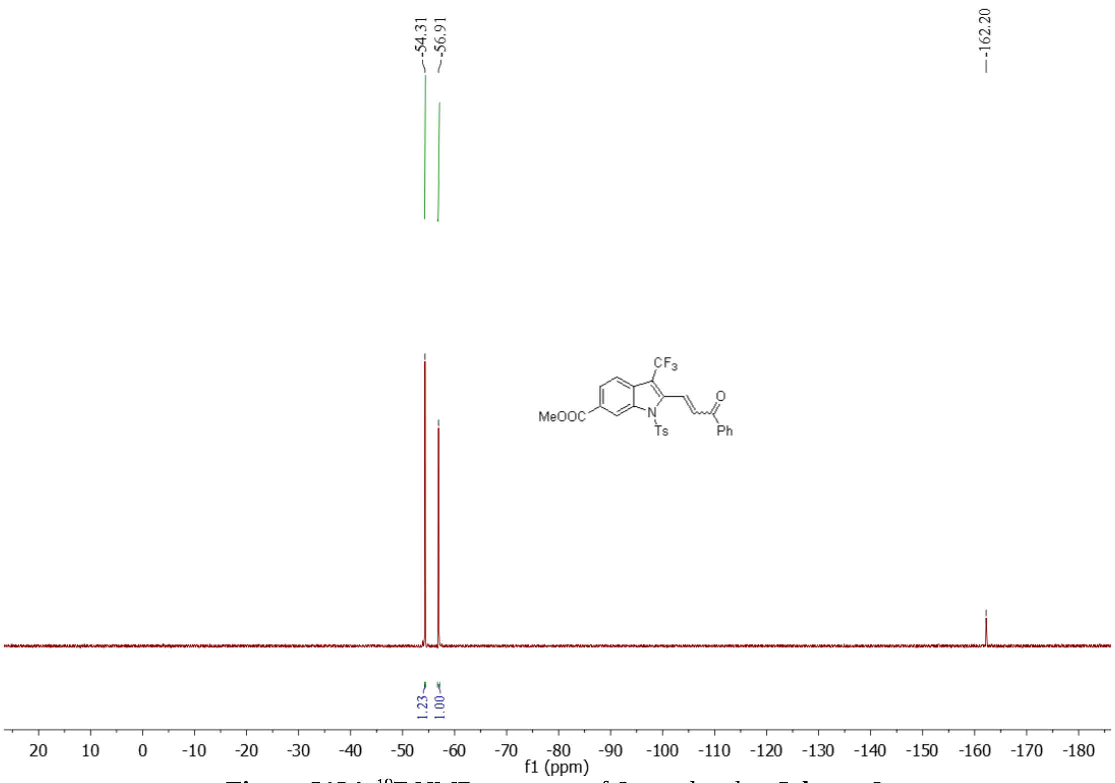

Figure S134. ^{19}F NMR spectrum of **6ga**, related to **Scheme 6**.

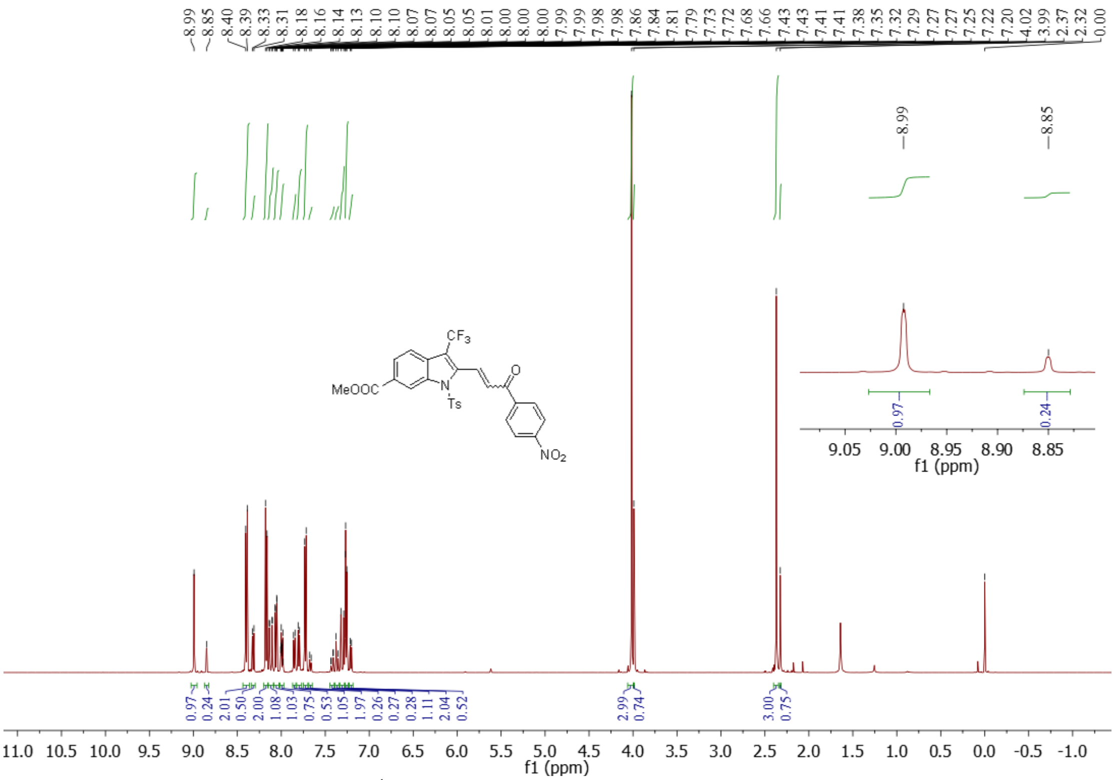

Figure S135. ^{1}H NMR spectrum of **6gd**, related to **Scheme 6**.

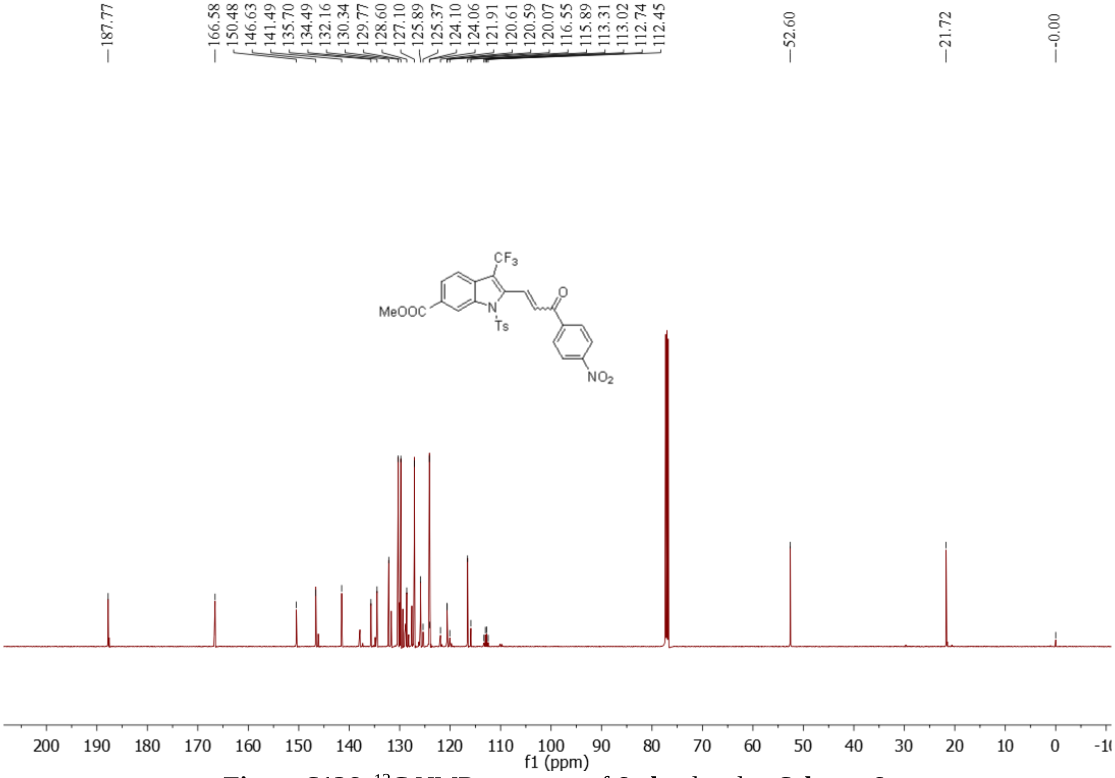

Figure S136. ^{13}C NMR spectrum of **6gd**, related to **Scheme 6**.

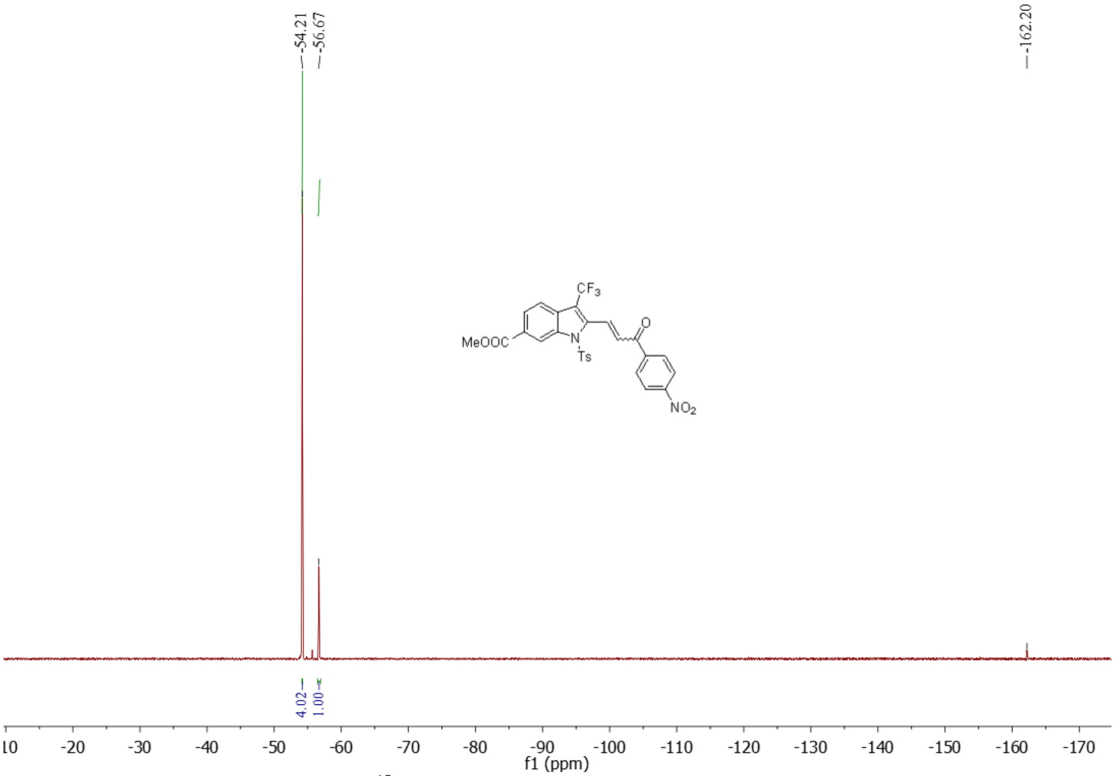

Figure S137. ^{19}F NMR spectrum of **6gd**, related to **Scheme 6**.

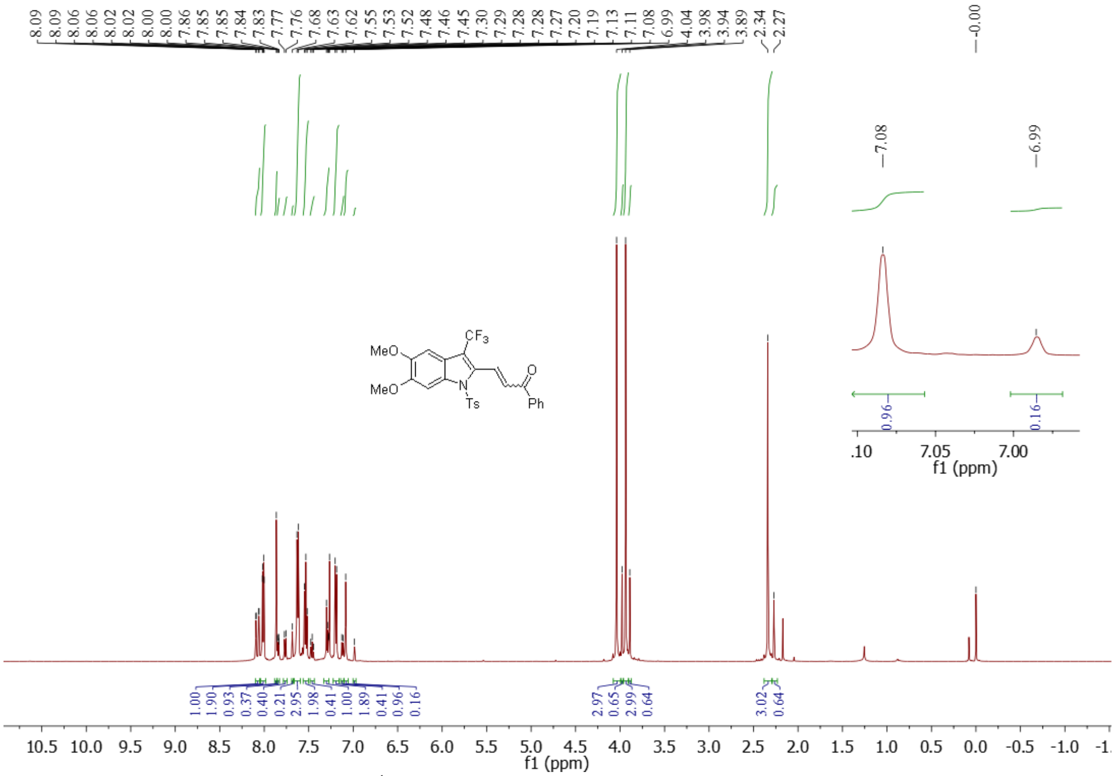

Figure S138. ^{1}H NMR spectrum of **6ha**, related to **Scheme 6**.

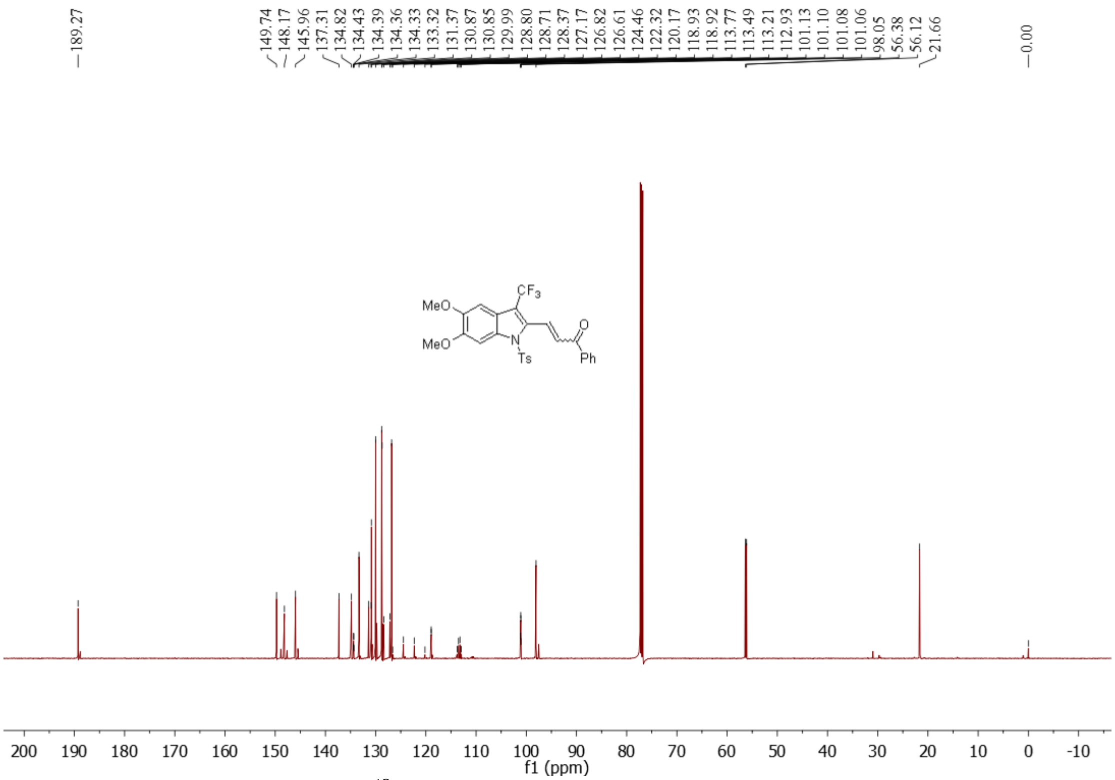

Figure S139. ^{13}C NMR spectrum of **6ha**, related to **Scheme 6**.

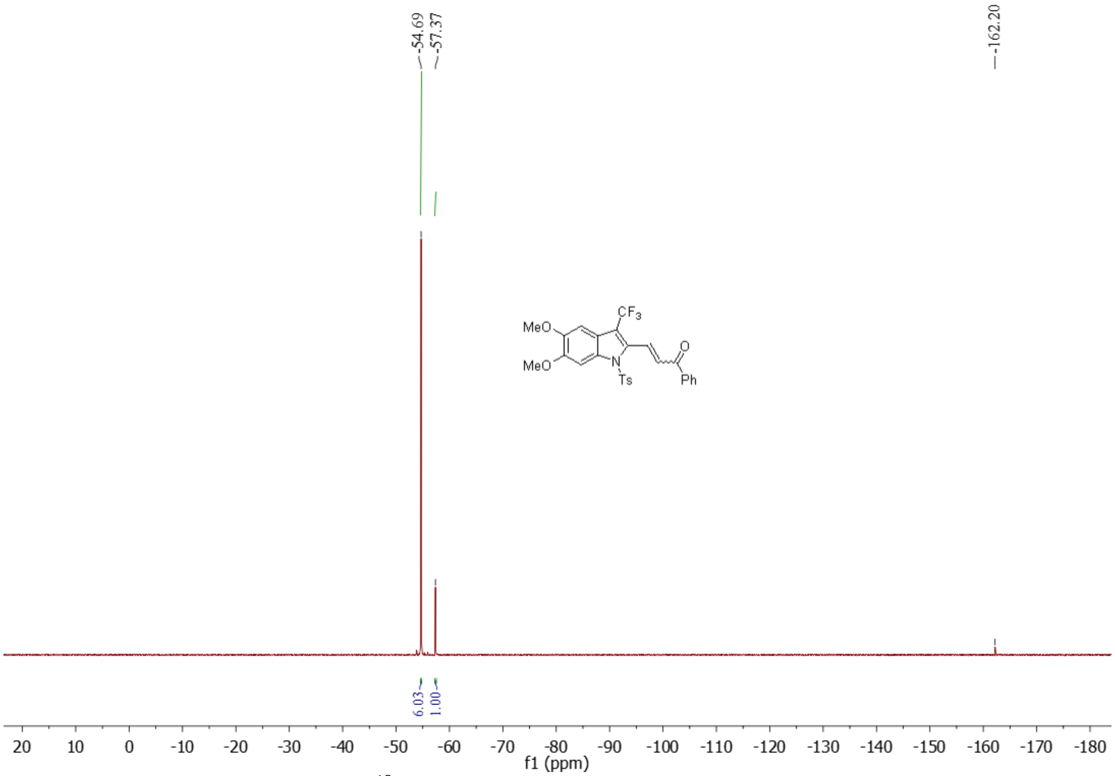

Figure S140. ^{19}F NMR spectrum of **6ha**, related to **Scheme 6**.

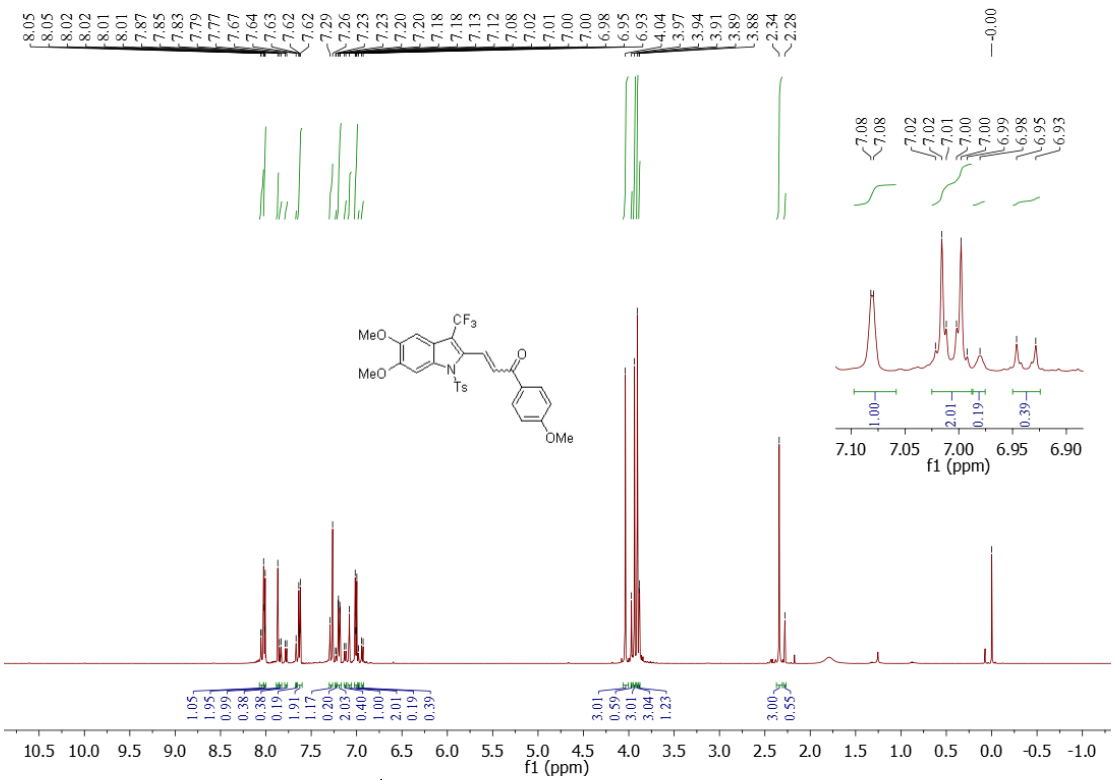

Figure S141. ^{1}H NMR spectrum of **6hb**, related to **Scheme 6**.

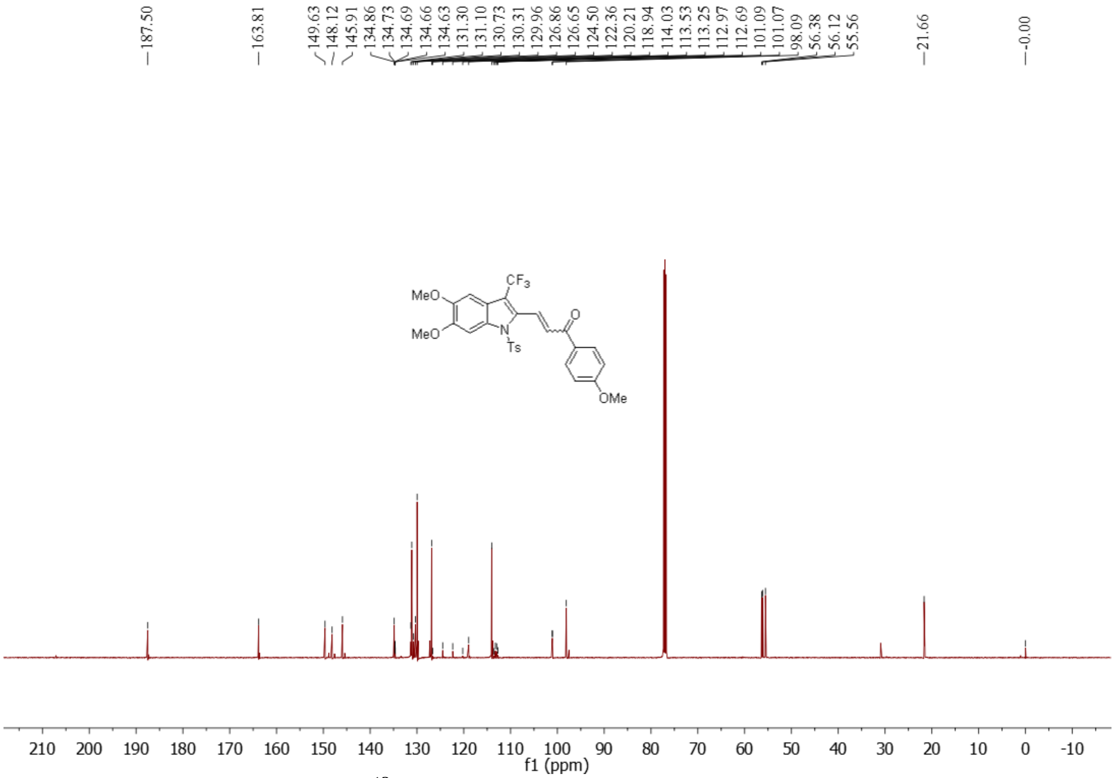

Figure S142. ^{13}C NMR spectrum of **6hb**, related to **Scheme 6**.

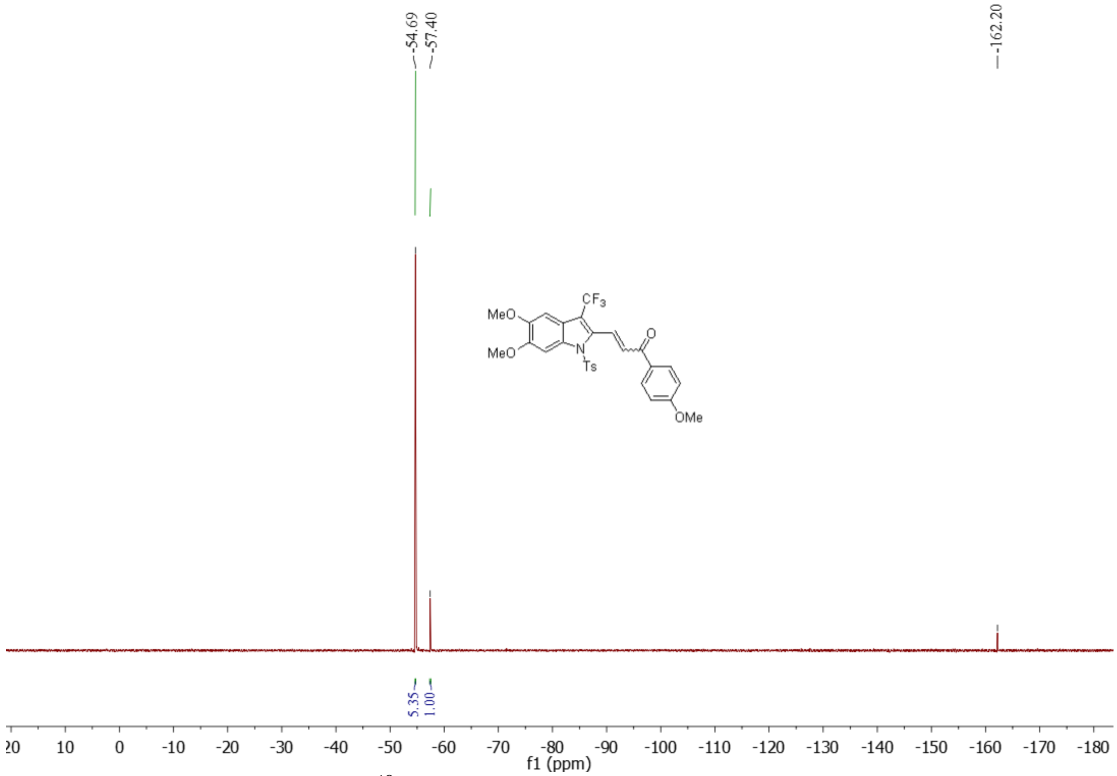

Figure S143. ^{19}F NMR spectrum of **6hb**, related to **Scheme 6**.

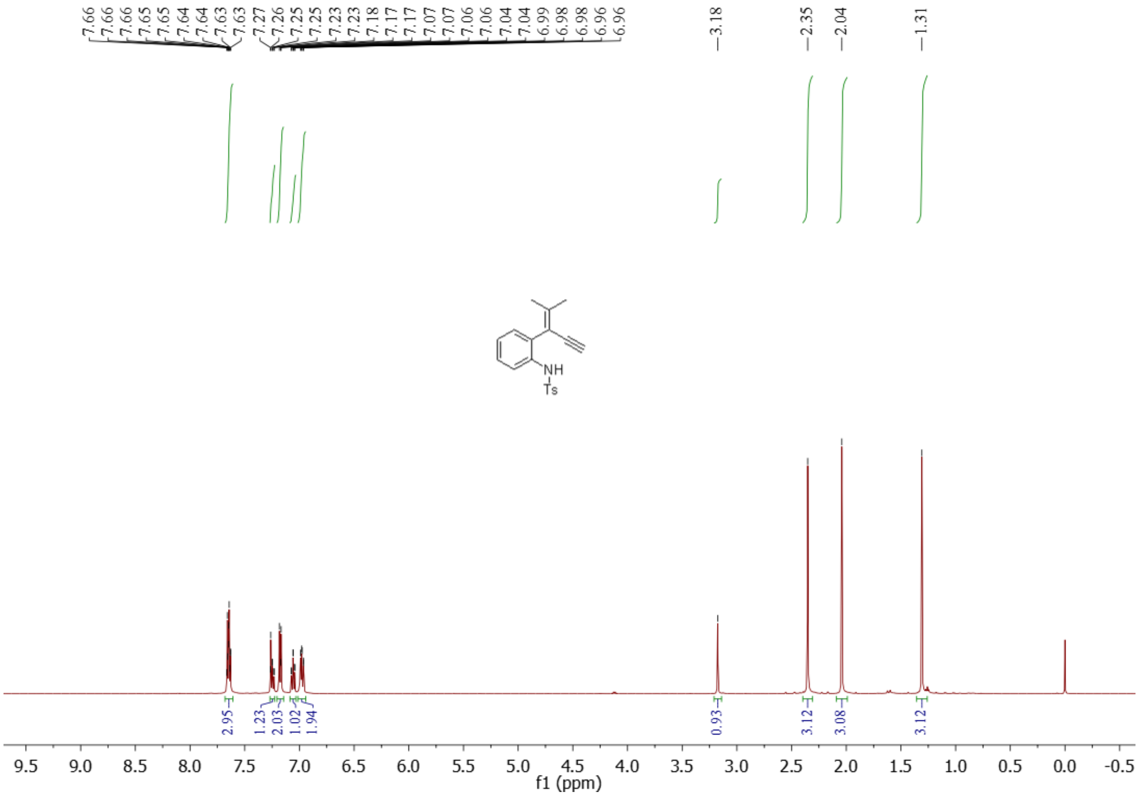

Figure S144. ^{1}H NMR spectrum of **5ha**, related to **Figure 2**.

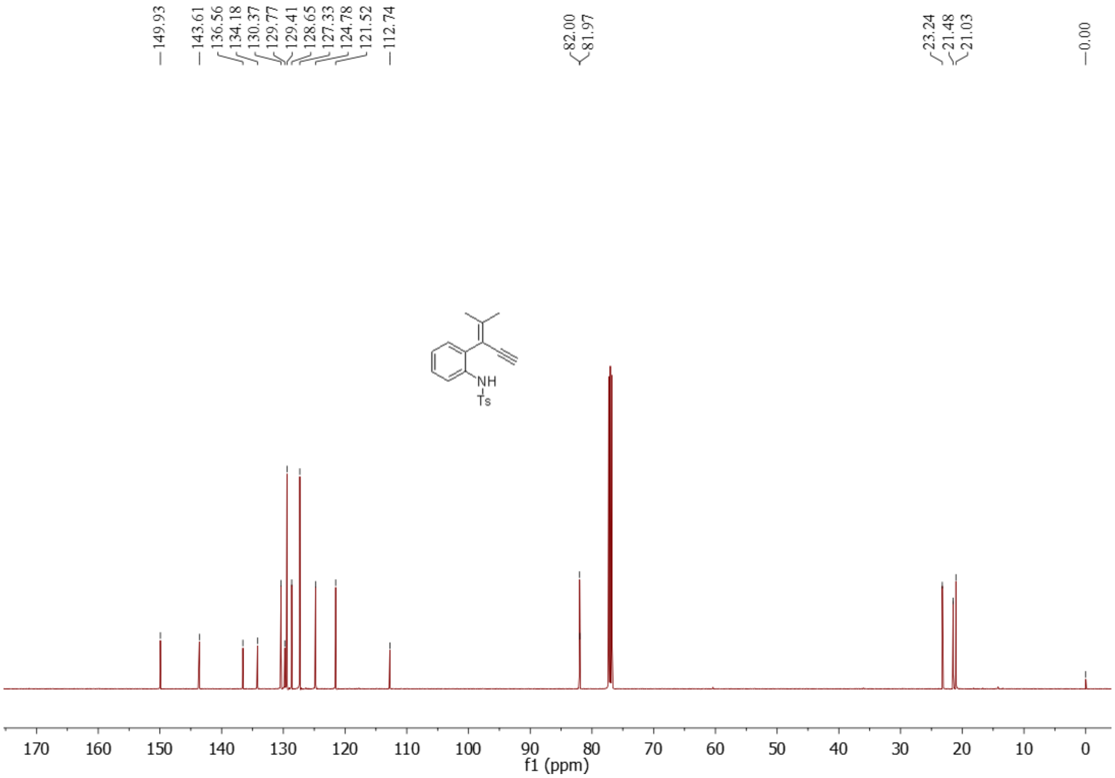

Figure S145. ^{13}C NMR spectrum of **5ha**, related to **Figure 2**.

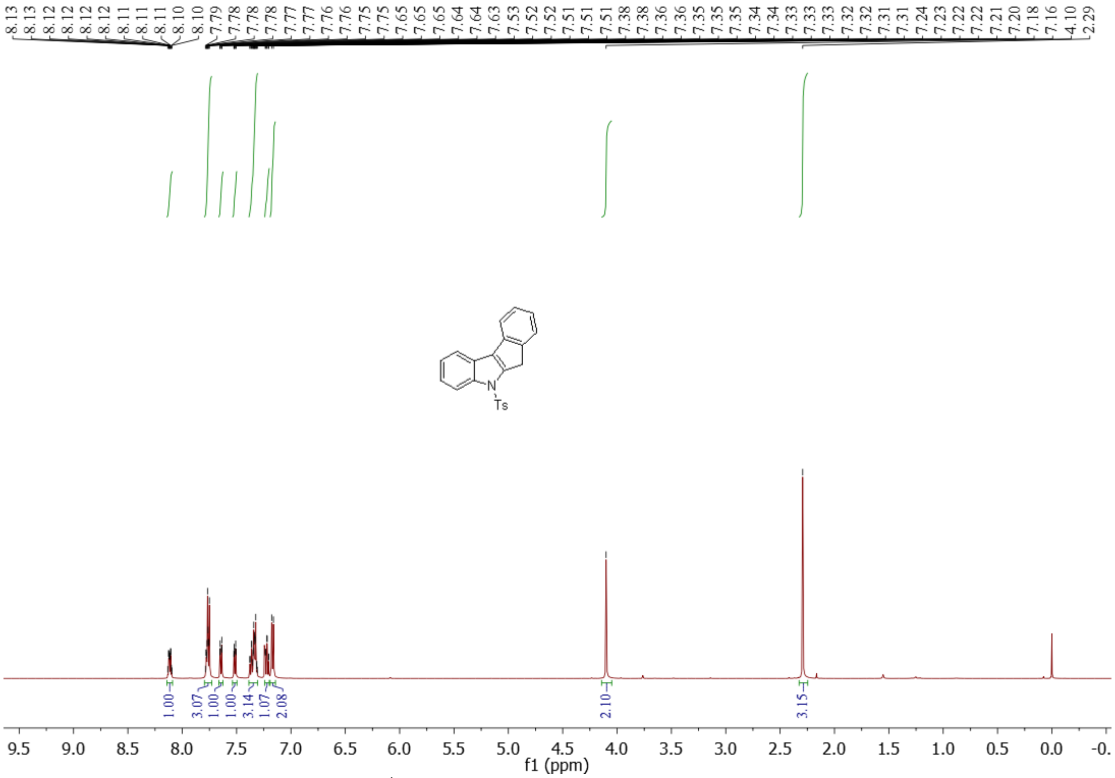

Figure S146. ^{1}H NMR spectrum of **5ia**, related to **Figure 2**.

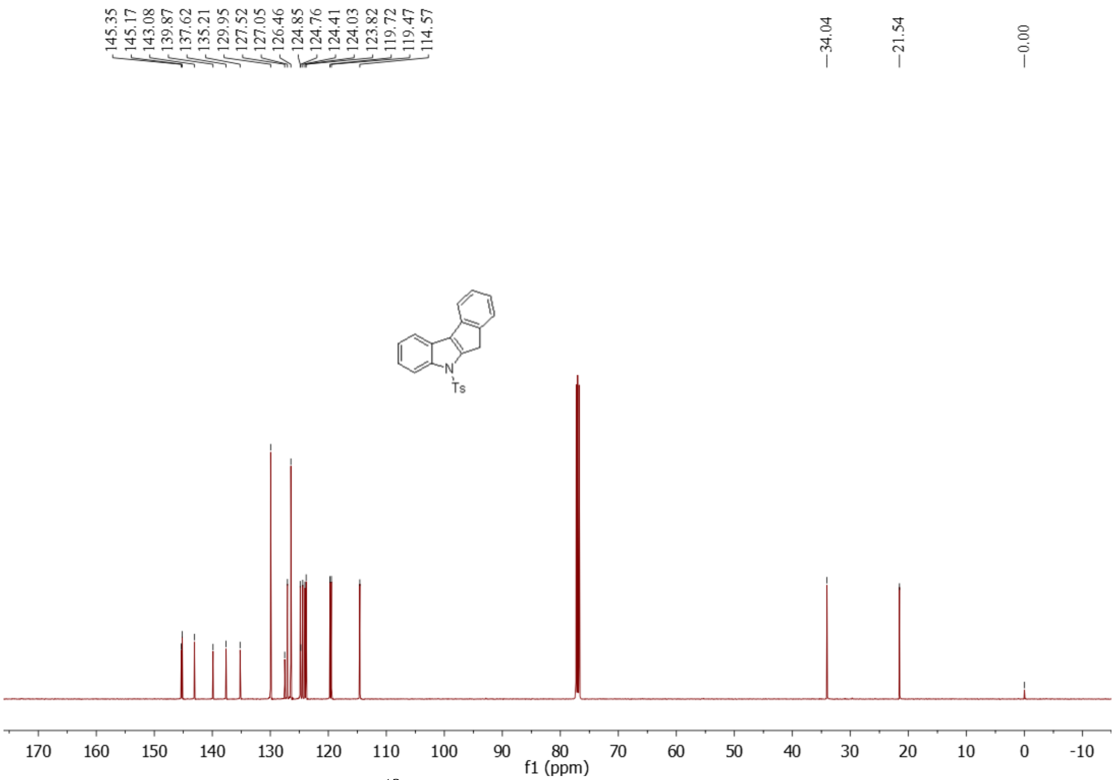

Figure S147. ^{13}C NMR spectrum of **5ia**, related to **Figure 2**.

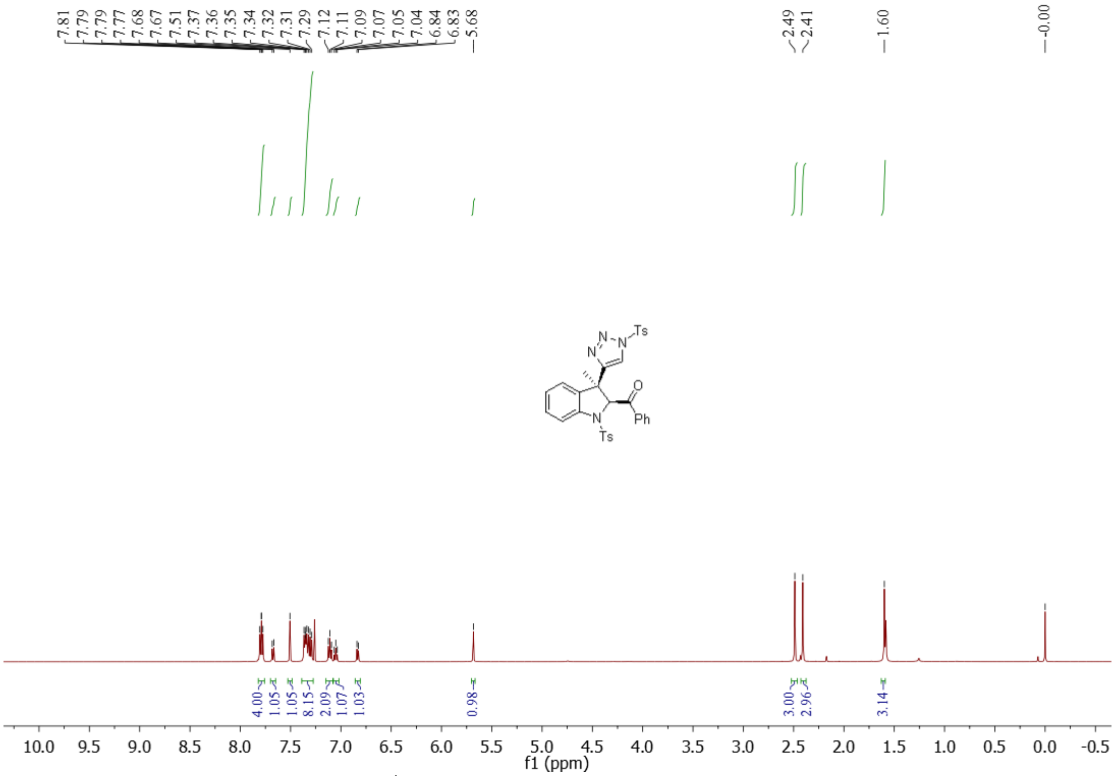

Figure S148. ^{1}H NMR spectrum of **7**, related to **Scheme 5**.

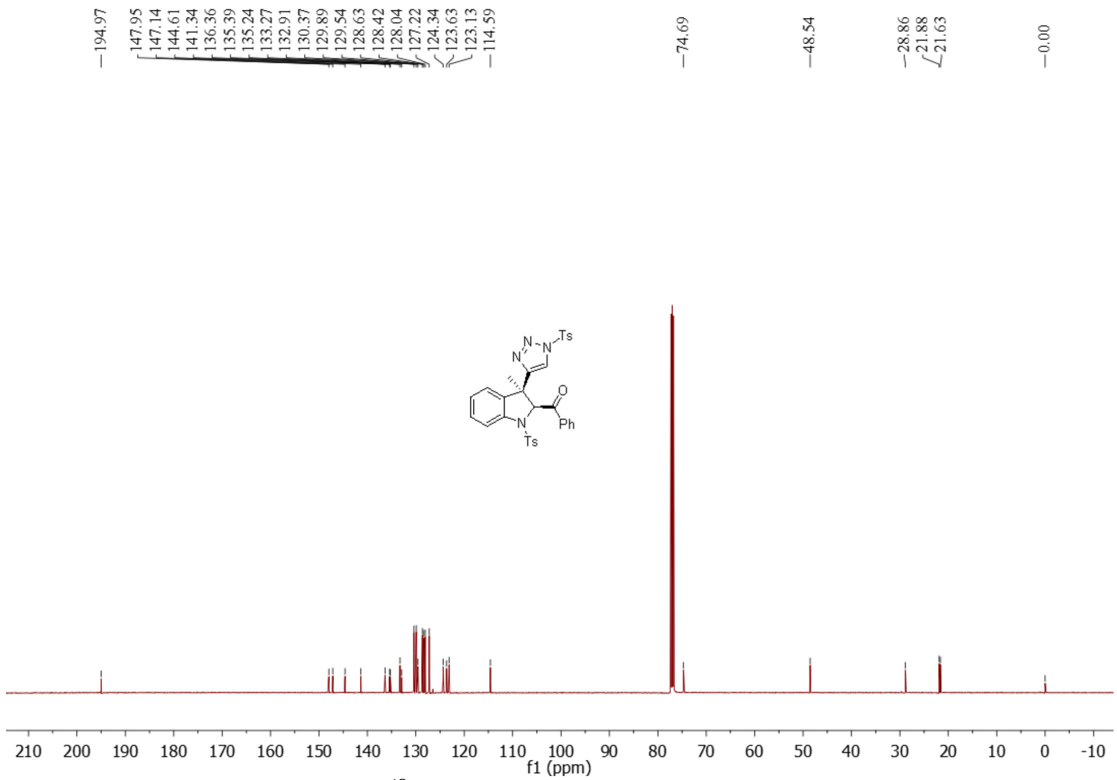

Figure S149. ^{13}C NMR spectrum of **7**, related to **Scheme 5**.

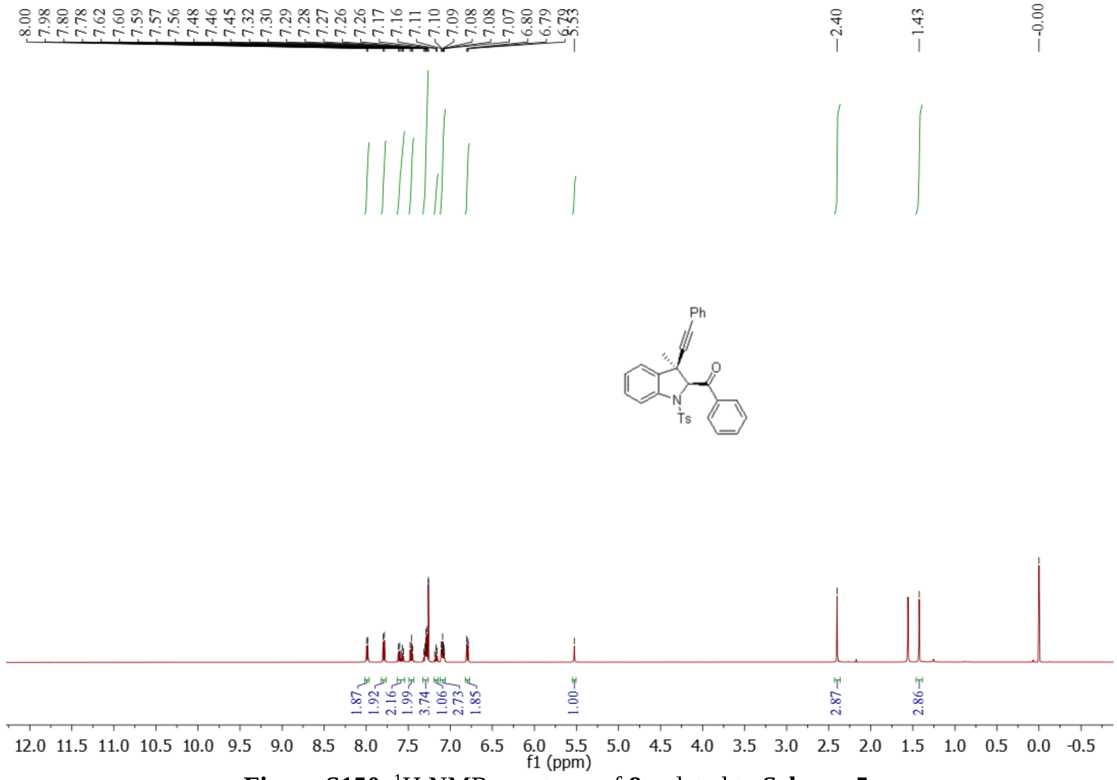

Figure S150. ^{1}H NMR spectrum of **8**, related to **Scheme 5**.

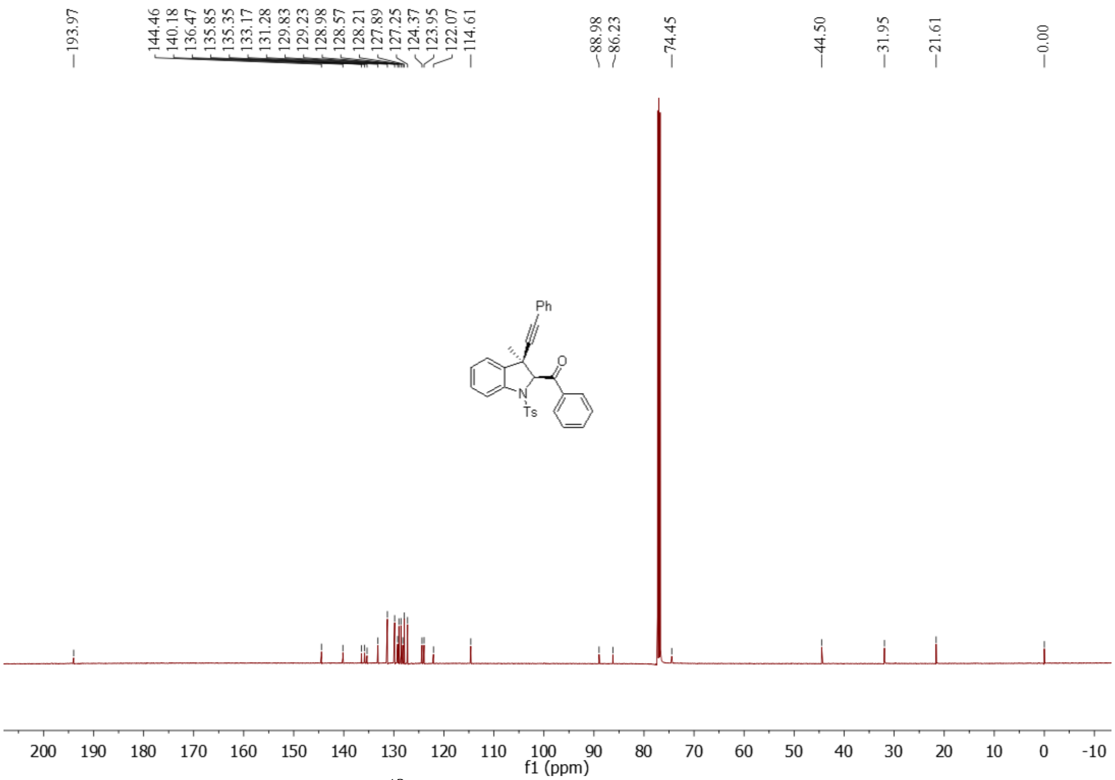

Figure S151. ${}^{13}C$ NMR spectrum of **8**, related to **Scheme 5**.

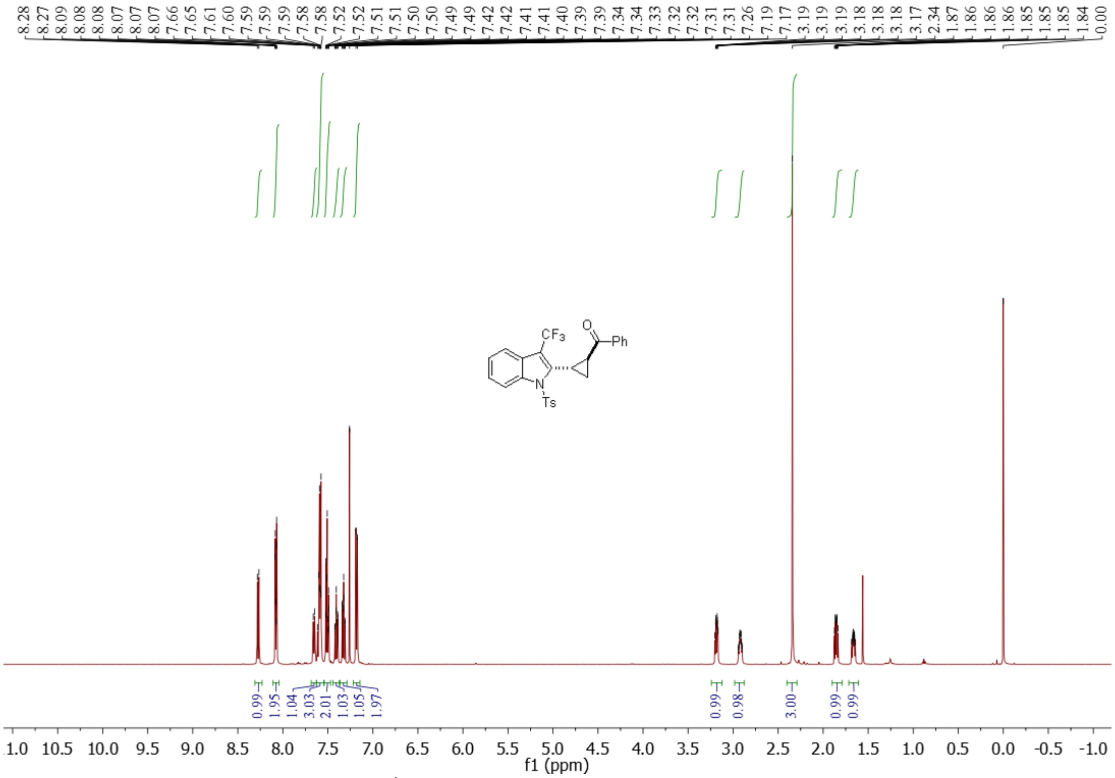

Figure S152. ${}^{1}H$ NMR spectrum of **9**, related to **Scheme 7**.

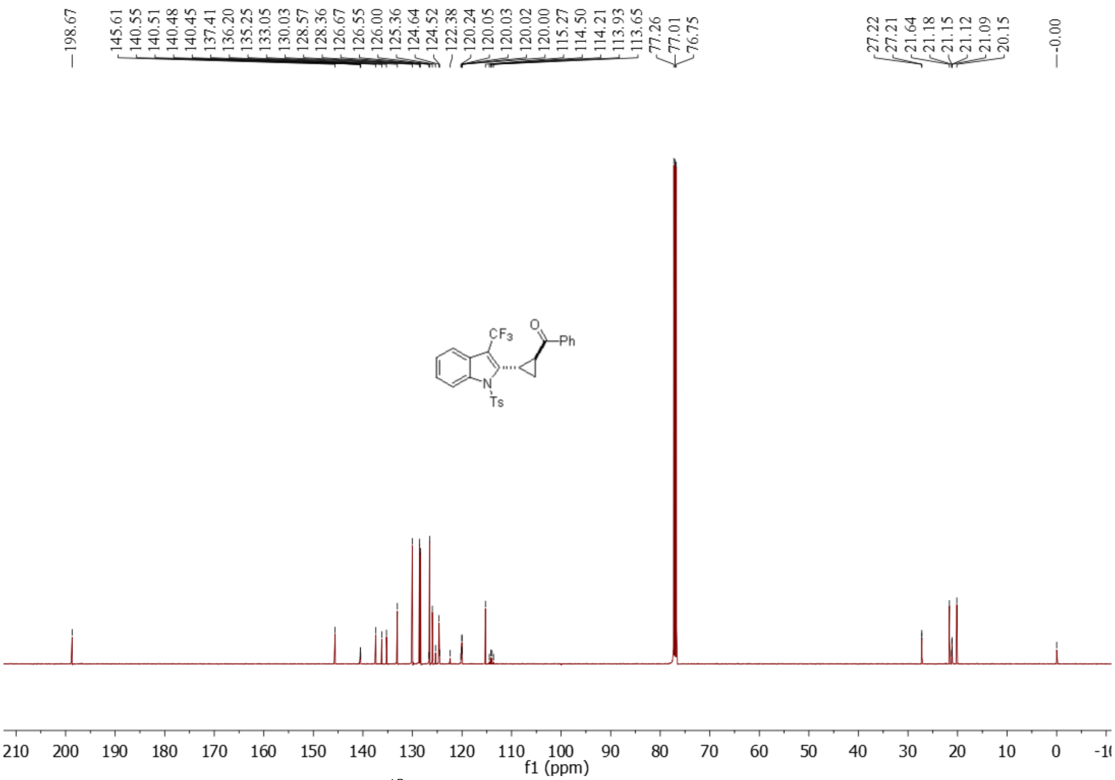

Figure S153. ^{13}C NMR spectrum of **9**, related to **Scheme 7**.

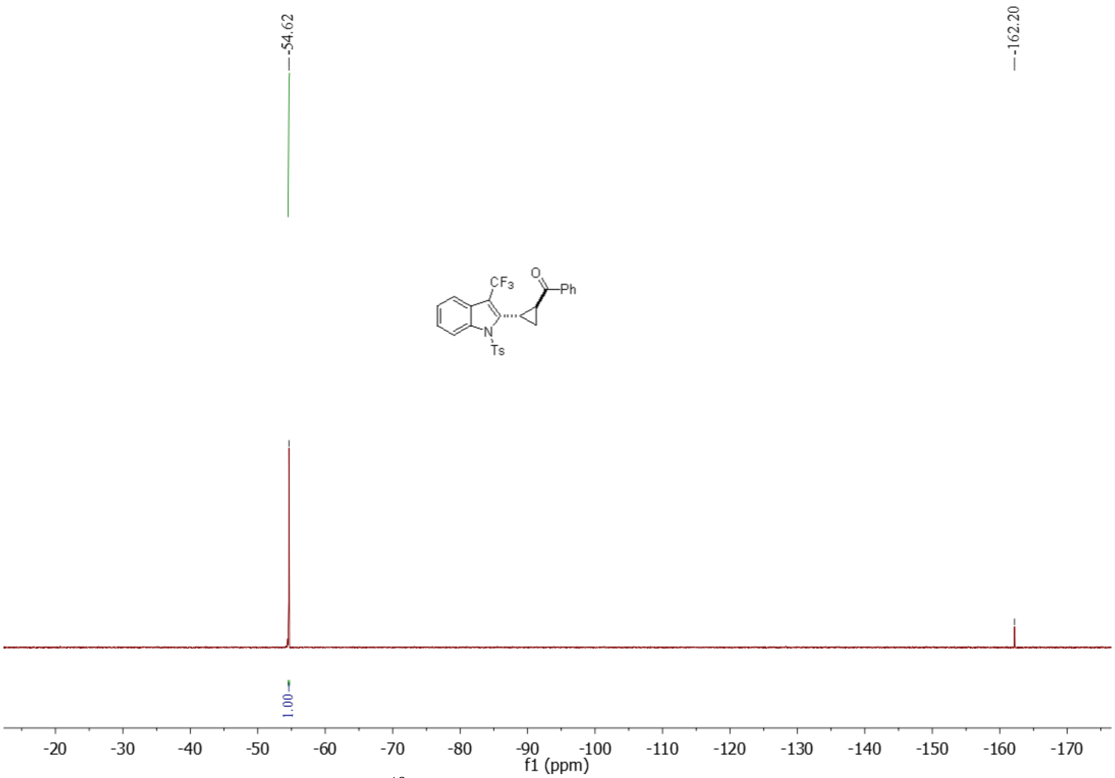

Figure S154. ^{19}F NMR spectrum of **9**, related to **Scheme 7**.

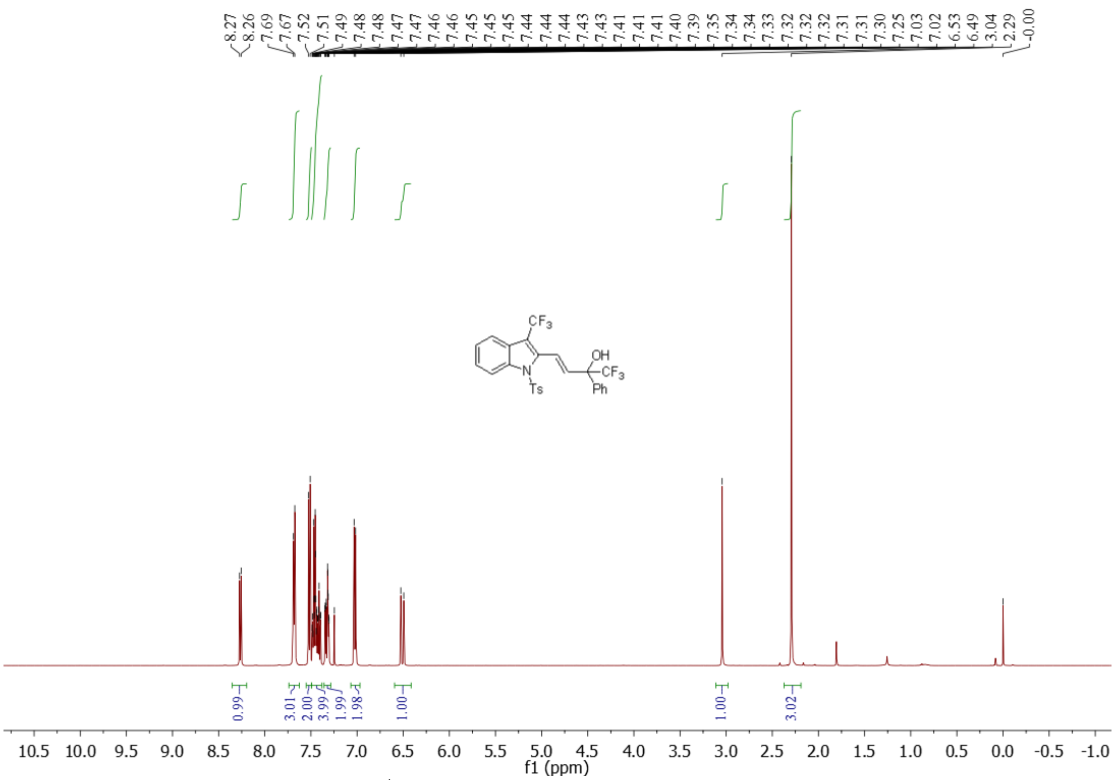

Figure S155. ^{1}H NMR spectrum of **10**, related to **Scheme 7**.

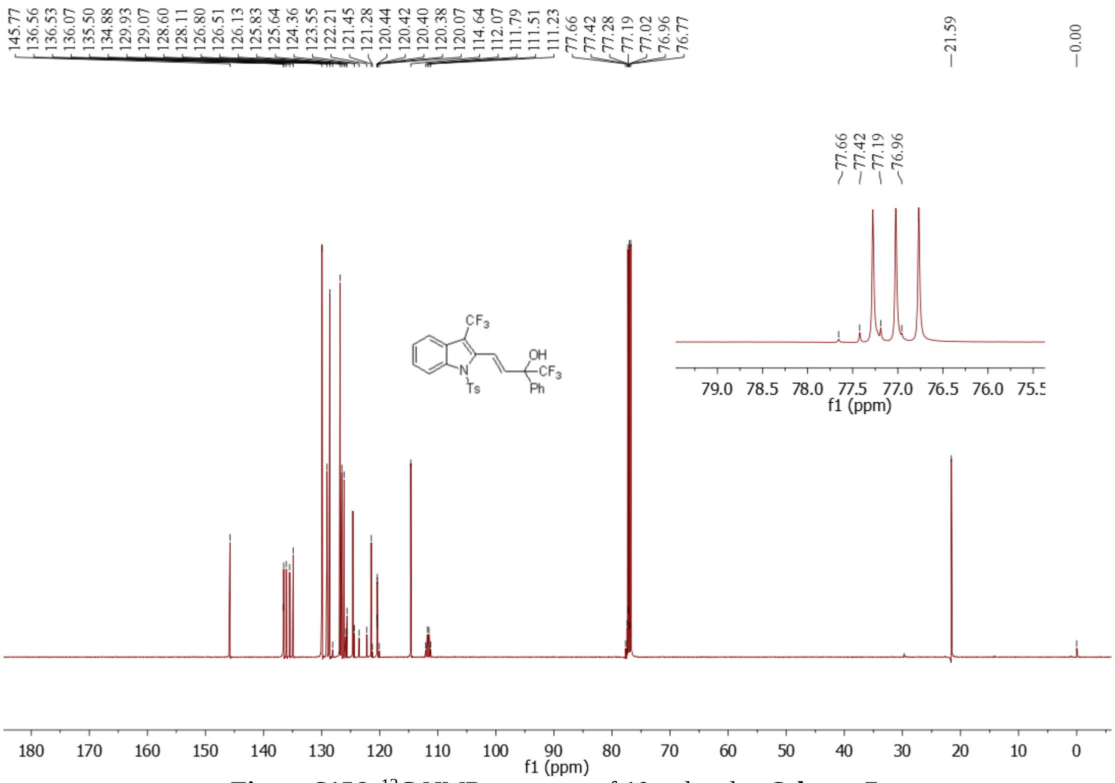

Figure S156. ^{13}C NMR spectrum of **10**, related to **Scheme 7**.

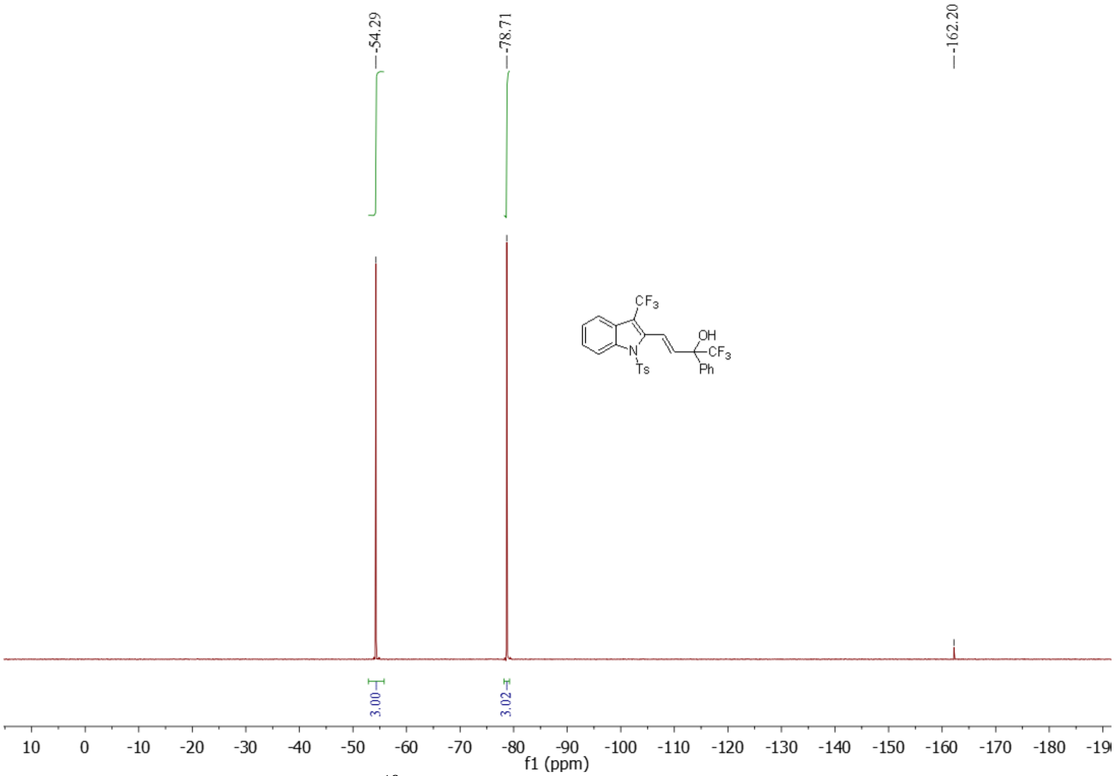

Figure S157. ^{19}F NMR spectrum of **10**, related to **Scheme 7**.

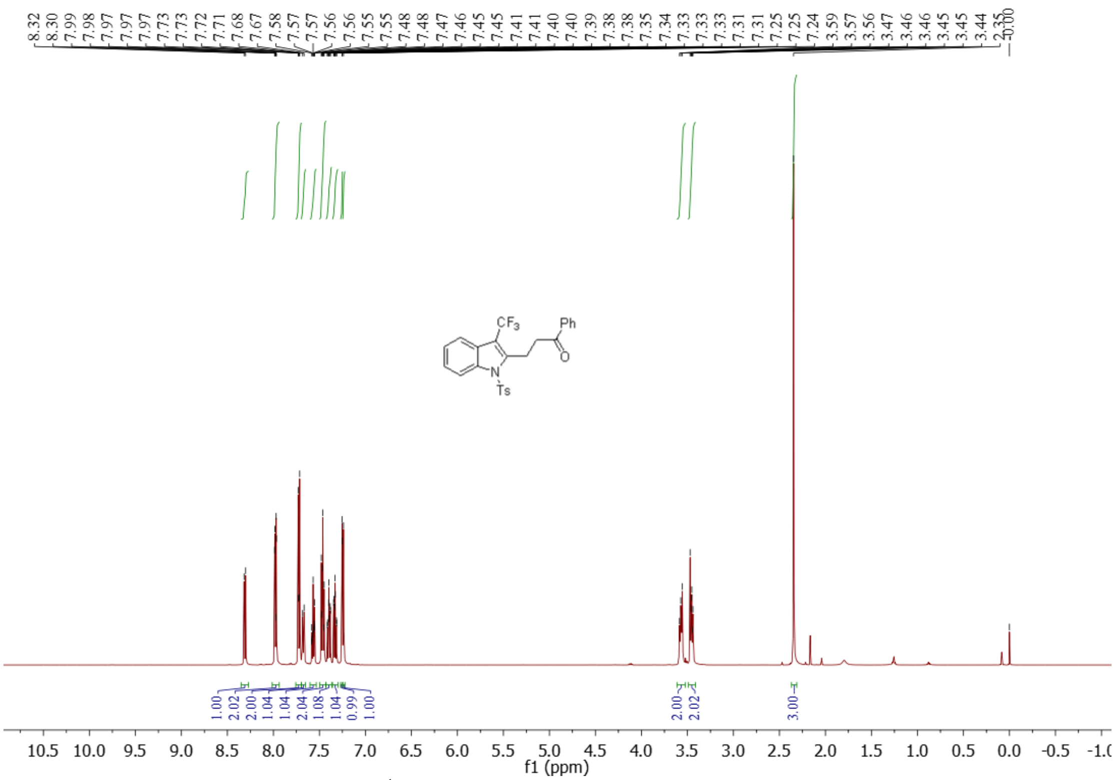

Figure S158. ^{1}H NMR spectrum of **11**, related to **Scheme 7**.

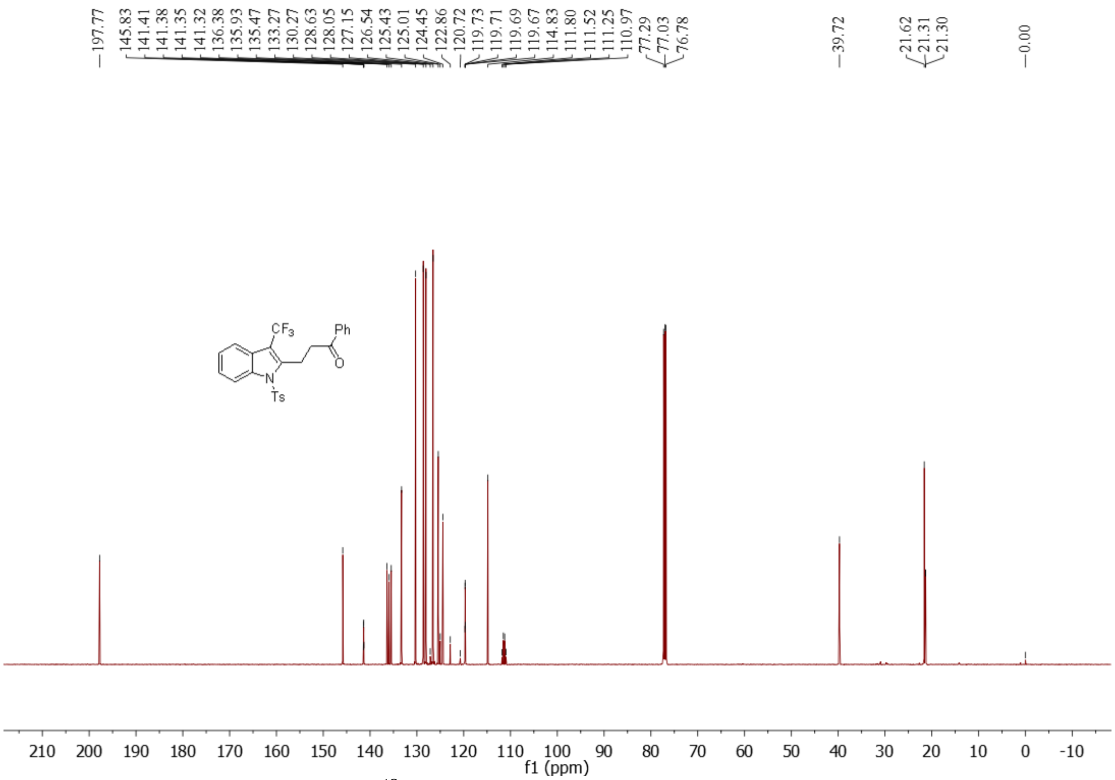

Figure S159. ^{13}C NMR spectrum of **11**, related to **Scheme 7**.

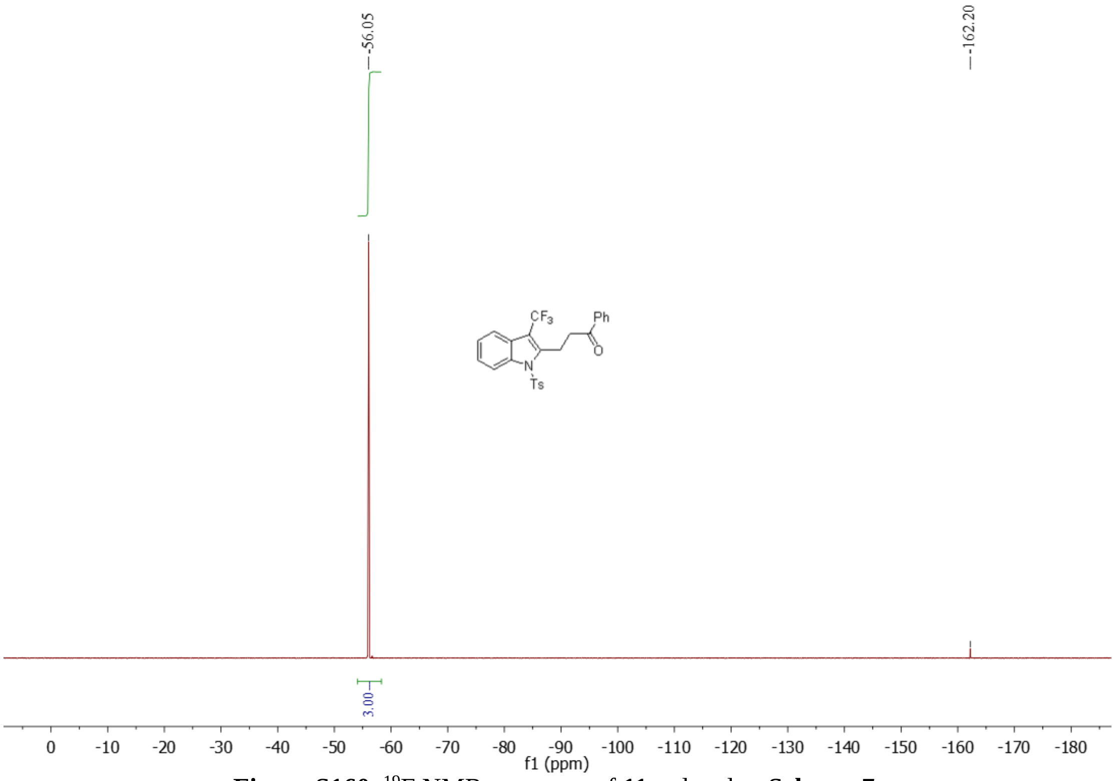

Figure S160. ^{19}F NMR spectrum of **11**, related to **Scheme 7**.

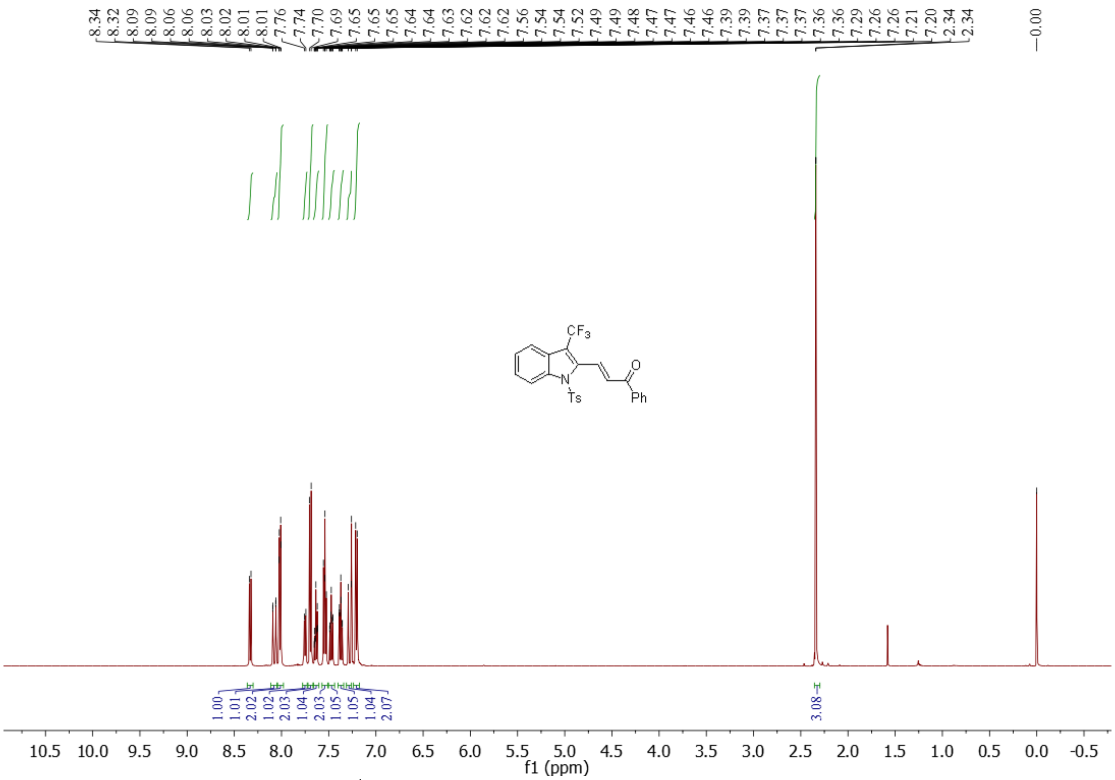

Figure S161. ^{1}H NMR spectrum of **(*E*)-6aa**, related to **Scheme 7**.

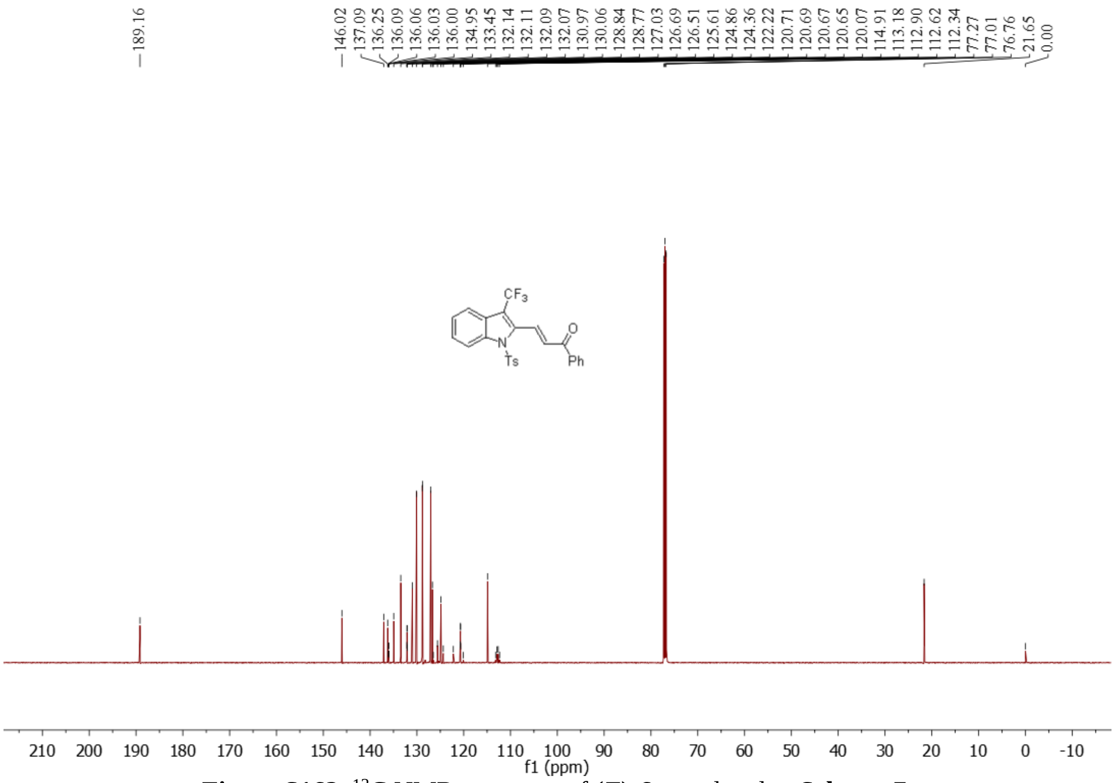

Figure S162. ^{13}C NMR spectrum of **(*E*)-6aa**, related to **Scheme 7**.

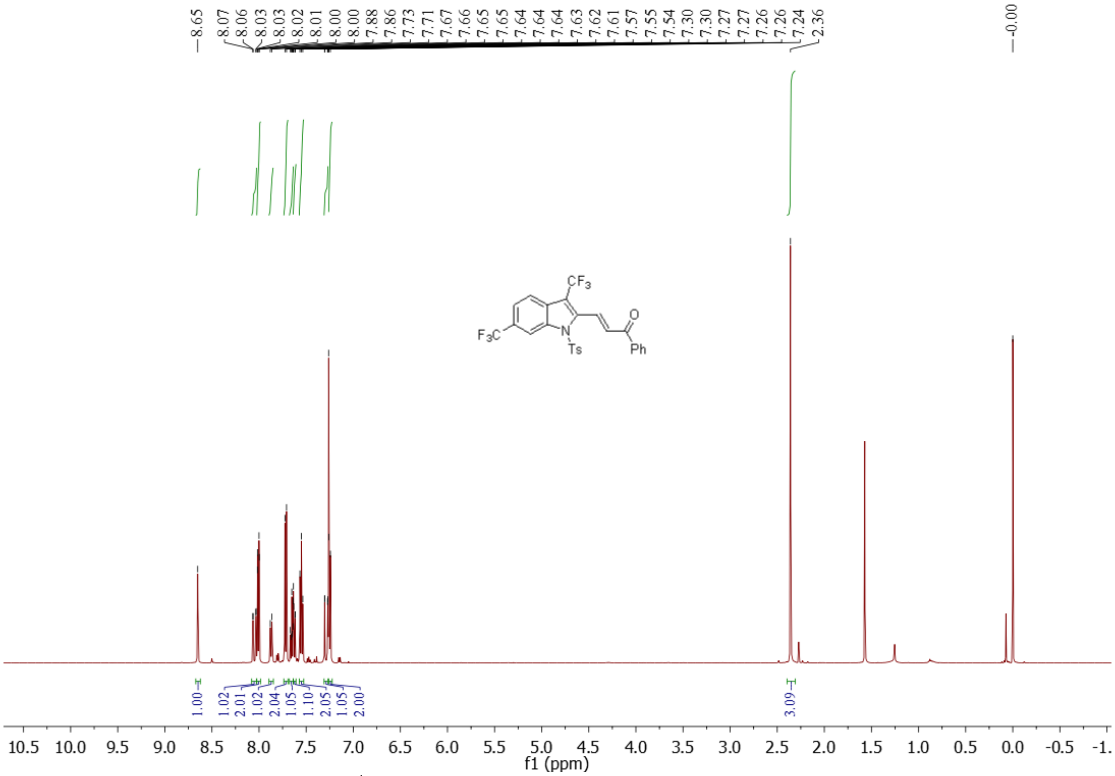

Figure S163. ^{1}H NMR spectrum of ***(E)*-6ca**, related to **Scheme 7**.

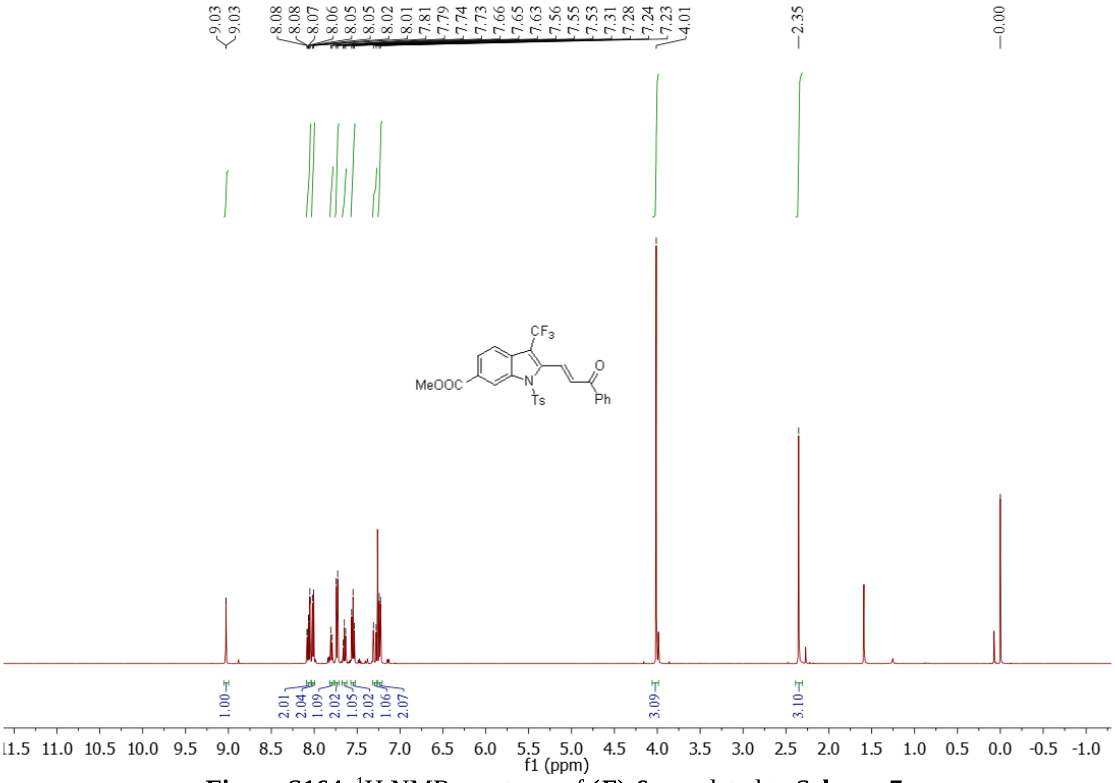

Figure S164. ^{1}H NMR spectrum of ***(E)*-6ga**, related to **Scheme 7**.

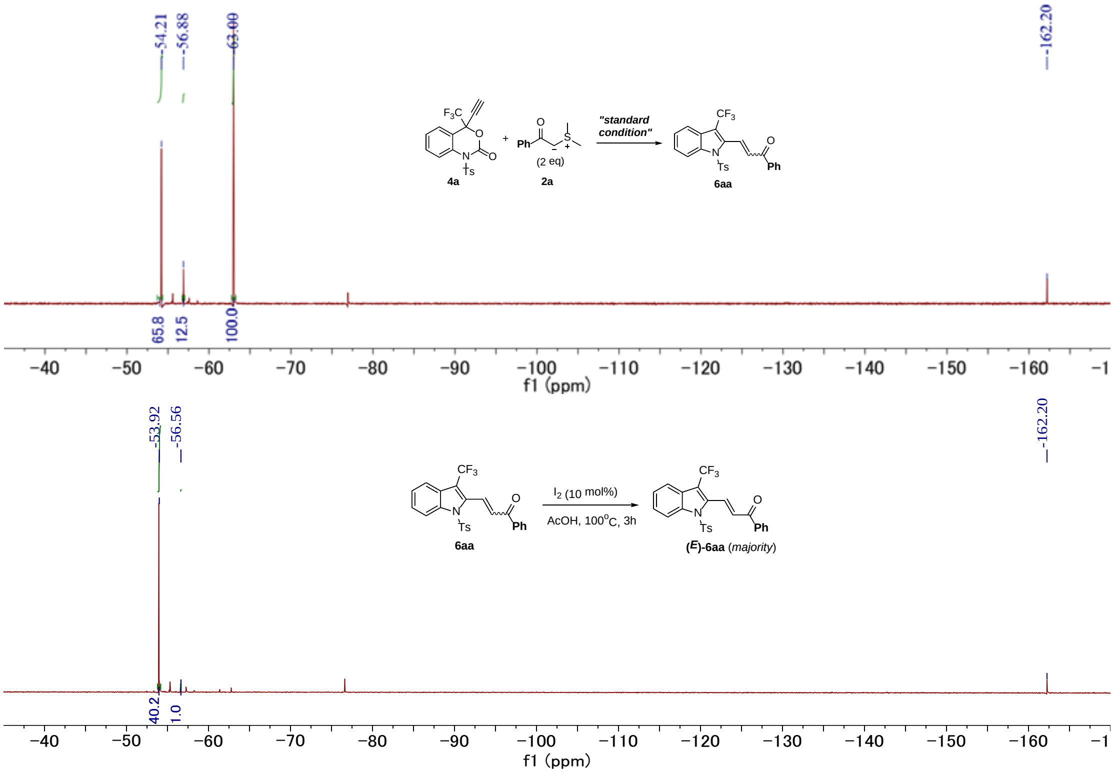

Figure S165. ^{19}F NMR spectrum of **6aa**, related to **Scheme 8**.

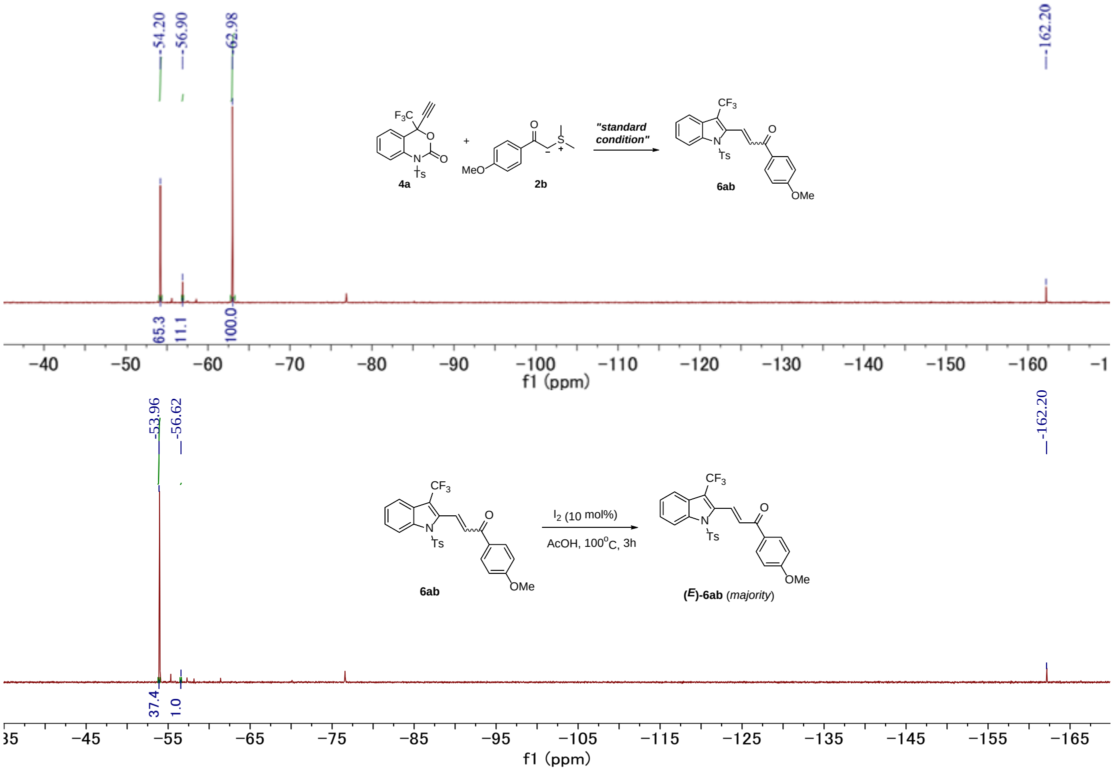

Figure S166. ^{19}F NMR spectrum of **6ab**, related to **Scheme 8**.

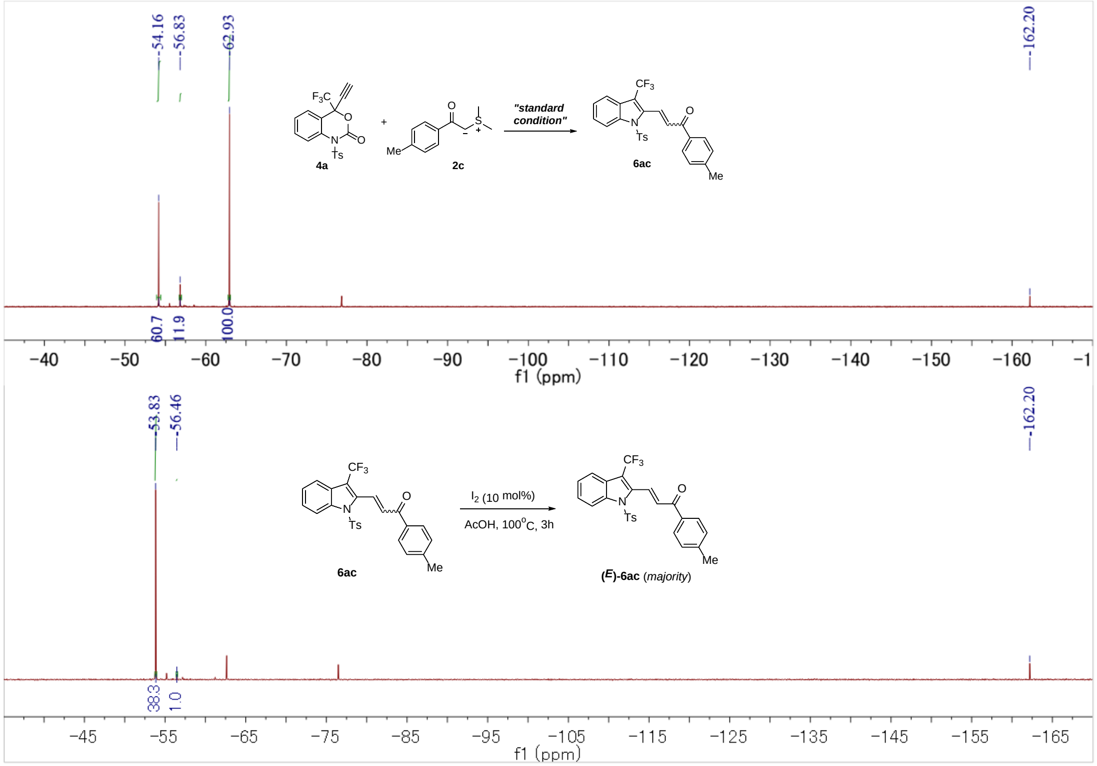

Figure S167. ^{19}F NMR spectrum of **6ac**, related to **Scheme 8**.

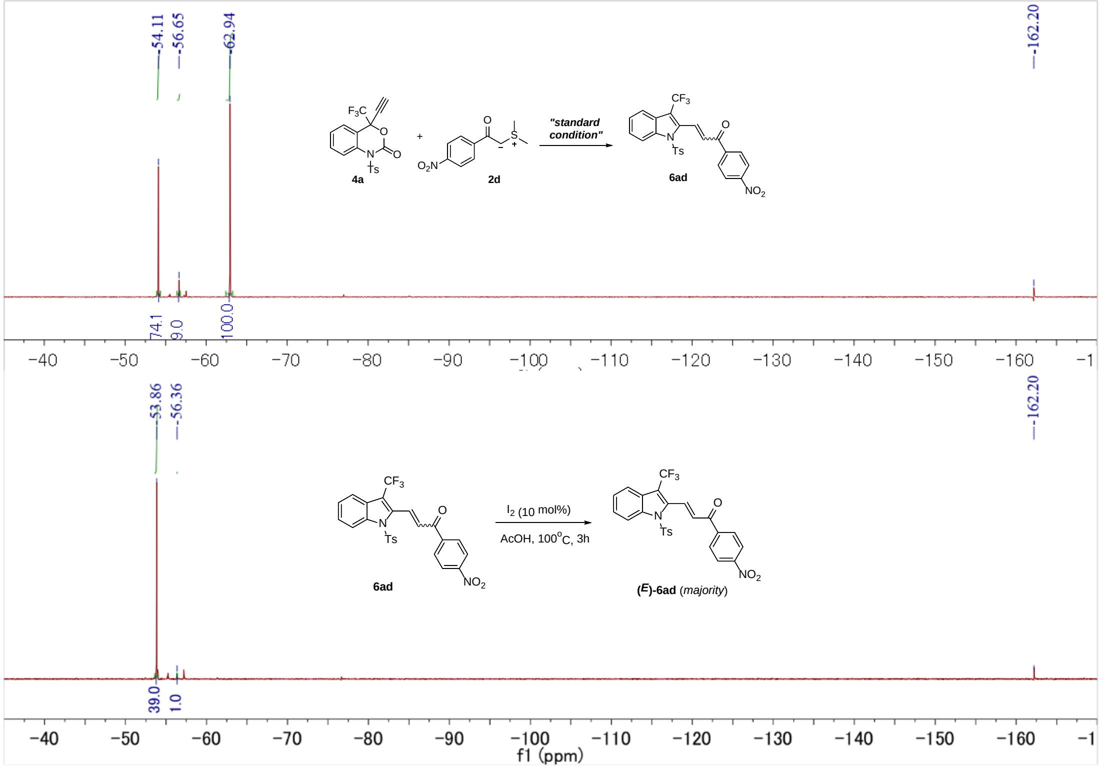

Figure S168. ^{19}F NMR spectrum of **6ad**, related to **Scheme 8**.

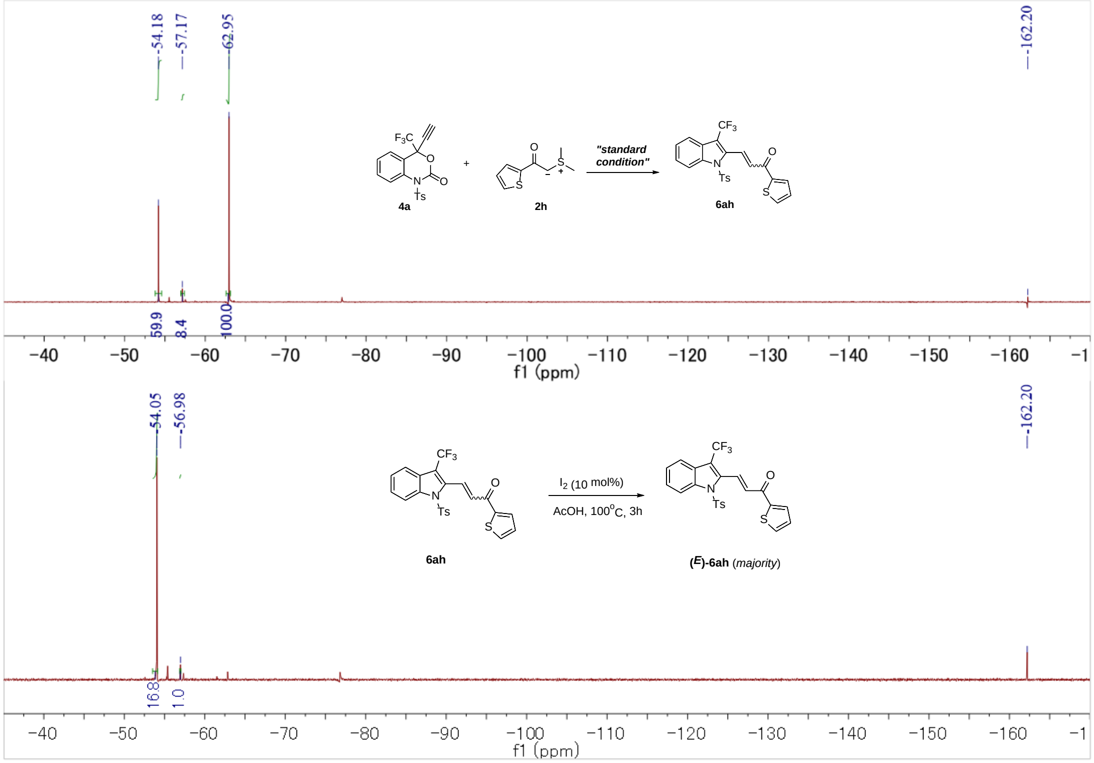

Figure S169. ^{19}F NMR spectrum of **6ah**, related to **Scheme 8**.

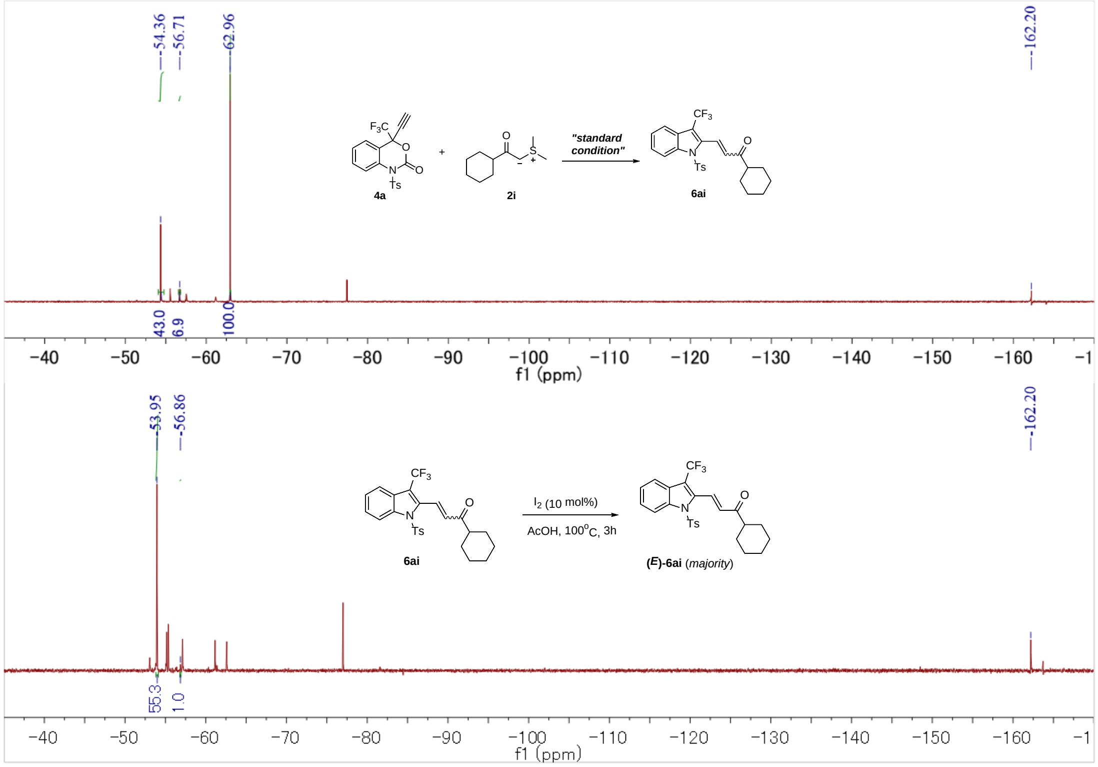

Figure S170. ^{19}F NMR spectrum of **6ai**, related to **Scheme 8**.

Supplemental Table

Table S1. Ligand screening [a], related to **Table 1**

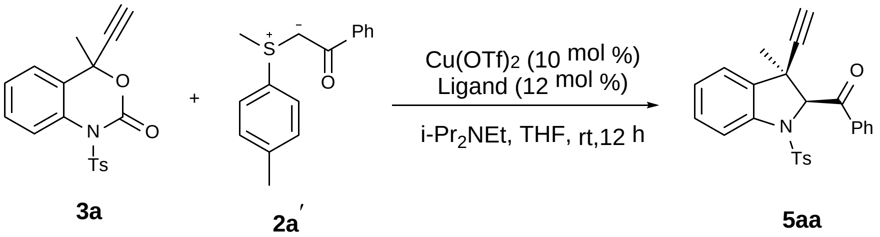

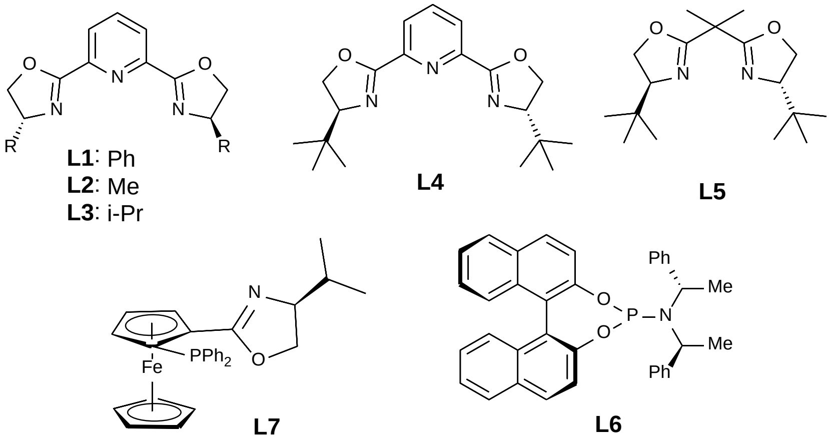

Entry	Ligand	dr[b]	Yield (%)[c]	*ee* (%)[d]
1	**L-1**	>95:5	30	42
2	**L-2**	>95:5	50	56
3	**L-3**	**>95:5**	**72**	**74**
4	**L-4**	>95:5	63	−46
5	**L-5**	>95:5	49	3
6	**L-6**	>95:5	48	−8
7	**L-7**	ND	15	−43
8	**DBFOX/Ph**	>95:5	48	19
9	**(R)-DTBM-SEGPHOS**	ND	<10	−87
10	**(R)-SEGPHOS**	ND	23	−32

[a] Reactions were carried out with **3a** (0.1 mmol), **2a'** (0.2 mmol), $Cu(OTf)_2$ (10 mol %), ligand (12 mol %), *i*-Pr_2NEt (DIPEA, 1.2 equiv.) in THF at room temperature.
[b] Determined by ^{1}H NMR analysis of the reaction mixture.
[c] Determined by ^{1}H NMR analysis of the crude reaction mixture using 1,3,5-trimethoxybenzene as an internal standard.
[d] The *ee* was determined by chiral HPLC analysis.

Table S2. Cu salts screening [a]**,** related to **Table 1**

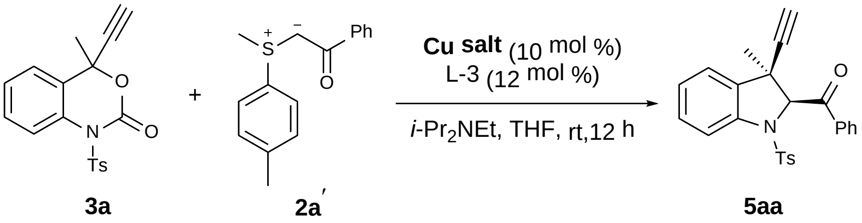

Entry	Copper salt	dr[b]	Yield (%)[c]	*ee* (%)[d]
1	**$Cu(OTf)_2$**	**>95:5**	**72**	**74**
2	CuOTf-Toluene	>95:5	73	29
3	$[(CH_3CN)_4Cu]PF_6$	>95:5	70	57
4	CuBr	>95:5	72	-12
5	CuI	>95:5	69	-0.8
6	$Cu(OAc)_2$	>95:5	51	15

[a] Reactions were carried out with **3a** (0.1 mmol), **2a'** (0.2 mmol), $Cu(OTf)_2$ (10 mol %), ligand (12 mol %), *i*-Pr_2NEt (1.2 equiv.) in THF at room temperature. [b] Determined by ^1H NMR analysis of the reaction mixture. [c] Determined by ^1H NMR analysis of the crude reaction mixture using 1,3,5-trimethoxybenzene as an internal standard. [d] The *ee* was determined by chiral HPLC analysis.

Table S3. Solvent screening[a], related to **Table 1**

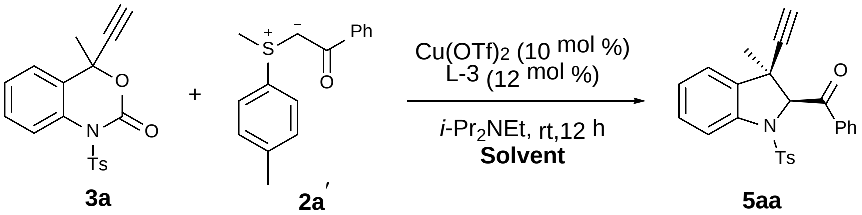

Entry	Solvent	dr[b]	Yield (%)[c]	*ee* (%)[d]
1	THF	>95:5	72	74
2	MeOH	>95:5	36	67
3	Dioxane	>95:5	61	66
4	ACN	>95:5	76	69
5	**DCM**	**>95:5**	**69**	**78**
6	Xylene	>95:5	56	52
7	DMF	>95:5	52	69
8	CPME	>95:5	52	66
9	DCE	>95:5	69	77
10	HFIP	-	NR	-

[a] Reactions were carried out with **3a** (0.1 mmol), **2a'** (0.2 mmol), $Cu(OTf)_2$ (10 mol %), ligand (12 mol %), *i*-Pr_2NEt (1.2 equiv.) in THF at room temperature. [b] Determined by ^1H NMR analysis of the reaction mixture. [c] Determined by ^1H NMR analysis of the crude reaction mixture using 1,3,5-trimethoxybenzene as an internal standard. [d] The *ee* was determined by chiral HPLC analysis.

Table S4. Base screening[a], related to **Table 1**

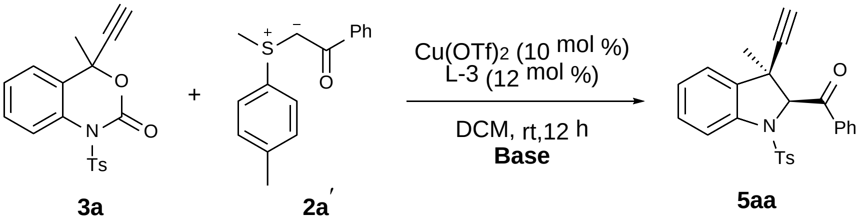

Entry	Base	Ratio	dr[b]	Yield (%)[c]	*ee* (%)[d]
1	DIPEA	1.2	>95:5	69	78
2	TEA	1.2	>95:5	67	75

3	***N*-Ethylmorpholine**	**1.2**	**>95:5**	**84**	**82**
4	DBU	1.2	>95:5	32	21
5	K_2CO_3	1.2	>95:5	67	81
6[e]	-	-	>95:5	69	82
7	*N*-Ethylmorpholine	0.5	>95:5	82	81
8	*N*-Ethylmorpholine	2.0	>95:5	74	81
9	*N*-Ethylmorpholine	3.0	>95:5	62	83

[a] Reactions were carried out with **3a** (0.1 mmol), **2a'** (0.2 mmol), $Cu(OTf)_2$ (10 mol %), ligand (12 mol %), base (1.2 equiv.) in THF at room temperature. [b] Determined by ^{1}H NMR analysis of the reaction mixture. [c] Determined by ^{1}H NMR analysis of the crude reaction mixture using 1,3,5-trimethoxybenzene as an internal standard. [d] The *ee* was determined by chiral HPLC analysis. [e] without base.

Table S5. Sulfide screening[a], related to **Table 1**

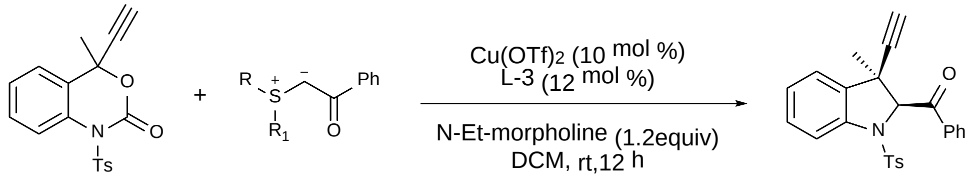

3a **2** **5aa**

Entry	R	R_1	dr[b]	Yield (%)[c]	*ee* (%)[d]
1	Me	Me	>95:5	79	63
2	Me	Ph	>95:5	71	78
3	**Me**	**4-methyl phenyl**	**>95:5**	**84**	**82**
4	Me	4-tertbutyl phenyl	>95:5	78	82
5	Ph	Ph	>95:5	36	61

[a] Reactions were carried out with **3a** (0.1 mmol), **2** (0.2 mmol), $Cu(OTf)_2$ (10 mol %), ligand (12 mol %), *N*-Ethylmorpholine (1.2 equiv.) in THF at room temperature. [b] Determined by ^{1}H NMR analysis of the reaction mixture. [c] Determined by ^{1}H NMR analysis of the crude reaction mixture using 1,3,5-trimethoxybenzene as an internal standard. [d] The *ee* was determined by chiral HPLC analysis.

Table S6. Temperature screening[a], related to **Table 1**

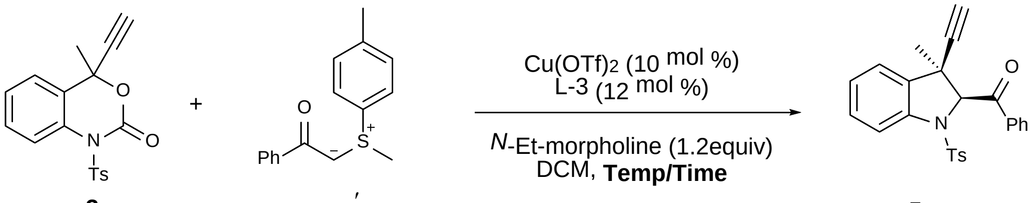

3a **2a'** **5aa**

Entry	Temp (°C)	Time (h)	dr[b]	Yield (%)[c]	*ee* (%)[d]
1	35	2	>95:5	73	80
2	**R.T.**	**12**	**>95:5**	**84**	**82**
3	0	24	>95:5	55	82
4	-10	48	>95:5	64	72
5	-20	90	>95:5	59	38

[a] Reactions were carried out with **3a** (0.1 mmol), **2a'** (0.2 mmol), $Cu(OTf)_2$ (10 mol %), ligand (12 mol %), *N*-Ethylmorpholine (1.2 equiv.) in THF at room temperature. [b] Determined by ^{1}H NMR analysis of the reaction mixture. [c] Determined by ^{1}H NMR analysis of the crude reaction mixture using 1,3,5-trimethoxybenzene as an internal standard. [d] The *ee* was determined by chiral HPLC analysis.

Table S7. Sulfide ratio screening[a], related to **Table 1**

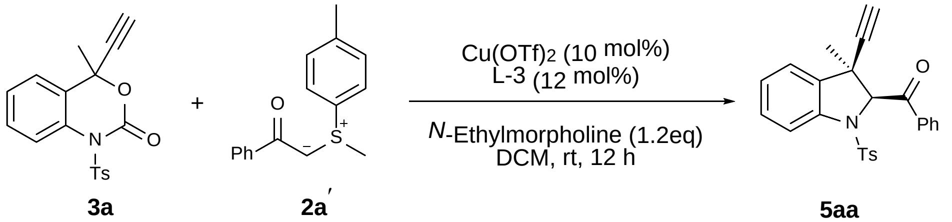

Entry	**2a'** (eq mol)	dr[b]	Yield (%)[c]	*ee* (%)[d]
1	1.2	>95:5	67	83
2	**1.5**	**>95:5**	**83**	**84**
3	2	>95:5	84	82
4	2.5	>95:5	82	80
5	3	>95:5	95	78

[a] Reactions were carried out with **3a** (0.1 mmol), **2a'** (0.2 mmol), $Cu(OTf)_2$ (10 mol %), ligand (12 mol %), *N*-Ethylmorpholine (1.2 equiv.) in THF at room temperature. [b] Determined by ^1H NMR analysis of the reaction mixture. [c]Determined by ^1H NMR analysis of the crude reaction mixture using 1,3,5-trimethoxybenzene as an internal standard. [d] The *ee* was determined by chiral HPLC analysis.

Table S8. Ligand screening[a], related to **Scheme 6**

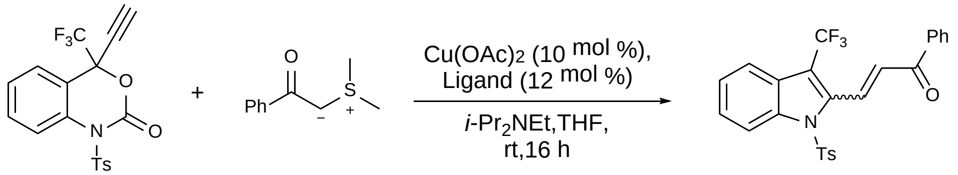

Entry	Ligand	Yield (%)[b]	*E/Z* ratio[b]
**1**	**(R)-BINAP**	**72**	**5.0/1**
2	(R)-Xyl-BINAP	56	3.0/1
3	(R)-SEGPHOS	62	3.4/1
4	(R)-DTBM-SEGPHOS	27	2.0/1
5	DPEPhos	28	6.0/1
6	Dppe	45	2.5/1
7	1,10-Phenanthroline	11	10.0/1
8	**L-1**	33	2.7/1
9	**L-2**	51	3.3/1
10	**L-4**	30	1.5/1
11	**L-6**	17	4.7:1

[a] Reactions were carried out with **4a** (0.1 mmol), **2a** (0.2 mmol), $Cu(OAc)_2$ (10 mol %), ligand (12 mol %), *i*-Pr_2NEt (2.1 equiv.) in THF at room temperature for 16 h. [b] yield and *E/Z* ratio were determined by ^{19}F NMR analysis of the reaction mixture.

Table S9. Conditions screening[a], related to **Scheme 6**

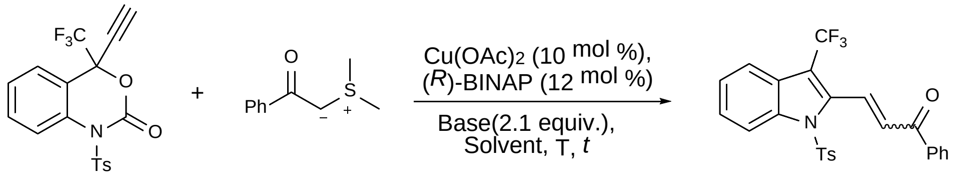

Entry	Ligand	Base	Solvent	*t* (°C)	T(h)	Yield(%)[b]	*E* / *Z*[b]
1	(*R*)-BINAP	DIPEA	THF	rt	12	77	2.6:1
2	(*R*)-BINAP	DIPEA	Dioxane	rt	12	68	2.5:1
3	(*R*)-BINAP	DIPEA	CH_3CN	rt	12	53	2.3:1

4	(*R*)-BINAP	DIPEA	Toluene	rt	12	64	3.1:1
5	(*R*)-BINAP	DIPEA	DMF	rt	12	43	2.0:1
6	(*R*)-BINAP	DIPEA	DCE	rt	12	55	3.3:1
7	(*R*)-BINAP	DIPEA	DCM	rt	12	76	3.9:1
8	(*R*)-BINAP	Cs_2CO_3	DCM	rt	12	31	2.6:1
9	(*R*)-BINAP	DABCO	DCM	rt	12	25	6.2:1
10	(*R*)-BINAP	DMAP	DCM	rt	12	trace	--
11	(*R*)-BINAP	DIPEA	DCM	30	12	75	3.2:1
12	(*R*)-BINAP	DIPEA	DCM	0	12	72	4.0:1
13[c]	(*R*)-BINAP	DIPEA	DCM	rt	2	70	3.5:1
14[d]	(*R*)-BINAP	DIPEA	DCM	rt	2	63	3.8:1
15[e]	(*R*)-BINAP	DIPEA	DCM	rt	2	69	3.9:1
16	(*R*)-BINAP	DIPEA	DCM	rt	2	77(73)	3.6:1
17	*rac*-BINAP	DIPEA	DCM	rt	2	77(75)	3.5:1
18[f]	*rac*-BINAP	DIPEA	DCM	rt	2	81	3.7:1
18[f, g]	***rac*-BINAP**	**DIPEA**	**DCM**	**rt**	**2**	**83**(79)	**3.9:1**

[a] Reactions were carried out with **4a** (0.05 mmol), **2a** (0.1 mmol), $Cu(OAc)_2$ (10 mol %), ligand (12 mol %), base (2.1 equiv.) and solvent (1.0 mL) under corresponding reaction condition. [b] Yield and *E/Z* ratio were determined by ^{19}F NMR analysis of the reaction mixture, in which using $PhCF_3$ as internal standard. [c] 0.075 mmol **2a** were used. [d] 0.2 mmol **4a** were used. [e] 0.5 mL DCM were used. [f] 0.08 mmol DIPEA were used. [g] 0.1 mmol **4a** scale were performed.

Table S10. Single step formation of **6** into predominantly the ***E*** isomer[a], related to **Scheme 8**.

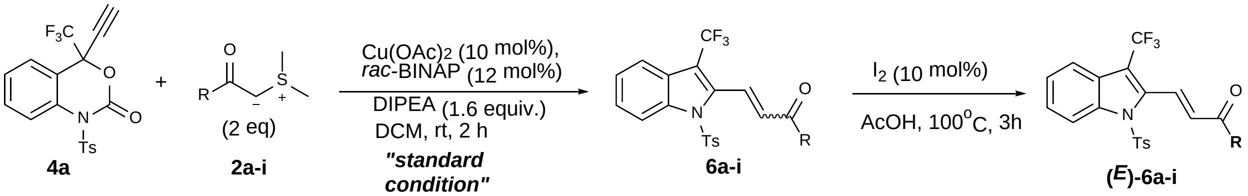

Entry	R	6ax(%)	*E/Z*	(*E*)-6ax / (*Z*)-6ax
**1**	Ph	78	5.4 / 1	40 / 1
**2**	4-OMe-Ph	75	5.9 / 1	37 / 1
**3**	4-Me-Ph	73	5.1 / 1	38 / 1
**4**	4-NO_2-Ph	83	8.2 / 1	39 / 1
**5**	2-Thiophenyl	68	7.1 / 1	17 / 1
**6**	*c*-Hexyl	50	6.2 / 1	55 / 1

[a] Fellow the general method **J**, the crude product **6ax** was then filtered through a short pad of silica, the filtrate was concentrated for the next run. Fellow the literature procedure (Makarov et al., 2018), an oven-dried tube was charged with **6ax**, Iodine (10 mol%) and AcOH. The tube was sealed, and the resulting solution was stirred at 100 °C for 3 h. The resulting solution were then taken ^{19}F NMR to give the corresponding isomer rate. The ^{19}F NMR spectrum were attached below.

Transparent Methods

General Information

All reactions were performed in oven-dried glassware under a positive pressure of nitrogen or argon. Solvents were transferred via syringe and were introduced into the reaction vessels through a rubber septum. All solvents were dried by standard method. All the reactions were monitored by thin-layer chromatography (TLC) carried out on 0.25 mm

Merck silica gel (60-F254). The TLC plates were visualized with UV light. All the reaction products were purified by column chromatography and was carried out on a column packed with silica gel 60N spherical neutral size 50-63 mm. The ^{1}H NMR (300 MHz and 500 MHz) and ^{19}F NMR (282 MHz) spectra as for solution in $CDCl_3$ and DMSO were recorded on a Varian Mercury 300 and BRUKER 500 Ultra Shield TR. ^{13}C NMR (125.8 MHz) spectra for solution in $CDCl_3$ was recorded on a BRUKER 500 Ultra Shield TR. The chemical shifts (δ) are expressed in ppm downfield from internal TMS (δ = 0.00) and coupling constants (*J*) are reported in hertz (Hz). The hexafluorobenzene (C_6F_6) [δ = −162.2 ($CDCl_3$)] was used as internal standard for ^{19}F NMR. The following abbreviations were used to explain the multiplicities: s = singlet, d = doublet, t = triplet, q = quartet, m = multiplet, br = broad. Mass spectra were recorded on a SHIMADZU GCMS-QP5050A (EI-MS) and SHIMADZU LCMS-2020 (ESI-MS). High resolution mass spectrometry (HRMS) was carried out on an electron impact ionization mass spectrometer with a micro-TOF analyzer and recorded on a Waters, GCT Premier (EI-MS) with a TOF analyzer. Infrared spectra were recorded on a JASCO FT/IR-4100 spectrometer. Melting points were recorded on a BUCHI M-565. Optical rotations were measured on a SEPA-300 instrument (HORIBA Ltd, Kyoto, Japan). HPLC analyses were performed on a JASCOLC-2000 Plus series using 4.6 x 250 mm CHIRALPAK series.

Commercially available chemicals were obtained from Aldrich Chemical Co., Alfa Aesar, TCI and used as received unless otherwise noted. Solvents acetonitrile, ethyl acetate, ethanol, Dioxane, DMF, DCM and THF were dried and distilled before use.

Supplemental Experimental Procedures for the synthesis of starting materials.

Synthesis of substituted alkyl ethynyl benzoxazinanones 3, related to Scheme 4.

Overall reaction steps for the synthesis of substituted alkynyl benzoxazinanones **3a** to **3g** is showing below.

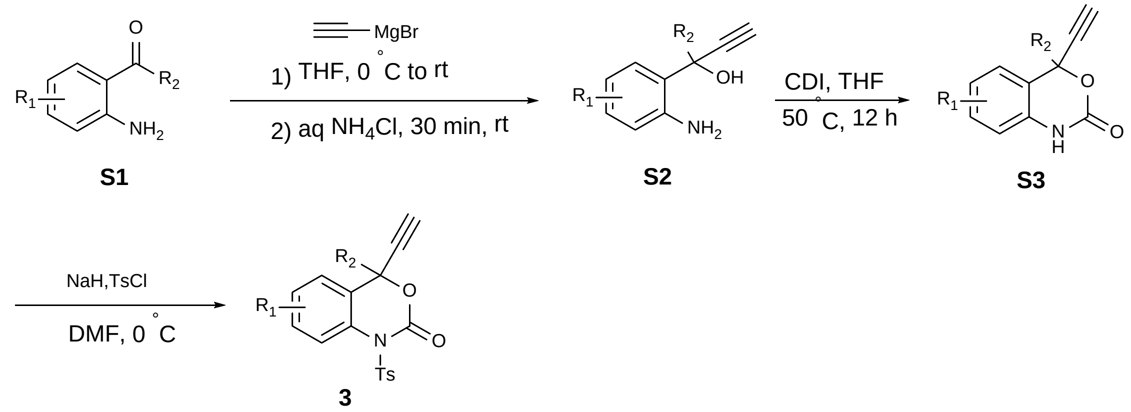

General procedure for the synthesis of substituted 1-(2-aminophenyl) propargyl alcohol derivatives (S2a-S2i) (Method A), related to Scheme 4.

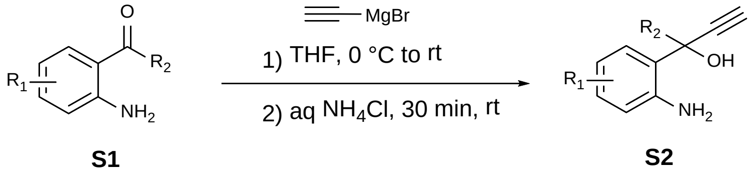

The substituted 1-(2-aminophenyl) ketones **S1** were prepared according to literature procedures (Huang et al., 2012; Xia et al., 2018; Kehler et al., 2013; Kumar et al.; 2015 Song et al., 2019). To a stirred solution of **S1** (1 equiv., 5 mmol) in anhydrous THF (20 mL) was added ethynyl magnesium bromide (40 mL, 0.5 M in THF, 4 equiv., 20 mmol) at 0°C over 30 min. The reaction mixture was allowed to warm to room temperature and stirred at this temperature overnight. When the reaction was completed as determined by TLC, the reaction mixture was quenched with saturated aqueous NH_4Cl and then extracted with EtOAc. The organic phase was washed by brine, dried over Na_2SO_4 and concentrated in vacuo. Purification of the residue by column chromatography gave the corresponding **S2**. The characterization data of **S2** are summarized below. The characterization data of 2-(2-aminophenyl)but-3-yn-2-ol (**S2a**), 2-(2-Amino-5-bromophenyl)but-3-yn-2-ol (**S2f**), 3-(2-aminophenyl)-4-methylpent-1-yn-3-ol **(S2h)** and 1-(2-aminophenyl)-1-phenylprop-2-yn-1-ol **(S2i)** were matched with reported data in literature.

2-(2-Amino-4-fluorophenyl)but-3-yn-2-ol (S2b):

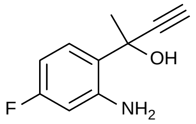

Following the general method **A**, compound **S2b** was obtained as a pale yellow solid (0.59 g, Yield: 66%), m.p. = 65.1 – 65.7 °C. **^{1}H NMR** (500 MHz, $CDCl_3$) δ 7.43 (dd, *J* = 8.7, 6.4 Hz, 1H), 6.42 (ddd, *J* = 11.0, 7.4, 3.2 Hz, 1H), 6.35 (dd, *J* = 10.5, 2.6 Hz, 1H). 4.59 (br s, 2H), 3.14 (br s, 1H), 2.72 (s, 1H), 1.87 (s, 3H). **^{13}C NMR** (126 MHz, $CDCl_3$) δ 163.3 (d, *J* = 244.6 Hz), 146.3 (d, *J* = 10.8 Hz), 128.1, 123.2, 104.5 (d, *J* = 21.3 Hz), 104.1 (d, *J* = 24.4 Hz), 86.6, 73.6, 70.1, 28.7. **^{19}F NMR** (282 MHz, $CDCl_3$) δ −114.7 – −115.1 (m, 1F). **IR** (KBr): 3477, 3271, 2111, 1616, 1502, 1168, 1093, 975, 846, 655 cm^{-1}. **HRMS (EI)** calculated for $C_{10}H_{10}FNO$ $[M]^+$: 179.0746, found: 179.0750.

2-(2-Amino-4-(trifluoromethyl)phenyl)but-3-yn-2-ol (S2c):

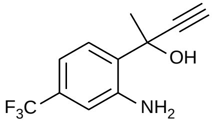

Following the general method **A**, compound **S2c** was obtained as a pale yellow solid (0.56 g, Yield: 49%), m.p. = 73.5 – 74.1 °C. **^{1}H NMR** (500 MHz, $CDCl_3$) δ 7.64 – 7.58 (m, 1H), 7.00 – 6.95 (m, 1H), 6.92 – 6.87 (m, 1H), 4.69 (br s, 2H), 3.03 (br s, 1H), 2.77 (s, 1H), 1.90 (s, 3H). **^{13}C NMR** (126 MHz, $CDCl_3$) δ 144.8, 131.2 (q, *J* = 32.2 Hz), 130.2, 127.1, 124.0 (q, *J* = 272.2 Hz), 114.6 (q, *J* = 3.8 Hz), 114.1 (q, *J* = 3.8 Hz), 86.0, 74.2, 70.3, 28.3. **^{19}F NMR** (282 MHz, $CDCl_3$) δ −63.44 (s, 3F). **IR** (KBr): 3411, 3378, 3299, 1621, 1587, 1428, 1336, 1128, 1085, 889 cm^{-1}. **HRMS (EI)** calculated for $C_{11}H_{10}F_3NO$ $[M]^+$: 229.0714, found: 229.0723.

2-(2-Amino-5-chlorophenyl)but-3-yn-21-ol (S2d):

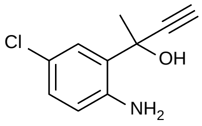

Following the general method **A**, compound **S2d** was obtained as a pale yellow solid (0.86 g, Yield: 73%), m.p. = 91.1 – 92.6 °C. **^{1}H NMR** (500 MHz, $CDCl_3$) δ 7.46 (d, *J* = 2.5 Hz, 1H), 7.06 (dd, *J* = 8.4, 2.4 Hz, 1H), 6.60 (d, *J* = 8.5 Hz, 1H), 4.38 (br s, 2H), 3.46 (br s, 1H), 2.74 (s, 1H), 1.87 (s, 3H). **^{13}C NMR** (126 MHz, $CDCl_3$) δ 142.81, 128.99, 128.73, 126.35, 123.16, 119.01, 86.07, 73.87, 69.82, 28.26. **IR** (KBr): 3370, 3303, 1610, 1486, 1228, 1051, 879, 723, 651 cm□1. **HRMS (EI)** calculated for $C_{10}H_{10}NOCl$ $[M]^+$: 195.0451, found: 195.0458.

2-(2-Amino-4-methylphenyl)but-3-yn-2-ol (S2e):

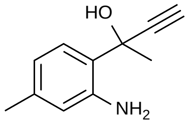

Following the general method **A**, compound **S2e** was obtained as a pale yellow solid (0.69 g, Yield: 79%), m.p. = 72.8 – 73.3 °C. **^{1}H NMR** (500 MHz, $CDCl_3$) δ 7.34 (d, *J* = 7.9 Hz, 1H), 6.61 – 6.56 (m, 1H), 6.52 – 6.49 (m, 1H), 4.31 (br s, 2H), 3.67 (br s, 1H), 2.68 (s, 1H), 2.25 (s, 3H), 1.88 (s, 3H). **^{13}C NMR** (126 MHz, $CDCl_3$) δ 144.0, 139.0, 126.2, 125.4, 119.5, 118.8, 87.0, 72.9, 69.7, 28.4, 20.9. **IR** (KBr): 3374, 3257, 3131, 2354, 1617, 1575, 1419, 1079, 889 cm^{-1}. **HRMS (ESI)** calculated for $C_{11}H_{13}NONa$ $[M+Na]^+$: 198.0895, found: 198.0898.

3-(2-Aminophenyl)pent-1-yn-3-ol (S2g):

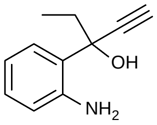

Following the general method **A**, compound **S2g** was obtained as a red oil (0.75 g, Yield: 86%). **^{1}H NMR** (500 MHz, $CDCl_3$) δ 7.52 (dd, *J* = 7.8, 1.6 Hz, 1H), 7.13 – 7.07 (m, 1H), 6.76 – 6.71 (m, 1H), 6.65 (dd, *J* = 7.9, 1.2 Hz, 1H), 4.46 (br s, 2H), 3.10 (br s, 1H), 2.75 (s, 1H), 2.25 – 2.08 (m, 2H), 1.01 (t, *J* = 7.4 Hz, 3H). **^{13}C NMR** (126 MHz, $CDCl_3$) δ 144.5, 128.9, 127.8, 126.1, 117.9, 117.7, 85.6, 75.2, 74.9, 32.5, 9.2. **IR** (KBr): 3374, 3295, 2973, 1614, 1492, 1454, 1095, 754, 640 cm^{-1}. **HRMS (ESI)** calculated for $C_{11}H_{13}NONa$ $[M+Na]^+$: 198.0895, found: 198.0896.

General procedure for the synthesis of substituted 4-ethynyl-4-alkyl-1*H*-benzo[*d*][1,3]oxazin-2(4*H*)-one (S3a-S3i) (Method B), related to Scheme 4.

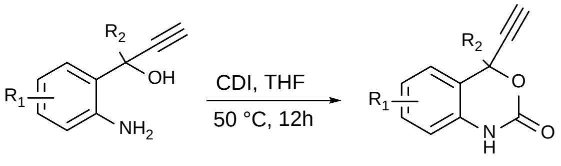

S2 **S3**

In a flame dried 50 mL round bottom flask, alcohol **S2** (3 mmol, 1 equiv.) and 12 mL dry THF was added. To this suspension carbonyldiimidazole (CDI) (6 mmol, 0.973 g, 2.0 equiv.) was added in one portion and the mixture was heated to 50 °C overnight. Completion of the reaction was monitored by TLC, then solvent was removed under

reduced pressure. To the residue, water was added slowly and followed by extraction with ethyl acetate (3 X 30 mL). Combined organic layers were finally washed with brine solution, dried over anhydrous Na_2SO_4 and then solvent was removed under reduced pressure. The crude product was purified by flash column chromatography ((Hexane/Ethyl Acetate = 9:1)) to obtain the pure product **S3**. The characterization data of **S3** are summarized below.

4-Ethynyl-4-methyl-1*H*-benzo[*d*][1,3]oxazin-2(4*H*)-one (S3a):

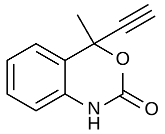

Following the general method **B**, compound **S3a** was obtained as a white solid (0.45 g, Yield: 80%), m.p. = 170.8 – 171.3 °C. **^{1}H NMR** (500 MHz, $CDCl_3$) δ 9.71 (s, 1H), 7.35-7.31 (m, 1H), 7.30 – 7.25 (m, 1H), 7.12 – 7.05 (m, 1H), 6.98 – 6.93 (m, 1H), 2.75 (s, 1H), 1.99 (s, 3H). **^{13}C NMR** (126 MHz, $CDCl_3$) δ 151.6, 134.4, 129.7, 123.8, 123.5, 123.0, 114.9, 82.2, 76.2, 75.1, 28.0. **IR (KBr)**: 3243, 3098, 2129, 1706, 1681, 1357, 1047, 756 cm^{-1}. **HRMS (ESI)** calculated for $C_{11}H_9NO_2Na$ $[M+Na]^+$: 210.0531, found: 210.0534.

4-Ethynyl-7-fluoro-4-methyl-1*H*-benzo[*d*][1,3]oxazin-2(4*H*)-one (S3b):

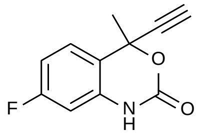

Following the general method **B**, compound **S3b** was obtained as a white solid (0.39 g, Yield: 63%), m.p. = 171.1 – 173.4 °C. **^{1}H NMR** (500 MHz, $CDCl_3$) δ 9.01 (s, 1H), 7.31 (dd, *J* = 8.6, 5.5 Hz, 1H), 6.81 (td, *J* = 8.5, 2.5 Hz, 1H), 6.65 (dd, *J* = 8.9, 2.4 Hz, 1H), 2.76 (s, 1H), 2.01 (s, 3H). **^{13}C NMR** (126 MHz, $CDCl_3$) δ 163.3 (d, *J* = 248.5 Hz), 151.6, 135.6 (d, *J* = 11.1 Hz), 125.7 (d, *J* = 9.8 Hz), 119.0, 110.7 (d, *J* = 22.2 Hz), 102.4 (d, *J* = 26.2 Hz), 81.6, 76.4, 75.5, 28.0. **^{19}F NMR** (282 MHz, $CDCl_3$) δ −111.95 – −111.17 (m, 1F). **IR (KBr)**: 3237, 3091, 2115, 1716, 1614, 1355, 1062, 850 cm^{-1}. **HRMS (ESI)** calculated for $C_{11}H_8FNO_2Na$ $[M+Na]^+$: 228.0437, found: 228.0437.

4-Ethynyl-4-methyl-7-(trifluoromethyl)-1*H*-benzo[*d*][1,3]oxazin-2(4*H*)-one (S3c):

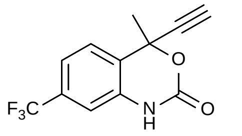

Following the general method **B**, compound **S3c** was obtained as a white solid (0.436 g, Yield: 57%), m.p. = 126.4 – 127.0 °C. **^{1}H NMR** (500 MHz, $CDCl_3$) δ 9.42 (s, 1H), 7.50 – 7.47 (m, 1H), 7.42 – 7.36 (m, 1H), 7.18 – 7.16 (m, 1H), 2.80 (s, 1H), 2.05 (s, 3H). **^{13}C NMR** (126 MHz, $CDCl_3$) δ 151.6, 132.4 (q, *J* = 33.2 Hz), 126.5, 124.8, 123.3 (q, *J* = 272.6 Hz), 120.7 (q, *J* = 3.8 Hz), 112.0 (q, *J* = 3.8 Hz), 81.0, 76.4, 76.2, 27.9. **^{19}F NMR** (282 MHz, $CDCl_3$) δ −63.41 (s, 3F). **IR (KBr)**: 3241, 3151, 2111, 1720, 1602, 1407, 1166, 1135, 877cm^{-1}. **HRMS (ESI)** calculated for $C_{12}H_8F_3NO_2Na$ $[M+Na]^+$: 278.0405, found: 278.0414.

6-Chloro-4-ethynyl-4-methyl-1*H*-benzo[*d*][1,3]oxazin-2(4*H*)-one (S3d):

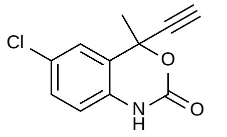

Following the general method **B**, compound **S3d** was obtained as a white solid (0.46 g, Yield: 69%), m.p. = 194.8 – 195.8 °C. **^{1}H NMR** (500 MHz, $CDCl_3$) δ 8.83 (s, 1H), 7.35 – 7.32 (m, 1H), 7.30 – 7.26 (m, 1H), 6.84 (d, *J* = 8.4 Hz, 1H), 2.78 (s, 1H), 2.00 (s, 3H). **^{13}C NMR** (126 MHz, $CDCl_3$) δ 151.3, 132.6, 129.9, 129.0, 124.7, 124.3, 116.1, 81.2, 76.2, 75.9, 27.9. **IR (KBr)**: 3232, 3092, 2129, 1702, 1677, 1355, 1047, 734 cm^{-1}. **HRMS (ESI)** calculated for $C_{11}H_8NO_2ClNa$ $[M+Na]^+$: 244.0141, found: 244.0139.

4-Ethynyl-4,7-dimethyl-1*H*-benzo[*d*][1,3]oxazin-2(4*H*)-one (S3e):

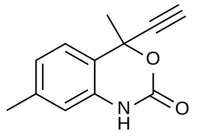

Following the general method **B**, compound **S3e** was obtained as a white solid (0.55 g, Yield: 91%), m.p. = 176.9 – 179.3 °C. **^{1}H NMR** (500 MHz, $CDCl_3$) δ 9.55 (s, 1H), 7.23 – 7.18 (m, 1H), 6.94 – 6.89 (m, 1H), 6.78 – 6.75 (m, 1H), 2.73 (s, 1H), 2.32 (s, 3H), 2.00 (s, 3H). **^{13}C NMR** (126 MHz, $CDCl_3$) δ 152.6, 140.3, 133.8, 124.6, 123.7, 120.2, 115.5, 82.2, 76.6, 75.1, 28.0, 21.1. **IR (KBr)**: 3239, 3004, 2107, 1718, 1596, 1349, 1064, 1022, 765 cm^{-1}. **HRMS (ESI)** calculated for $C_{12}H_{11}NO_2Na$ $[M+Na]^+$: 224.0687, found: 224.0688.

6-Bromo-4-ethynyl-4-methyl-1*H*-benzo[*d*][1,3]oxazin-2(4*H*)-one (S3f):

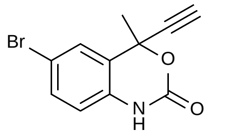

Following the general method **B**, compound **S3f** was obtained as a white solid (0.596 g, Yield: 75%), m.p. = 187.4 – 188.7 °C. **^{1}H NMR** (500 MHz, $CDCl_3$) δ 9.07 (s, 1H), 7.49 – 7.46 (m, 1H), 7.44 – 7.41 (m, 1H), 6.80 (d, *J* = 8.4 Hz, 1H), 2.78 (s, 1H), 2.00 (s, 3H). **^{13}C NMR** (126 MHz, $CDCl_3$) δ 151.5, 133.0, 132.8, 127.0, 124.9, 116.5, 116.2, 81.2, 76.1, 75.9, 27.9. **IR (KBr)**: 3232, 3092, 2129, 1702, 1677, 1348, 1049, 817 cm^{-1}. **HRMS (ESI)** calculated for $C_{11}H_8NO_2BrNa$ $[M+Na]^+$: 287.9636, found: 287.9641.

4-Ethyl-4-ethynyl-1*H*-benzo[*d*][1,3]oxazin-2(4*H*)-one (S3g):

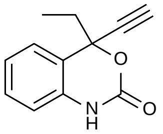

Following the general method **B**, compound **S3g** was obtained as a white solid (0.42 g, Yield: 69%), m.p. = 94.4 – 95.2 °C. **^{1}H NMR** (500 MHz, $CDCl_3$) δ 9.66 (s, 1H), 7.36 – 7.25 (m, 1H), 7.10 (td, *J* = 7.6, 1.0 Hz, 1H), 6.97 – 6.91 (m, 1H), 2.78 (s, 1H), 2.30 – 2.14 (m, 2H), 1.12 (t, *J* = 7.3 Hz, 3H). **^{13}C NMR** (126 MHz, CDCl3) δ 152.3, 134.1, 129.7, 124.7, 123.6, 121.5, 115.0, 81.0, 80.9, 76.1, 34.0, 8.1. **IR (KBr)**: 3270, 3102, 2103, 1720, 1596, 1357, 1070, 761, 657 cm^{-1}. **HRMS (ESI)** calculated for $C_{12}H_{11}NO_2Na$ [M+Na]$^+$: 224.0687, found: 224.0684.

4-Ethynyl-4-isopropyl-1,4-dihydro-2*H*-benzo[*d*][1,3]oxazin-2-one (S3h):

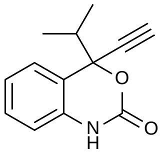

Following the general method **B**, compound **S3h** was obtained as a white solid (0.65 g, Yield: 92%), m.p. = 118.6 – 119.2 °C. **^{1}H NMR** (500 MHz, $CDCl_3$) δ 9.17 (s, 1H), 7.38 – 7.33 (m, 1H), 7.31 – 7.25 (m, 1H), 7.09 (td, *J* = 7.6, 1.1 Hz, 1H), 6.89 (dd, *J* = 7.9, 1.1 Hz, 1H), 2.80 (s, 1H), 2.39 (hept, *J* = 6.7 Hz, 1H), 1.14 (d, *J* = 6.7 Hz, 3H), 1.07 (d, *J* = 6.7 Hz, 3H). **^{13}C NMR** (126 MHz, CDCl3) δ 151.8, 134.0, 129.6, 126.0, 123.2, 121.0, 114.7, 84.8, 79.8, 76.9, 37.3, 17.4, 16.5. **IR (KBr)**: 3239, 3104, 2979, 1708, 1598, 1496, 1351, 1259, 1027, 759 cm^{-1}. **HRMS (ESI)** calculated for $C_{13}H_{13}NO_2Na$ [M+Na]$^+$: 238.0844, found: 238.0849.

4-Ethynyl-4-phenyl-1,4-dihydro-2*H*-benzo[*d*][1,3]oxazin-2-one (S3i):

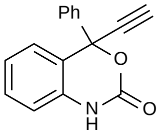

Following the general method **B**, compound **S3i** was obtained as a white solid (0.41 g, Yield: 76%), m.p. = 160.4 – 161.6 °C. **^{1}H NMR** (500 MHz, $CDCl_3$) δ 9.32 (s, 1H), 7.58 – 7.52 (m, 2H), 7.42 – 7.37 (m, 3H), 7.34 – 7.29 (m, 1H), 7.11 – 7.03 (m, 2H), 6.98 – 6.93 (m, 1H), 3.01 (s, 1H). **^{13}C NMR** (126 MHz, CDCl3) δ 151.9, 138.7, 134.5, 130.1, 129.4, 128.5, 127.0, 126.2, 123.6, 122.5, 114.9, 81.1, 80.6, 78.7. **IR (KBr)**: 3288, 3091, 2925, 1720, 1600, 1492, 1344, 1006, 754, 646 cm^{-1}. **HRMS (ESI)** calculated for $C_{16}H_{11}NO_2Na$ [M+Na]$^+$: 272.0682, found: 272.0685.

General experimental procedure for the synthesis of substituted ethynyl benzaxinanones (3a-3i) (Method C), related to Scheme 4.

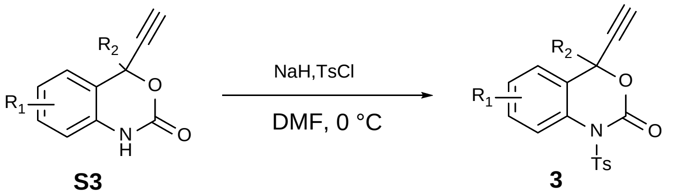

S3 **3**

In a flame dried 100 mL round bottom flask, compound **S3** (2 mmol, 1.0 equiv.) was suspended in dry DMF (6 mL) and allowed to cool to 0 °C. To this solution NaH (60% dispersion in mineral oil, 3 mmol, 0.12 g, 1.5 equiv.) was added and the mixture was allowed to stir for 30 min under N_2 atmosphere. After 30 min, the solution of *p*-toluenesulfonyl chloride (0.419 g, 2.2 mmol, 1.1 equiv.) in dry DMF (3 mL) was added dropwise to the reaction mixture and stirred the reaction mixture at 0 °C until completion of the reaction. After that, the reaction mixture was poured into crushed ice followed by extraction with ethyl acetate (3 X 30 mL). Combined organic layers were finally washed with brine solution, dried over anhydrous Na_2SO_4 and then solvent was removed under reduced pressure. The crude product was purified by flash column chromatography (Hexane/Ethyl Acetate = 9:1) to obtain the pure product **3**. The characterization data of **3** are summarized below. The characterization data of 4-ethynyl-4-methyl-1-tosyl-1*H*-benzo[*d*][1,3]oxazin-2(4*H*)-one (**3a**) (Wang et al., 2018) and 4-ethynyl-4-phenyl-1-tosyl-1,4-dihydro-2*H*-benzo[*d*][1,3]oxazin-2-one (**3i**) (Lu et al., 2018) was matched with reported data in literature.

4-Ethynyl-7-fluoro-4-methyl-1-tosyl-1*H*-benzo[*d*][1,3]oxazin-2(4*H*)-one (3b):

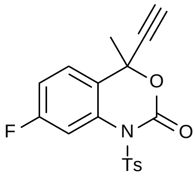

Following the general method **C**, compound **3b** was obtained as a white solid (0.23 g, Yield: 64%), m.p. = 153.1 – 155.0 °C. **^{1}H NMR** (500 MHz, $CDCl_3$) δ 8.15 – 8.07 (m, 2H), 7.43 (dd, *J* = 9.9, 2.4 Hz, 1H), 7.41 – 7.33 (m, 3H), 7.00 (td, *J* = 8.3, 2.4 Hz, 1H), 2.70 (s, 1H), 2.47 (s, 3H), 1.99 (s, 3H). **^{13}C NMR** (126 MHz, $CDCl_3$) δ 162.6 (d, *J* = 248.9 Hz), 148.0, 146.1, 135.1, 134.6 (d, *J* = 11.2 Hz), 129.7, 129.4, 125.1, 124.8, 113.1 (d, *J* = 22.2 Hz), 109.3 (d, *J* = 27.6 Hz), 80.8, 76.2, 75.2, 26.3, 21.8. **^{19}F NMR** (282 MHz, $CDCl_3$) δ −109.70 – −110.25 (m, 1F). **IR (KBr)**: 3262, 2125, 1756, 1612, 1502, 1428, 1371, 1286, 1178, 1062, 989, 846, 815, 757, 659, 559 cm^{-1}. **HRMS (ESI)** calculated for $C_{18}H_{14}FNO_4SNa$ [M+Na]$^+$: 382.0525, found: 382.0529.

4-Ethynyl-4-methyl-1-tosyl-7-(trifluoromethyl)-1*H*-benzo[*d*][1,3]oxazin-2(4*H*)-one (3c):

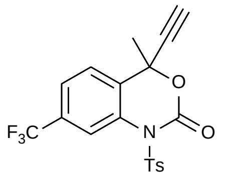

Following the general method **C**, compound **3c** was obtained as a white solid (0.425 g, Yield: 52%), m.p. = 155.6 – 157.1 °C. **^{1}H NMR** (500 MHz, $CDCl_3$) δ 8.15 – 8.10 (m, 2H), 7.96 – 7.93 (m, 1H), 7.59 – 7.51 (m, 2H), 7.43 – 7.38 (m, 2H), 2.72 (s, 1H), 2.48 (s, 3H), 2.02 (s, 3H). **^{13}C NMR** (126 MHz, $CDCl_3$) δ 147.7, 146.3, 134.9, 133.9, 132.6, 132.0 (q, *J* = 33.3 Hz), 129.7, 129.5, 124.1, 123.2 (q, *J* = 272.8 Hz), 123.1 (q, *J* = 3.6 Hz), 118.5 (q, *J* = 3.9 Hz), 80.2, 77.2, 75.1, 26.1, 21.8. **^{19}F NMR** (282 MHz, $CDCl_3$) δ −63.27 (s, 3F). **IR (KBr)**: 3270, 2125, 1760, 1594, 1430, 1382, 1332, 1232, 1132, 1178, 1062, 975, 817, 659, 543 cm^{-1}. **HRMS (ESI)** calculated for $C_{19}H_{14}F_3NO_4SNa$ $[M+Na]^+$: 432.0493, found: 432.0486.

6-Chloro-4-ethynyl-4-methyl-1-tosyl-1*H*-benzo[*d*][1,3]oxazin-2(4*H*)-one (3d):

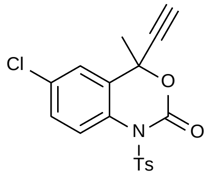

Following the general method **C**, compound **3d** was obtained as a white solid (0.525 g, Yield: 70%), m.p. = 142.0 – 143.8 °C. **^{1}H NMR** (500 MHz, $CDCl_3$) δ 8.13 – 8.06 (m, 2H), 7.59 – 7.64 (m, 1H), 7.45 – 7.34 (m, 4H), 2.70 (s, 1H), 2.47 (s, 3H), 1.98 (s, 3H). **^{13}C NMR** (126 MHz, $CDCl_3$) δ 147.9, 146.0, 135.1 131.9, 131.9, 130.9, 129.6, 129.5, 129.5, 123.7, 122.6, 80.4, 76.6, 75.0, 26.1, 21.8. **IR (KBr)**: 3288, 1754, 1484, 1361, 1238, 1164, 823, 667, 592, 541 cm^{-1}. **HRMS (ESI)** calculated for $C_{18}H_{14}NO_4SClNa$ $[M+Na]^+$: 398.0230, found: 398.0226.

4-Ethynyl-4,7-dimethyl-1-tosyl-1*H*-benzo[*d*][1,3]oxazin-2(4*H*)-one (3e):

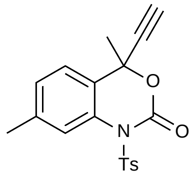

Following the general method **C**, compound **3e** was obtained as a white solid (0.42 g, Yield: 59%), m.p. = 137.2 – 139.1 °C. **^{1}H NMR** (500 MHz, $CDCl_3$) δ 8.13 – 8.09 (m, 2H), 7.49 (s, 1H), 7.40 – 7.34 (m, 2H), 7.28 – 7.24 (m, 1H), 7.12 –7.08 (m, 1H), 2.6 (s, 1H), 2.46 (s, 3H), 2.43 (s, 3H), 1.97 (s, 3H). **^{13}C NMR** (126 MHz, CDCl3) δ 148.5, 145.7, 139.8, 135.4, 133.2, 129.5, 129.5, 126.9, 126.4, 123.1, 121.7, 81.3, 75.7, 75.4, 26.2, 21.8, 21.6. **IR (KBr)**: 3293, 2121, 1749, 1612, 1359, 1280, 1238, 1164, 1080, 1063, 817, 763, 703, 661, 563 cm^{-1}. **HRMS (ESI)** calculated for $C_{19}H_{17}NO_4SNa$ $[M+Na]^+$: 378.0776, found: 378.0775.

6-Bromo-4-ethynyl-4-methyl-1-tosyl-1*H*-benzo[*d*][1,3]oxazin-2(4*H*)-one (3f):

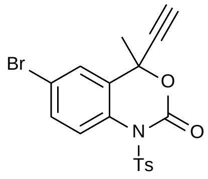

Following the general method **C**, compound **3f** was obtained as a white solid (0.436 g, Yield: 52%), m.p. = 133.2 – 135.0 °C. **^{1}H NMR** (500 MHz, $CDCl_3$) δ 8.12 – 8.07 (m, 2H), 7.57 – 7.55 (m, 2H), 7.53 – 7.50 (m, 1H), 7.38 (d, *J* = 8.1 Hz, 2H), 2.70 (s, 1H), 2.47 (s, 3H), 1.98 (s, 3H). **^{13}C NMR** (126 MHz, $CDCl_3$) δ 147.8, 146.0, 135.0, 132.5, 132.4, 131.1, 129.6, 129.5, 126.5, 122.9, 119.5, 80.4, 76.6, 74.9, 26.1, 21.8. **IR (KBr)**: 3259, 1754, 1590, 1479, 1359, 1295, 1232, 1164, 1085, 966, 667, 437 cm^{-1}. **HRMS (ESI)** calculated for $C_{18}H_{14}NO_4SBrNa$ $[M+Na]^+$: 441.9725, found: 441.9714.

4-Ethyl-4-ethynyl-1-tosyl-1*H*-benzo[*d*][1,3]oxazin-2(4*H*)-one (3g):

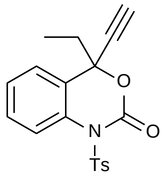

Following the general method **C**, compound **3g** was obtained as a white solid (0.39 g, Yield: 55%), m.p. = 94.4 – 95.2 °C. **^{1}H NMR** (500 MHz, $CDCl_3$) δ 8.15 – 8.11 (m, 2H), 7.66 – 7.62 (m, 1H), 7.46 – 7.41 (m, 2H), 7.41 – 7.36 (m, 2H), 7.29 (td, *J* = 7.8, 1.1 Hz, 1H), 2.75 (s, 1H), 2.47 (s, 3H), 2.24 (qd, *J* = 7.3, 1.2 Hz, 2H), 1.12 (t, *J* = 7.3 Hz, 3H). **^{13}C NMR** (126 MHz, $CDCl_3$) δ 148.2, 145.7, 135.5, 133.3, 129.6, 129.3, 129.3, 128.2, 126.0, 124.4, 121.1, 80.0, 79.8, 77.1, 32.2, 21.8, 8.3. **IR (KBr)**: 3270, 1751, 1594, 1459, 1373, 1297, 1220, 1174, 1085, 919, 815, 759, 674, 611, 543 cm□1. **HRMS (ESI)** calculated for $C_{19}H_{17}NO_4SNa$ $[M+Na]^+$: 378.0776, found: 378.0773.

4-Ethynyl-4-isopropyl-1-tosyl-1,4-dihydro-2*H*-benzo[*d*][1,3]oxazin-2-one (3h):

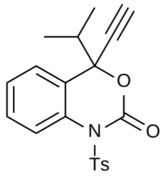

Following the general method **C**, compound **3h** was obtained as a white solid (0.43 g, Yield: 39%), m.p. = 111.6 – 112.4 °C. **^{1}H NMR** (500 MHz, $CDCl_3$) δ 8.15 – 8.09 (m, 2H), 7.63 – 7.58 (m, 1H), 7.52 – 7.48 (m, 1H), 7.46 – 7.36 (m, 3H), 7.28 (td, *J* = 7.6, 1.1 Hz, 1H), 2.83 (s, 1H), 2.50 – 2.38 (m, 4H), 1.13 (d, *J* = 6.6 Hz, 3H), 1.00 (d, *J* = 6.7 Hz, 3H).**^{13}C NMR** (126 MHz, $CDCl_3$) δ 147.6, 145.7, 135.57, 132.7, 129.7, 129.2, 129.0, 127.6, 126.3, 125.7, 121.0, 85.2, 78.4, 77.7, 35.8, 21.7, 18.1, 16.6. **IR (KBr)**: 3256, 2972, 1741, 1596, 1488, 1457, 1378, 1232, 1176, 757, 678, 593, 541 cm^{-1}. **HRMS (ESI)** calculated for $C_{20}H_{19}NO_4SNa$ $[M+Na]^+$: 392.0932, found: 392.0923.

General procedure for the synthesis of substituted 1-(2-aminophenyl)-2,2,2-trifluoroethanones (Method D), related to Scheme 6.

Route 1: The substituted 1-(2-aminophenyl)-2,2,2-trifluoroethanones (**S8**) were prepared according to the reported literature procedures with slight modification from the starting materials 2-nitrobenzaldehydes (**S4**) (Cheng et al., 2013; Punna et al., 2019; Sun et al., 2017; Kim et al., 2013).

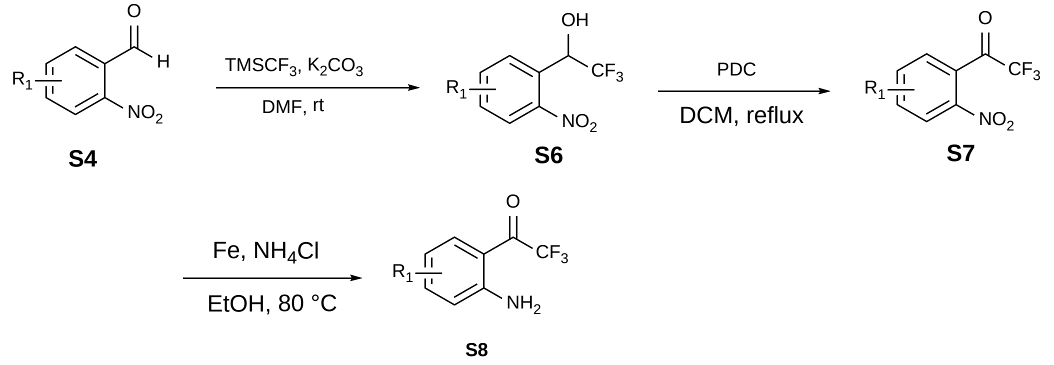

In a flame dried 100 mL round bottom flask, aldehyde **S4** (20 mmol, 1.0 equiv.) and dry K_2CO_3 (0.552 g, 0.2 equiv.) was suspended in anhydrous DMF (25 mL). To this solution $TMSCF_3$ (5.68 g, 2.0 equiv.) in 5 mL was added and the mixture was stirred vigorously at room temperature under N_2 atmosphere. Completion of the reaction was monitored by TLC. To this reaction mixture, aqueous HCl solution (2 M, 4 mL) was added and stirred for 30 min at room temperature. The reaction mixture was then extracted with ethyl acetate. Combined organic layers were finally washed with brine solution, dried and concentrated under reduced pressure. Then purification by chromatography on a short silica gel column (Hexane/Ethyl Acetate = 9:1) to afford compound **S6** as pure product.

In a flame dried 100 mL round bottom flask, **PDC** (9.4 g, 2.5 equiv.) was suspended in anhydrous DCM (25 mL). To this solution Alcohol **S6** (10 mmol, 1.0 equiv.) in 25 mL DCM was added and the mixture was stirred reflux under N_2 atmosphere. Completion of the reaction was monitored by TLC. Filtered through a pad of celite to remove the solid, and then concentrated under reduced pressure. Purification by chromatography on a short silica gel column (DCM) to afford compound **S7** as pure product.

In a 100 mL round bottom flask, ketone **S7** (9.1 mmol, 1.0 equiv.), Iron powder (1.55 g, 3.0 equiv.) and NH_4Cl (2.95 g, 6 equiv.) was added subsequently into 30mL H_2O/EtOH (v/v=1:5). The mixture was stirred at 80°C for 2h. Completion of the reaction was monitored by TLC. Filtered through a pad of celite to remove the solid, and then extracted with DCM, dried and concentrated under reduced pressure. Purification by chromatography on a short silica gel column (DCM) to afford compound **S8** as pure product.

Route 2: The substituted 1-(2-aminophenyl)-2,2,2-trifluoroethanones (**S8**) were prepared according to the reported literature procedures with slight modification from *o*-amino benzoic acids as starting materials (**S5**) (Allendörfer et al., 2012).

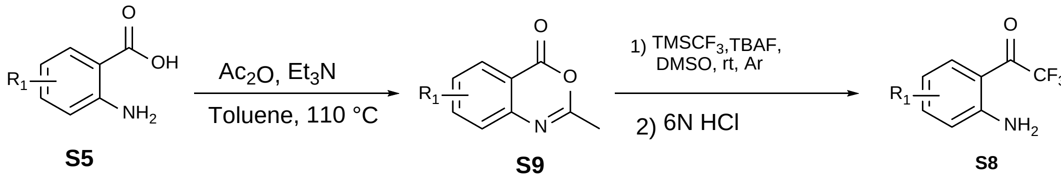

The Substituted *o*-amino benzoic acid **S5** (10 mmol, 1.0 equiv.) was dissolved in toluene (50 mL), then Ac_2O (2.84 mL, 3.0 equiv.) and NEt_3 (4.18 mL, 3.0 equiv.) were added. The mixture was stirred for 15 h at 110 °C. The solvent was removed under reduced pressure after complete consumption of starting material. The residue was taken up with water and ethyl acetate (3:1) and phases were separated. The organic layer was dried over Na_2SO_4 and the solvent removed under reduced pressure. The product **S9** was used immediately without further purification.

Under argon atmosphere benzoxazinone **S9** (9.17 mmol, 1.0 equiv.) was dissolved in dry DMSO. Trifluoromethylation reagent (4.0 mL, 3.00 equiv.) and TBAF (0.10 equiv., 1 M in THF) were added into the solution, and the mixture was stirred at rt for 15 h. After complete consumption of the starting material, the reaction mixture was quenched with 6 M HCl and stirred for an additional 1 h. Then, water was added, and the mixture was extracted with DCM. The organic layer was washed with saturated aq NH_4Cl and brine, dried and the solvent was removed under reduced pressure. Column chromatography (DCM) of the crude product yielded the trifluoromethylated ketones **S8**.

General procedure for the synthesis of trifluoromethyl substituted 4-methyl-*N*-(2-phenyl)benzenesulfonamides (Method E), related to Scheme 6.

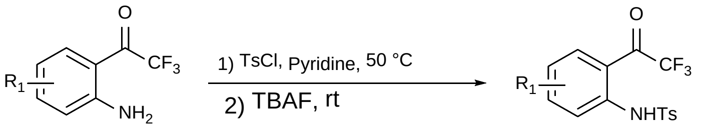

S8 **S10**

Fellow the general literature procedure with slight modification (Yasuhara et al., 1999), to a solution of trifluoromethylated ketones **S8** (5 mmol, 1.0 equiv.) in 10mL pyridine was added slowly *p*-toluenesulfonyl chloride (2.39 g, 2.5 equiv.). The resulting mixture was stirred at 50 °C under N_2 atmosphere. The mixture was evaporated to remove pyridine, quenched with water and extracted with DCM. The combined organic layer was washed with brine, then dried and concentrated. The crude residue was then dissolved in 15 mL dry THF, then TBAF (1.0 equiv., 1 M in THF) were added into the solution and keep the reaction at room temperature for 2 h under N_2 atmosphere. Completion of the reaction was monitored by TLC. The mixture was quenched with water and extracted with DCM. The combined organic layer was washed with brine, dried over anhydrous Na_2SO_4, filtered, and concentrated. The crude residue was purified by silica gel column chromatography to give **S10**.

4-Methyl-*N*-(2-(2,2,2-trifluoroacetyl)phenyl)benzenesulfonamide (S10a):

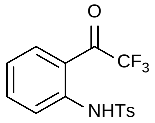

Following the **route 1** of general method **D** and method **E**, compound **S10a** was obtained as a light yellow solid (4.16 g, Yield: 80%), m.p. = 113.9 – 114.8 °C. **^{1}H NMR** (300 MHz, $CDCl_3$) δ 10.50 (s, 1H), 7.87 (d, *J* = 7.7 Hz, 1H), 7.83 – 7.68 (m, 3H), 7.61 (t, *J* = 7.5 Hz, 1H), 7.35 – 7.21 (m, 2H), 7.15 (t, *J* = 7.5 Hz, 1H), 2.38 (s, 3H). **^{13}C NMR** (126 MHz, $CDCl_3$) δ 182.7 (q, *J* = 34.9 Hz), 144.6, 142.4, 137.3, 135.9, 132.1, 129.9, 127.3, 123.0, 119.5, 116.3 (q, *J* = 291.2 Hz), 116.0, 21.6. **^{19}F NMR** (282 MHz, $CDCl_3$) δ −70.16 (s, 3F). **IR (KBr)**: 3234, 3064, 2922, 2867, 1682, 1606, 1573, 1496, 1454, 1346, 1278, 1159, 1089, 898, 816, 752 cm^{-1}. **HRMS (ESI)** calculated for $C_{15}H_{11}F_3NO_3S$ $[M-H]^+$: 342.0412, found: 342.0413.

***N*-(4-Fluoro-2-(2,2,2-trifluoroacetyl)phenyl)-4-methylbenzenesulfonamide (S10b):**

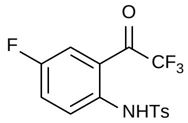

Following the **route 1** of general method **D** and method **E**, compound **S10b** was obtained as a light yellow solid (0.88 g, Yield: 77%), m.p. = 98.8 – 100.0 °C. **^{1}H NMR** (300 MHz, $CDCl_3$) δ 10.08 (s, 1H), 7.82 (dd, *J* = 9.4, 4.8 Hz, 1H), 7.73 – 7.58 (m, 2H), 7.52 (d, *J* = 8.5 Hz, 1H), 7.45 – 7.32 (m, 1H), 7.25 (d, *J* = 7.1 Hz, 2H), 2.38 (s, 3H). **^{13}C NMR** (126 MHz, $CDCl_3$) δ 181.9 (qd, *J* = 35.5, 2.5 Hz), 157.6 (d, *J* = 246.3 Hz), 144.9, 138.4, 135.6, 130.0, 127.3, 124.9 (d, *J* = 22.7 Hz), 122.9, 117.8 (dq, *J* = 24.7, 4.2 Hz), 117.5, 116.0 (q, *J* = 291.1 Hz), 21.6. **^{19}F NMR** (282 MHz, $CDCl_3$) δ −70.79 (s, 3F), −116.99 (q, *J* = 6.2 Hz, 1F). **IR (KBr)**: 3251, 3086, 2928, 2859, 1691, 1585, 1496, 1402, 1348, 1249, 1217, 1089, 987, 900, 815, 739, 682, 436 cm^{-1}. **HRMS (ESI)** calculated for $C_{15}H_{10}F_4NO_3S$ $[M-H]^+$: 360.0318, found: 360.0316.

4-Methyl-*N*-(2-(2,2,2-trifluoroacetyl)-5-(trifluoromethyl)phenyl)benzenesulfona-mide (S10c):

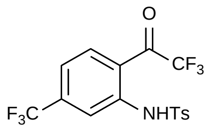

Following the **route 2** of general method **D** and method **E**, compound **S10c** was obtained as a light yellow solid (1.42 g, Yield: 42%), m.p. = 119.3 – 120.6 °C. **^{1}H NMR** (300 MHz, $CDCl_3$) δ 10.50 (s, 1H), 8.07 (s, 1H), 8.00 (d, *J* = 8.8 Hz, 1H), 7.87 – 7.64 (m, 2H), 7.36 (d, *J* = 8.6 Hz, 1H), 7.33 – 7.18 (m, 2H), 2.39 (s, 3H). **^{13}C NMR** (126 MHz, $CDCl_3$) δ 182.4 (q, *J* = 35.7 Hz), 145.3, 142.9, 138.0 (q, *J* = 33.5 Hz), 135.4, 132.9 (q, *J* = 4.2 Hz), 130.1, 127.5, 122.5 (q, *J* = 273.7 Hz), 119.1 (q, *J* = 3.6 Hz), 117.6, 116.2 (q, *J* = 4.0 Hz), 116.1 (q, *J* = 290.9 Hz), 21.6. **^{19}F NMR** (282 MHz, $CDCl_3$) δ −64.76 (s, 3F), −70.46 (s, 3F). **IR (KBr)**: 3246, 3064, 2924, 2864, 1695, 1574, 1512, 1431, 1338, 1296, 1163, 1088, 960, 920, 866, 783, 742, 661, 564 cm^{-1}. **HRMS (ESI)** calculated for $C_{16}H_{10}F_6NO_3S$ $[M-H]^+$: 410.0286, found: 410.0298.

***N*-(4-Chloro-2-(2,2,2-trifluoroacetyl)phenyl)-4-methylbenzenesulfonamide (S10d):**

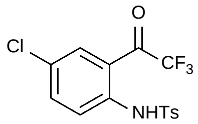

Following the **route 1** of general method **D** and method **E**, compound **S10d** was obtained as a light yellow solid (3.17 g, Yield: 69%), m.p. = 110.3 – 111.2 °C. **^{1}H NMR** (300 MHz, $CDCl_3$) δ 10.30 (s, 1H), 7.86 – 7.74 (m, 2H), 7.71 (d, *J* = 8.1 Hz, 2H), 7.56 (d, *J* = 9.0, 1H), 7.33 – 7.15 (m, 2H), 2.39 (s, 3H). **^{13}C NMR** (126 MHz, $CDCl_3$) δ 181.9 (q, *J* = 35.7 Hz), 144.9, 140.9, 137.2, 135.6, 131.3, 130.0, 128.6, 127.3, 121.3, 117.0, 116.0 (q, *J* = 291.1 Hz), 21.6. **^{19}F NMR** (282 MHz, $CDCl_3$) δ −70.42 (s, 3F). **IR (KBr)**: 3248, 3124, 2926, 2868, 1691, 1599, 1486, 1400, 1344, 1273, 1163, 1089, 962, 899, 816, 717, 574, 546 cm^{-1}. **HRMS (ESI)** calculated for $C_{15}H_{10}ClF_3NO_3S$ $[M-H]^+$: 376.0022, found: 376.0026.

Methyl 3-(4-methylphenylsulfonamido)-4-(2,2,2-trifluoroacetyl)benzoate (S10g)

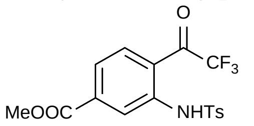

Following the **route 1** of general method **D** and method **E**, compound **S10g** was obtained as a light yellow solid (1.26 g, Yield: 78%), m.p. = 155.6 – 156.9 °C. **^{1}H NMR** (500 MHz, $CDCl_3$) δ 10.38 (s, 1H), 8.40 (s, 1H), 7.93 (dd, *J* = 8.5, 2.1 Hz, 1H), 7.79 (s, 1H), 7.78 (s, 1H), 7.75 (dd, *J* = 8.5, 1.5 Hz, 1H), 7.29 (s, 1H), 7.27 (s, 1H), 3.97 (s, 3H), 2.39 (s, 3H). **^{13}C NMR** (126 MHz, $CDCl_3$) δ 182.6 (q, *J* = 35.6 Hz), 164.9, 144.9, 142.4, 137.3, 135.6, 132.1, 130.0, 127.5, 123.2, 120.4, 118.4, 116.1 (q, *J* = 291.0 Hz), 53.1, 21.6. **^{19}F NMR** (282 MHz, $CDCl_3$) δ −70.48 (s, 3F). **IR (KBr)**: 3269, 3012, 2960, 2922, 1730, 1691, 1597, 1566, 1415, 1344, 1286, 1261, 1091, 951, 870, 816, 565 cm^{-1}. **HRMS (ESI)** calculated for $C_{17}H_{13}F_3NO_5S$ $[M-H]^+$: 400.0467, found: 400.0457.

***N*-(4,5-Dimethoxy-2-(2,2,2-trifluoroacetyl)phenyl)-4-methylbenzenesulfonamide (S10h)**

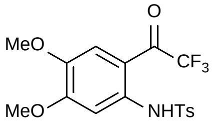

Following the **route 1** of general method **D** and method **E**, compound **S10h** was obtained as a light yellow solid (2.35 g, Yield: 97%), m.p. = 124.8 – 127.3 °C. **^{1}H NMR** (300 MHz, $CDCl_3$) δ 10.67 (s, 1H), 7.80 – 7.55 (m, 2H), 7.35 (s, 1H), 7.30 – 7.20 (m, 2H), 7.17 (s, 1H), 3.97 (s, 3H), 3.84 (s, 3H), 2.39 (s, 3H). **^{13}C NMR** (126 MHz, $CDCl_3$) δ 180.4 (q, *J* = 34.3 Hz), 156.8, 144.6, 144.6, 139.8, 135.8, 129.8, 127.3, 116.6 (q, *J* = 291.0 Hz), 112.2 (q, *J* = 4.5 Hz), 108.8, 102.8, 56.5, 56.1, 21.6. **^{19}F NMR** (282 MHz, $CDCl_3$) δ −70.25 (s, 3F). **IR (KBr)**: 3192, 2941, 2861, 1658, 1616, 1527, 1369, 1296, 1263, 1190, 1161, 1090, 1005, 897, 837, 725 cm^{-1}. **HRMS (ESI)** calculated for $C_{17}H_{15}F_3NO_5S$ $[M-H]^+$: 420.0623, found: 402.0626.

General procedure for the synthesis of perfluoroalkyl substituted 4-ethynyl-1-tosyl-1*H*-benzo[*d*][1,3]oxazin-2(4*H*)-ones (Method F), related to Scheme 6.

Overall reaction steps for the synthesis of trifluoromethyl substituted 4-ethynyl-1-tosyl-1*H*-benzo[*d*][1,3]oxazin-2(4*H*)-ones **4a** to **4h** is showing below (Sun et al., 2017).

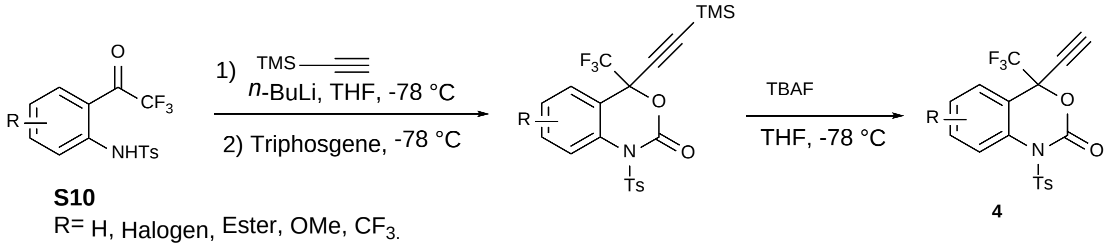

Under a dry nitrogen atmosphere, 30 mL of dry THF was added to a 100 mL round bottom flask, followed by the ethynyltrimethylsilane (2.2 mL, 16 mmol). The solution was then cooled at −78 °C and 1.6 M *n*-butyllithium solution in THF (10.0 mL, 16 mmol) was then added dropwise by syringe. After stirring for 20 min, 4-methyl-*N*-(2-(2,2,2-trifluoroacetyl)phenyl)benzenesulfon-amide (**S10**) (2.49 g, 7.24 mmol) in THF was added slowly to the reaction mixture for 30 min. The mixture was then keep stirring for 1 h, and then checked for conversion of sulfonamide by TLC.

After the complete conversion of sulfonamide, triphosgene (2.6 g, 9.4 mmol) in 5 mL dry THF was added dropwise. The reaction mixture was then stirred for 2 h. Once full conversion of the intermediate was verified by TLC, the reaction was quenched with water slowly. The solution was then concentrated to remove THF, then extracted with DCM, and the combined organic layers dried with sodium sulfate then concentrated to afford a dark brown crude solid. The residue was undergoing a short silica pad then directly used for next step.

Under a nitrogen atmosphere, the crude solid was added into a 100 mL round bottom flask and dissolved in 30 mL of dry THF and cooled at −78 °C. Tetrabutylammonium fluoride solution (1.0 M) in THF (8.5 mL, 6.9mmol) was then added dropwise, and reaction was then stirred for 30 min. After the reaction completed as checked by TLC, the reaction was quenched with water dropwise and warm to room temperature. The solution was then concentrated to remove THF, then extracted with DCM, and the combined organic layers dried, concentrated to afford a dark brown crude solid. Purification by column chromatography (hexane/ethyl acetate = 5:1) afforded the pure trifluoromethylated propargyl benzoxazinanones.

4-Ethynyl-1-tosyl-4-(trifluoromethyl)-1*H*-benzo[*d*][1,3]oxazin-2(4*H*)-one (4a):

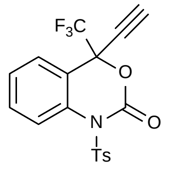

Following the general method **F**, compound **4a** was obtained as a white solid (1.9 g, Yield: 78%), m.p. = 173.6 – 174.9 °C. **^{1}H NMR** (300 MHz, $CDCl_3$) δ 8.14 – 8.00 (m, 2H), 7.82 – 7.71 (m, 1H), 7.71 – 7.62 (m, 1H), 7.62 – 7.51 (m, 1H), 7.45 – 7.33 (m, 3H), 2.94 (s, 1H), 2.48 (s, 3H). **^{13}C NMR** (126 MHz, $CDCl_3$) δ 146.3, 145.3, 134.8, 133.7, 131.2, 129.7, 129.5, 127.0, 126.4, 121.4 (q, *J* = 287.0 Hz), 121.1, 119.2, 79.7, 77.8 (q, *J* = 35.5 Hz), 74.0, 21.8. **^{19}F NMR** (282 MHz, $CDCl_3$) δ −78.47 (s, 3F). **IR (KBr)**: 3271, 3103, 2927, 2137, 1766, 1597, 1493, 1460, 1381, 1304, 1203, 1174, 1084, 818, 746 cm^{-1}. **HRMS (ESI)** calculated for $C_{18}H_{12}F_3NO_4SNa$ $[M+Na]^+$: 418.0337, found: 418.0342.

6-Fluoro-4-ethynyl-1-tosyl-4-(trifluoromethyl)-1*H*-benzo[*d*][1,3]oxazin-2(4*H*)-one (4b):

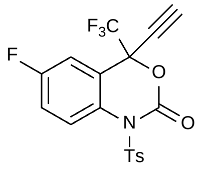

Following the general method **F**, compound **4b** was obtained as a white solid (0.81 g, Yield: 68%), m.p. = 167.7 – 168.7 °C. **^{1}H NMR** (300 MHz, $CDCl_3$) δ 8.20 – 7.93 (m, 2H), 8.04 (s, 1H), 7.76 (dd, *J* = 9.3, 4.4 Hz, 1H), 7.47 – 7.32 (m, 3H), 7.31 – 7.21 (m, 1H), 2.97 (s, 1H), 2.47 (s, 3H). **^{13}C NMR** (126 MHz, $CDCl_3$) δ 160.0 (d, *J* = 249.3 Hz), 146.5, 145.0, 134.5, 129.82, 129.80, 129.5, 123.2, 121.3 (q, *J* = 287.1 Hz), 121.2, 118.4 (d, *J* = 22.8 Hz), 114.2 (d, *J* = 26.4 Hz), 80.2, 77.2 (q, *J* = 36.0 Hz), 73.5, 21.8. **^{19}F NMR** (282 MHz, $CDCl_3$) δ −78.34 (s, 3F), −113.99 (q, *J* = 6.9 Hz, 1F). **IR (KBr)**: 3273, 3078, 2927, 2137, 1770, 1597, 1500, 1381, 1308, 1209, 1176, 1086, 867, 816, 742 cm^{-1}. **HRMS (ESI)** calculated for $C_{18}H_{11}F_4NO_4SNa$ $[M+Na]^+$: 436.0243, found: 436.0240.

4-Ethynyl-1-tosyl-4,7-bis(trifluoromethyl)-1*H*-benzo[*d*][1,3]oxazin-2(4*H*)-one (4c):

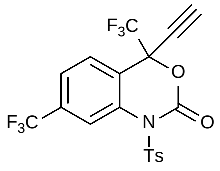

Following the general method **F**, compound **4c** was obtained as a white solid (0.91 g, Yield: 52%), m.p. = 113.0 – 114.3 °C. **^{1}H NMR** (300 MHz, $CDCl_3$) δ 8.16 – 7.95 (m, 3H), 7.80 (d, *J* = 8.3 Hz, 1H), 7.62 (d, *J* = 8.0 Hz, 1H), 7.41 (d, *J* = 8.0 Hz, 2H), 2.99 (s, 1H), 2.49 (s, 3H). **^{13}C NMR** (126 MHz, $CDCl_3$) δ 146.8, 144.6, 134.3, 134.2, 133.6 (q, *J* = 33.6 Hz), 129.9, 129.6, 127.8, 123.1, 123.0 (q, *J* = 273.2 Hz), 122.7, 121.2 (q, *J* = 287.1 Hz), 118.4, 80.4, 77.4 (q, *J* = 35.8 Hz), 73.3, 21.9. **^{19}F NMR** (282 MHz, $CDCl_3$) δ −63.60 (s, 3F), −78.24 (s, 3F). **IR (KBr)**: 3276, 3070, 2929, 2870, 2135, 1778, 1623, 1595, 1431, 1383, 1333, 1209, 1175, 1086, 885, 816, 741 cm^{-1}. **HRMS (ESI)** calculated for $C_{19}H_{11}F_6NO_4SNa$ $[M+Na]^+$: 486.0211, found: 486.0211.

6-Chloro-4-ethynyl-1-tosyl-4-(trifluoromethyl)-1*H*-benzo[*d*][1,3]oxazin-2(4*H*)-one (4d):

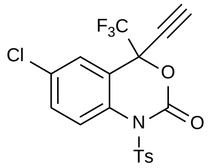

Following the general method **F**, compound **4d** was obtained as a white solid (0.78 g, Yield: 73%), m.p. = 140.7 – 143.0 °C. **^{1}H NMR** (300 MHz, $CDCl_3$) δ 8.13 – 7.94 (m, 2H), 8.03 (s, 1H), 7.73 (d, *J* = 8.9 Hz, 1H), 7.62 (s, 1H), 7.53 (d, *J* = 8.9 Hz, 1H), 7.45 – 7.32 (m, 2H), 2.98 (s, 1H), 2.48 (s, 3H). **^{13}C NMR** (126 MHz, $CDCl_3$) δ 146.6, 144.8, 134.3, 132.2, 132.2, 131.4, 129.8, 129.6, 127.0, 122.5, 121.3 (q, *J* = 287.3 Hz), 120.8, 80.3, 77.3 (q, *J* = 35.7 Hz), 73.4, 21.8. **^{19}F NMR** (282 MHz, $CDCl_3$) δ −78.44 (s, 3F). **IR (KBr)**: 3273, 2925, 2135, 1770, 1595, 1487, 1381, 1297, 1203, 1174, 1084, 965, 928, 816, 701 cm^{-1}. **HRMS (ESI)** calculated for $C_{18}H_{11}ClF_3NO_4SNa$ $[M+Na]^+$: 451.9947, found: 451.9955.

Methyl 4-ethynyl-2-oxo-1-tosyl-4-(trifluoromethyl)-2,4-dihydro-1*H*-benzo[*d*][1,3] oxazine-7-carboxylate (4g):

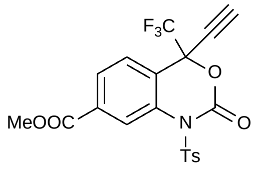

Following the general method **F**, compound **4g** was obtained as a white solid (1.0 g, Yield: 70%), m.p. = 146.3 – 147.1 °C. **^{1}H NMR** (300 MHz, $CDCl_3$) δ 8.42 (s, 1H), 8.11 (d, *J* = 8.5 Hz, 2H), 8.01 (d, *J* = 8.2 Hz, 1H), 7.72 (d, *J* = 8.2 Hz, 1H), 7.42 (d, *J* = 8.1 Hz, 2H), 3.99 (s, 3H), 2.99 (s, 1H), 2.48 (s, 3H). **^{13}C NMR** (126 MHz, $CDCl_3$) δ 165.1, 146.6, 144.9, 134.5, 134.0, 133.1, 129.8, 129.6, 127.2, 127.1, 123.4, 122.1, 121.3 (q, *J* = 287.2 Hz), 80.3, 77.5 (q, *J* = 35.7 Hz), 73.4, 52.9, 21.8. **^{19}F NMR** (282 MHz, $CDCl_3$) δ −78.15 (s, 3F). **IR (KBr)**: 3271, 2956, 2927, 2847, 2133, 1774, 1728, 1591, 1381, 1292, 1204, 1090, 814, 764, 741 cm^{-1}. **HRMS (ESI)** calculated for $C_{20}H_{14}F_3NO_6SNa$ $[M+Na]^+$: 476.0392, found: 476.0385.

4-Ethynyl-6,7-dimethoxy-1-tosyl-4-(trifluoromethyl)-1*H*-benzo[*d*][1,3]oxazin2(4-*H*)-one (4h):

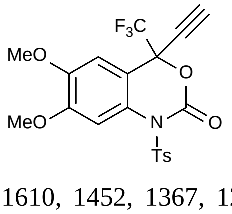

Following the general method **F**, compound **4h** was obtained as a white solid (1.28 g, Yield: 78%), m.p. = 146.3 – 147.1 °C. **^{1}H NMR** (300 MHz, $CDCl_3$) δ 8.02 (d, *J* = 8.1 Hz, 2H), 7.38 (d, *J* = 8.1 Hz, 2H), 7.31 (s, 1H), 7.03 (s, 1H), 3.97 (s, 3H), 3.92 (s, 3H), 2.95 (s, 1H), 2.47 (s, 3H). **^{13}C NMR** (126 MHz, $CDCl_3$) δ 150.8, 147.3, 146.3, 145.4, 134.7, 129.6, 129.5, 127.5, 121.5 (q, *J* = 287.1 Hz), 110.2, 109.0, 105.0, 79.6, 77.9 (q, *J* = 35.5 Hz), 74.2, 56.4, 21.8. **^{19}F NMR** (282 MHz, $CDCl_3$) δ −78.97 (s, 3F). **IR (KBr)**: 3271, 3066, 2941, 2866, 2131, 1766, 1610, 1452, 1367, 1230, 1174, 1088, 1039, 854, 814, 739 cm^{-1}. **HRMS (ESI)** calculated for $C_{20}H_{16}F_3NO_6SNa$

$[M+Na]^+$: 478.0548, found: 478.0547.

General experimental procedure for the preparation of sulfur ylides and sulfonium salts (Method H), related to Scheme 4 and Scheme 6.

Sulfur ylides **2** were prepared according to known methods. A typical experimental procedure for the preparation of sulfur ylides were described below.

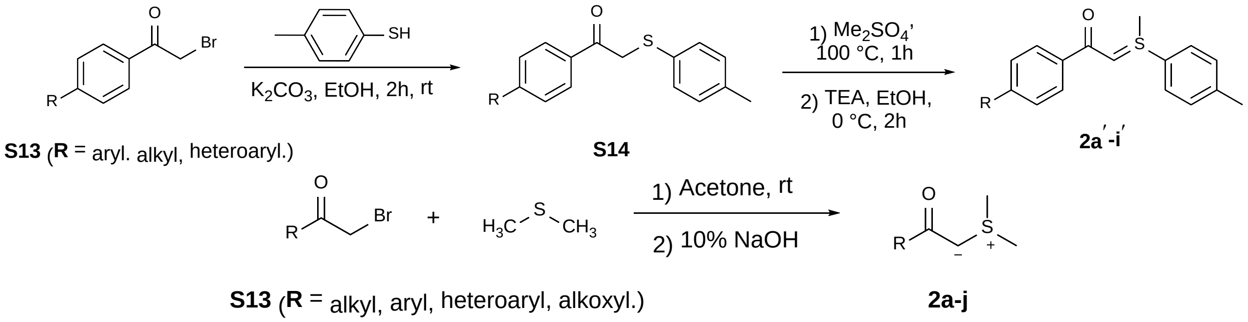

4-Methylthiophenol (1.0 equiv., 10.00 mmol, 1.24 g) was charged into a dry 100 mL flask along with ethanol (20 mL), magnetic stir bar and K_2CO_3 (1.0 equiv., 10.0 mmol, 1.38 g). The α-bromo ketone (1.0 equiv., 10.0 mmol) was added in one portion. The resulting suspension was stirred for 2h at room temperature. The crude reaction mixture was filtered through a pad of celite and washed with EtOH. The solvent was removed in vacuo. The residue was purified by flash silica gel chromatography (using 95:5 hexane/ethyl acetate). The resulting sulfide was transferred into a vial. In a glove box, Me_2SO_4 (1.0 equiv.) was added and the vial was sealed. The vial was stirred for 1 h at 100 °C and allowed to cool to room temperature. The resulting semi-solid was transferred to a flask, EtOH (99.9%, 1.0 M) added and the mixture cooled to 0 °C. Triethylamine (1.1 equiv.) was added and the reaction stirred 2 hours at 0 °C. The reaction mixture was transferred to a separatory funnel containing water and DCM. The phases were separated and the aqueous was extracted twice with DCM. The combined organic phases were washed with water and then dried over $MgSO_4$. All solvent was removed in vacuo yielding a solid which further recrystallized from DCM and hexane. The characterization data of **2a'-2g'** are summarized below, and sulfur ylides **2a-2i** were prepared according to the known procedure, the characterization data are match with the previous data (Søren et al., 2012; Anderson et al., 1984; Ratts et al., 1966; Payne et al., 1967; Quintana et al., 1973).

Methyl(4-methylphenyl)sulfonium phenacylide (2a'):

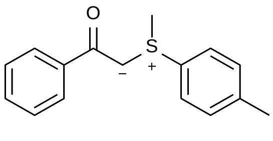

Following the general method **H**, compound **2a'** was obtained as a white solid (1.59 g, Yield: 62%), m.p. = 90.5 – 92.0 °C. **^{1}H NMR** (500 MHz, $CDCl_3$) δ 7.90 – 7.83 (m, 2H), 7.63 (d, J = 8.3 Hz, 2H), 7.39 – 7.33 (m, 3H), 7.27 (d, J = 7.4 Hz, 2H), 4.57 (s, 1H), 3.14 (s, 3H), 2.37 (s, 3H). **^{13}C NMR** (126 MHz, $CDCl_3$) δ 182.1, 141.4, 140.7, 131.8, 130.4, 129.4, 127.8, 126.9, 126.5, 53.1, 30.6, 21.2. **IR (KBr)**: 3068, 1583, 1513, 1394, 1205, 987, 858, 707 cm^{-1}. **HRMS (ESI)** calculated for $C_{16}H_{17}OS$ $[M+H]^+$: 257.1000, found: 257.1002.

Methyl (4-methylphenyl)sulfonium 4-methoxyphenacylide (2b'):

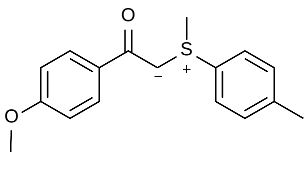

Following the general method **H**, compound **2b'** was obtained as a white solid (1.6 g, Yield: 56%), m.p. = 78.2 – 79.2 °C. **^{1}H NMR** (500 MHz, $CDCl_3$) δ 7.86 – 7.80 (m, 2H), 7.63 (d, J = 8.3 Hz, 2H), 7.28 (d, J = 8.0 Hz, 2H), 6.91 – 6.84 (m, 2H), 4.51 (s, 1H), 3.83 (s, 3H), 3.15 (s, 3H), 2.38 (s, 3H). **^{13}C NMR** (126 MHz, $CDCl_3$) δ 181.8, 160.9, 141.4, 133.6, 132.2, 130.5, 128.3, 127.0, 113.1, 55.3, 52.0, 30.8, 21.3. **IR (KBr)**: 3064, 1606, 1583, 1498, 1253, 1091, 985, 862, 619 cm^{-1}. **HRMS (ESI)** calculated for $C_{17}H_{19}O_2S$ $[M+H]^+$: 287.1106, found: 287.1107.

Methyl (4-methylphenyl)sulfonium 4-methylphenacylide (2c'):

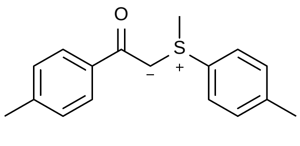

Following the general method **H**, compound **2c'** was obtained as a white solid (1.67 g, Yield: 62%), m.p. = 86.2 – 87.8 °C. **^{1}H NMR** (500 MHz, $CDCl_3$) δ 7.77 (d, J = 7.5 Hz, 2H), 7.63 (d, J = 7.6 Hz, 2H), 7.27 (d, J = 9.0 Hz, 2H), 7.16 (d, J = 7.7 Hz, 2H), 4.54 (s, 1H), 3.15 (s, 3H), 2.38 (s, 3H), 2.36 (s, 3H). **^{13}C NMR** (126 MHz, $CDCl_3$) δ 182.3,

141.4, 139.6, 138.1, 132.0, 130.5, 128.5, 127.0, 126.6, 52.5, 30.7, 21.3, 21.3. **IR (KBr)**: 3066, 1579, 1502, 1392, 983, 862, 742 cm^{-1}. **HRMS (ESI)** calculated for $C_{17}H_{19}OS$ [M+H]$^+$: 271.1157, found: 271.1164.

Methyl(4-methylphenyl)sulfonium 4-nitrophenacylide (2d'):

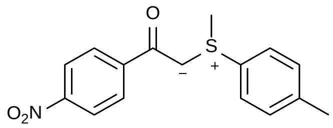

Following the general method **H**, compound **2d'** was obtained as a yellow solid (1.87 g, Yield: 62%), m.p. = 82.4 – 83.8 °C. **^{1}H NMR** (500 MHz, $CDCl_3$) δ 8.20 (d, *J* = 8.9 Hz, 2H), 7.98 (d, *J* = 8.6 Hz, 2H), 7.67 (d, *J* = 8.0 Hz, 2H), 7.32 (d, *J* = 8.1 Hz, 2H), 4.67 (s, 1H), 3.19 (s, 3H), 2.40 (s, 3H). **^{13}C NMR** (126 MHz, $CDCl_3$) δ 179.1, 148.3, 146.6, 142.2, 130.7, 127.5, 127.2, 123.2, 56.6, 30.3, 21.3. **IR (KBr)**: 3062, 1529, 1346, 983, 848, 711, 464 cm^{-1}. **HRMS (ESI)** calculated for $C_{16}H_{16}O_3NS$ [M+H]$^+$: 302.0851, found: 302.0850.

Methyl(4-methylphenyl)sulfonium 4-fluorophenacylide (2e'):

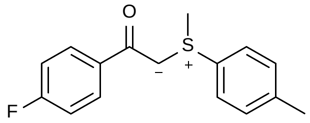

Following the general method **H**, compound **2e'** was obtained as a pale yellow solid (1.72 g, Yield: 63%), m.p. = 89.6 – 91.3 °C. **^{1}H NMR** (500 MHz, $CDCl_3$) δ 7.95 – 7.76 (m, 2H), 7.63 (d, *J* = 6.6 Hz, 2H), 7.35 – 7.23 (m, 2H), 7.02 (t, *J* = 8.7 Hz, 2H), 4.52 (s, 1H), 3.14 (s, 3H), 2.38 (s, 3H). **^{13}C NMR** (126 MHz, $CDCl_3$) δ 180.9, 163.7 (d, *J* = 248.0 Hz), 141.7, 137.0, 131.7, 130.6, 128.7 (d, *J* = 7.4 Hz), 127.0, 114.6 (d, *J* = 21.3 Hz), 53.5, 30.7, 21.3. **^{19}F NMR** (282 MHz, $CDCl_3$) δ −112.40 – −112.80 (m, 1F). **IR (KBr)**: 3068, 1598, 1517, 1390, 1081, 985, 846, 750, 620 cm^{-1}. **HRMS (ESI)** calculated for $C_{16}H_{16}FOS$ [M+H]$^+$: 275.0906, found: 275.0911.

Methyl(4-methylphenyl)sulfonium 4 - trifluorophenacylide (2f'):

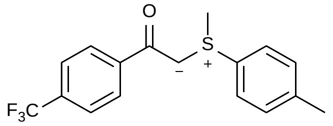

Following the general method **H**, compound **2f'** was obtained as a pale yellow solid (2.49 g, Yield: 77%), m.p. = 90.5 – 91.6 °C. **^{1}H NMR** (500 MHz, $CDCl_3$) δ 7.94 (d, *J* = 8.1 Hz, 2H), 7.65 (d, *J* = 8.3 Hz, 2H), 7.61 (d, *J* = 8.1 Hz, 2H), 7.30 (d, *J* = 8.0 Hz, 2H), 4.61 (s, 1H), 3.18 (s, 3H), 2.39 (s, 3H). **^{13}C NMR** (126 MHz, $CDCl_3$) δ 180.5, 144.1, 141.9, 131.2, 131.0, 130.6, 127.1, 126.9, 124.9 (q, *J* = 3.7 Hz), 124.2 (q, 272.1 Hz), 54.9, 30.5, 21.3. **^{19}F NMR** (282 MHz, $CDCl_3$) δ −63.01 (s, 3F). **IR (KBr)**: 3068, 1517, 1328, 1157, 1124, 862, 495 cm^{-1}. **HRMS (ESI)** calculated for $C_{17}H_{16}OSF_3$ [M+H]$^+$: 325.0874, found: 325.0881.

Methyl(4-methylphenyl)sulfonium 4-bromophenacylide (2g'):

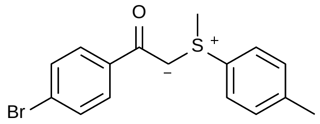

Following the general method **H**, compound **2g'** was obtained as a pale yellow solid (2.37 g, Yield: 71%), m.p. = 79.5 – 80.9 °C. **^{1}H NMR** (500 MHz, $CDCl_3$) δ 7.76 – 7.69 (m, 2H), 7.63 (d, *J* = 8.3 Hz, 2H), 7.51 – 7.43 (m, 2H), 7.29 (d, *J* = 8.1 Hz, 2H), 4.55 (s, 1H), 3.14 (s, 3H), 2.38 (s, 3H). **^{13}C NMR** (126 MHz, $CDCl_3$) δ 180.8, 141.8, 139.7, 131.5, 131.0, 130.6, 128.4, 127.1, 123.8, 54.0, 30.6, 21.3. **IR (KBr)**: 3066, 1573, 1509, 1085, 985, 858, 740, 553 cm^{-1}. **HRMS (ESI)** calculated for $C_{16}H_{16}OSBr$ [M+H]$^+$: 335.0105, found: 335.0104.

(*E*)-2-(methyl(p-tolyl)-λ^4-sulfaneylidene)-1-(thiophen-2-yl)ethan-1-one (2h'):

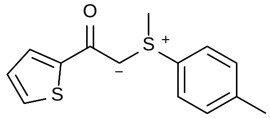

Following the general method **H**, compound **2h'** was obtained as a pale reddish solid (0.5 g, Yield: 38%), m.p. = 110.2 – 111.2 °C. **^{1}H NMR** (500 MHz, $CDCl_3$) δ 7.70 – 7.63 (m, 2H), 7.48 – 7.44 (m, 1H), 7.33 – 7.24 (m, 3H), 7.04 – 6.99 (m, 1H), 4.49 (s, 1H), 3.19 (s, 3H), 2.37 (s, 3H). **^{13}C NMR** (126 MHz, $CDCl_3$) δ 175.7, 147.5, 141.6, 131.9, 130.5, 127.3, 127.2, 127.2, 125.7, 51.7, 30.5, 21.3. **IR (KBr)**: 3064, 1523, 1421, 1380, 1201, 1081, 973, 856, 725, 501 cm^{-1}. **HRMS (ESI)** calculated for $C_{14}H_{15}OS_2$ [M+H]$^+$: 263.0564, found: 263.0568.

(*E*)-1-cyclohexyl-2-(methyl(p-tolyl)-λ^4-sulfaneylidene)ethan-1-one (2i'):

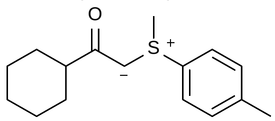

Following the general method **H**, compound **2i'** was obtained as a pale reddish solid (0.71 g, Yield: 67%), m.p. = 104.2 – 105.2 °C. **^{1}H NMR** (500 MHz, $CDCl_3$) δ 7.58 – 7.53 (m, 2H), 7.28 – 7.23 (m, 2H), 3.86 (s, 1H), 3.02 (s, 3H), 2.38 (s, 3H), 2.13 (tt, *J* = 11.8, 3.4 Hz, 1H), 1.90 – 1.81 (m, 2H), 1.80 – 1.72 (m, 2H), 1.69 – 1.60 (m, 1H), 1.43 (qd, *J* = 12.4, 3.3 Hz, 2H), 1.33 – 1.14 (m, 3H). **^{13}C NMR** (126 MHz, $CDCl_3$) δ 194.5, 141.2, 132.7, 130.4, 126.8, 51.3, 49.4, 31.3, 30.8, 30.7, 26.4, 26.4, 26.2, 21.3. **IR (KBr)**: 2925, 2850, 1546, 1376, 1105, 985, 804, 570 cm^{-1}. **HRMS (ESI)** calculated for $C_{16}H_{23}OS$ [M+H]$^+$: 263.1470, found: 263.1468.

Supplemental Experimental Procedures and Spectral Data of Products:

General Procedure for the asymmetric [4 + 1] cycloaddition reaction (Method I), related to scheme 4

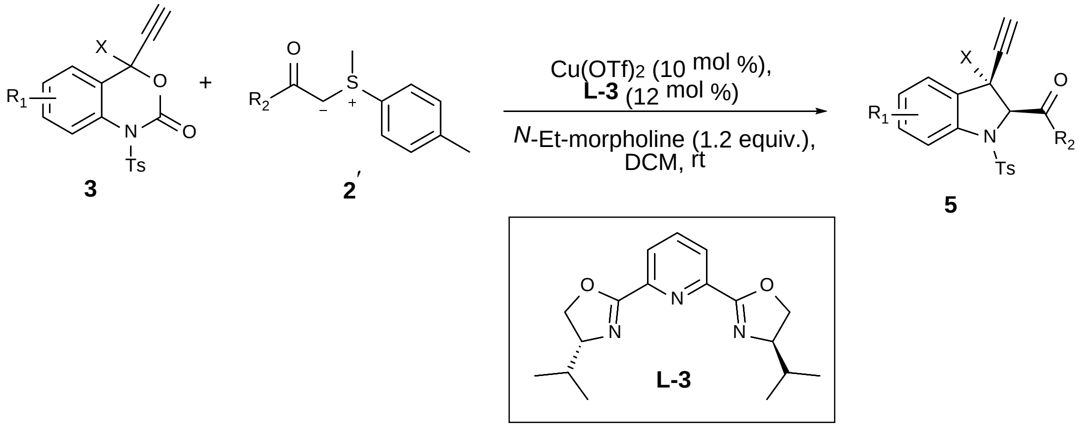

Under argon atmosphere, a flame-dried 10 mL Schlenk tube was charged with copper (II) trifluoromethanesulfonate (3.62 mg, 0.01 mmol, 10 mol %), 2,6-bis[(4*R*)-isopropyl-2-oxazolin-2-yl]-pyridine **L3** (3.62 mg, 0.012 mmol, 12 mol%) and anhydrous DCM (1 mL). The resulting solution was stirred for 1 h at room temperature. Then ethynyl benzoxazinanones **3** (0.1 mmol), sulfur ylides **2'** (0.15 mmol) and *N*-ethylmorpholine (15.2 μL, 0.12 mmol, 1.2 equiv.) were added. The resulting solution was stirred until complete conversion of ethynyl benzoxazinanones (monitored by TLC). The reaction was quenched by saturated NH_4Cl aqueous solution (2 mL). The resulting solution was extracted with ethyl acetate (5 mL x 3). The combined organic layers were dried over Na_2SO_4, filtered and concentrated in *vacuo*. The diastereomeric ratio was determined by ^{1}H NMR analysis of the crude reaction mixture. The residue was purified by flash silica gel chromatography (Hexane/EtOAc= 95:5) to afford the title compound **5**. The characterization data of **5** are summarized below.

General Procedure for the copper catalyzed intermolecular cyclization reactions (Method J), related to scheme 6

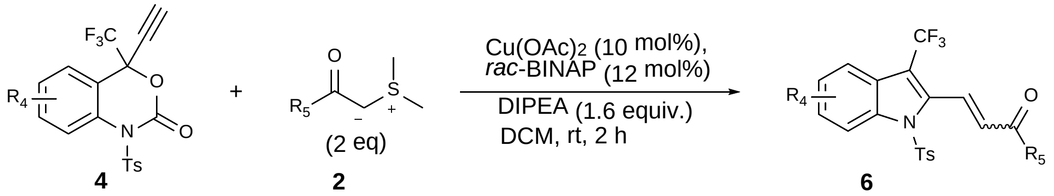

In a flam dried tube, $Cu(OAc)_2$ (0.01 mmol, 1.8 mg) and *rac*-BINAP (0.012 mmol, 7.5 mg) were mixed in 2.0 mL dry DCM and stirred at ambient temperature for 30 min under argon atmosphere. After the mixture became clarify, *i*-Pr_2NEt (0.16 mmol, 35 μL) and substrate **2** (0.2 mmol, 2.0 eq.) were added, followed by **4** (0.1 mmol, 1.0 eq.) after stirred for 1h. The reaction mixture was stirred at ambient temperature until the substrate **4** fully disappeared (determined by TLC). After that, the reaction was quenched by saturated NH_4Cl solution. The organic layer was separated and dried over anhydrous Na_2SO_4. The concentrated crude product was purified by flash column chromatography to afford the corresponding compounds **11**.

((2*S*,3*R*)-3-Ethynyl-3-methyl-1-tosylindolin-2-yl)(phenyl)methanone (5aa):

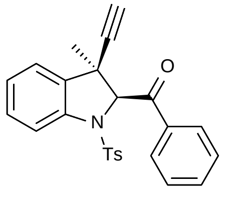

Following the general method **I**, compound **5aa** was obtained as a white solid (31.5 mg, Yield: 76%), m.p. = 139.6 – 140.4 °C. The enantiomeric excess (85% *ee*) was determined by chiral HPLC using CHIRALPAK® IC (*n*-hexane/isopropanol = 95.0/5.0, flow rate 1.0 mL/min, λ=254 nm) t (major) = 48.275 min, t (minor) = 68.258 min). $[\alpha]^{25}_D$ = +30.54 (c = 1.0, $CHCl_3$, 85% *ee*). **^{1}H NMR** (500 MHz, $CDCl_3$) δ 7.94 – 7.89 (m, 2H), 7.77 – 7.72 (m, 2H), 7.61 – 7.56 (m, 2H), 7.50 – 7.44 (m, 2H), 7.32 – 7.24 (m, 3H), 7.24 – 7.20 (m, 1H), 7.08 (td, *J* = 7.5, 1.0 Hz, 1H), 5.41 (s, 1H), 2.39 (s, 3H), 2.08 (s, 1H), 1.34 (s, 3H). **^{13}C NMR** (126 MHz, $CDCl_3$) δ 194.5, 144.5, 140.2, 136.5, 135.4, 135.1, 133.3, 129.8, 129.3, 128.9, 128.5, 127.2, 124.4, 123.8, 114.7, 83.5, 74.3, 74.2, 43.8, 31.9, 21.6. **IR (KBr)**: 3262, 1698, 1664, 1596, 1475, 1357, 1276, 1216, 1170, 1091, 968, 809, 757, 659, 570 cm^{-1}. **HRMS (ESI)** calculated for $C_{25}H_{21}NO_3SNa$ $[M+Na]^+$: 438.1140, found: 438.1133.

((2*S*,3*R*)-3-Ethynyl-6-fluoro-3-methyl-1-tosylindolin-2-yl)(phenyl)methanone (5ba):

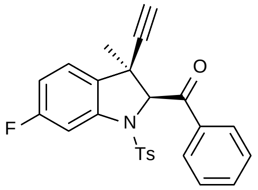

Following the general method **I**, compound **5ba** was obtained as a white solid (35.5 mg, Yield: 82%), m.p. = 136.0 – 136.6 °C. The enantiomeric excess (86% *ee*) was determined by chiral HPLC using CHIRALPAK® IC (*n*-hexane/isopropanol = 95.0/5.0, flow rate 1.0 mL/min, λ = 254 nm) t (major) = 22.600 min, t (minor) = 37.633 min). $[\alpha]^{25}_{D}$ = +24.06 (c = 1.3, $CHCl_3$, 86% *ee*). **^{1}H NMR** (500 MHz, $CDCl_3$) δ 7.95 – 7.88 (m, 2H), 7.73 – 7.79 (m, 2H), 7.63 – 7.57 (m, 1H), 7.45 – 7.52 (m, 2H), 7.34 – 7.27 (m, 3H), 7.14 (dd, *J* = 8.3, 5.4 Hz, 1H), 6.75 (td, *J* = 8.6, 2.4 Hz, 1H), 5.45 (s, 1H), 2.41 (s, 3H), 2.08 (s, 1H), 1.34 (s, 3H). **^{13}C NMR** (126 MHz, $CDCl_3$) δ 194.0, 163.5 (d, *J* = 245.6 Hz), 144.9, 141.6, 136.4, 135.0, 133.4, 130.9, 130.0, 128.8, 128.6, 127.2, 124.7, 111.0 (d, *J* = 23.2 Hz), 102.7 (d, *J* = 28.8 Hz), 83.1, 74.6, 74.5, 43.3, 31.9, 21.6. **^{19}F NMR** (282 MHz, $CDCl_3$) δ −111.80 – −112.10 (m, 1F). **IR (KBr)**: 3295, 1702, 1598, 1486, 1446, 1357, 1166, 1089, 987, 869, 813, 727, 665, 584, 543 cm^{-1}. **HRMS (ESI)** calculated for $C_{25}H_{20}FNO_3SNa$ $[M+Na]^+$: 456.1046, found: 456.1044.

((2*S*,3*R*)-3-Ethynyl-3-methyl-1-tosyl-6-(trifluoromethyl)indolin-2-yl)(phenyl) methanone (5ca):

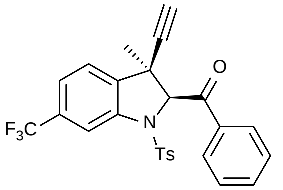

Following the general method **I**, compound **5ca** was obtained as a white solid (40.1 mg, Yield: 83%), m.p. = 171.3 – 171.9 °C. The enantiomeric excess (77% *ee*) was determined by chiral HPLC using CHIRALPAK® IB-IC (*n*-hexane/isopropanol = 95.0/5.0, flow rate 1.0 mL/min, λ = 254 nm) t (major) = 19.958 min, t (minor) = 21.775 min). $[\alpha]^{25}_{D}$ = +17.19 (c = 0.5, $CHCl_3$, 77% *ee*). **^{1}H NMR** (500 MHz, $CDCl_3$) δ 7.95 – 7.89 (m, 2H), 7.80 – 7.84 (m, 1H), 7.78 – 7.72 (m, 2H), 7.65 – 7.59 (m, 1H), 7.53 – 7.47 (m, 2H), 7.34 – 7.27 (m, 4H), 5.53 (s, 1H), 2.40 (s, 3H), 2.11 (s, 1H), 1.38 (s, 3H). **^{13}C NMR** (126 MHz, $CDCl_3$) δ 193.7, 145.0, 140.8, 139.1, 139.1, 136.3, 134.9, 133.6, 131.8 (q, *J* = 32.5 Hz), 130.0, 128.8, 128.7, 124.2, 123.8 (q, *J* = 272.6 Hz), 121.4 (q, *J* = 3.8 Hz), 111.3 (q, *J* = 3.9 Hz), 82.4, 75.0, 73.7, 43.8, 31.5, 21.6. **^{19}F NMR** (282 MHz, $CDCl_3$) δ −62.76 (s, 3F). **IR (KBr)**: 3309, 1700, 1598, 1438, 1363, 1321, 1272, 1168, 1124, 1087, 971, 823, 665, 576 cm^{-1}. **HRMS (ESI)** calculated for $C_{26}H_{20}F_3NO_3SNa$ $[M+Na]^+$: 506.1014, found: 506.0999.

((2*S*,3*R*)-5-Chloro-3-ethynyl-3-methyl-1-tosylindolin-2-yl)(phenyl)methanone (5da):

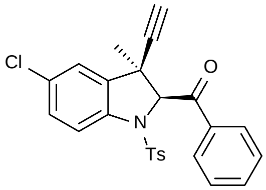

Following the general method **I**, compound **5da** was obtained as a white solid (26.9 mg, Yield: 60%), m.p. = 143.0 – 144.2 °C. The enantiomeric excess (79% *ee*) was determined by chiral HPLC using CHIRALPAK® IG (*n*-hexane/isopropanol = 95.0/5.0, flow rate 1.0 mL/min, λ = 254 nm) t (major) = 53.500 min, t (minor) = 74.108 min). $[\alpha]^{25}_{D}$ = +69.50 (c = 1.0, $CHCl_3$, 79% *ee*). **^{1}H NMR** (500 MHz, $CDCl_3$) δ 7.93 – 7.88 (m, 2H), 7.75 – 7.70 (m, 2H), 7.63 – 7.57 (m, 1H), 7.55 – 7.45 (m, 3H), 7.31 – 7.23 (m, 3H), 7.16 – 7.19 (m, 1H), 5.42 (s, 1H), 2.40 (s, 3H), 2.11 (s, 1H), 1.30 (s, 3H). **^{13}C NMR** (126 MHz, $CDCl_3$) δ 194.0, 144.8, 139.0, 137.3, 136.3, 134.9, 133.5, 130.0, 129.6, 129.4, 128.8, 128.6, 127.1, 124.2, 115.7, 82.6, 74.9, 74.0, 43.7, 31.7, 21.6. **IR (KBr)**: 3266, 1691, 1469, 1359, 1164, 1093, 817, 759, 665, 586, 547 cm^{-1}. **HRMS (ESI)** calculated for $C_{25}H_{20}NO_3SClNa$ $[M+Na]^+$: 472.0750, found: 472.0739.

((2*S*,3*R*)-3-Ethynyl-3,6-dimethyl-1-tosylindolin-2-yl)(phenyl)methanone (5ea):

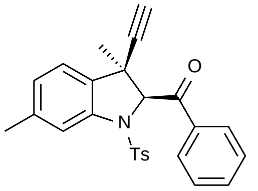

Following the general method **I**, compound **5ea** was obtained as a white solid (31.7 mg, Yield: 74%), m.p. = 147.6 – 149.2 °C. The enantiomeric excess (82% *ee*) was determined by chiral HPLC using CHIRALPAK® IC (*n*-hexane/isopropanol = 95.0/5.0, flow rate 1.0 mL/min, λ = 254 nm) t (major) = 40.158 min, t (minor) = 51.733 min). $[\alpha]^{25}_{D}$ = +49.36 (c = 1.76, $CHCl_3$, 82% *ee*). **^{1}H NMR** (500 MHz, $CDCl_3$) δ 7.93 – 7.88 (m, 2H), 7.78 – 7.72 (m, 2H), 7.61 – 7.55 (m, 1H), 7.49 – 7.41 (m, 3H), 7.30 – 7.23 (m, 2H), 7.11 – 7.07 (m, 1H), 6.91 – 6.86 (m, 1H), 5.37 (s, 1H), 2.39 (s, 6H), 2.06 (s, 1H), 1.30 (s, 3H). **^{13}C NMR** (126 MHz, $CDCl_3$) δ 194.5, 144.5, 140.3, 139.6, 136.5, 135.2, 133.2, 132.6, 129.8, 128.9, 128.5, 127.2, 125.3, 123.5, 115.3, 83.7, 74.5, 74.2, 43.6, 32.0, 21.8, 21.6. **IR (KBr)**: 3303, 1697, 1598, 1498, 1448, 1353, 1168, 1089, 809, 725, 665, 584, 543 cm^{-1}. **HRMS (ESI)** calculated for $C_{26}H_{23}NO_3SNa$ $[M+Na]^+$: 452.1296, found: 452.1291.

((2*S*,3*R*)-5-Bromo-3-ethynyl-3-methyl-1-tosylindolin-2-yl)(phenyl)methanone (5fa):

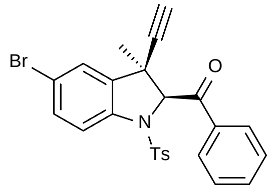

Following the general method **I**, compound **5fa** was obtained as a white solid (36.5 mg, Yield: 74%), m.p. = 152.8 – 154.2 °C. The enantiomeric excess (82% *ee*) was determined by chiral HPLC using CHIRALPAK® IF (*n*-hexane/isopropanol = 95.0/5.0, flow rate 1.0 mL/min, λ = 254 nm) t (major) = 34.792 min, t (minor) = 43.925 min). $[\alpha]^{25}_{D}$ = +91.87 (c = 0.9, $CHCl_3$, 82% *ee*). **^{1}H NMR** (500 MHz, $CDCl_3$) δ 7.93 – 7.88 (m, 2H), 7.75 – 7.71 (m, 2H), 7.63 – 7.58 (m, 1H), 7.51 – 7.46 (m, 3H), 7.42 – 7.38 (m, 1H), 7.33 – 7.31 (m, 1H), 7.30 – 7.27 (m, 2H), 5.41 (s, 1H), 2.40 (s, 3H), 2.11 (s, 1H), 1.31 (s, 3H). **^{13}C NMR** (126 MHz, $CDCl_3$) δ 193.9, 144.9, 139.5, 137.6, 136.3, 134.9, 133.5, 132.3, 130.0, 128.8, 128.6, 127.1 127.0, 116.9, 116.1, 82.6, 75.0, 73.9, 43.7, 31.7, 21.6. **IR (KBr)**: 3262, 1691, 1465, 1359, 1166, 1091, 809, 752, 663, 586, 545 cm^{-1}. **HRMS (ESI)** calculated for $C_{25}H_{20}NO_3SBrNa$ $[M+Na]^+$: 516.0245, found: 516.0248.

((2*S*,3*R*)-3-Ethyl-3-ethynyl-1-tosylindolin-2-yl)(phenyl)methanone (5ga):

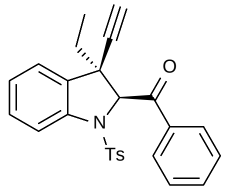

Following the general method **I**, compound **5ga** was obtained as a white solid (40.4 mg, Yield: 46%), m.p. = 109.5 – 110.5 °C. The enantiomeric excess (91% *ee*) was determined by chiral HPLC using CHIRALPAK® IC (*n*-hexane/isopropanol = 90.0/10.0, flow rate 1.0 mL/min, λ=254 nm) t (major) = 24.075 min, t (minor) = 31.683 min). $[\alpha]^{25}_{D}$ = +43.33 (c = 1.44, $CHCl_3$, 91% *ee*). **^{1}H NMR** (500 MHz, $CDCl_3$) δ 7.92 – 7.86 (m, 2H), 7.78 – 7.71 (m, 2H), 7.60 – 7.53 (m, 2H), 7.48 – 7.41 (m, 2H), 7.33 – 7.22 (m, 3H), 7.16 – 7.21 (m, 1H), 7.06 (td, *J* = 7.5, 1.0 Hz, 1H), 5.46 (s, 1H), 2.39 (s, 3H), 2.08 (s, 1H), 1.51 (dq, *J* = 14.7, 7.4 Hz, 1H), 1.40 (dq, *J* = 14.5, 7.3 Hz, 1H), 0.88 (t, *J* = 7.3 Hz, 3H). **^{13}C NMR** (126 MHz, $CDCl_3$) δ 195.0 144.5, 140.6, 136.6, 135.4, 134.1, 133.2, 129.8, 129.3, 128.9, 128.5, 127.1, 124.6, 124.0, 114.5, 82.1, 75.1, 71.9, 48.9, 36.5, 21.6, 8.8. **IR (KBr)**: 3268, 1693, 1596, 1475, 1359, 1218, 1168, 1093, 970, 811, 742, 684, 659, 578, 541 cm^{-1}. **HRMS (ESI)** calculated for $C_{26}H_{23}NO_3SNa$ $[M+Na]^+$: 452.1296, found: 452.1290.

((2*S*,3*R*)-3-Ethynyl-3-methyl-1-tosylindolin-2-yl)(4-methoxyphenyl)methanone (5ab):

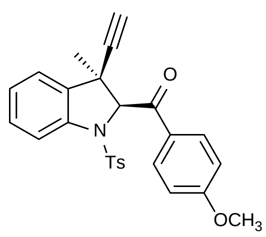

Following the general method **I**, compound **5ab** was obtained as a white solid (35.6 mg, Yield: 80%), m.p. = 149.3 – 151.0 °C. The enantiomeric excess (78% *ee*) was determined by chiral HPLC using CHIRALPAK® IF (*n*-hexane/isopropanol = 90.0/10.0, flow rate 1.0 mL/min, λ=254 nm) t (major) = 52.333 min, t (minor) = 77.925 min). $[\alpha]^{25}_{D}$ = +10.70 (c = 1.6, $CHCl_3$, 78% *ee*). **^{1}H NMR** (500 MHz, $CDCl_3$) δ 7.95 – 7.89 (m, 2H), 7.78 – 7.72 (m, 2H), 7.60 – 7.55 (m, 1H), 7.31 – 7.19 (m, 4H), 7.06 (td, *J* = 7.5, 0.9 Hz, 1H), 6.97 – 6.92 (m, 2H), 5.37 (s, 1H), 3.87 (s, 3H), 2.38 (s, 3H), 2.08 (s, 1H), 1.34 (s, 3H). **^{13}C NMR** (126 MHz, $CDCl_3$) δ 192.7, 163.7, 144.5, 140.3, 135.5, 135.2, 131.2, 129.8, 129.4, 129.3, 127.2, 124.4, 123.8, 114.6, 113.8, 83.5, 74.1, 74.0, 55.5, 43.9, 31.9, 21.6. **IR (KBr)**: 3276, 1683, 1600, 1471, 1357, 1255, 1170, 1085, 1027, 794, 759, 661, 574 cm^{-1}. **HRMS (ESI)** calculated for $C_{26}H_{23}NO_4SNa$ $[M+Na]^+$: 468.1245, found: 468.1243.

((2*S*,3*R*)-3-Ethynyl-3-methyl-1-tosylindolin-2-yl)(*p*-tolyl)methanone (5ac):

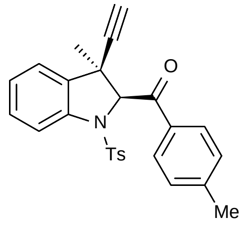

Following the general method **I**, compound **5ac** was obtained as a white solid (32.6 mg, Yield: 76%), m.p. = 141.1 – 142.6 °C. The enantiomeric excess (79% *ee*) was determined by chiral HPLC using CHIRALPAK® IG (*n*-hexane/isopropanol = 90.0/10.0, flow rate 1.5 mL/min, λ=254 nm) t (major) = 38.158 min, t (minor) = 63.133 min). $[\alpha]^{25}_{D}$ = +17.28 (c = 0.79, $CHCl_3$, 79% *ee*). **^{1}H NMR** (500 MHz, $CDCl_3$) δ 7.84 – 7.80 (m, 2H), 7.77 – 7.73 (m, 2H), 7.59 – 7.55 (m, 1H), 7.31 – 7.24 (m, 5H), 7.23 – 7.20 (m, 1H), 7.07 (td, *J* = 7.5, 1.0 Hz, 1H), 5.39 (s, 1H), 2.43 (s, 3H), 2.39 (s, 3H), 2.08 (s, 1H), 1.34 (s, 3H). **^{13}C NMR** (126 MHz, $CDCl_3$) δ 194.0, 144.5, 144.12 140.3, 135.5, 135.2, 134.0, 129.8, 129.3, 129.3, 129.0, 127.2, 124.4, 123.8, 114.6, 83.5, 74.2, 74.1, 43.9, 31.9, 21.8, 21.6. **IR (KBr)**: 3303, 1697, 1606, 1475, 1361, 1278, 1224, 1168, 1114, 1095, 1024, 970, 813, 757, 659, 566, 545 cm^{-1}. **HRMS (ESI)** calculated for $C_{26}H_{23}NO_3SNa$ $[M+Na]^+$: 452.1296, found: 452.1300.

((2*S*,3*R*)-3-Ethynyl-3-methyl-1-tosylindolin-2-yl)(4-nitrophenyl)methanone (5ad):

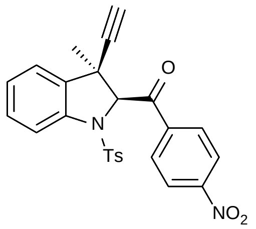

Following the general method **I**, compound **5ad** was obtained as a pale yellow solid (29.7 mg, Yield: 66%), m.p. = 87.2 – 88.5 °C. The enantiomeric excess (78% *ee*) was determined by chiral HPLC using CHIRALPAK® IB-IC (*n*-hexane/isopropanol = 90.0/10.0, flow rate 1.5 mL/min, λ=254 nm) t (major) = 40.633 min, t (minor) = 51.075 min). $[\alpha]^{25}_{D}$ = +52.50 (c = 1.4, $CHCl_3$, 78% *ee*). **^{1}H NMR** (500 MHz, $CDCl_3$) δ 8.30 – 8.26 (m, 2H), 8.04 – 7.99 (m, 2H), 7.74 – 7.69 (m, 2H), 7.66 – 7.61 (m, 1H), 7.38 – 7.32 (m, 1H), 7.30 – 7.23 (m, 3H), 7.14 (td, *J* = 7.5, 1.0 Hz, 1H), 5.12 (s, 1H), 2.40 (s, 3H), 2.16 (s, 1H), 1.33 (s, 3H). **^{13}C NMR** (126 MHz, $CDCl_3$) δ 194.4, 150.1, 145.0, 141.1, 139.8, 134.9, 134.2, 130.0, 129.9, 129.8, 127.2, 125.0, 124.1, 123.6, 115.0, 83.4, 76.0, 75.1, 43.9, 32.3, 21.6. **IR (KBr)**: 3278, 1708, 1600, 1525, 1346, 1166, 1091, 740, 661, 584, 570 cm^{-1}. **HRMS (ESI)** calculated for $C_{25}H_{20}N_2O_5SNa$ $[M+Na]^+$: 483.0991, found: 483.0993.

((2*S*,3*R*)-3-Ethynyl-3-methyl-1-tosylindolin-2-yl)(4-fluorophenyl)methanone (5ae):

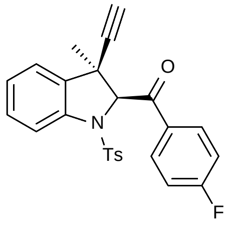

Following the general method **I**, compound **5ae** was obtained as a white solid (32.0 mg, Yield: 74%), m.p. = 157.3 – 158.7 °C. The enantiomeric excess (78% *ee*) was determined by chiral HPLC using CHIRALPAK® IC (*n*-hexane/isopropanol = 95.0/5.0, flow rate 1.0 mL/min, λ=254 nm) t (major) = 36.967 min, t (minor) = 57.000 min). $[\alpha]^{25}_{D}$ = +51.62 (c = 1.0, $CHCl_3$, 78% *ee*). **^{1}H NMR** (500 MHz, $CDCl_3$) δ 7.97 – 7.92 (m, 2H), 7.77 – 7.72 (m, 2H), 7.62 – 7.57 (m, 1H), 7.34 – 7.20 (m, 4H), 7.17 – 7.11 (m, 2H), 7.09 (td, *J* = 7.5, 1.0 Hz, 1H), 5.29 (s, 1H), 2.39 (s, 3H), 2.10 (s, 1H), 1.33 (s, 3H). **^{13}C NMR** (126 MHz, $CDCl_3$) δ 193.2, 165.8 (d, *J* = 255.3 Hz), 144.7, 140.1, 135.3, 134.9, 132.8, 131.6 (d, *J* = 9.3 Hz), 129.9, 129.4, 127.2, 124.6, 123.9, 115.7 (d, *J* = 22.0 Hz), 114.7, 83.4, 74.6, 74.5, 43.8, 32.0, 21.6. **^{19}F NMR** (282 MHz, $CDCl_3$) δ −105.04 – −105.27 (m, 1F). **IR (KBr)**: 3297, 1704, 1596, 1481, 1361, 1222, 1164, 1087, 1000, 958, 755, 657, 578 cm^{-1}. **HRMS (ESI)** calculated for $C_{25}H_{20}FNO_3SNa$ $[M+Na]^+$: 456.1046, found: 456.1046.

((2*S*,3*R*)-3-Ethynyl-3-methyl-1-tosylindolin-2-yl)(4-(trifluoromethyl)phenyl)methanone (5af):

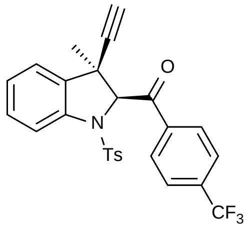

Following the general method **I**, compound **5af** was obtained as a white solid (39.6 mg, Yield: 82%), m.p. = 176.4 – 177.0 °C. The enantiomeric excess (74% *ee*) was determined by chiral HPLC using CHIRALPAK® IG (*n*-hexane/isopropanol = 90.0/10.0, flow rate 1.5 mL/min, λ=254 nm) t (major) = 12.625 min, t (minor) = 16.675 min). $[\alpha]^{25}_{D}$ = +51.62 (c = 1.73, $CHCl_3$, 74% *ee*). **^{1}H NMR** (500 MHz, $CDCl_3$) δ 8.01 – 7.96 (m, 2H), 7.75 – 7.69 (m, 4H), 7.60 – 7.64 (m, 1H), 7.36 – 7.30 (m, 1H), 7.30 – 7.22 (m, 3H), 7.11 (td, *J* = 7.5, 1.0 Hz, 1H), 5.23 (s, 1H), 2.39 (s, 3H), 2.13 (s, 1H), 1.33 (s, 3H). **^{13}C NMR** (126 MHz, $CDCl_3$) δ 194.4, 144.8, 140.0, 139.2, 135.1, 134.6, 134.3 (q, *J* = 32.7 Hz), 129.9, 129.6, 129.2, 127.2, 125.5 (q, *J* = 3.7 Hz), 124.8, 124.0, 123.6 (q, *J* = 272.8 Hz), 114.9, 83.4, 75.3, 74.8, 43.9, 32.2, 21.6. **IR (KBr)**: 3318, 1702, 1598, 1477, 1359, 1321, 1168, 1124, 1064, 757, 659, 586 cm^{-1}. **^{19}F NMR** (282 MHz, $CDCl_3$) δ −63.60 (s, 3F). **HRMS (ESI)** calculated for $C_{26}H_{20}NO_3F_3SNa$ $[M+Na]^+$: 506.1014, found: 506.1016.

(4-Bromophenyl)((2*S*,3*R*)-3-ethynyl-3-methyl-1-tosylindolin-2-yl)methanone (5ag):

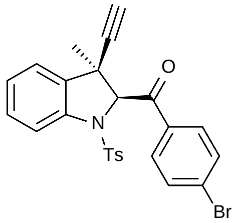

Following the general method **I**, compound **5ag** was obtained as a white solid (38.4 mg, Yield: 78%), m.p. = 180.4 – 181.3 °C. The enantiomeric excess (79% *ee*) was determined by chiral HPLC using CHIRALPAK® IC (*n*-hexane/isopropanol = 95.0/5.0, flow rate 1.0 mL/min, λ=254 nm) t (major) = 38.225 min, t (minor) = 73.358 min). $[\alpha]^{25}_{D}$ = +17.47 (c = 0.7, $CHCl_3$, 79% *ee*). **^{1}H NMR** (500 MHz, $CDCl_3$) δ 7.79 – 7.71 (m, 4H), 7.62 – 7.57 (m, 3H), 7.34 – 7.20 (m, 4H), 7.09 (td, *J* = 7.5, 1.0 Hz, 1H), 5.24 (s, 1H), 2.39 (s, 3H), 2.11 (s, 1H), 1.33 (s, 3H). **^{13}C NMR** (126 MHz, $CDCl_3$) δ 193.9, 144.7, 140.0, 135.2, 135.1, 134.8, 131.8, 130.4, 129.9, 129.5, 128.5, 127.2, 124.6, 123.9, 114.8, 83.4, 74.8, 74.6, 43.8, 32.0, 21.6. **IR (KBr)**: 3293, 1697, 1585, 1477, 1357, 1220, 1166, 1093, 1006, 962, 813, 754, 661, 570 cm^{-1}. **HRMS (ESI)** calculated for $C_{25}H_{20}NO_3SBrNa$ $[M+Na]^+$: 516.0245, found: 516.0242.

((2*S*,3*R*)-3-ethynyl-3-methyl-1-tosylindolin-2-yl)(thiophen-2-yl)methanone (5ah):

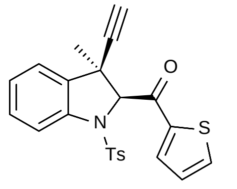

Following the general method **I**, compound **5ah** was obtained as a white solid (33.7 mg, Yield: 80%), m.p. = 80.4 – 81.3 °C. The enantiomeric excess (80% *ee*) was determined by chiral HPLC using CHIRALPAK® IA (*n*-hexane/isopropanol = 95.0/5.0, flow rate 1.0 mL/min, λ=254 nm) t (minor) = 51.758 min, t (major) = 77.533 min). $[\alpha]^{25}_{D}$ = +30.35 (c = 2.4, $CHCl_3$, 80% *ee*). **^{1}H NMR** (500 MHz, $CDCl_3$) δ 7.82 – 7.78 (m, 1H), 7.77 – 7.72 (m, 2H), 7.70 – 7.65 (m, 2H), 7.33 – 7.29 (m, 1H), 7.29 – 7.24 (m, 2H), 7.23 – 7.19 (m, 1H), 7.14 – 7.06 (m, 2H), 5.02 (s, 1H), 2.39 (s, 3H), 2.15 (s, 1H), 1.30 (s, 3H). **^{13}C NMR** (126 MHz, $CDCl_3$) δ 187.0, 144.7, 141.9, 140.1, 135.4, 134.7, 134.3, 133.2, 129.8, 129.4, 128.1, 127.3, 124.8, 124.0, 115.0, 83.1, 76.2, 74.2, 44.2, 32.2, 21.6. **IR (KBr)**: 3288, 1670, 1602, 1471, 1411, 1363, 1168, 1093, 750, 730, 574 cm^{-1}. **HRMS (ESI)** calculated for $C_{23}H_{20}NO_3S_2$ $[M+H]^+$: 422.0885, found: 422.0869.

Cyclohexyl((2*S*,3*R*)-3-ethynyl-3-methyl-1-tosylindolin-2-yl)methanone (5ai):

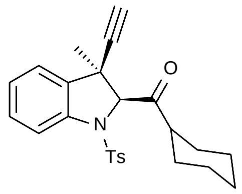

Following the general method **I**, compound **5ai** was obtained as a white solid (28.6 mg, Yield: 68%), m.p. = 118.9 – 120.3 °C. The enantiomeric excess (62% *ee*) was determined by chiral HPLC using CHIRALPAK® IG (*n*-hexane/isopropanol = 95.0/5.0, flow rate 1.0 mL/min, λ=254 nm) t (minor) = 50.825 min, t (major) = 55.658 min). $[\alpha]^{25}_{D}$ = +41.72 (c = 1.7, $CHCl_3$, 62% *ee*). **^{1}H NMR** (500 MHz, $CDCl_3$) δ 7.72 – 7.66 (m, 2H), 7.66 – 7.61 (m, 1H), 7.32 – 7.22 (m, 3H), 7.22 – 7.16 (m, 1H), 7.08 (td, *J* = 7.5, 1.0 Hz, 1H), 4.56 (s, 1H), 2.64 (tt, *J* = 11.4, 3.3 Hz, 1H), 2.37 (s, 3H), 2.03 – 1.95 (m, 1H), 1.95 – 1.87 (m, 1H), 1.84 – 1.70 (m, 2H), 1.69 – 1.60 (m, 1H), 1.55 – 1.43 (m, 1H), 1.38 – 1.15 (m, 4H), 1.07 (s, 3H). **^{13}C NMR** (126 MHz, $CDCl_3$) δ 207.6, 144.6, 140.1, 135.8, 134.8, 129.8, 129.3, 127.1, 124.8, 123.7, 115.3, 83.8, 77.2, 74.1, 48.5, 43.5, 32.6, 28.9, 28.1, 25.9, 25.8, 25.5, 21.6. **IR (KBr)**: 3282, 2931, 2848, 1722, 1598, 1471, 1452, 1359, 1166, 1097, 754, 663, 574 cm^{-1}. **HRMS (ESI)** calculated for $C_{25}H_{27}NO_3SNa$ $[M+Na]^+$: 444.1609, found: 444.1613.

(4-Bromophenyl)((2*S*,3*R*)-3-ethyl-3-ethynyl-1-tosylindolin-2-yl)methanone (5gg):

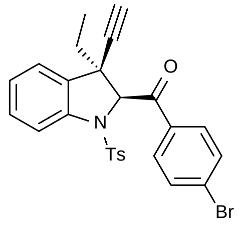

Following the general method **I**, compound **5gg** was obtained as a white solid (42.6 mg, Yield: 42%), m.p. = 160.6 – 161.2 °C. The enantiomeric excess (91% *ee*) was determined by chiral HPLC using CHIRALPAK® IC (*n*-hexane/isopropanol = 95.0/5.0, flow rate 1.0 mL/min, λ=254 nm) t (major) = 26.925 min, t (minor) = 49.192 min). $[\alpha]^{25}_{D}$ = +23.45 (c = 0.65, $CHCl_3$, 91% *ee*). **^{1}H NMR** (500 MHz, $CDCl_3$) δ 7.76 – 7.70 (m, 4H), 7.62 – 7.55 (m, 3H), 7.34 – 7.28 (m, 1H), 7.28 – 7.23 (m, 2H), 7.22 – 7.17 (m, 1H), 7.08 (td, *J* = 7.5, 1.0 Hz, 1H), 5.29 (s, 1H), 2.39 (s, 3H), 2.12 (s, 1H), 1.51 (dq, *J* = 14.6, 7.3 Hz, 1H), 1.36 (dq, *J* = 14.5, 7.3 Hz, 1H), 0.85 (t, *J* = 7.3 Hz, 3H). **^{13}C NMR** (126 MHz, $CDCl_3$) δ 194.4, 144.7, 140.4, 135.2, 135.0, 133.9, 131.8, 130.4, 129.8, 129.5, 128.3, 127.1, 124.7, 124.2, 114.6, 82.1, 75.4, 72.4, 48.8, 36.7, 21.6, 8.7. **IR (KBr)**: 3288, 1695, 1589, 1467, 1359, 1216, 1166, 1091, 1008, 754, 659, 572 cm^{-1}. **HRMS (ESI)** calculated for $C_{26}H_{22}NO_3SBrNa$ $[M+Na]^+$: 530.0401, found: 530.0403.

1-Phenyl-3-(1-tosyl-3-(trifluoromethyl)-1*H*-indol-2-yl) prop-2-en-1-one (6aa):

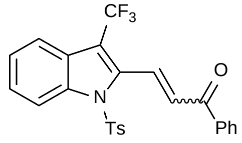

Following the general method **J**, the purification by column chromatography on silica gel (Toluene) to give **6aa** (37.0 mg, Yield: 79%) as a light yellow solid, m.p. = 108.4 – 109.4 °C. The ratio for *E/Z* isomers (4.4:1) was determined by ^{19}F NMR. (*E*)-**6aa**: **^{1}H NMR** (500 MHz, $CDCl_3$) δ 8.33 (d, *J* = 8.6 Hz, 1H), 8.08 (d, *J* = 16.0 Hz, 1H), 8.04 – 7.97 (m, 2H), 7.77 – 7.73 (m, 1H), 7.72 – 7.66 (m, 2H), 7.65 – 7.60 (m, 1H), 7.56 – 7.50 (m, 2H), 7.49 – 7.44 (m, 1H), 7.40 – 7.36 (m, 1H), 7.28 (d, *J* = 15.9 Hz, 1H), 7.22 – 7.16 (m, 2H), 2.33 (s, 3H). **^{13}C NMR** (126 MHz, $CDCl_3$) δ 189.2, 146.0, 137.1, 136.2, 134.9, 133.5, 132.1, 131.0, 130.1, 129.8, 128.8, 127.4, 127.0, 126.7, 125.7, 124.9, 123.3 (q, *J* = 269.8 Hz), 120.7, 114.9, 112.9 (q, *J* = 35.4 Hz), 21.6. **^{19}F NMR** (282 MHz, $CDCl_3$) δ −54.39 (s, 3F). **IR (KBr)**: 3018, 2944, 2884, 1672, 1613, 1597, 1450, 1394, 1291, 1234, 1177, 1120, 1089, 974, 812, 746, 702, 671, 575 cm^{-1}. **HRMS (ESI)** calculated for $C_{25}H_{18}F_3NO_3SNa$ $[M+Na]^+$: 492.0857, found: 492.0862.

1-(4-Methoxyphenyl)-3-(1-tosyl-3-(trifluoromethyl)-1*H*-indol-2-yl)prop-2-en-1-one (6ab):

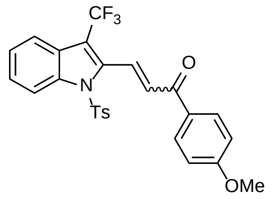

Following the general method **J**, the purification by column chromatography on silica gel (Toluene) to give **6ab** (39.5 mg, Yield: 79%) as a light yellow solid, m.p. = 111.7 – 113.4 °C. The ratio for *E/Z* isomers (5.4:1) was determined by ^{19}F NMR. (*E*)-**6ab**: **^{1}H NMR** (500 MHz, $CDCl_3$) δ 8.33 (d, *J* = 8.6 Hz, 1H), 8.05 (d, *J* = 15.5 Hz, 1H), 8.03 – 7.99 (m, 2H), 7.77 – 7.72 (m, 1H), 7.72 – 7.67 (m, 2H), 7.50 – 7.43 (m, 1H), 7.39 – 7.34 (m, 1H), 7.28 (d, *J* = 16.4 Hz, 1H), 7.23 – 7.17 (m, 2H), 7.04 – 6.98 (m, 2H), 3.90 (s, 3H), 2.34 (s, 3H). **^{13}C NMR** (126 MHz, $CDCl_3$) δ 187.4, 163.9, 146.0, 136.4, 136.2, 135.0, 132.2, 131.2, 130.1, 130.0, 127.5, 127.1, 126.6, 125.6, 124.8, 123.4 (d, *J* = 269.8 Hz), 120.6, 114.9, 114.1, 112.6 (q, *J* = 35.3 Hz), 55.6, 21.7. **^{19}F NMR** (282 MHz, $CDCl_3$) δ −54.39(s, 3F). **IR (KBr)**: 3032, 2960, 2930, 2876, 2843, 1666, 1599, 1512, 1450, 1396, 1378, 1253, 1238, 1171, 1118, 1062, 1029, 745, 668, 573 cm^{-1}. **HRMS (ESI)** calculated for $C_{26}H_{20}F_3NO_4SNa$ [M+Na]$^+$: 522.0963, found: 522.0970.

1-(*p*-Tolyl)-3-(1-tosyl-3-(trifluoromethyl)-1*H*-indol-2-yl)prop-2-en-1-one (6ac):

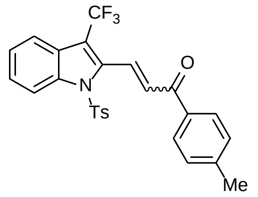

Following the general method **J**, the purification by column chromatography on silica gel (Toluene) to give **6ac** (35.3 mg, Yield: 73%) as a light yellow solid, m.p. = 127.7 – 129.4 °C. The ratio for *E/Z* isomers (5.3:1) was determined by ^{19}F NMR. (*E*)-**6ac**: **^{1}H NMR** (500 MHz, $CDCl_3$) δ 8.34 (d, *J* = 8.5 Hz, 1H), 8.08 (dd, *J* = 15.9, 1.3 Hz, 1H), 7.96 – 7.89 (m, 2H), 7.77 – 7.73 (m, 1H), 7.72 – 7.67 (m, 2H), 7.50 – 7.44 (m, 1H), 7.39 – 7.34 (m, 1H), 7.35 – 7.31 (m, 2H), 7.29 (d, *J* = 15.9 Hz, 1H), 7.22 – 7.17 (m, 2H), 2.45 (s, 3H), 2.33 (s, 3H). **^{13}C NMR** (126 MHz, $CDCl_3$) δ 188.6, 146.0, 144.5, 136.2, 135.0, 134.6, 132.2, 130.5, 130.0, 129.6, 128.9, 127.4, 127.0, 126.6, 125.6, 124.8, 123.1 (q, *J* = 269.8 Hz), 120.6, 114.8, 112.6 (q, *J* = 35.4 Hz), 21.8, 21.6. **^{19}F NMR** (282 MHz, $CDCl_3$) δ −54.38 (s, 3F). **IR (KBr)**: 3055, 2957, 2923, 2866, 1668, 1604, 1450, 1396, 1294, 1236, 1176, 1120, 1089, 1028, 748, 669, 575 cm^{-1}. **HRMS (ESI)** calculated for $C_{26}H_{20}F_3NO_3SNa$ [M+Na]$^+$: 506.1014, found: 506.1010.

1-(4-Nitrophenyl)-3-(1-tosyl-3-(trifluoromethyl)-1*H*-indol-2-yl)prop-2-en-1-one (6ad):

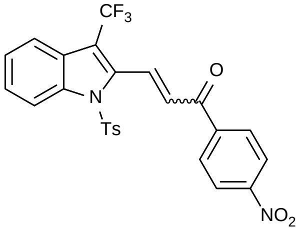

Following the general method **J**, the purification by column chromatography on silica gel (Toluene) to give **6ad** (36.0 mg, Yield: 70%) as a light yellow solid, m.p. = 183.8 – 186.5 °C. The ratio for *E/Z* isomers (5.7:1) was determined by ^{19}F NMR. (*E*)-**6ad**: **^{1}H NMR** (500 MHz, $CDCl_3$) δ 8.38 (d, *J* = 8.8 Hz, 2H), 8.30 (d, *J* = 8.3 Hz, 1H), 8.16 (d, *J* = 8.8 Hz, 2H), 8.13 (d, *J* = 16.1 Hz, 1H), 7.75 (d, *J* = 8.1 Hz, 1H), 7.68 (d, *J* = 8.5 Hz, 2H), 7.49 (t, *J* = 7.8 Hz, 1H), 7.38 (t, *J* = 7.6 Hz, 1H), 7.29 (d, *J* = 15.9 Hz, 1H), 7.23 (d, *J* = 8.2 Hz, 2H), 2.35 (s, 3H). **^{13}C NMR** (126 MHz, $CDCl_3$) δ 188.0, 150.4, 146.2, 141.7, 136.3, 135.3, 134.7, 132.7, 131.1, 130.2, 129.7, 127.1, 126.9, 125.6, 125.1, 124.3, 124.0, 123.2 (q, *J* = 270.0 Hz), 120.9, 115.0, 113.5 (q, *J* = 35.4 Hz), 21.7. **^{19}F NMR** (282 MHz, $CDCl_3$) δ −54.34 (s, 3F). **IR (KBr)**: 3033, 2937, 2855, 1721, 1676, 1601, 1527, 1450, 1434, 1394, 1348, 1305, 1248, 1176, 1120, 1064, 1027, 996, 852, 824, 748, 667, 575 cm^{-1}. **HRMS (ESI)** calculated for $C_{25}H_{17}F_3N_2O_5SNa$ [M+Na]$^+$: 537.0708, found: 537.0712.

1-(Thiophen-2-yl)-3-(1-tosyl-3-(trifluoromethyl)-1*H*-indol-2-yl)prop-2-en-1-one (6ah):

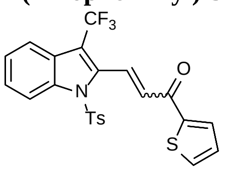

Following the general method **J**, the purification by column chromatography on silica gel (Toluene) to give **6ah** (38.1 mg, Yield: 80%) as a light yellow solid, m.p. = 132.8 – 134.2 °C. The ratio for *E/Z* isomers (6.9:1) was determined by ^{19}F NMR. (*E*)-**6ah**: **^{1}H NMR** (500 MHz, $CDCl_3$) δ 8.34 (d, *J* = 8.6 Hz, 1H), 8.11 (dd, *J* = 15.8, 1.3 Hz, 1H), 7.85 – 7.80 (m, 1H), 7.78 – 7.73 (m, 2H), 7.72 – 7.68 (m, 2H), 7.50 – 7.44 (m, 1H), 7.39 – 7.35 (m, 1H), 7.23 – 7.20 (m, 3H), 7.18 (d, *J* = 15.9 Hz, 1H), 2.34 (s, 3H). **^{13}C NMR** (126 MHz, $CDCl_3$) δ 180.9, 146.1, 144.6, 136.3, 135.9, 135.1, 134.9, 132.9, 131.0, 130.2, 130.1, 128.5, 127.0, 126.7, 125.6, 124.9, 123.3 (q, *J* = 269.8 Hz), 120.7, 114.9, 112.8 (q, *J* = 35.4 Hz), 21.6. **^{19}F NMR** (282 MHz, $CDCl_3$) δ −54.45 (s, 3F). **IR (KBr)**: 3029, 2927, 2855, 1658, 1610, 1597, 1514, 1450, 1414, 1355, 1292, 1238, 1176, 1120, 1089, 1063, 1030, 970, 814, 746, 671, 574, 540 cm^{-1}. **HRMS (ESI)** calculated for $C_{23}H_{16}F_3NO_3S_2Na$ [M+Na]$^+$: 498.0421, found: 498.0421.

1-Cyclohexyl-3-(1-tosyl-3-(trifluoromethyl)-1*H*-indol-2-yl)prop-2-en-1-one (6ai):

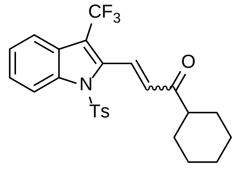

Following the general method **J**, the purification by column chromatography on silica gel (Toluene) to give **6ai** (29.0 mg, Yield: 61%) as a light yellow solid, m.p. = 103.5 – 104.9 °C. The ratio for *E/Z* isomers (5.0: 1) was determined by ^{19}F NMR. (*E*)-**6ai**: **^{1}H NMR** (500 MHz, $CDCl_3$) δ 8.29 (d, *J* = 8.5 Hz, 1H), 7.90 (dd, *J* = 16.2, 1.4 Hz, 1H), 7.74 – 7.68 (m, 1H), 7.67 – 7.62 (m, 2H), 7.48 – 7.42 (m 1H), 7.38 – 7.32 (m, 1H), 7.25 – 7.20 (m, 2H), 6.58 – 6.50 (m, 1H), 2.73 – 2.64 (m, 1H), 2.36 (s, 3H), 1.99 – 1.90 (m, 2H), 1.88 – 1.81 (m, 2H), 1.77 – 1.69 (m, 2H), 1.52 – 1.32 (m, 4H). **^{13}C NMR** (126 MHz, $CDCl_3$) δ 201.7, 146.0, 136.1, 134.9, 134.2, 130.0, 129.1, 127.4, 126.9, 126.6, 125.6, 124.8, 123.2 (q, *J* = 269.8 Hz), 120.7 (d, *J* = 2.6 Hz), 114.8, 112.7 (q, *J* = 35.4 Hz), 49.1, 28.3, 25.9, 25.6, 21.7. **^{19}F NMR** (282 MHz, $CDCl_3$) δ −54.60 (s, 3F). **IR (KBr)**: 3018, 2931, 2856, 1693, 1670, 1622, 1596, 1568, 1450, 1394, 1378, 1293, 1247, 1176, 1120, 1030, 977, 746, 703, 671, 575 cm^{-1}. **HRMS (ESI)** calculated for $C_{25}H_{24}F_3NO_3SNa$ $[M+Na]^+$: 498.1327, found: 498.1325.

tert-Butyl 3-(1-tosyl-3-(trifluoromethyl)-1*H*-indol-2-yl)acrylate (6aj):

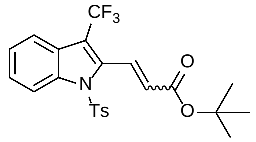

Following the general method **J**, the purification by column chromatography on silica gel (Toluene) to give **6aj** (20.7 mg, Yield: 44%) as a colorless oil. The ratio for *E/Z* isomers (1.44: 1) was determined by ^{19}F NMR. (*E*)-**6aj**: **^{1}H NMR** (500 MHz, $CDCl_3$) δ 8.32 – 8.26 (m, 1H), 7.94 (dd, *J* = 16.0, 1.2 Hz, 1H), 7.74 – 7.69 (m, 1H), 7.69 – 7.66 (m, 2H), 7.47 – 7.41 (m, 1H), 7.37 – 7.32 (m, 1H), 7.25 – 7.20 (m, 2H), 6.13 (dd, *J* = 16.1, 1.0 Hz, 1H), 2.37 (s, 3H), 1.58 (s, 9H). **^{13}C NMR** (126 MHz, $CDCl_3$) δ 164.4, 146.0, 135.5, 135.2, 135.0, 130.6, 130.3, 130.0, 129.4, 127.0, 126.5, 124.7, 123.1 (q, *J* = 269.8 Hz).120.6, 114.8, 112.4 (q, *J* = 35.5 Hz), 81.5, 28.2, 21.7. **^{19}F NMR** (282 MHz, $CDCl_3$) δ −54.71 (s, 3F). **IR (KBr)**: 2985, 1716, 1394, 1243, 1157, 1116, 1060, 667, 574 cm^{-1}. **HRMS (ESI)** calculated for $C_{23}H_{22}F_3NO_4SNa$ $[M+Na]^+$: 488.1119, found: 488.1114.

3-(5-Fluoro-1-tosyl-3-(trifluoromethyl)-1*H*-indol-2-yl)-1-(*p*-tolyl)prop-2-en-1-one (6bc):

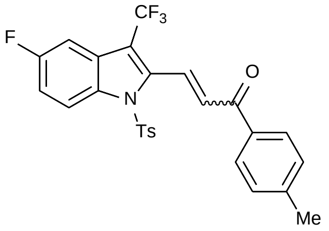

Following the general method **J**, the purification by column chromatography on silica gel (Toluene) to give **6bc** (29.8 mg, Yield: 60%) as a light yellow solid, m.p. = 123.4 – 125.0 °C. The ratio for *E/Z* isomers (2.2:1) was determined by ^{19}F NMR. (*E*)-**6bc**: **^{1}H NMR** (500 MHz, $CDCl_3$) δ 8.30 (dd, *J* = 9.3, 4.4 Hz, 1H), 8.02 (dd, *J* = 15.9, 1.2 Hz, 1H), 7.91 (d, *J* = 8.2 Hz, 2H), 7.67 (d, *J* = 8.5 Hz, 2H), 7.42 – 7.37 (m, 1H), 7.36 – 7.31 (m, 2H), 7.30 – 7.27 (m, 1H), 7.23 – 7.20 (m, 2H), 7.19 (td, *J* = 9.1, 2.6 Hz, 1H), 2.46 (s, 3H), 2.35 (s, 3H). **^{13}C NMR** (126 MHz, $CDCl_3$) δ 188.4, 160.1 (d, *J* = 242.8 Hz), 145.5 (d, *J* = 208.1 Hz), 137.8, 134.5, 132.6, 130.1, 130.0, 129.8, 129.6, 129.4, 128.9, 128.5, 127.5, 127.1, 123.1 (q, *J* = 269.6 Hz), 116.3, 114.9 (d, *J* = 25.4 Hz), 112.4 (q, *J* = 35.7 Hz), 106.3 (dq, *J* = 25.7, 2.7 Hz), 21.8, 21.7. **^{19}F NMR** (282 MHz, $CDCl_3$) δ −54.64 (s, 3F), −117.01 – −117.63 (m, 1F). **IR (KBr)**: 3056, 3020, 2960, 2925, 2866, 2358, 2341, 1670, 1606, 1570, 1471, 1452, 1392, 1303, 1269, 1173, 1118, 1061, 806, 667, 697 cm^{-1}. **HRMS (ESI)** calculated for $C_{26}H_{19}F_4NO_3SNa$ $[M+Na]^+$: 524.0919, found: 524.0920.

3-(5-Fluoro-1-tosyl-3-(trifluoromethyl)-1*H*-indol-2-yl)-1-(4-nitrophenyl)prop-2-en-1-one (**6bd**):

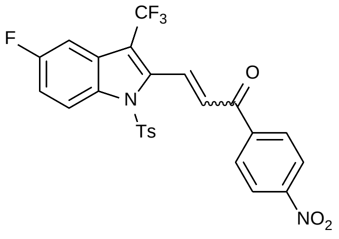

Following the general method **J**, the purification by column chromatography on silica gel (Toluene) to give **6bd** (32.1 mg, Yield: 60%) as a light yellow solid. m.p. = 149.9 – 151.3 °C. The ratio for *E/Z* isomers (4.2:1) was determined by ^{19}F NMR. (*E*)-**6bd**: **^{1}H NMR** (500 MHz, $CDCl_3$) δ 8.41 – 8.35 (m, 2H), 8.27 (dd, *J* = 9.3, 4.4 Hz, 1H), 8.18 – 8.13 (m, 2H), 8.09 (dd, *J* = 15.9, 1.3 Hz, 1H), 7.68 – 7.63 (m, 2H), 7.42 – 7.38 (m, 1H), 7.29 (d, *J* = 16.0, 1H), 7.26 – 7.22 (m, 2H), 7.20 (td, *J* = 9.0, 2.8 Hz, 1H), 2.37 (s, 3H). **^{13}C NMR** (126 MHz, $CDCl_3$) δ 187.9, 160.2 (d, *J* = 243.6 Hz), 150.5, 146.5, 141.6, 136.8, 134.5, 132.6, 132.2, 131.4, 130.3, 129.8, 127.4, 126.9, 124.1, 123.0 (q, *J* = 269.9 Hz), 116.4, 115.4 (d, *J* = 25.4 Hz), 113.2 (qd, *J* = 35.7, 4.3 Hz), 106.5 (dq, *J* = 25.7, 2.7 Hz), 21.7. **^{19}F NMR** (282 MHz, $CDCl_3$) δ −54.54 (s, 3F), −116.49 – −116.95 (m, 1F). **IR (KBr)**: 3022, 2927, 2852, 1676, 1617, 1599, 1527, 1475, 1451, 1392, 1348, 1172, 1118, 1087, 1062, 1010, 973, 935, 850, 812, 665 cm^{-1}. **HRMS (ESI)** calculated for $C_{25}H_{16}F_4N_2O_5SNa$ $[M+Na]^+$: 555.0614, found: 555.060.

1-(4-Bromophenyl)-3-(5-fluoro-1-tosyl-3-(trifluoromethyl)-1*H*-indol-2-yl)prop-2-en-1-one (6bg):

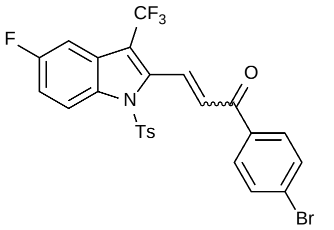

Following the general method **J**, the purification by column chromatography on silica gel (Toluene) to give **6bg** (39.8 mg, Yield: 70%) as a light yellow solid, m.p. = 135.0 – 136.9 °C. The ratio for *E/Z* isomers (1.8:1) was determined by ^{19}F NMR. (*E*)-**6bg**: **^{1}H NMR** (500 MHz, $CDCl_3$) δ 8.29 (dd, *J* = 9.3, 4.4 Hz, 1H), 8.04 (dd, *J* = 15.9, 1.3 Hz, 1H), 7.90 – 7.85 (m, 2H), 7.69 – 7.67 (m, 2H), 7.67 – 7.64 (m, 2H), 7.42 – 7.37 (m, 1H), 7.30 (s, 1H), 7.25 – 7.21 (m, 2H), 7.21 (td, *J* = 9.0, 2.7 Hz, 1H), 2.36 (s, 3H). **^{13}C NMR** (126 MHz, $CDCl_3$) δ 188.0, 160.2 (d, *J* = 243.2 Hz), 146.4, 137.4, 135.7, 134.6, 132.2, 132.1, 131.9, 131.0, 130.2, 129.9, 128.9, 127. 4, 127.0, 123.1 (q, *J* = 269.8 Hz), 116.4, 115.2 (d, *J* = 25.5 Hz), 112.7 (q, *J* = 35.6 Hz), 106.4 (dd, *J* = 25.6, 2.7 Hz), 21.7. **^{19}F NMR** (282 MHz, $CDCl_3$) δ −54.59 (s, 3F), −116.88 – −117.23 (m, 1F). **IR (KBr)**: 3036, 2997, 2930, 2870, 1672, 1615, 1587, 1475, 1453, 1394, 1301, 1270, 1172, 1120, 1063, 1007, 934, 859, 837, 812, 665, 577, 545 cm^{-1}. **HRMS (ESI)** calculated for $C_{25}H_{16}BrF_4NO_3SNa$ $[M+Na]^+$: 587.9868, found: 587.9858.

3-(5-Fluoro-1-tosyl-3-(trifluoromethyl)-1*H*-indol-2-yl)-1-(thiophen-2-yl)prop-2-en-1-one (6bh):

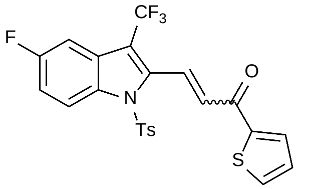

Following the general method **J**, the purification by column chromatography on silica gel (Toluene) to give **6bh** (39.4 mg, Yield: 80%) as a light yellow solid, m.p. = 139.9 – 142.5 °C. The ratio for *E/Z* isomers (4.3:1) was determined by ^{19}F NMR. (*E*)-**6bh**:**^{1}H NMR** (500 MHz, $CDCl_3$) δ 8.30 (dd, *J* = 9.3, 4.4 Hz, 1H), 8.07 (dd, *J* = 15.8, 1.3 Hz, 1H), 7.82 (dd, *J* = 3.8, 1.1 Hz, 1H), 7.76 (dd, *J* = 5.0, 1.1 Hz, 1H), 7.70 – 7.66 (m, 2H), 7.42 – 7.36 (m, 1H), 7.25 – 7.21 (m, 2H), 7.22 – 7.15 (m, 3H), 2.35 (s, 3H). **^{13}C NMR** (126 MHz, $CDCl_3$) δ 180.7, 160.0 (d, *J* = 243.1 Hz), 146.3, 144.5, 137.4, 135.2, 133.8, 132.9, 132.1, 130.2, 129.7, 128.6, 127.3, 127.1, 126.5, 123.1 (q, *J* = 269.8 Hz), 116.3, 115.0 (d, *J* = 25.4 Hz), 112.5 (qd, *J* = 35.7, 4.3 Hz), 106.3 (dd, *J* = 25.7, 2.7 Hz), 21.7. **^{19}F NMR** (282 MHz, $CDCl_3$) δ −54.61 (s, 3F), −117.00 – −117.40 (m, 1F). **IR (KBr)**: 3047, 2930, 2856, 1658, 1612, 1593, 1516, 1475, 1452, 1392, 1300, 1242, 1120, 1088, 970, 915, 848, 812, 723, 667, 577, 544 cm^{-1}. **HRMS (ESI)** calculated for $C_{23}H_{15}F_4NO_3S_2Na$ $[M+Na]^+$: 516.0327, found: 516.0325.

1-Phenyl-3-(1-tosyl-3,6-bis(trifluoromethyl)-1*H*-indol-2-yl)prop-2-en-1-one (6ca):

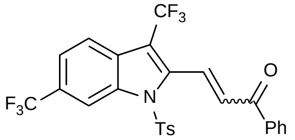

Following the general method **J**, the purification by column chromatography on silica gel (Toluene) to give **6ca** (31.2 mg, Yield: 58%) as a light yellow solid, m.p. = 118.9 – 119.7 °C. The ratio for *E/Z* isomers (1.2:1) was determined by ^{19}F NMR. (*E*)-**6ca**: **^{1}H NMR** (500 MHz, $CDCl_3$) δ 8.65 (s, 1H), 8.05 (dd, *J* = 15.9, 1.3 Hz, 1H), 8.02 – 7.99 (m, 2H), 7.87 (d, *J* = 8.4 Hz, 1H), 7.74 – 7.69 (m, 2H), 7.68 – 7.63 (m, 1H), 7.64 – 7.60 (m, 1H), 7.58 – 7.52 (m, 2H), 7.29 (dd, *J* = 15.9, 0.9 Hz, 1H), 7.25 (d, *J* = 8.1 Hz, 2H), 2.36 (s, 3H). **^{13}C NMR** (126 MHz, $CDCl_3$) δ 188.8, 146.7, 138.5, 136.9, 135.3, 134.6, 133.7, 133.0, 130.3, 130.0, 129.0, 128.8, 128.4, 127.6, 127.2, 124.3 (q, *J* = 251.8 Hz), 123.0 (q, *J* = 269.8 Hz), 121.5, 121.3, 112.3, 112.1 (q, *J* = 35.9 Hz), 21.7. **^{19}F NMR** (282 MHz, $CDCl_3$) δ −54.37 (s, 3F), -61.94 (s, 3F). **IR (KBr)**: 3066, 2960, 2927, 2858, 1674, 1621, 1597, 1492, 1404, 1329, 1282, 1227, 1174, 1124, 1053, 1010, 968, 812, 739, 665, 570, 546 cm^{-1}. **HRMS (ESI)** calculated for $C_{26}H_{17}F_6NO_3SNa$ $[M+Na]^+$: 560.0731, found: 560.0724.

1-(*p*-Tolyl)-3-(1-tosyl-3,6-bis(trifluoromethyl)-1*H*-indol-2-yl)prop-2-en-1-one (6cc)

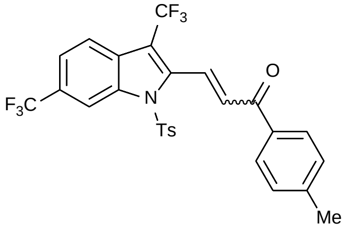

Following the general method **J**, the purification by column chromatography on silica gel (Toluene) to give **6cc** (29.8 mg, Yield: 60%) as a colorless oil, m.p. = 128.5 – 130.3 °C. The ratio for *E/Z* isomers (2.0:1) was determined by ^{19}F NMR. (*E*)-**6cc**: **^{1}H NMR** (500 MHz, $CDCl_3$) δ 8.66 (s, 1H), 8.04 (dd, *J* = 15.9, 1.4 Hz, 1H), 7.91 (d, *J* = 8.2 Hz, 2H), 7.87 (d, *J* = 8.5 Hz, 1H), 7.74 – 7.68 (m, 2H), 7.65 – 7.59 (m, 1H), 7.34 (d, *J* = 8.0 Hz, 2H), 7.27 (s, 1H), 7.25 (d, *J* = 8.8 Hz, 2H), 2.46 (s, 3H), 2.36 (s, 3H). **^{13}C NMR** (126 MHz, $CDCl_3$) δ 188.2, 146.6, 144.8, 138.7, 135.3, 134.6, 134.4, 133.2, 130.3, 130.0, 129.7, 129.4, 128.9, 128.5, 127.3, 124.2 (q, *J* = 272.3 Hz), 123.0 (q, *J* = 269.8 Hz), 121.2, 120.8, 112.4, 111.9 (q, *J* = 35.9 Hz), 21.8, 21.7. **^{19}F NMR** (282 MHz, $CDCl_3$) δ −54.37 (s, 3F), −61.91 (s, 3F). **IR (KBr)**: 3025, 2927, 2852, 1670, 1606, 1607, 1572, 1430, 1404, 1329, 1284, 1174, 1122, 1053, 968, 890, 821, 665, 567 cm^{-1}. **HRMS (ESI)** calculated for $C_{27}H_{19}F_6NO_3SNa$ $[M+Na]^+$: 574.0888, found: 574.0881.

3-(5-Chloro-1-tosyl-3-(trifluoromethyl)-1*H*-indol-2-yl)-1-phenylprop-2-en-1-one (6da):

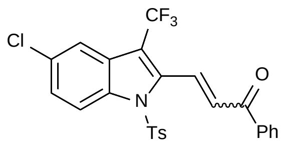

Following the general method **J**, the purification by column chromatography on silica gel (Toluene) to give **6da** (30.7 mg, Yield: 60%) as a light yellow solid, m.p. = 164.8 – 166.3 °C. The ratio for E/Z isomers (2.1:1) was determined by ^{19}F NMR. (*E*)-**6da**: **^{1}H NMR** (500 MHz, $CDCl_3$) δ 8.27 (d, *J* = 9.1 Hz, 1H), 8.04 (dd, *J* = 15.9, 1.4 Hz, 1H), 8.02 – 7.98 (m, 2H), 7.72 (s, 1H), 7.70 – 7.66 (m, 2H), 7.66 – 7.61 (m, 1H), 7.56 – 7.51 (m, 2H), 7.43 (dd, *J* = 9.0, 2.1 Hz, 1H), 7.28 (dd, *J* = 15.9, 1.0 Hz, 1H), 7.25 – 7.20 (m, 2H), 2.35 (s, 3H). **^{13}C NMR** (126 MHz, $CDCl_3$) δ 188.9, 146.4, 137.3, 137.0, 134.7, 134.5, 133.6, 132.6, 130.8, 130.4, 130.2, 129.9, 128.9, 128.8, 127.4, 127.1, 123.0 (q, *J* = 269.9 Hz), 120.2, 116.0, 111.9 (q, *J* = 35.7 Hz), 21.7. **^{19}F NMR** (282 MHz, $CDCl_3$) δ −54.48 (s, 3F). **IR (KBr)**: 3022, 2951, 2880, 1672, 1616, 1448, 1386, 1296, 1234, 1169, 1117, 1082, 1059, 798, 719, 663, 588 cm^{-1}. **HRMS (ESI)** calculated for $C_{25}H_{17}ClF_3NO_3SNa$ $[M+Na]^+$: 526.0467, found: 526.0465.

Methyl 2-(3-oxo-3-phenylprop-1-en-1-yl)-1-tosyl-3-(trifluoromethyl)-1*H*-indole-6-carboxylate (6ga):

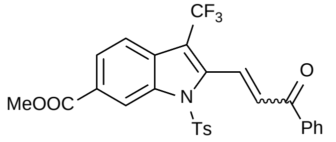

Following the general method **J**, the purification by column chromatography on silica gel (Toluene) to give **6ga** (21.0 mg, Yield: 40%) as a light yellow solid, m.p. = 132.3 – 133.0 °C. The ratio for *E/Z* isomers (1.2:1) was determined by ^{19}F NMR. (*E*)-**6ga**: **^{1}H NMR** (500 MHz, $CDCl_3$) δ 9.03 (s, 1H), 8.07 (dd, J = 15.9, 1.2 Hz, 1H), 8.03 – 8.00 (m, 2H), 7.83 – 7.80 (m, 2H), 7.76 – 7.71 (m, 2H), 7.66 – 7.62 (m, 1H), 7.57 – 7.52 (m, 2H), 7.30 (dd, *J* = 15.9, 0.9 Hz, 1H), 7.23 (d, *J* = 8.1 Hz, 2H), 4.01 (s, 3H), 2.35 (s, 3H). **^{13}C NMR** (126 MHz, $CDCl_3$) δ 188.9, 166.7, 146.4, 138.7, 136.9, 135.6, 134.7, 133.6, 132.8, 130.4, 130.2, 128.9, 128.8, 128.4, 127.6, 127.2, 125.7, 123.1 (q, *J* = 269.9 Hz), 120.5, 116.5, 112.3 (q, *J* = 35.8 Hz), 52.5, 21.7. **^{19}F NMR** (282 MHz, $CDCl_3$) δ −54.31 (s, 3F). **IR (KBr)**: 3050, 2960, 2921, 2848, 1722, 1674, 1611, 1596, 1492, 1402, 1297, 1273, 1171, 1118, 1052, 995, 907, 744, 701, 663, 580, 544 cm^{-1}. **HRMS (ESI)** calculated for $C_{27}H_{20}F_3NO_5SNa$ $[M+Na]^+$: 527.5142, found: 527.5139.

Methyl 2-(3-(4-nitrophenyl)-3-oxoprop-1-en-1-yl)-1-tosyl-3-(trifluoromethyl)-1*H*-indole-6-carboxylate (6gd):

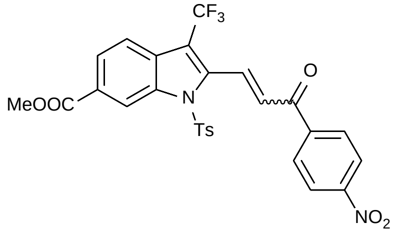

Following the general method **J**, the purification by column chromatography on silica gel (Toluene) to give **6gd** (26.0 mg, Yield: 45%) as a light yellow solid, m.p. = 173.0 – 174.1 °C. The ratio for *E/Z* isomers (4.0:1) was determined by ^{19}F NMR. (*E*)-**6gd**: **^{1}H NMR** (500 MHz, $CDCl_3$) δ 8.99 (s, 1H), 8.44 – 8.36 (m, 2H), 8.20 – 8.14 (m, 2H), 8.12 (dd, *J* = 15.9, 1.2 Hz, 1H), 8.09 – 8.03 (m, 1H), 7.83 – 7.77 (m z, 1H), 7.76 – 7.70 (m, 2H), 7.31 (d, *J* = 15.9 Hz, 1H), 7.27 – 7.24 (m, 2H), 4.02 (s, 3H), 2.37 (s, 3H). **^{13}C NMR** (126 MHz, $CDCl_3$) δ 187.8, 166.6, 150.5, 146.6, 141.5, 137.9 (q, *J* = 3.9 Hz), 135.7, 134.5, 132.2, 131.7 (q, *J* = 3.0 Hz), 130.3, 129.8, 128.6, 127.1, 125.9, 124.1, 123.0 (q, *J* = 270.0 Hz), 120.6 (q, *J* = 2.7 Hz), 116.6, 112.9 (q, *J* = 35.7 Hz), 52.6, 21.7. **^{19}F NMR** (282 MHz, $CDCl_3$) δ −54.21 (s, 3F). **IR (KBr)**: 3029, 2952, 2936, 2854, 1722, 1678, 1599, 1527, 1402, 1352, 1273, 1248, 1174, 1120, 1058, 993, 846, 746, 655, 580 cm^{-1}. **HRMS (ESI)** calculated for $C_{27}H_{19}F_3N_2O_7SNa$ $[M+Na]^+$: 595.0763, found: 595.0759.

3-(5,6-Dimethoxy-1-tosyl-3-(trifluoromethyl)-1*H*-indol-2-yl)-1-phenylprop-2-en-1-one (6ha):

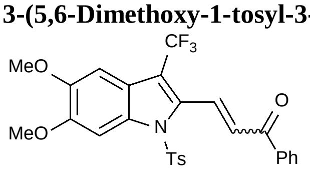

Following the general method **J**, the purification by column chromatography on silica gel (Toluene) to give **6ha** (35.4 mg, Yield: 67%) as a light yellow solid, m.p. = 155.2 – 157.5 °C. The ratio for *E/Z* isomers (6.0:1) was determined by ^{19}F NMR. (*E*)-**6ha**: **^{1}H NMR** (500 MHz, $CDCl_3$) δ 8.07 (dd, *J* = 15.9, 1.4 Hz, 1H), 8.03 – 7.98 (m, 2H), 7.86 (s, 1H), 7.66 – 7.59 (m, 3H), 7.53 (t, *J* = 7.7 Hz, 2H), 7.28 (d, *J* = 15.9 Hz, 1H), 7.19 (d, *J* = 8.1 Hz, 2H), 7.08 (s, 1H), 4.03 (s, 3H), 3.93 (s, 3H), 2.33 (s, 3H). **^{13}C NMR** (126 MHz, $CDCl_3$) δ 189.3, 149.7, 148.2, 146.0, 137.3, 134.8, 134.4, 133.3, 131.4, 130.9, 130.8, 130.0, 128.8, 128.7, 126.8, 123.4 (q, *J* = 269.8 Hz), 118.9, 113.4 (q, *J* = 35.3 Hz), 101.1, 98.1, 56.4, 56.1, 21.7. **^{19}F NMR** (282 MHz, $CDCl_3$) δ −54.69 (s, 3F). **IR (KBr)**: 3021, 2937, 2838, 1670, 1608, 1493, 1477, 1439, 1377, 1298, 1209, 1172, 1115, 1063, 1014, 981, 910, 850, 733, 665, 577, 542 cm^{-1}. **HRMS (ESI)** calculated for $C_{27}H_{22}F_3NO_5SNa$ $[M+Na]^+$: 552.1068, found: 552.1059.

3-(5,6-Dimethoxy-1-tosyl-3-(trifluoromethyl)-1*H*-indol-2-yl)-1-(4-methoxyphenyl) pro*p*-2-en-1-one (6hb):

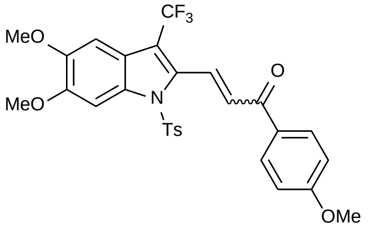

Following the general method **J**, the purification by column chromatography on silica gel (Toluene) to give **6hb** (33.5 mg, Yield: 60%) as a light yellow solid, m.p. = 141.9 – 144.6 °C. The ratio for *E/Z* isomers (5.3:1) was determined by ^{19}F NMR. (*E*)-**6hb**: **^{1}H NMR** (500 MHz, $CDCl_3$) δ 8.04 (d, *J* = 15.9 Hz, 1H), 8.03 – 7.99 (m, 2H), 7.87 (s, 1H), 7.65 – 7.61 (m, 2H), 7.31 (d, *J* = 16.0 Hz, 1H), 7.24 – 7.20 (m, 2H), 7.11 (s, 1H), 7.05 – 7.02 (m, 2H), 4.04 (s, 3H), 3.94 (s, 3H), 3.91 (s, 3H), 2.34 (s, 3H). **^{13}C NMR** (126 MHz, $CDCl_3$) δ 187.5, 163.8, 149.6, 148.1, 145.9, 134.9, 134.7, 131.3, 131.1, 130.7, 130.3, 130.0, 126.9, 123.5 (q, *J* = 269.8 Hz), 118.9, 114.0, 113.1 (q, *J* = 35.2 Hz), 101.1, 98.1, 56.4, 56.1, 55.6, 21.7. **^{19}F NMR** (282 MHz, $CDCl_3$) δ −54.69 (s, 3F). **IR (KBr)**: 3010, 2937, 2837, 2578, 1664, 1599, 1572, 1491, 1377, 1307, 1259, 1209, 1170, 1116, 1109, 1062, 1019, 914, 839, 733, 665, 577 cm^{-1}. **HRMS (ESI)** calculated for $C_{28}H_{24}F_3NO_6SNa$ [M+Na]$^+$: 582.1174, found: 582.1165.

4-Methyl-*N*-(2-(4-methylpent-3-en-1-yn-3-yl)phenyl)benzenesulfonamide (**5ha**):

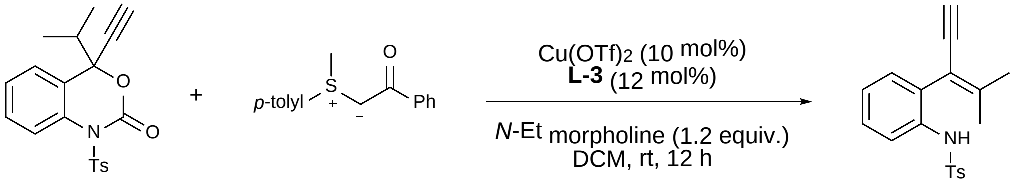

3h **2a'** **5ha**

Scheme S1. Reaction of 4-isopropyl benzoxazinanones with sulfur ylides, related to **Figure 2**

Following the general method **I**, compound **5ha** was obtained as a white solid (17.9 mg, Yield: 55%), m.p. = 88.4 – 90.2 °C. **^{1}H NMR** (500 MHz, $CDCl_3$) δ 7.67 – 7.62 (m, 3H), 7.28 – 7.22 (m, 1H), 7.20 – 7.15 (m, 2H), 7.06 (td, *J* = 7.5, 1.2 Hz, 1H), 7.01 – 6.94 (m, 2H), 3.18 (s, 1H), 2.35 (s, 3H), 2.04 (s, 3H), 1.31 (s, 3H). **^{13}C NMR** (126 MHz, $CDCl_3$) δ 149.9, 143.6, 136.6, 134.2, 130.4, 129.8, 129.4, 128.6, 127.3, 124.8, 121.5, 112.7, 82.0, 82.0, 23.2, 21.5, 21.0. **IR (KBr)**: 3270, 1486, 1400, 1330, 1160, 1093, 929, 761, 661, 541 cm^{-1}. **HRMS (ESI)** calculated for $C_{19}H_{19}NO_2SNa$ [M+Na]$^+$: 348.1034, found: 348.1029.

5-Tosyl-5,6-dihydroindeno[2,1-b]indole (**5ia**) (Yamashiro et al., 2019):

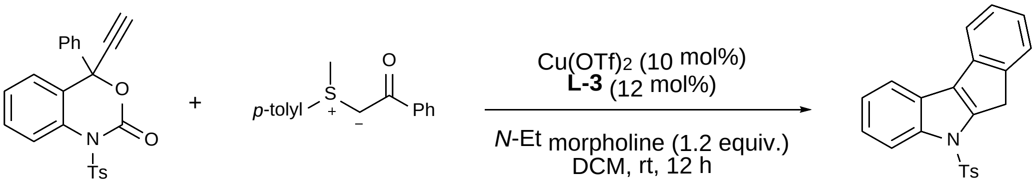

3i **2a'** **5ia**

Scheme S2. Reaction of 4-phenyl benzoxazinanones with sulfur ylides, related to **Figure 2**

Following the general method **I**, compound **5ia** was obtained as a white solid (16.5 mg, Yield: 23%), m.p. = 167.0 – 168.0 °C. **^{1}H NMR** (500 MHz, $CDCl_3$) δ 8.14 – 8.09 (m, 1H), 7.80 – 7.73 (m, 3H), 7.64 (dt, *J* = 7.5, 0.9 Hz, 1H), 7.52 (dt, *J* = 7.5, 1.0 Hz, 1H), 7.38 – 7.31 (m, 3H), 7.22 (td, *J* = 7.5, 1.1 Hz, 1H), 7.17 (d, *J* = 8.2 Hz, 2H), 4.10 (s, 2H), 2.29 (s, 3H). **^{13}C NMR** (126 MHz, $CDCl_3$) δ 145.35, 145.17, 143.08, 139.87, 137.62, 135.21, 129.95, 127.52, 127.05, 126.46, 124.85, 124.76, 124.41, 124.03, 123.82, 119.72, 119.47, 114.57, 34.04, 21.54. **HRMS (ESI)** calculated for $C_{22}H_{17}NO_2SNa$ [M+Na]$^+$: 382.0878, found: 382.0875.

Synthetic transformation:

((2S,3R)-3-Methyl-1-tosyl-3-(1-tosyl-1*H*-1,2,3-triazol-4-yl)indolin-2-yl)(phenyl)methanone (7):

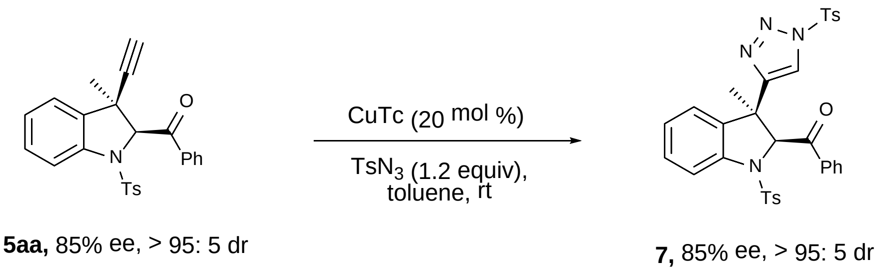

Scheme S3. Cycloaddition reactions of **5aa** with tosylazide, related to **scheme 5**

Under argon atmosphere, a flame-dried 10 mL Schlenk tube was charged with **5aa** (41.5 mg, 0.1 mmol, 85% *ee*, 95:5 dr), copper(I) thiophene-2-carboxylate (CuTc, 3.8 mg, 0.02 mmol, 20 mol %) and anhydrous toluene (1.0 mL). The resulting solution was cooled to 0 °C in an ice-water bath. Subsequently, the tosylazide (23.7 mg, 0.12 mmol, 1.2 equiv.) was added slowly. The resulting solution could warm to room temperature and stirred for 5h. The reaction was quenched by saturated NH_4Cl aqueous solution (2 mL) and extracted with EtOAc (3×5 mL). The combined organic layers were dried over Na_2SO_4, filtered and concentrated in *vacuo*. The dr value was determined by ^1H NMR analysis of the crude reaction mixture. Then the residue was purified by flash silica gel chromatography (PE/EA = 7/3) to afford the title compound **7** as a white solid (60.6 mg, 99% yield). m.p. = 148.4 – 149.0 °C, the enantiomeric excess (85% *ee*) was determined by chiral HPLC using CHIRALPAK® IC (*n*-hexane/isopropanol = 85.0/15.0, flow rate 1.0 mL/min, λ = 254 nm) t (major) = 64.408 min, t (minor) = 77.175 min). $[\alpha]^{25}_D$ = +36.40 (c = 1.78, $CHCl_3$, 85% *ee*). **^{1}H NMR** (500 MHz, $CDCl_3$) δ 7.83 – 7.75 (m, 4H), 7.70 – 7.65 (m, 1H), 7.51 (s, 1H), 7.40 – 7.27 (m, 8H), 7.16 – 7.08 (m, 2H), 7.08 – 7.01 (m, 1H), 6.87 – 6.80 (m, 1H), 5.68 (s, 1H), 2.49 (s, 3H), 2.41 (s, 3H), 1.60 (s, 3H). **^{13}C NMR** (126 MHz, $CDCl_3$) δ 195.0, 147.9, 147.1, 144.6, 141.3, 136.4, 135.4, 135.2, 133.3, 132.9, 130.4, 129.9, 129.5, 128.6, 128.4, 128.0, 127.2, 124.3, 123.6, 123.1, 114.6, 74.7, 48.5, 28.9, 21.9, 21.6. **IR (KBr)**: 3124, 1693, 1598, 1392, 1355, 1170, 1093, 1006, 964, 809, 669, 590, 543 cm^{-1}. **HRMS (ESI)** calculated for $C_{32}H_{28}N_4O_5NaS_2$ $[M+Na]^+$: 635.1399, found: 635.1400.

((2S,3R)-3-methyl-3-(phenylethynyl)-1-tosylindolin-2-yl)(phenyl)methanone (8):

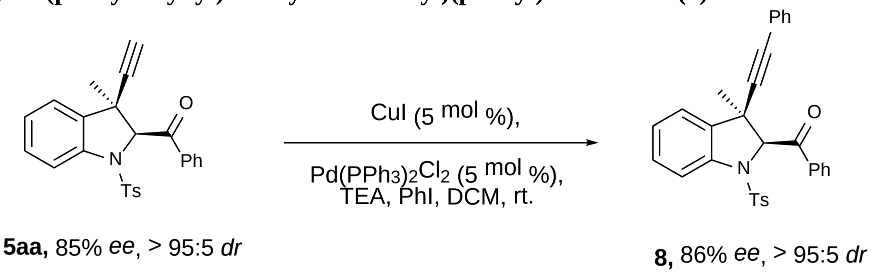

Scheme S4. Cross-coupling reaction of **5aa** with iodobenzene, related to **scheme 5**

Under argon atmosphere, a flame-dried Schlenk tube was charged with **5aa** (83 mg, 0.20 mmol, 85% ee), iodobenzene (49 mg, 0.24 mmol, 1.2 equiv.), $Pd(PPh_3)_2Cl_2$ (7.0 mg, 0.01 mmol, 5 mol %), CuI (1.9 mg, 0.01 mmol, 5 mol %), then anhydrous DCM (5 mL) and Et_3N (1 mL) were added. The resulting solution was stirred at room temperature for 5h. The reaction was quenched by saturated NH_4Cl aqueous solution (10 mL) and extracted with CH_2Cl_2 (3×10 mL). The combined organic layers were washed with water and brine, then dried over Na_2SO_4, filtrated, and concentrated under vacuum. The residue was purified by silica gel column chromatography (PE/EtOAc = 20/1) to afford the desired product **8** (68.8 mg, yield: 70 %) as white solid. m.p. = 145.4 – 146.6 °C. The enantiomeric excess (86% *ee*) was determined by chiral HPLC using CHIRALPAK® IB IB (*n*-hexane/isopropanol = 98.0/2.0, flow rate 1.0 mL/min, λ = 254 nm) t (major) = 45.333 min, t (minor) = 58.517 min). $[\alpha]^{25}_D$ = −52.97 (c = 0.8 in $CHCl_3$). **^{1}H NMR** (500 MHz, $CDCl_3$) δ 8.01 – 7.96 (m, 2H), 7.81 – 7.77 (m, 2H), 7.63 – 7.55 (m, 2H), 7.49 – 7.43 (m, 2H), 7.32 – 7.25 (m, 4H), 7.19 – 7.14 (m, 1H), 7.12 – 7.06 (m, 3H), 6.82 – 6.77 (m, 2H), 5.53 (s, 1H), 2.40 (s, 3H), 1.43 (s, 3H). **^{13}C NMR** (126 MHz, $CDCl_3$) δ 194.0, 144.5, 140.2, 136.5, 135.9, 135.4, 133.2, 131.3, 129.8, 129.2, 129.0, 128.6, 128.2, 127.9, 127.3, 124.4, 124.0, 122.1, 114.6, 89.0, 86.2, 74.5, 44.5, 31.9, 21.6. **IR (KBr)**: 2981, 1698, 1596, 1479, 1355, 1213, 1168, 1091, 759, 717, 671, 588, 566 cm^{-1}. **HRMS (ESI)** calculated for $C_{31}H_{25}NO_3SNa$ $[M+Na]^+$: 514.1453, found: 514.1461.

Conformation transformation reactions of 6

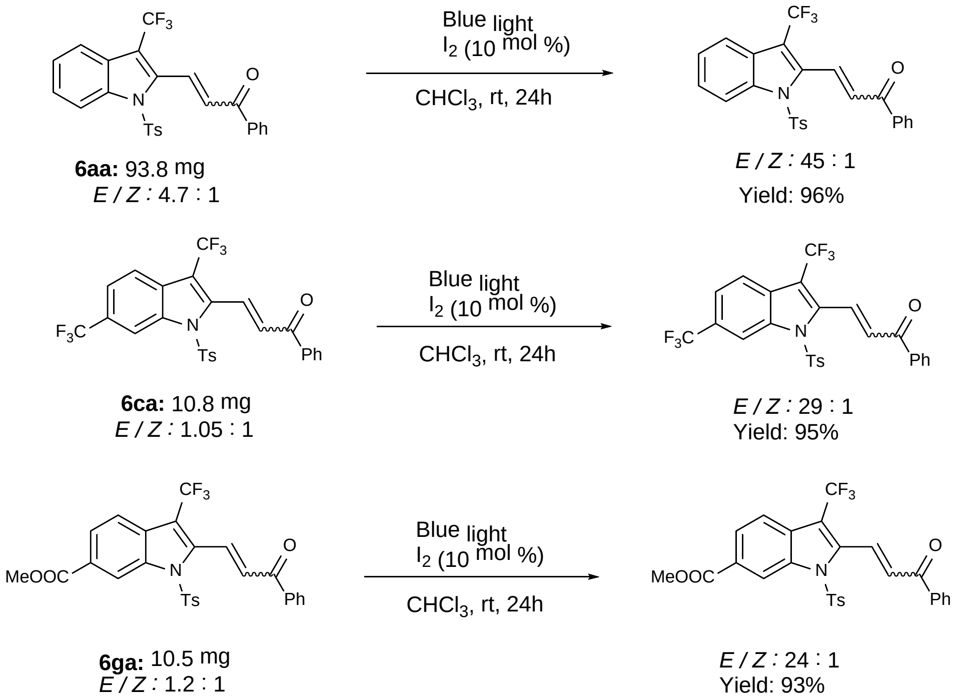

Scheme S5. Conformation transformation reactions of **6**, related to **scheme 7a**

Fellow the literature procedure (Clark et al., 2008), an oven-dried tube was charged with **6**, Iodine (10 mol%) and anhydrous $CHCl_3$. The tube was sealed, and the resulting solution was stirred and irradiated using 7 W blue LED lamps (with cooling fan to keep the reaction at room temperature) for 24 h. The resulting solution were then taken ^{19}F NMR, and dried and isolated to give the corresponding yield.

(*E*)-6aa: ^{1}H NMR (500 MHz, $CDCl_3$) δ 8.33 (d, *J* = 8.6 Hz, 1H), 8.08 (dd, *J* = 15.9, 1.3 Hz, 1H), 8.04 – 7.98 (m, 2H), 7.75 (d, *J* = 8.0 Hz, 1H), 7.72 – 7.67 (m, 2H), 7.66 – 7.60 (m, 1H), 7.57 – 7.51 (m, 2H), 7.50 – 7.44 (m, 1H), 7.41 – 7.34 (m, 1H), 7.28 (d, *J* = 16.0 Hz, 1H), 7.21 (d, *J* = 7.8 Hz, 2H), 2.34 (s, 3H). **^{13}C NMR** (126 MHz, $CDCl_3$) δ 189.2, 146.0, 137.1, 136.3, 136.0 (q, *J* = 4.0 Hz), 135.0, 133.5, 132.1, 131.0, 130.1, 128.8, 128.8, 127.0, 126.7, 125.6, 124.9, 123.3 (q, *J* = 269.8 Hz), 120.7, 114.9, 112.8 (q, *J* = 35.3 Hz), 21.6.

(*E*)-6ca: ^{1}H NMR (500 MHz, $CDCl_3$) δ 8.65 (s, 1H), 8.05 (dd, *J* = 15.9, 1.3 Hz, 1H), 8.02 – 7.98 (m, 2H), 7.87 (d, *J* = 8.7, 1H), 7.73 – 7.69 (m, 2H), 7.65 (t, *J* = 7.4 Hz, 1H), 7.62 (dd, *J* = 8.6, 1.6 Hz, 1H), 7.55 (t, *J* = 7.7 Hz, 2H), 7.29 (dd, *J* = 15.9, 1.0 Hz, 1H), 7.25 (d, *J* = 8.1 Hz, 2H), 2.36 (s, 3H).

(*E*)-6ga: ^{1}H NMR (500 MHz, $CDCl_3$) δ 9.03 (d, *J* = 0.7 Hz, 1H), 8.07 (dd, *J* = 15.9, 1.2 Hz, 1H), 8.05 (d, *J* = 1.5 Hz, 1H), 8.03 – 7.99 (m, 2H), 7.80 (d, *J* = 8.5 Hz, 1H), 7.76 – 7.71 (m, 2H), 7.65 (t, *J* = 7.4 Hz, 1H), 7.55 (t, *J* = 7.6 Hz, 2H), 7.29 (d, *J* = 15.9 Hz, 1H), 7.23 (d, *J* = 8.0 Hz, 2H), 4.01 (s, 3H), 2.35 (s, 3H).

Phenyl-2-(1-tosyl-3-(trifluoromethyl)-1*H*-indol-2-yl)cyclopropyl)methanone (11) (Makarov et al., 2018)**:**

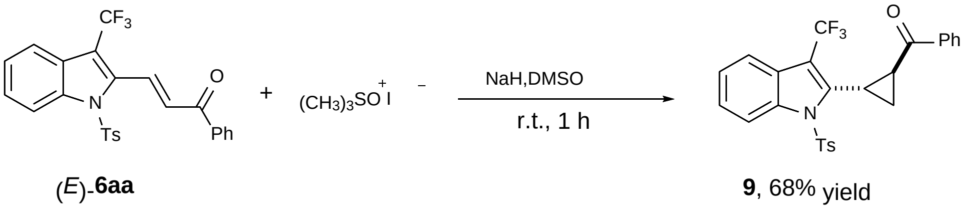

Scheme S6. Cyclopropanation reaction of (*E*)-**6aa**, related to **scheme 7b**

Under argon atmosphere, a suspension of NaH (60% w/w in mineral oil, 6 mg, 0.3 mmol, 1.5 equiv) and trimethylsulfoxonium iodide (33 mg, 0.3 mmol, 1.5 equiv) in DMSO (2 mL) was stirred at 20 °C for 0.5 h followed by dropwise addition of the solution of indole **6aa** (94 mg, 0.2 mmol, 1 equiv) in DMSO (2 mL) at room temperature.

The resulted suspension was stirred for 1 h, then quenched with saturated aqueous solution of NH_4Cl (5 mL). Ethyl acetate (25 mL) was added, the organic phase was separated, washed with brine (30 mL), dried over Na_2SO_4 and concentrated under reduced pressure. The residue was purified by silica gel column chromatography (Hexane/EtOAc = 20/1) to afford the desired product **9** (66 mg, yield: 68 %) as a white solid, m.p. = 139.4 – 140.6 °C. **^{1}H NMR** (500 MHz, $CDCl_3$) δ 8.29 – 8.25 (m, 1H), 8.13 – 8.03 (m, 2H), 7.65 (d, *J* = 7.9 Hz, 1H), 7.63 – 7.56 (m, 3H), 7.54 – 7.47 (m, 2H), 7.43– 7.38 (m, 1H), 7.35 – 7.30 (m, 1H), 7.21 – 7.15 (m, 2H), 3.24 – 3.14 (m, 1H), 2.98 – 2.87 (m, 1H), 2.34 (s, 3H), 1.90 – 1.80 (m, 1H), 1.71 – 1.61 (m, 1H). **^{13}C NMR** (126 MHz, $CDCl_3$) δ 198.7, 145.6, 140.5, 137.4, 136.2, 135.3, 133.1, 130.0, 128.6, 128.4, 126.6, 126.0, 125.4, 124.6, 123.5 (q, *J* = 269.6 Hz), 120.0, 115.3, 114.1 (q, *J* = 35.7 Hz), 27.2 (2), 21.6, 21.1, 20.2. **^{19}F NMR** (282 MHz, $CDCl_3$) δ -54.6 (s, 3F). **IR (KBr)**: 3059, 2960, 2922, 2873, 1672, 1597, 1479, 1450, 1390, 1342, 1225, 1178, 1124, 1061, 1001, 954, 912, 748, 717, 665, 574 cm^{-1}. **HRMS (ESI)** calculated for $C_{26}H_{20}$ F_3NO_3SNa $[M+Na]^+$: 506.1014, found: 506.1024.

***(E)*-1,1,1-Trifluoro-2-phenyl-4-(1-tosyl-3-(trifluoromethyl)-1*H*-indol-2-yl)but-3-en-2-ol (10)** (Cheng et al., 2013)**:**

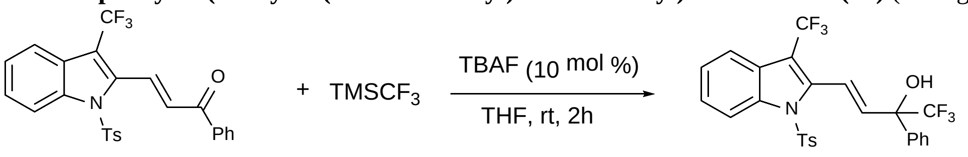

***(E)*-6aa** **10**, 97% yield

Scheme S7. Trifluoromethylation reaction of (*E*)-**6aa**, related to **scheme 7b**

In a flame dried tube, ***(E)*-6aa** (0.1 mmol, 47 mg, 1.0 equiv.) and $TMSCF_3$ (neat, 0.2 mmol, 29 µL, 2.0 equiv.) was suspended in anhydrous THF (2 mL) then cooled to 0 °C. After 10 min TBAF (1.0 M in THF, 10 µL. 0.01 equiv.) was then added. and the mixture was stirred vigorously at room temperature under N_2 atmosphere. After completion of the reaction, aqueous HCl solution (2 M, 0.5 mL) was added and stirred for 30 min at room temperature. The reaction mixture was then extracted with ethyl acetate (3 × 5 mL) and purified by column chromatography (Hexane/EtOAc = 10/1) to afford the pure product **10** as a light-yellow oil (52.3 mg, Yield: 97 %). **^{1}H NMR** (500 MHz, $CDCl_3$) δ 8.26 (d, *J* = 8.6 Hz, 1H), 7.74 – 7.62 (m, 3H), 7.55 – 7.49 (m, 2H), 7.49 – 7.39 (m, 4H), 7.36 – 7.28 (m, 2H), 7.02 (d, *J* = 8.2 Hz, 2H), 6.51 (d, *J* = 16.0 Hz, 1H), 3.04 (s, 1H), 2.29 (s, 3H). **^{13}C NMR** (126 MHz, $CDCl_3$) δ 145.8, 136.6, 136.5, 136.1, 135.5, 134.9, 129.9, 129.1, 128.60, 126.8, 126.5, 126.1, 125.6, 124.69 (q, *J* = 286.2 Hz), 124.6, 123.28 (q, *J* = 269.7 Hz), 121.5, 120.4, 114.6, 111.7 (q, *J* = 35.1 Hz), 77.31 (d, *J* = 29.3 Hz), 21.6. **^{19}F NMR** (282 MHz, $CDCl_3$) δ −54.3 (s, 3F), −78.7 (s, 3F). **IR (KBr)**: 3508, 3066, 2960, 2933, 2869, 1597, 1479, 1452, 1396, 1309, 1248, 1170, 1089, 1062, 974, 910, 742, 730, 669, 574 cm^{-1}. **HRMS (ESI)** calculated for $C_{26}H_{19}$ F_6NO_3SNa $[M+Na]^+$: 562.0888, found: 562.0891.

1-Phenyl-3-(1-tosyl-3-(trifluoromethyl)-1*H*-indol-2-yl)propan-1-one (11) (Cui et al., 2018)**:**

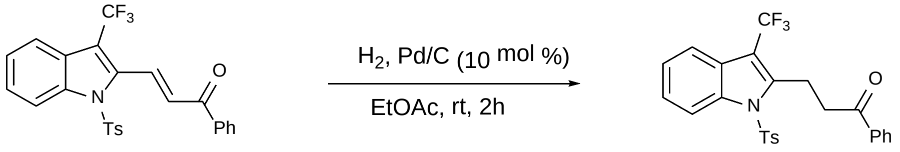

***(E)*-6aa** **11**, 87% yield

Scheme S8. Reduction reaction of (*E*)-**6aa**, related to **scheme 7b**

An oven-dried tube was charged with **6aa** (0.2 mmol, 94 mg, 1.0 equiv) and Pd/C (10% wt Palladium on carbon, 2mg, 0.1 equiv.) was dissolved in EtOAc at room temperature, then vacuum and refilled with N_2 for 3 times, the reaction was then performed under H_2 balloon conditions for 2 h. Completion of the reaction was monitored by TLC. Then mixture was filtered and removed by reduced pressure to afford the crude mixture. The crude product was purified by flash column chromatography (Hexane/EtOAc = 10/1) to obtain the pure product **11** as a light-yellow oil (81.9 mg, Yield: 87 %). **^{1}H NMR** (500 MHz, $CDCl_3$) δ 8.31 (d, *J* = 8.5 Hz, 1H), 8.01 – 7.93 (m, 2H), 7.76 – 7.70 (m, 2H), 7.67 (d, *J* = 7.8 Hz, 1H), 7.60 – 7.53 (m, 1H), 7.50 – 7.43 (m, 2H), 7.43 – 7.36 (m, 1H), 7.36 – 7.30 (m, 1H), 7.27 – 7.21 (m, 2H), 3.62 – 3.53 (m, 2H), 3.49 – 3.41 (m, 2H), 2.35 (s, 3H). **^{13}C NMR** (126 MHz, $CDCl_3$) δ 197.8, 145.8, 141.4 (q, *J* = 4.0 Hz), 136.4, 135.9, 135.5, 133.3, 130.3, 128.6, 128.1, 126.5, 125.4, 124.5, 123.9 (q, J = 269.4 Hz), 119.7 (q,

J = 2.3 Hz), 114.8, 111.4 (q, *J* = 34.8 Hz), 39.7, 21.6, 21.3. **^{19}F NMR** (282 MHz, $CDCl_3$) δ −56.1 (s, 3F). **IR (KBr)**: 3062, 3028, 2925, 2864, 1687, 1597, 1479, 1450, 1400, 1375, 1288, 1236, 1176, 1116, 1056, 973, 812, 742, 692, 671, 574 cm^{-1}. **HRMS (ESI)** calculated for $C_{25}H_{20}F_3NO_3SNa$ $[M+Na]^+$: 494.1014, found: 494.1016.

Supplemental References:

Allendörfer, N., Es-Sayed, M., Nieger, M., and Bräs, S. (2012). Nucleophilic ring-opening reaction of benzoxazinones-access to *o*-amino-2,2,2-trifluoroacetophenones. Tetrahedron Lett. *53*, 388-391.

Anderson, W.K., and Jones, A.N. (1984). Synthesis and evaluation of furan, thiophene, and azole bis[(carbamoyloxy)methyl] derivatives as potential antineoplastic agents. J. Med. Chem. *27*, 1559-1565.

Cheng, H.C., Pei, Y., Leng, F.Q., and Wu, Y.S. (2013). Highly efficient synthesis of aryl and heteroaryl trifluoromethyl ketones via o-iodobenzoic acid (IBX). Tetrahedron Lett. *54*, 4483-4486.

Clark, D.A., Clark, J.R., Diver, S.T. (2008). Alkenol−Alkyne Cross Metathesis. Org. Lett. *10*, 2055-2058.

Cui, B.Q., Jia, S.C., Tokunaga, E., Shibata, N. (2018). Defluorosilylation of fluoroarenes and fluoroalkanes. Nature Comm. 9, 4393.

Huang, Z.X., Yang, Y., Xiao, Q., Zhang, Y., and Wang, J.B. (2012). Auto-tandem catalysis: synthesis of acridines by Pd-catalyzed C=C bond formation and C(sp2)-N cross-coupling. Eur. J. Org. Chem. 6586-6593.

Kehler, J., Kilburn, J.P., Nielsen, J., Puschl, A., Langgard, M., Jessing, M. (2013). Preparation of quinazoline derivatives for use as PDE10A enzyme inhibitors. Patent. WO/2013/050527.

Kim, D.H., Yun, B.H., and Lee, Y.S. (2013). Formal Synthesis of Fesoterodine by Acid□Facilitated Aromatic Alkylation. Bull. Korean Chem. Soc. *34*, 2507-2510.

Kumar, Y.K., Kumar, G.R., Reddy, T.J., Sridhar, B., and Reddy, M.S. (2015). Synthesis of 3-Sulfonylamino Quinolines from 1-(2-Aminophenyl) Propargyl Alcohols through a Ag(I)-Catalyzed Hydroamination, (2 + 3) Cycloaddition, and an Unusual Strain-Driven Ring Expansion. Org. Lett. *17*, 2226-2229.

Lu, S.C., Ong, J.Y., Poh, S.B., Tsang, T., and Zhao, Y. (2018). Transition‐metal‐free decarboxylative propargylic substitution/cyclization with either azolium enolates or acyl anions. Angew. Chem. Int. Ed. Enｇｌ. *57*, 5714-5719.

Makarov, A. S., Uchuskin, M. G., Gevorgyan, V. (2018). Intramolecular Palladium-Catalyzed Oxidative Amination of Furans: Synthesis of Functionalized Indoles J. Org. Chem. *83*, 14010−14021.

Payne, G. B. (1967). Cyclopropanes from reactions of ethyl dimethylsulfuranylideneacetate with. alpha.,.beta. -unsaturated compounds. J. Org. Chem. *32*, 3351-3355.

Punna, N., Harada, K., Zhou, J., and Shibata. N. (2019). Pd-Catalyzed Decarboxylative Cyclization of Trifluoromethyl Vinyl Benzoxazinanones with Sulfur Ylides: Access to Trifluoromethyl Dihydroquinolines. Org. Lett. *21*, 1515-1520.

Quintana, J., Torres, M., and Serratosa, F. (1973). Decomposition of diazoketones in organic sulfides and sulfoxides: Cyclopropane formation from diazoketones via sulfonium ylides. Tetrahedron *29*, 2065-2076.

Ratts, K.W., and Yao, A.N. (1966). Stable Sulfonium Ylids. J. Org. Chem. *31*, 1185-1188.

Song, W.Z., Li, M., He, J.N., Li, J.H, Dong, K., and Zheng, Y.B. (2019). Copper catalyzed tandem annulation/enol nucleophilic addition to access multisubstituted indoles. Org. Biomol. Chem.*17*, 2663-2669.

Søren, K., and Troels, S. (2012). Gold‐catalyzed carbene transfer to alkynes: access to 2,4‐disubstituted furans. Angew. Chem. Int. Ed. Enｇｌ. *51*, 4681-4684.

Sun, Y.L., Wei, Y., and Shi, M. (2017). Tunable regiodivergent phosphine-catalyzed [3 + 2] cycloaddition of alkynones and trifluoroacetyl phenylamides. Org. Chem. Front. *4*, 2392-2402.

Sun, Y.L., Wei, Y., and Shi, M. (2017). Tunable regiodivergent phosphine-catalyzed [3 + 2] cycloaddition of alkynones and trifluoroacetyl phenylamides. Org. Chem. Front. *4*, 2392-2402.

Wang, B.C., Wang, Y. N., Zhang, M.M., Xiao, W.J., and Lu, L.Q. (2018). Copper-catalyzed decarboxylative cyclization *via* tandem C-P and C-N bond formation: access to 2-phosphorylmethyl indoles. Chem. Comm. *54*, 3154-315.

Xia, H.D., Zhang, Y.D., Wang, Y.H., and Zhang, C. (2018). Water-Soluble Hypervalent Iodine(III) Having an I-N Bond. A Reagent for the Synthesis of Indoles. Org. Lett. *20*, 4052-4056.

Yamashiro, T., Yamada, K., Yoshida, H., Tomisaka, Y., Nishi, T., Abe, T. (2019). Silver-Mediated Intramolecular Friedel–Crafts-Type Cyclizations of 2-Benzyloxy-3-bromoindolines: Synthesis of Isochromeno[3,4-b] indolines and 3-Arylindoles. Synlett., *30*, 2247-2252.

Yasuhara, A., Kameda, M., and Sakamoto, T. (1999). Selective monodesulfonylation of N, N-disulfonylarylamines with tetrabutylammonium fluoride. Chem. Pharm. Bull. *47*, 809-812.
